# Supplementary material for: Risk factor associations for severe COVID-19, influenza and pneumonia in people with diabetes to inform future pandemic preparations: UK population-based cohort study
Source: BMJ Open. 2024 Jan 31;14(1):e078135. doi: 10.1136/bmjopen-2023-078135 (PMC10831438; doi:10.1136/bmjopen-2023-078135)
Supplement: Supplementary data [file bmjopen-2023-078135supp001.pdf]

Supplemental material

Table of Contents

**Page**	
**2**	Supplemental figure 1. Flow diagram of study participants and exclusions.
**3**	Supplemental table 1. ICD-10 codes used to define Covid-19, influenza, and pneumonia outcomes.
**4**	Supplemental table 2. Definitions of exposure variables.
	**Type 2 diabetes (hospitalisation)**
**5**	Supplemental table 3. Frequency of ICD-10 codes recorded in hospitalisation outcomes in type 2 diabetes for Covid-19, influenza, and pneumonia.
**6-7**	Supplemental table 4. Full baseline characteristics and Covid-19, influenza, and pneumonia hospitalisations in type 2 diabetes.
**8**	Supplemental figure 2. Association of potential risk factors with hospitalisation for Covid-19, influenza, and pneumonia in type 2 diabetes.
**9-10**	Supplemental figure 5. Hazard ratio values for the association of potential risk factors with hospitalisation for Covid-19, influenza, and pneumonia in type 2 diabetes.
**11**	Supplemental figure 3A. Association of continuous HbA1c with hospitalisation for Covid-19, influenza, and pneumonia in type 2 diabetes by ethnicity subgroup.
**11**	Supplemental figure 3B. Association of continuous BMI with hospitalisation for Covid-19, influenza, and pneumonia in type 2 diabetes by ethnicity subgroup.
	**Type 2 diabetes (mortality)**
**12-13**	Supplemental table 6. Baseline characteristics and Covid-19 and pneumonia deaths in type 2 diabetes.
**14**	Supplemental figure 4. Association of potential risk factors with Covid-19, and pneumonia mortality in type 2 diabetes.
	**Type 1 diabetes**
**15**	Supplemental table 7. Baseline characteristics and Covid-19, influenza, and pneumonia hospitalisations in type 1 diabetes.
**16**	Supplemental figure 5. Association of potential risk factors with hospitalisation for Covid-19, influenza, and pneumonia in type 1 diabetes.
**17**	Supplemental figure 6A. Association of continuous HbA1c with hospitalisation for Covid-19, influenza, and pneumonia in type 1 diabetes.
**17**	Supplemental figure 6B. Association of continuous BMI with hospitalisation for Covid-19, influenza, and pneumonia in type 1 diabetes.
	**Sensitivity analyses**
**18**	Supplemental table 8. Baseline characteristics and Covid-19, influenza, and pneumonia hospitalisations in type 1 diabetes, restricting hospitalisation definition to primary diagnosis in HES.
**19**	Supplemental figure 7. Association of potential risk factors with hospitalisation for Covid-19, influenza, and pneumonia in type 1 diabetes, restricting hospitalisation definition to primary diagnosis in HES.
**20-21**	Supplemental table 9. Baseline characteristics and Covid-19, influenza, and pneumonia hospitalisations in type 2 diabetes, restricting hospitalisation definition to primary diagnosis in HES.
**22**	Supplemental figure 8. Association of potential risk factors with hospitalisation for Covid-19, influenza, and pneumonia in type 2 diabetes, restricting hospitalisation definition to primary diagnosis in HES.
**23-24**	Supplemental table 10. Baseline characteristics and Covid-19 and pneumonia deaths in type 2 diabetes, restricting mortality definition to primary cause in ONS.
**25**	Supplemental figure 9. Association of potential risk factors with Covid-19, and pneumonia mortality in type 2 diabetes, restricting mortality definition to primary cause in ONS.
**26**	Supplemental figure 10. Association of potential risk factors with pneumonia hospitalisation in those with a pneumococcal vaccination and those without.
**27**	Supplemental figure 11. Association of potential risk factors with influenza hospitalisation in those with an influenza vaccination and those without.
**28**	References

Supplemental figure 1. Flow diagram of study participants and exclusions.

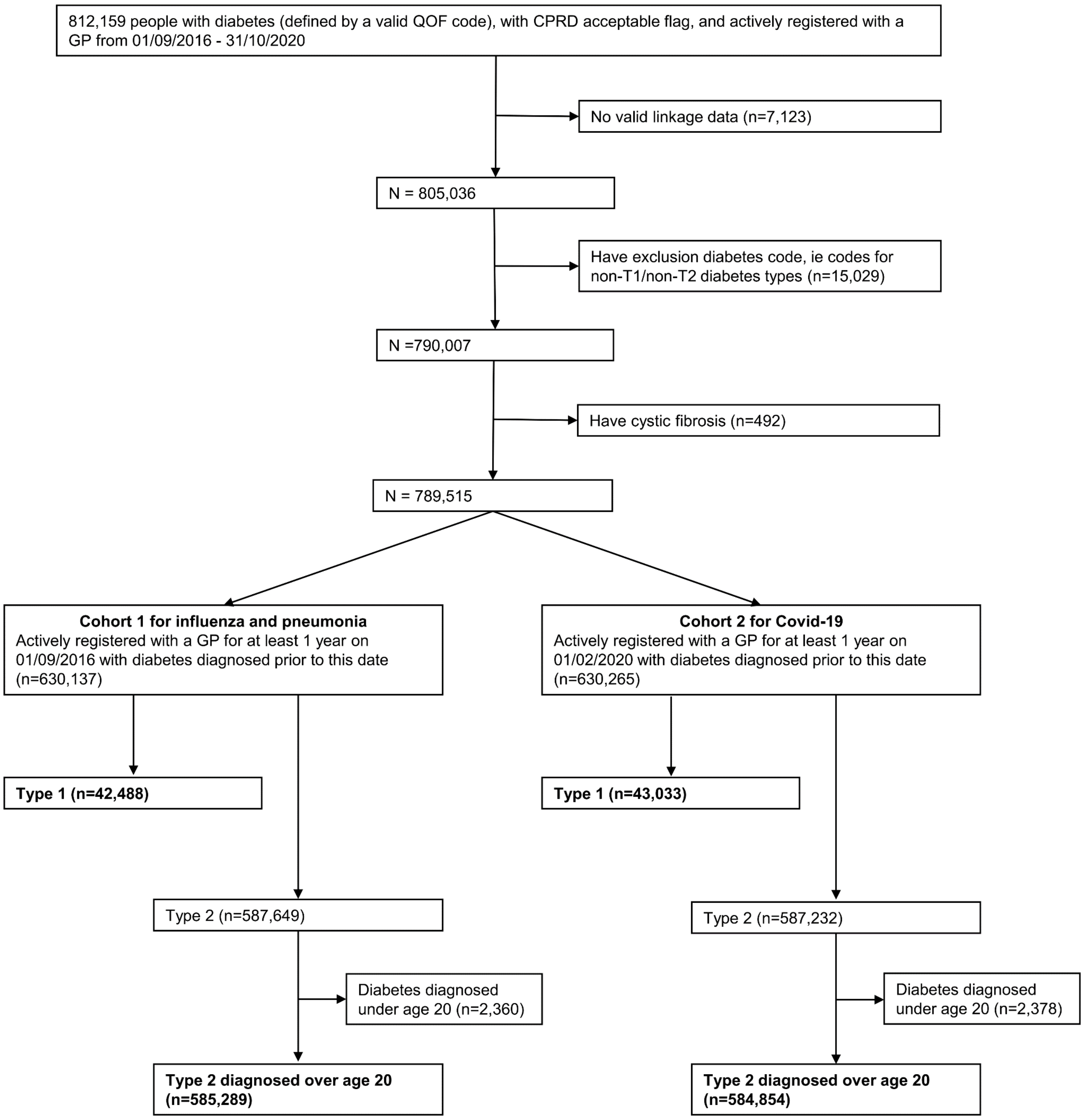

Supplemental table 1. ICD-10 codes used to define Covid-19, influenza, and pneumonia hospitalisation and mortality outcomes.

**Infection**	**ICD-10 codes**
Covid-19	U07.1, U07.2
Influenza	J09-J11
Pneumonia	J12-J18

Supplemental table 2. Definitions of exposure variables.

Variable	Definition	Data source	Categories	Missing data method
Sex	Sex recorded in CPRD	Primary care	Male, or female	No missing data
Age	Age at baseline date, calculated from date of birth	Primary care	<40, 40-49, 50-59, 60-69, 70-79, 80-89 or 90+ years for type 2 diabetes cohorts, and <18, 18-39, 40-49, 50-59, 60-69, 70-79 or 80+ years for type 1 diabetes cohorts	No missing data
Ethnicity	Major UK ethnic group based on a previous study [1]	Primary care and HES	White, south Asian, black, mixed, other, or unknown	Missing indicator method
Deprivation	Index of Multiple Deprivation (official national measure) [2]	Linked Index of Multiple Deprivation dataset	1, 2, 3, 4, 5, or missing (Quintile of national distribution)	Missing indicator method
Smoking status	Smoking status at baseline date	Primary care	Non-smoker, active smoker, ex-smoker, or unknown	Missing indicator method
Region	UK strategic health region based on GP practice location	Primary care	East Midlands, East of England, London, North East, North West, South Central, South East Coast, South West, West Midlands, Yorkshire and the Humber, or missing	Missing indicator method
HbA1c	Latest recorded HbA1c measurement in the last two years prior to baseline date	Primary care	<48 mmol/ mol (<6.5%), 48-53 mmol/mol (6.5-7%), 53-64 mmol/mol (7-8%), 64-75 mmol/mol (8-9%), 75-86 mmol/mol (9-10%), >86 mmol/mol (>10%), or missing	Missing indicator method
BMI	Latest recorded BMI measurement in the last two years prior to baseline date, grouped using the World Health Organisation classification [3]	Primary care	<18.5 kg/m², 18.5-24.9 kg/m², 25-29.9 kg/m², 30-34.5 kg/m², 35-39.9 kg/m², 40+ kg/m², or missing	Missing indicator method
Diabetes duration	Years since diabetes diagnosis, with diagnosis date defined using an algorithm adapted from a previous study [4] taking the earliest date of first diabetes clinical code, first HbA1c measurement of 48 mmol/mol (6.5%) or above, first oral hypoglycaemic agent (OHA) prescription or first insulin prescription	Primary care	<1 year, 1-2 years, 3-5 years, 6-9 years, 10-14 years, 15-19 years or 20+ years for the type 2 diabetes cohorts, and <5 years, 5-9 years, 10-14 years, 15-19 years, 20-24 years, 25-29 years or 30+ years for type 1 diabetes cohorts	No missing data
Microvascular complications	Total number of coded microvascular complications (diabetic nephropathy, neuropathy, and retinopathy) recorded prior to baseline date	Primary care and HES	0, 1, 2, or 3 complications	Non by definition
Diabetes treatment	Diabetes treatment recorded within last 6 months prior to baseline date	Primary care	Insulin prescription, oral hypoglycaemic agent (OHA) prescriptions only, or no treatment	Non by definition
Comorbidities	Record of comorbidity with diagnosis date prior to baseline date. Comorbidities studied covered cardiovascular (hypertension, atrial fibrillation, angina, previous myocardial infarction, previous cardiac revascularisation, other ischaemic heart disease, heart failure, peripheral vascular disease), respiratory (asthma, chronic obstructive pulmonary disease), neurological (previous transient ischaemic attack, previous stroke, dementia, or other neurological condition), oncological (haematological cancer, solid cancer), and other conditions (chronic liver disease, solid organ transplant)	Primary care and HES	Yes, or no	Non by definition
Chronic kidney disease stage	Stage defined from estimated glomerular filtration rate (eGFR), stage 5 also ascertained by coded record prior to baseline [5]	Primary care and HES	Stage 1, stage 2, stage 3a, stage 3b, stage 4, stage 5, or missing	Missing indicator method
Albumin creatinine ratio	Defined from latest albumin creatinine ratio measurement prior to baseline	Primary care	A1, A2, A3, or missing	Missing indicator method
Recent hospitalisation for respiratory infection	A hospitalisation recorded with a code for respiratory infection in the 2 years prior to baseline	HES	Yes, or no	Non by definition
Recent hospitalisation for other causes	A hospitalisation recorded for any other cause (excluding respiratory infection) in the 2 years prior to baseline	HES	Yes, or no	Non by definition
Additional respiratory infections	Respiratory conditions previously included in the 2020 QCOVID risk prediction algorithm [6] (pulmonary fibrosis, pulmonary hypertension, bronchiectasis) with diagnosis occurring before the baseline date	Primary care and HES	Yes, or no	Non by definition
Other medications	Medications previously included in 2020 QCOVID risk prediction algorithm [6] (immunosuppressants, leukotrienes, long acting β agonists, oral steroids) with a prescription issued within 6 months prior to baseline	Primary care	Yes, or no	Non by definition

Supplemental table 3. Frequency of ICD-10 codes recorded in hospitalisation outcomes in type 2 diabetes for Covid-19 (2020), influenza (2016-2019), and pneumonia (2016-2019).

Covid-19		
**ICD10 code**	**Description**	**Frequency**
U07.1	COVID-19, confirmed by lab testing	4972
U07.2	COVID-19, not confirmed by lab testing (includes clinically-epidemiologically diagnosed, probable and suspected)	1073
**Influenza**		
**ICD10 code**	**Description**	**Frequency**
J10.1	Influenza with other respiratory manifestations, seasonal influenza virus identified	1645
J10.0	Influenza with pneumonia, seasonal influenza virus identified	1158
J11.1	Influenza with other respiratory manifestations, virus not identified	250
J10.8	Influenza with other manifestations, seasonal influenza virus identified	134
J11.0	Influenza with pneumonia, virus not identified	101
J09	Influenza due to identified zoonotic or pandemic influenza virus	11
J11.8	Influenza with other manifestations, virus not identified	10
**Pneumonia**		
**ICD10 code**	**Description**	**Frequency**
J18.1	Lobar pneumonia, unspecified	20367
J18.9	Pneumonia, unspecified	16382
J18.0	Bronchopneumonia, unspecified	885
J13	Pneumonia due to Streptococcus pneumoniae	407
J14	Pneumonia due to Haemophilus influenzae	138
J15.0	Pneumonia due to Klebsiella pneumoniae	137
J15.1	Pneumonia due to Pseudomonas	116
J15.2	Pneumonia due to staphylococcus	88
J15.4	Pneumonia due to other streptococci	73
J12.1	Respiratory syncytial virus pneumonia	71
J15.6	Pneumonia due to other Gram-negative bacteria	53
J15.9	Bacterial pneumonia, unspecified	53
J12.3	Human metapneumovirus pneumonia	46
J15.5	Pneumonia due to Escherichia coli	43
J17.2	Pneumonia in mycoses	41
J12.2	Parainfluenza virus pneumonia	35
J12.9	Viral pneumonia, unspecified	34
J17.3	Pneumonia in parasitic diseases	34
J18.8	Other pneumonia, organism unspecified	29
J15.8	Other bacterial pneumonia	24
J18.2	Hypostatic pneumonia, unspecified	22
J12.8	Other viral pneumonia	21
J15.3	Pneumonia due to streptococcus, group B	14
J15.7	Pneumonia due to Mycoplasma pneumoniae	14
J17.1	Pneumonia in viral diseases classified elsewhere	9
J12.0	Adenoviral pneumonia	6
J17.0	Pneumonia in bacterial diseases classified elsewhere	2
J16.0	Chlamydial pneumonia	1
J16.8	Pneumonia due to other specified infectious organisms	1

Supplemental table 4. Full baseline characteristics and Covid-19 (2020), influenza (2016-2019), and pneumonia (2016-2019) hospitalisations in type 2 diabetes.

	2020 cohort	Covid-19 hospitalisations (in 2020 cohort)	2016 cohort	Influenza hospitalisations (in 2016 cohort)	Pneumonia hospitalisations (in 2016 cohort)
**Number of individuals**	584854	5965	585289	3226	38088
**Mean (SD) follow-up, days**		265.0 (38.3)		915.2 (224.8)	897.4 (246.3)
**Sex**					
Female	253360 (43.3)	2317 (38.8)	256656 (43.9)	1581 (49.0)	17140 (45.0)
Male	331494 (56.7)	3648 (61.2)	328633 (56.1)	1645 (51.0)	20948 (55.0)
**Age group, years**					
<40	12898 (2.2)	70 (1.2)	13468 (2.3)	46 (1.4)	164 (0.4)
40-49	46075 (7.9)	258 (4.3)	49649 (8.5)	128 (4.0)	717 (1.9)
50-59	114537 (19.6)	729 (12.2)	113285 (19.4)	329 (10.2)	2495 (6.6)
60-69	151493 (25.9)	1198 (20.1)	153782 (26.3)	679 (21.0)	6068 (15.9)
70-79	155424 (26.6)	1660 (27.8)	152401 (26.0)	1078 (33.4)	12073 (31.7)
80-89	89871 (15.4)	1671 (28.0)	88492 (15.1)	811 (25.1)	13324 (35.0)
90+	14556 (2.5)	379 (6.4)	14212 (2.4)	155 (4.8)	3247 (8.5)
**Ethnicity**					
White	445160 (76.1)	4200 (70.4)	457714 (78.2)	2586 (80.2)	33206 (87.2)
South Asian	77253 (13.2)	891 (14.9)	71050 (12.1)	403 (12.5)	2935 (7.7)
Black	35144 (6.0)	645 (10.8)	33175 (5.7)	156 (4.8)	1324 (3.5)
Other	9886 (1.7)	144 (2.4)	8178 (1.4)	35 (1.1)	306 (0.8)
Mixed	6267 (1.1)	69 (1.2)	5654 (1.0)	37 (1.1)	246 (0.6)
Unknown	11144 (1.9)	16 (0.3)	9518 (1.6)	9 (0.3)	71 (0.2)
**Index of multiple deprivation quintile**					
1 (least deprived)	102950 (17.6)	761 (12.8)	103184 (17.6)	502 (15.6)	6296 (16.5)
2	106968 (18.3)	921 (15.4)	109006 (18.6)	548 (17.0)	6923 (18.2)
3	113454 (19.4)	1088 (18.2)	113661 (19.4)	601 (18.6)	7264 (19.1)
4	127048 (21.7)	1446 (24.2)	125492 (21.4)	698 (21.6)	8199 (21.5)
5 (most deprived)	134146 (22.9)	1747 (29.3)	133563 (22.8)	873 (27.1)	9380 (24.6)
Missing	288 (0.0)	2 (0.0)	383 (0.1)	4 (0.1)	26 (0.1)
**Duration of diagnosed diabetes, years**					
<1	14311 (2.4)	67 (1.1)	36503 (6.2)	146 (4.5)	1401 (3.7)
1-2	60219 (10.3)	397 (6.7)	77510 (13.2)	306 (9.5)	3324 (8.7)
3-5	105094 (18.0)	796 (13.3)	111199 (19.0)	491 (15.2)	5218 (13.7)
6-9	128817 (22.0)	1116 (18.7)	125083 (21.4)	621 (19.2)	7088 (18.6)
10-14	124471 (21.3)	1250 (21.0)	126421 (21.6)	771 (23.9)	9620 (25.3)
15-19	89704 (15.3)	1225 (20.5)	62373 (10.7)	465 (14.4)	5764 (15.1)
20+	62238 (10.6)	1114 (18.7)	46200 (7.9)	426 (13.2)	5673 (14.9)
**HbA1c, mmol/mol**					
<48	167739 (28.7)	1679 (28.1)	175659 (30.0)	934 (29.0)	12271 (32.2)
48-53	116015 (19.8)	1054 (17.7)	116131 (19.8)	557 (17.3)	7015 (18.4)
53-64	147209 (25.2)	1352 (22.7)	142662 (24.4)	791 (24.5)	8912 (23.4)
64-75	66071 (11.3)	767 (12.9)	62291 (10.6)	412 (12.8)	4062 (10.7)
75-86	33970 (5.8)	408 (6.8)	32257 (5.5)	184 (5.7)	2191 (5.8)
86+	39727 (6.8)	580 (9.7)	37470 (6.4)	270 (8.4)	2646 (6.9)
Missing	14123 (2.4)	125 (2.1)	18819 (3.2)	78 (2.4)	991 (2.6)
**Number of microvascular complications**					
0	264011 (45.1)	1889 (31.7)	281811 (48.1)	1077 (33.4)	11930 (31.3)
1	216115 (37.0)	2140 (35.9)	204550 (34.9)	1256 (38.9)	14444 (37.9)
2	96948 (16.6)	1621 (27.2)	91671 (15.7)	789 (24.5)	10288 (27.0)
3	7780 (1.3)	315 (5.3)	7257 (1.2)	104 (3.2)	1426 (3.7)
**BMI, kg/m2**					
<18.5	3026 (0.5)	66 (1.1)	2801 (0.5)	16 (0.5)	497 (1.3)
18.5-24.9	86199 (14.7)	1009 (16.9)	79018 (13.5)	476 (14.8)	6985 (18.3)
25-29.9	184626 (31.6)	1747 (29.3)	178501 (30.5)	885 (27.4)	10950 (28.7)
30-34.9	151732 (25.9)	1350 (22.6)	151161 (25.8)	809 (25.1)	8249 (21.7)
35-39.9	74766 (12.8)	752 (12.6)	75339 (12.9)	405 (12.6)	3976 (10.4)
40+	48455 (8.3)	517 (8.7)	48685 (8.3)	302 (9.4)	2877 (7.6)
Missing	36050 (6.2)	524 (8.8)	49784 (8.5)	333 (10.3)	4554 (12.0)
**Smoking status**					
Active smoker	87220 (14.9)	556 (9.3)	90218 (15.4)	548 (17.0)	6259 (16.4)
Ex-smoker	336719 (57.6)	3847 (64.5)	316723 (54.1)	1852 (57.4)	22587 (59.3)
Non-smoker	138840 (23.7)	1288 (21.6)	149524 (25.5)	651 (20.2)	7305 (19.2)
Unknown	22075 (3.8)	274 (4.6)	28824 (4.9)	175 (5.4)	1937 (5.1)
**Comorbidities**					
**Cardiovascular**					
Hypertension	425278 (72.7)	5055 (84.7)	419645 (71.7)	2692 (83.4)	33013 (86.7)
Atrial fibrillation	70633 (12.1)	1413 (23.7)	63872 (10.9)	727 (22.5)	10486 (27.5)
Angina	87970 (15.0)	1526 (25.6)	91026 (15.6)	883 (27.4)	10973 (28.8)
Previous myocardial infarction	63738 (10.9)	1249 (20.9)	60576 (10.3)	645 (20.0)	8239 (21.6)
Previous cardiac revascularisation	57332 (9.8)	949 (15.9)	54961 (9.4)	497 (15.4)	5926 (15.6)
Other ischaemic heart disease	121776 (20.8)	2102 (35.2)	121753 (20.8)	1170 (36.3)	14645 (38.5)

	2020 cohort	Covid-19 hospitalisations (in 2020 cohort)	2016 cohort	Influenza hospitalisations (in 2016 cohort)	Pneumonia hospitalisations (in 2016 cohort)
Heart failure	61014 (10.4)	1573 (26.4)	53579 (9.2)	735 (22.8)	10402 (27.3)
Peripheral arterial disease	50045 (8.6)	1157 (19.4)	48489 (8.3)	481 (14.9)	7700 (20.2)
**Recent hospitalisation**					
Respiratory infection	25535 (4.4)	1049 (17.6)	21353 (3.6)	436 (13.5)	6127 (16.1)
Anything else	96421 (16.5)	2426 (40.7)	95232 (16.3)	1195 (37.0)	15007 (39.4)
**Respiratory**					
Asthma	125582 (21.5)	1567 (26.3)	118207 (20.2)	1063 (33.0)	11229 (29.5)
Chronic obstructive pulmonary disease	65615 (11.2)	1273 (21.3)	60108 (10.3)	806 (25.0)	10948 (28.7)
**Neurological**					
Previous transient ischaemic attack	31024 (5.3)	598 (10.0)	29997 (5.1)	294 (9.1)	4200 (11.0)
Previous stroke	49873 (8.5)	987 (16.5)	47721 (8.2)	504 (15.6)	6839 (18.0)
Dementia	20629 (3.5)	718 (12.0)	19548 (3.3)	226 (7.0)	3710 (9.7)
Other neurological condition	35000 (6.0)	741 (12.4)	33569 (5.7)	352 (10.9)	4967 (13.0)
**Oncological**					
Haematological cancer	11751 (2.0)	212 (3.6)	10352 (1.8)	155 (4.8)	1499 (3.9)
Solid cancer	79179 (13.5)	1130 (18.9)	72001 (12.3)	544 (16.9)	7887 (20.7)
**Other**					
Solid organ transplant	3682 (0.6)	99 (1.7)	3013 (0.5)	49 (1.5)	509 (1.3)
Chronic liver disease	69136 (11.8)	860 (14.4)	48011 (8.2)	333 (10.3)	3387 (8.9)
**Chronic Kidney Disease (CKD) stage**					
Stage 1	165845 (28.4)	933 (15.6)	147798 (25.3)	448 (13.9)	3873 (10.2)
Stage 2	286696 (49.0)	2438 (40.9)	290821 (49.7)	1399 (43.4)	15243 (40.0)
Stage 3a	72539 (12.4)	1079 (18.1)	80222 (13.7)	642 (19.9)	8056 (21.2)
Stage 3b	33246 (5.7)	699 (11.7)	37256 (6.4)	372 (11.5)	5844 (15.3)
Stage 4	9006 (1.5)	301 (5.0)	10490 (1.8)	143 (4.4)	2323 (6.1)
Stage 5	12871 (2.2)	496 (8.3)	13365 (2.3)	205 (6.4)	2543 (6.7)
Missing	4651 (0.8)	19 (0.3)	5337 (0.9)	17 (0.5)	206 (0.5)
**Albumin creatinine ratio (ACR) category**					
A1	402847 (68.9)	3284 (55.1)	388839 (66.4)	1812 (56.2)	19926 (52.3)
A2	121002 (20.7)	1719 (28.8)	106879 (18.3)	845 (26.2)	11550 (30.3)
A3	21899 (3.7)	614 (10.3)	18817 (3.2)	252 (7.8)	3219 (8.5)
Missing	39106 (6.7)	348 (5.8)	70754 (12.1)	317 (9.8)	3393 (8.9)
**Diabetes treatment (last 6 months)**					
Insulin prescription	79286 (13.6)	1490 (25.0)	78755 (13.5)	742 (23.0)	8481 (22.3)
OHA prescription only	362651 (62.0)	3167 (53.1)	349629 (59.7)	1739 (53.9)	19713 (51.8)
No treatment	142917 (24.4)	1308 (21.9)	156905 (26.8)	745 (23.1)	9894 (26.0)

Supplemental figure 2. Association of potential risk factors with hospitalisation for Covid-19 (2020), influenza (2016-2019), and pneumonia (2016-2019) in type 2 diabetes.

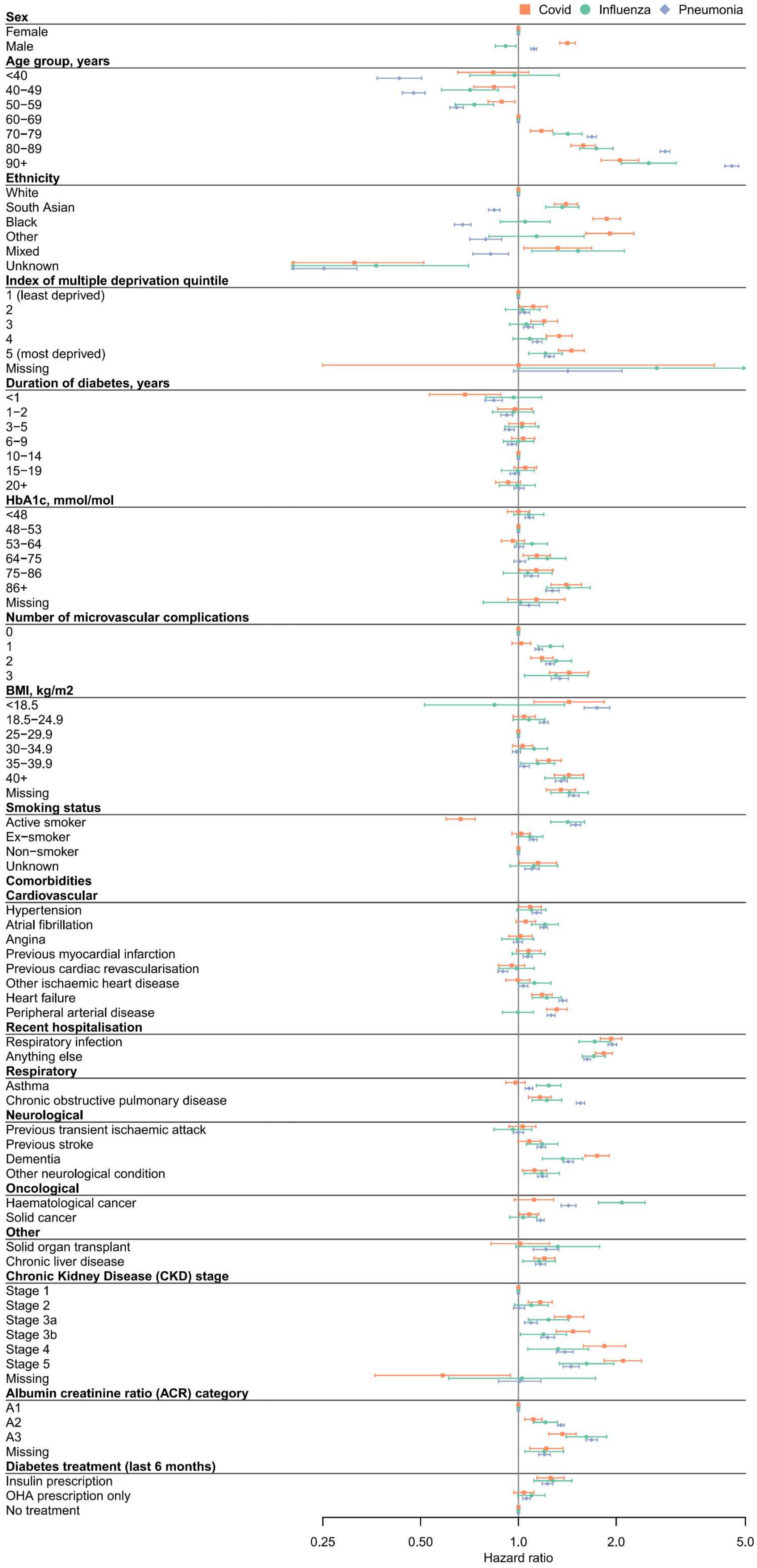

Supplemental table 5. Hazard ratio (HR) values and 95% confidence intervals (95% CI) for the association of potential risk factors with hospitalisation for Covid-19 (2020), influenza (2016-2019), and pneumonia (2016-2019) in type 2 diabetes.

	Covid-19		Influenza		Pneumonia	
	HR (95% CI)	p value	HR (95% CI)	p value	HR (95% CI)	p value
**Sex**						
Female	1 (ref)		1 (ref)		1 (ref)	
Male	1.42 (1.34-1.5)	<0.001	0.91 (0.85-0.98)	0.016	1.12 (1.09-1.14)	<0.001
**Age group, years**						
<40	0.84 (0.65-1.08)	0.165	0.97 (0.71-1.33)	0.862	0.43 (0.37-0.5)	<0.001
40-49	0.84 (0.73-0.97)	0.019	0.71 (0.58-0.87)	<0.001	0.48 (0.44-0.52)	<0.001
50-59	0.89 (0.81-0.98)	0.014	0.73 (0.64-0.84)	<0.001	0.65 (0.62-0.68)	<0.001
60-69	1 (ref)		1 (ref)		1 (ref)	
70-79	1.18 (1.09-1.27)	<0.001	1.42 (1.28-1.57)	<0.001	1.68 (1.63-1.74)	<0.001
80-89	1.58 (1.45-1.73)	<0.001	1.74 (1.54-1.96)	<0.001	2.83 (2.73-2.93)	<0.001
90+	2.05 (1.8-2.35)	<0.001	2.52 (2.07-3.06)	<0.001	4.54 (4.32-4.77)	<0.001
**Ethnicity**						
White	1 (ref)		1 (ref)		1 (ref)	
South Asian	1.4 (1.29-1.52)	<0.001	1.36 (1.21-1.54)	<0.001	0.84 (0.81-0.88)	<0.001
Black	1.87 (1.7-2.06)	<0.001	1.05 (0.88-1.25)	0.593	0.67 (0.64-0.71)	<0.001
Other	1.91 (1.61-2.27)	<0.001	1.14 (0.81-1.59)	0.456	0.79 (0.71-0.89)	<0.001
Mixed	1.32 (1.04-1.68)	0.023	1.53 (1.1-2.12)	0.011	0.82 (0.72-0.93)	0.002
Unknown	0.31 (0.19-0.51)	<0.001	0.36 (0.19-0.7)	0.003	0.25 (0.2-0.32)	<0.001
**Index of multiple deprivation quintile**						
1 (least deprived)	1 (ref)		1 (ref)		1 (ref)	
2	1.11 (1.01-1.23)	0.031	1.03 (0.91-1.16)	0.637	1.05 (1.01-1.08)	0.009
3	1.2 (1.09-1.32)	<0.001	1.06 (0.94-1.19)	0.357	1.07 (1.04-1.11)	<0.001
4	1.34 (1.22-1.46)	<0.001	1.08 (0.96-1.22)	0.185	1.14 (1.1-1.18)	<0.001
5 (most deprived)	1.46 (1.33-1.6)	<0.001	1.21 (1.08-1.36)	0.002	1.25 (1.2-1.29)	<0.001
Missing	1 (0.25-4.01)	0.999	2.67 (1-7.14)	0.051	1.42 (0.97-2.09)	0.075
**Duration of diagnosed diabetes, years**						
<1	0.68 (0.53-0.88)	0.003	0.97 (0.79-1.18)	0.739	0.84 (0.79-0.89)	<0.001
1-2	0.98 (0.86-1.1)	0.693	0.96 (0.83-1.11)	0.619	0.92 (0.88-0.96)	<0.001
3-5	1.03 (0.93-1.13)	0.585	1.03 (0.91-1.15)	0.683	0.94 (0.9-0.97)	<0.001
6-9	1.04 (0.95-1.12)	0.408	1 (0.9-1.11)	0.992	0.96 (0.93-0.99)	0.004
10-14	1 (ref)		1 (ref)		1 (ref)	
15-19	1.05 (0.97-1.14)	0.232	1 (0.89-1.12)	0.935	0.97 (0.94-1.01)	0.135
20+	0.93 (0.85-1.02)	0.108	0.99 (0.87-1.13)	0.906	1.01 (0.97-1.04)	0.774
**HbA1c, mmol/mol**						
<48	1 (0.93-1.08)	0.975	1.08 (0.97-1.2)	0.172	1.08 (1.05-1.11)	<0.001
48-53	1 (ref)		1 (ref)		1 (ref)	
53-64	0.96 (0.89-1.04)	0.349	1.1 (0.99-1.23)	0.09	1 (0.97-1.04)	0.776
64-75	1.14 (1.03-1.25)	0.009	1.23 (1.07-1.4)	0.003	1.01 (0.97-1.05)	0.642
75-86	1.13 (1.01-1.28)	0.037	1.07 (0.9-1.27)	0.457	1.1 (1.04-1.15)	<0.001
86+	1.4 (1.26-1.56)	<0.001	1.43 (1.22-1.66)	<0.001	1.27 (1.21-1.33)	<0.001
Missing	1.14 (0.93-1.39)	0.22	1.01 (0.78-1.32)	0.913	1.08 (1-1.16)	0.048
**Number of microvascular complications**						
0	1 (ref)		1 (ref)		1 (ref)	
1	1.02 (0.96-1.09)	0.527	1.26 (1.15-1.37)	<0.001	1.16 (1.13-1.19)	<0.001
2	1.18 (1.09-1.28)	<0.001	1.31 (1.17-1.46)	<0.001	1.25 (1.21-1.29)	<0.001
3	1.43 (1.25-1.65)	<0.001	1.31 (1.04-1.64)	0.019	1.34 (1.26-1.43)	<0.001
**BMI, kg/m2**						
<18.5	1.43 (1.12-1.84)	0.005	0.84 (0.51-1.39)	0.504	1.75 (1.59-1.91)	<0.001
18.5-24.9	1.04 (0.96-1.13)	0.312	1.08 (0.96-1.21)	0.19	1.2 (1.16-1.23)	<0.001
25-29.9	1 (ref)		1 (ref)		1 (ref)	
30-34.9	1.03 (0.96-1.11)	0.397	1.12 (1.01-1.23)	0.025	0.99 (0.96-1.02)	0.383
35-39.9	1.24 (1.14-1.35)	<0.001	1.15 (1.02-1.29)	0.026	1.04 (1-1.08)	0.035
40+	1.43 (1.29-1.59)	<0.001	1.39 (1.21-1.59)	<0.001	1.35 (1.3-1.41)	<0.001
Missing	1.35 (1.22-1.5)	<0.001	1.44 (1.26-1.64)	<0.001	1.48 (1.43-1.54)	<0.001
**Smoking status**						
Active smoker	0.66 (0.6-0.74)	<0.001	1.42 (1.26-1.6)	<0.001	1.5 (1.45-1.55)	<0.001
Ex-smoker	1.02 (0.96-1.09)	0.552	1.09 (0.99-1.19)	0.083	1.11 (1.08-1.14)	<0.001
Non-smoker	1 (ref)		1 (ref)		1 (ref)	
Unknown	1.15 (1.01-1.31)	0.039	1.12 (0.94-1.32)	0.204	1.1 (1.05-1.16)	<0.001
**Comorbidities**						
**Cardiovascular**						
Hypertension	1.09 (1.01-1.18)	0.037	1.1 (0.99-1.22)	0.077	1.14 (1.1-1.17)	<0.001
Atrial fibrillation	1.05 (0.98-1.13)	0.139	1.21 (1.1-1.33)	<0.001	1.2 (1.17-1.23)	<0.001
Angina	1.02 (0.93-1.1)	0.714	0.99 (0.89-1.11)	0.927	1 (0.97-1.03)	0.836
Previous myocardial infarction	1.08 (0.99-1.17)	0.099	1.08 (0.96-1.21)	0.221	1.07 (1.03-1.1)	<0.001
Previous cardiac revascularisation	0.95 (0.87-1.05)	0.315	0.99 (0.87-1.12)	0.841	0.9 (0.86-0.93)	<0.001
Other ischaemic heart disease	1 (0.92-1.09)	0.938	1.12 (1-1.26)	0.057	1.03 (1-1.07)	0.041
Heart failure	1.18 (1.1-1.27)	<0.001	1.22 (1.1-1.35)	<0.001	1.37 (1.33-1.41)	<0.001
Peripheral arterial disease	1.31 (1.22-1.41)	<0.001	1 (0.89-1.11)	0.957	1.26 (1.23-1.3)	<0.001
**Recent hospitalisation**						
Respiratory infection	1.93 (1.79-2.08)	<0.001	1.72 (1.54-1.92)	<0.001	1.95 (1.89-2.01)	<0.001
Anything else	1.83 (1.73-1.95)	<0.001	1.71 (1.57-1.85)	<0.001	1.63 (1.59-1.67)	<0.001

	Covid-19		Influenza		Pneumonia	
	HR (95% CI)	p value	HR (95% CI)	p value	HR (95% CI)	p value
**Respiratory**						
Asthma	0.98 (0.92-1.05)	0.547	1.24 (1.14-1.35)	<0.001	1.08 (1.05-1.11)	<0.001
Chronic obstructive pulmonary disease	1.16 (1.07-1.26)	<0.001	1.22 (1.1-1.36)	<0.001	1.55 (1.51-1.6)	<0.001
**Neurological**						
Previous transient ischaemic attack	1.03 (0.94-1.13)	0.543	0.96 (0.84-1.1)	0.568	1 (0.96-1.04)	0.991
Previous stroke	1.08 (1-1.17)	0.06	1.18 (1.06-1.32)	0.003	1.18 (1.14-1.21)	<0.001
Dementia	1.75 (1.61-1.9)	<0.001	1.37 (1.19-1.58)	<0.001	1.42 (1.37-1.48)	<0.001
Other neurological condition	1.12 (1.03-1.22)	0.009	1.18 (1.04-1.34)	0.009	1.19 (1.15-1.23)	<0.001
**Oncological**						
Haematological cancer	1.12 (0.97-1.29)	0.121	2.08 (1.76-2.46)	<0.001	1.43 (1.35-1.5)	<0.001
Solid cancer	1.08 (1.01-1.15)	0.027	1.03 (0.94-1.14)	0.49	1.17 (1.14-1.2)	<0.001
**Other**						
Solid organ transplant	1.01 (0.82-1.25)	0.897	1.32 (0.98-1.78)	0.066	1.22 (1.11-1.33)	<0.001
Chronic liver disease	1.2 (1.12-1.3)	<0.001	1.16 (1.03-1.3)	0.013	1.17 (1.13-1.21)	<0.001
**Chronic Kidney Disease (CKD) stage**						
Stage 1	1 (ref)		1 (ref)		1 (ref)	
Stage 2	1.17 (1.07-1.27)	<0.001	1.1 (0.97-1.24)	0.132	1 (0.97-1.04)	0.827
Stage 3a	1.43 (1.29-1.59)	<0.001	1.24 (1.07-1.43)	0.004	1.09 (1.04-1.14)	<0.001
Stage 3b	1.47 (1.31-1.66)	<0.001	1.19 (1.01-1.41)	0.033	1.23 (1.17-1.29)	<0.001
Stage 4	1.84 (1.58-2.14)	<0.001	1.33 (1.07-1.64)	0.01	1.39 (1.31-1.48)	<0.001
Stage 5	2.1 (1.84-2.39)	<0.001	1.62 (1.34-1.97)	<0.001	1.45 (1.37-1.54)	<0.001
Missing	0.58 (0.36-0.95)	0.029	1.03 (0.61-1.73)	0.921	1.01 (0.87-1.17)	0.903
**Albumin creatinine ratio (ACR) category**						
A1	1 (ref)		1 (ref)		1 (ref)	
A2	1.11 (1.05-1.18)	<0.001	1.21 (1.11-1.32)	<0.001	1.35 (1.32-1.38)	<0.001
A3	1.37 (1.24-1.51)	<0.001	1.62 (1.4-1.87)	<0.001	1.68 (1.61-1.75)	<0.001
Missing	1.22 (1.08-1.37)	<0.001	1.2 (1.05-1.38)	0.008	1.2 (1.15-1.25)	<0.001
**Diabetes treatment (last 6 months)**						
Insulin prescription	1.26 (1.14-1.38)	<0.001	1.28 (1.12-1.46)	<0.001	1.23 (1.18-1.28)	<0.001
OHA prescription only	1.04 (0.97-1.12)	0.286	1.1 (1-1.21)	0.062	1.06 (1.03-1.09)	<0.001
No treatment	1 (ref)		1 (ref)		1 (ref)	

Hopkins R, *et al. BMJ Open* 2024; 14:e078135. doi: 10.1136/bmjopen-2023-078135

Supplemental figure 3A. Association of continuous HbA1c with hospitalisation for Covid-19 (2020), influenza (2016-2019), and pneumonia (2016-2019) in type 2 diabetes by ethnicity subgroup. Density plots show the distribution of HbA1c in each group.

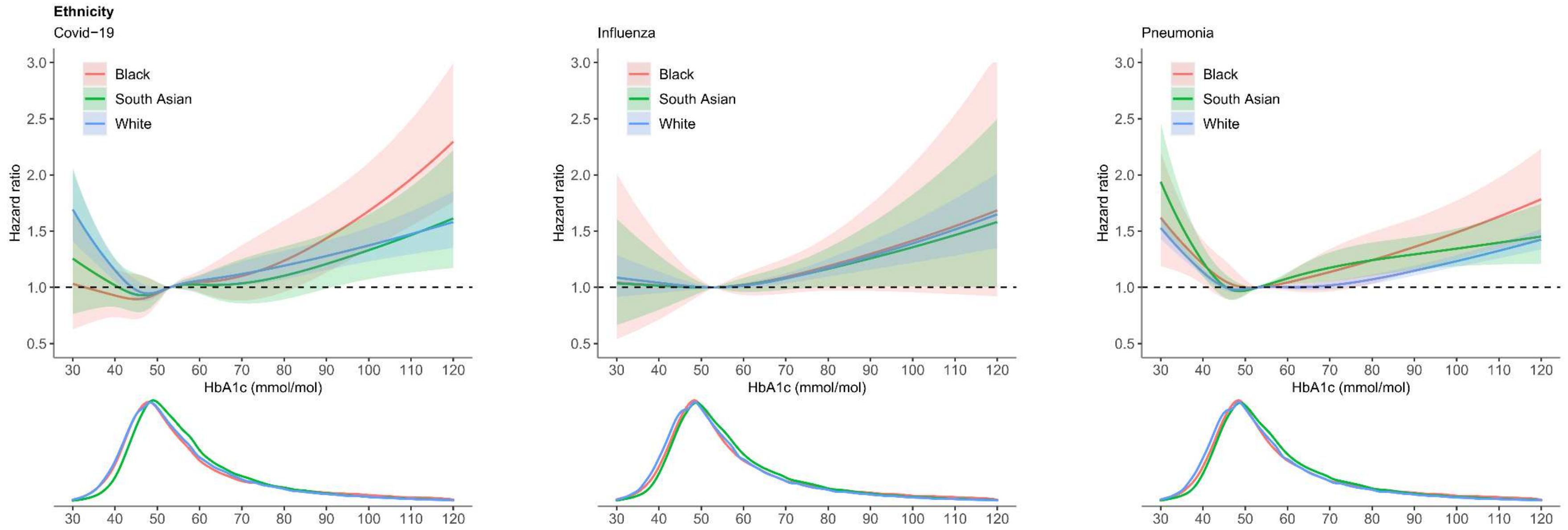

Supplemental figure 3B. Association of continuous BMI with hospitalisation for Covid-19 (2020), influenza (2016-2019), and pneumonia (2016-2019) in type 2 diabetes by ethnicity subgroup. Density plots show the distribution of BMI in each group.

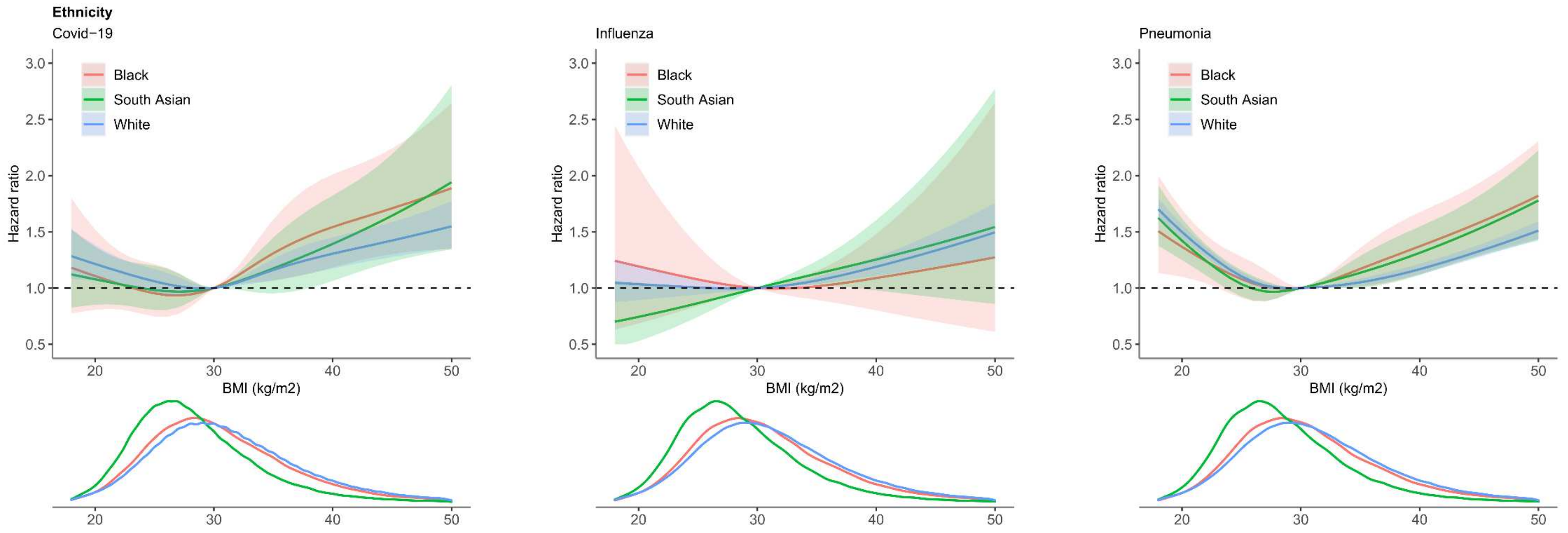

Supplemental table 6. Baseline characteristics and Covid-19 (2020) and pneumonia (2016-2019) deaths in type 2 diabetes.

	2020 cohort	Covid-19 deaths (in 2020 cohort)	2016 cohort	Pneumonia deaths (in 2016 cohort)
**Number of individuals**	584854	2635	585289	12652
**Mean (SD) follow-up, days**		266.1 (35.5)		917.0 (223.2)
**Sex**				
Female	253360 (43.3)	1023 (38.8)	256656 (43.9)	5499 (43.5)
Male	331494 (56.7)	1612 (61.2)	328633 (56.1)	7153 (56.5)
**Age group, years**				
<40	12898 (2.2)	7 (0.3)	13468 (2.3)	9 (0.1)
40-49	46075 (7.9)	38 (1.4)	49649 (8.5)	91 (0.7)
50-59	114537 (19.6)	150 (5.7)	113285 (19.4)	404 (3.2)
60-69	151493 (25.9)	381 (14.5)	153782 (26.3)	1401 (11.1)
70-79	155424 (26.6)	729 (27.7)	152401 (26.0)	3667 (29.0)
80-89	89871 (15.4)	1047 (39.7)	88492 (15.1)	5370 (42.4)
90+	14556 (2.5)	283 (10.7)	14212 (2.4)	1710 (13.5)
**Ethnicity**				
White	445160 (76.1)	1944 (73.8)	457714 (78.2)	11398 (90.1)
South Asian	77253 (13.2)	341 (12.9)	71050 (12.1)	748 (5.9)
Black	35144 (6.0)	267 (10.1)	33175 (5.7)	326 (2.6)
Other	9886 (1.7)	52 (2.0)	8178 (1.4)	86 (0.7)
Mixed	6267 (1.1)	21 (0.8)	5654 (1.0)	66 (0.5)
Unknown	11144 (1.9)	10 (0.4)	9518 (1.6)	28 (0.2)
**Index of multiple deprivation quintile**				
1 (least deprived)	102950 (17.6)	371 (14.1)	103184 (17.6)	2177 (17.2)
2	106968 (18.3)	418 (15.9)	109006 (18.6)	2378 (18.8)
3	113454 (19.4)	488 (18.5)	113661 (19.4)	2481 (19.6)
4	127048 (21.7)	607 (23.0)	125492 (21.4)	2708 (21.4)
5 (most deprived)	134146 (22.9)	749 (28.4)	133563 (22.8)	2904 (23.0)
Missing	288 (0.0)	2 (0.1)	383 (0.1)	4 (0.0)
**Duration of diagnosed diabetes, years**				
<1	14311 (2.4)	13 (0.5)	36503 (6.2)	331 (2.6)
1-2	60219 (10.3)	120 (4.6)	77510 (13.2)	845 (6.7)
3-5	105094 (18.0)	315 (12.0)	111199 (19.0)	1553 (12.3)
6-9	128817 (22.0)	438 (16.6)	125083 (21.4)	2294 (18.1)
10-14	124471 (21.3)	562 (21.3)	126421 (21.6)	3349 (26.5)
15-19	89704 (15.3)	604 (22.9)	62373 (10.7)	2112 (16.7)
20+	62238 (10.6)	583 (22.1)	46200 (7.9)	2168 (17.1)
**HbA1c, mmol/mol**				
<48	167739 (28.7)	838 (31.8)	175659 (30.0)	4411 (34.9)
48-53	116015 (19.8)	453 (17.2)	116131 (19.8)	2370 (18.7)
53-64	147209 (25.2)	567 (21.5)	142662 (24.4)	2862 (22.6)
64-75	66071 (11.3)	349 (13.2)	62291 (10.6)	1269 (10.0)
75-86	33970 (5.8)	168 (6.4)	32257 (5.5)	642 (5.1)
86+	39727 (6.8)	189 (7.2)	37470 (6.4)	756 (6.0)
Missing	14123 (2.4)	71 (2.7)	18819 (3.2)	342 (2.7)
**Number of microvascular complications**				
0	264011 (45.1)	706 (26.8)	281811 (48.1)	3447 (27.2)
1	216115 (37.0)	957 (36.3)	204550 (34.9)	4804 (38.0)
2	96948 (16.6)	804 (30.5)	91671 (15.7)	3868 (30.6)
3	7780 (1.3)	168 (6.4)	7257 (1.2)	533 (4.2)
**BMI, kg/m2**				
<18.5	3026 (0.5)	47 (1.8)	2801 (0.5)	319 (2.5)
18.5-24.9	86199 (14.7)	587 (22.3)	79018 (13.5)	2900 (22.9)
25-29.9	184626 (31.6)	755 (28.7)	178501 (30.5)	3616 (28.6)
30-34.9	151732 (25.9)	526 (20.0)	151161 (25.8)	2236 (17.7)
35-39.9	74766 (12.8)	246 (9.3)	75339 (12.9)	968 (7.7)
40+	48455 (8.3)	166 (6.3)	48685 (8.3)	656 (5.2)
Missing	36050 (6.2)	308 (11.7)	49784 (8.5)	1957 (15.5)
**Smoking status**				
Active smoker	87220 (14.9)	234 (8.9)	90218 (15.4)	1937 (15.3)
Ex-smoker	336719 (57.6)	1742 (66.1)	316723 (54.1)	7553 (59.7)
Non-smoker	138840 (23.7)	551 (20.9)	149524 (25.5)	2478 (19.6)
Unknown	22075 (3.8)	108 (4.1)	28824 (4.9)	684 (5.4)
**Comorbidities**				
**Cardiovascular**				
Hypertension	425278 (72.7)	2334 (88.6)	419645 (71.7)	11173 (88.3)
Atrial fibrillation	70633 (12.1)	769 (29.2)	63872 (10.9)	4175 (33.0)
Angina	87970 (15.0)	749 (28.4)	91026 (15.6)	3842 (30.4)
Previous myocardial infarction	63738 (10.9)	633 (24.0)	60576 (10.3)	3064 (24.2)
Previous cardiac revascularisation	57332 (9.8)	462 (17.5)	54961 (9.4)	2035 (16.1)
Other ischaemic heart disease	121776 (20.8)	1022 (38.8)	121753 (20.8)	5196 (41.1)
Heart failure	61014 (10.4)	848 (32.2)	53579 (9.2)	4195 (33.2)
Peripheral arterial disease	50045 (8.6)	635 (24.1)	48489 (8.3)	3060 (24.2)

	2020 cohort	Covid-19 deaths (in 2020 cohort)	2016 cohort	Pneumonia deaths (in 2016 cohort)
**Recent hospitalisation**				
Respiratory infection	25535 (4.4)	563 (21.4)	21353 (3.6)	2387 (18.9)
Anything else	96421 (16.5)	1171 (44.4)	95232 (16.3)	5409 (42.8)
**Respiratory**				
Asthma	125582 (21.5)	683 (25.9)	118207 (20.2)	3395 (26.8)
Chronic obstructive pulmonary disease	65615 (11.2)	642 (24.4)	60108 (10.3)	3755 (29.7)
**Neurological**				
Previous transient ischaemic attack	31024 (5.3)	334 (12.7)	29997 (5.1)	1597 (12.6)
Previous stroke	49873 (8.5)	570 (21.6)	47721 (8.2)	2591 (20.5)
Dementia	20629 (3.5)	572 (21.7)	19548 (3.3)	1831 (14.5)
Other neurological condition	35000 (6.0)	447 (17.0)	33569 (5.7)	1974 (15.6)
**Oncological**				
Haematological cancer	11751 (2.0)	119 (4.5)	10352 (1.8)	550 (4.3)
Solid cancer	79179 (13.5)	555 (21.1)	72001 (12.3)	2965 (23.4)
**Other**				
Solid organ transplant	3682 (0.6)	41 (1.6)	3013 (0.5)	193 (1.5)
Chronic liver disease	69136 (11.8)	322 (12.2)	48011 (8.2)	1034 (8.2)
**Chronic Kidney Disease (CKD) stage**				
Stage 1	165845 (28.4)	239 (9.1)	147798 (25.3)	988 (7.8)
Stage 2	286696 (49.0)	968 (36.7)	290821 (49.7)	4481 (35.4)
Stage 3a	72539 (12.4)	553 (21.0)	80222 (13.7)	2787 (22.0)
Stage 3b	33246 (5.7)	415 (15.7)	37256 (6.4)	2279 (18.0)
Stage 4	9006 (1.5)	186 (7.1)	10490 (1.8)	999 (7.9)
Stage 5	12871 (2.2)	261 (9.9)	13365 (2.3)	1043 (8.2)
Missing	4651 (0.8)	13 (0.5)	5337 (0.9)	75 (0.6)
**Albumin creatinine ratio (ACR) category**				
A1	402847 (68.9)	1271 (48.2)	388839 (66.4)	6044 (47.8)
A2	121002 (20.7)	845 (32.1)	106879 (18.3)	4334 (34.3)
A3	21899 (3.7)	322 (12.2)	18817 (3.2)	1281 (10.1)
Missing	39106 (6.7)	197 (7.5)	70754 (12.1)	993 (7.8)
**Diabetes treatment (last 6 months)**				
Insulin prescription	79286 (13.6)	682 (25.9)	78755 (13.5)	2772 (21.9)
OHA prescription only	362651 (62.0)	1289 (48.9)	349629 (59.7)	6309 (49.9)
No treatment	142917 (24.4)	664 (25.2)	156905 (26.8)	3571 (28.2)

Supplemental figure 4. Association of potential risk factors with Covid-19 (2020), and pneumonia (2016-2019) mortality in type 2 diabetes.

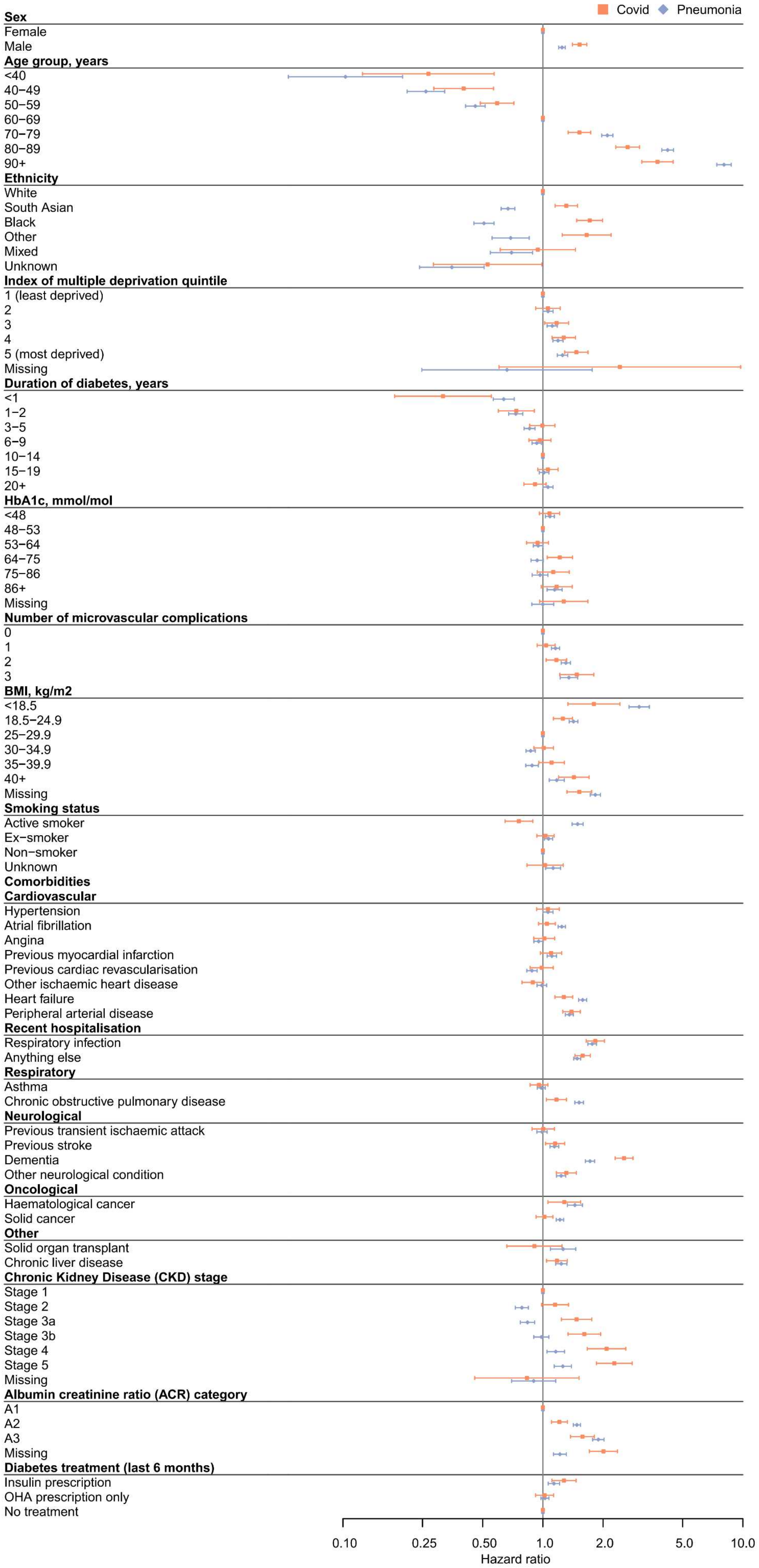

14

Supplemental table 7. Baseline characteristics and Covid-19 (2020), influenza (2016-2019), and pneumonia (2016-2019) hospitalisations in type 1 diabetes.

	2020 cohort	Covid-19 hospitalisations (in 2020 cohort)	2016 cohort	Influenza hospitalisations (in 2016 cohort)	Pneumonia hospitalisations (in 2016 cohort)
**Number of individuals**	43033	209	42488	208	1401
**Mean (SD) follow-up, days**		267.0 (33.2)		917.3 (223.0)	907.1 (236.2)
**Sex**					
Female	18172 (42.2)	76 (36.4)	17889 (42.1)	103 (49.5)	611 (43.6)
Male	24861 (57.8)	133 (63.6)	24599 (57.9)	105 (50.5)	790 (56.4)
**Age, years**					
<18	4458 (10.4)	1 (0.5)	4502 (10.6)	12 (5.8)	13 (0.9)
18-39	14454 (33.6)	34 (16.3)	13996 (32.9)	59 (28.4)	191 (13.6)
40-49	7480 (17.4)	31 (14.8)	7995 (18.8)	43 (20.7)	208 (14.8)
50-59	8056 (18.7)	42 (20.1)	7911 (18.6)	31 (14.9)	283 (20.2)
60-69	5079 (11.8)	43 (20.6)	4731 (11.1)	34 (16.3)	281 (20.1)
70-79	2689 (6.2)	37 (17.7)	2460 (5.8)	23 (11.1)	239 (17.1)
80+	817 (1.9)	21 (10.0)	893 (2.1)	6 (2.9)	186 (13.3)
**Ethnicity**					
White	38478 (89.4)	170 (81.3)	38337 (90.2)	187 (89.9)	1311 (93.6)
South Asian	1576 (3.7)	14 (6.7)	1494 (3.5)	14 (6.7)	37 (2.6)
Black	1385 (3.2)	16 (7.7)	1253 (2.9)	3 (1.4)	33 (2.4)
Other	464 (1.1)	3 (1.4)	373 (0.9)	0 (0.0)	5 (0.4)
Mixed	525 (1.2)	6 (2.9)	476 (1.1)	4 (1.9)	15 (1.1)
Unknown	605 (1.4)	0 (0.0)	555 (1.3)	0 (0.0)	0 (0.0)
**Index of multiple deprivation quintile**					
1 (least deprived)	9395 (21.8)	43 (20.6)	9251 (21.8)	28 (13.5)	254 (18.1)
2	8710 (20.2)	30 (14.4)	8652 (20.4)	37 (17.8)	237 (16.9)
3	8334 (19.4)	33 (15.8)	8347 (19.6)	44 (21.2)	276 (19.7)
4	8444 (19.6)	38 (18.2)	8342 (19.6)	45 (21.6)	292 (20.8)
5 (most deprived)	8128 (18.9)	65 (31.1)	7864 (18.5)	54 (26.0)	342 (24.4)
Missing	22 (0.1)	0 (0.0)	32 (0.1)	0 (0.0)	0 (0.0)
**Duration of diagnosed diabetes, years**					
<5	5655 (13.1)	8 (3.8)	6556 (15.4)	17 (8.2)	81 (5.8)
5-9	6194 (14.4)	13 (6.2)	6077 (14.3)	21 (10.1)	101 (7.2)
10-14	5609 (13.0)	23 (11.0)	5899 (13.9)	28 (13.5)	144 (10.3)
15-19	5466 (12.7)	27 (12.9)	5355 (12.6)	28 (13.5)	174 (12.4)
20-24	4621 (10.7)	25 (12.0)	4612 (10.9)	22 (10.6)	176 (12.6)
25-29	4101 (9.5)	27 (12.9)	3747 (8.8)	27 (13.0)	149 (10.6)
30+	11387 (26.5)	86 (41.1)	10242 (24.1)	65 (31.2)	576 (41.1)
**HbA1c, mmol/mol**					
<48	3104 (7.2)	12 (5.7)	2796 (6.6)	13 (6.2)	109 (7.8)
48-53	3232 (7.5)	13 (6.2)	2967 (7.0)	13 (6.2)	111 (7.9)
53-64	10995 (25.6)	50 (23.9)	10340 (24.3)	37 (17.8)	263 (18.8)
64-75	9951 (23.1)	36 (17.2)	10098 (23.8)	46 (22.1)	283 (20.2)
75-86	5891 (13.7)	30 (14.4)	5979 (14.1)	32 (15.4)	228 (16.3)
86+	6097 (14.2)	57 (27.3)	6351 (14.9)	45 (21.6)	327 (23.3)
Missing	3763 (8.7)	11 (5.3)	3957 (9.3)	22 (10.6)	80 (5.7)
**BMI, kg/m2**					
<18.5	1462 (3.4)	6 (2.9)	1267 (3.0)	1 (0.5)	37 (2.6)
18.5-24.9	13478 (31.3)	61 (29.2)	13132 (30.9)	70 (33.7)	465 (33.2)
25-29.9	12966 (30.1)	57 (27.3)	12606 (29.7)	54 (26.0)	372 (26.6)
30-34.9	6258 (14.5)	37 (17.7)	5704 (13.4)	24 (11.5)	181 (12.9)
35-39.9	1934 (4.5)	16 (7.7)	1735 (4.1)	11 (5.3)	78 (5.6)
40+	949 (2.2)	8 (3.8)	782 (1.8)	8 (3.8)	57 (4.1)
Missing	5986 (13.9)	24 (11.5)	7262 (17.1)	40 (19.2)	211 (15.1)

Hopkins R, *et al. BMJ Open* 2024; 14:e078135. doi: 10.1136/bmjopen-2023-078135

Supplemental figure 5. Association of potential risk factors with hospitalisation for Covid-19 (2020), influenza (2016-2019), and pneumonia (2016-2019) in type 1 diabetes.

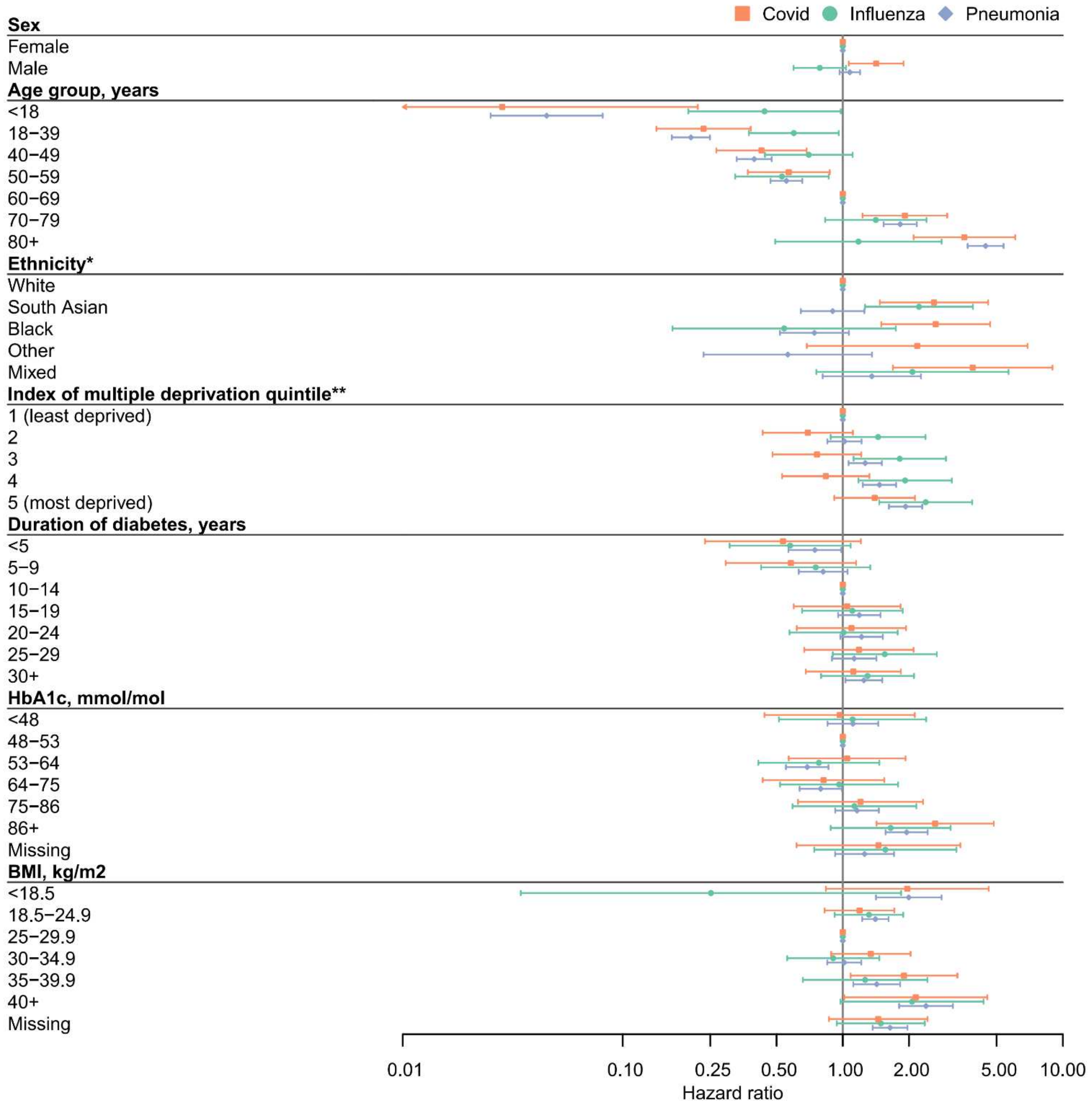

*No influenza hospitalisations occurred in other ethnicity group. No Covid-19, influenza or pneumonia hospitalisations occurred in unknown ethnicity group. ** No Covid-19, influenza or pneumonia hospitalisations occurred in missing IMD group.

Supplemental figure 6A. Association of continuous HbA1c with hospitalisation for Covid-19 (2020), influenza (2016-2019), and pneumonia (2016-2019) in type 1 diabetes. Density plots show the distribution of HbA1c in each group.

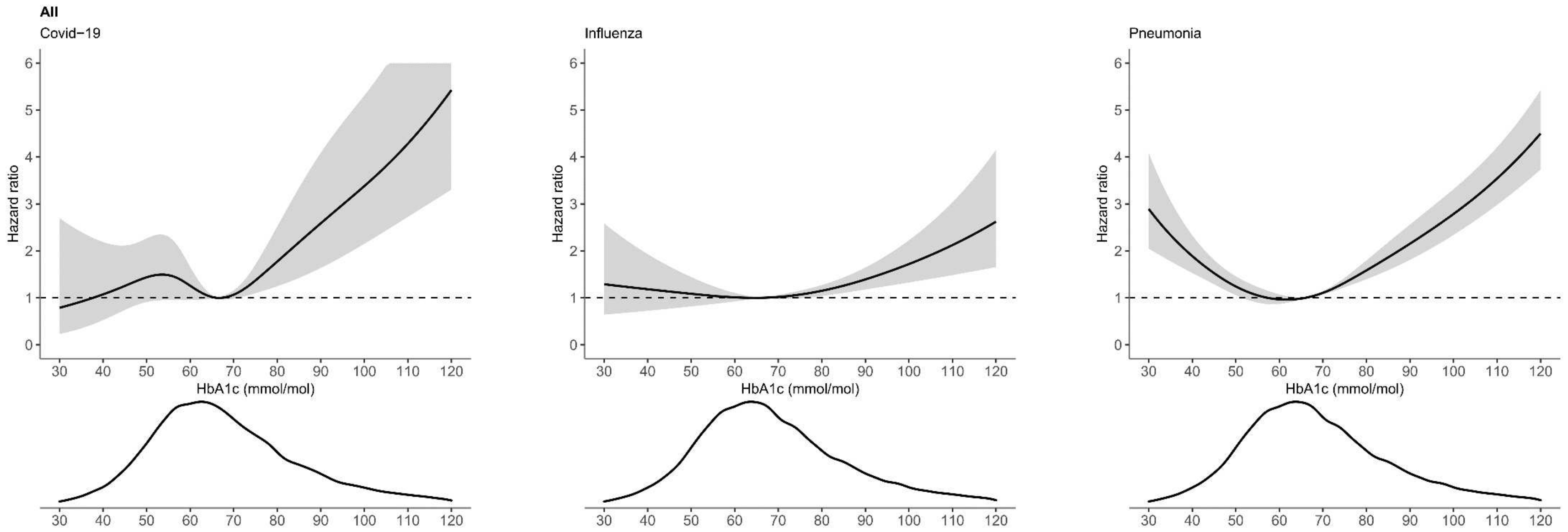

Supplemental figure 6B. Association of continuous BMI with hospitalisation for Covid-19 (2020), influenza (2016-2019), and pneumonia (2016-2019) in type 1 diabetes. Density plots show the distribution of BMI in each group.

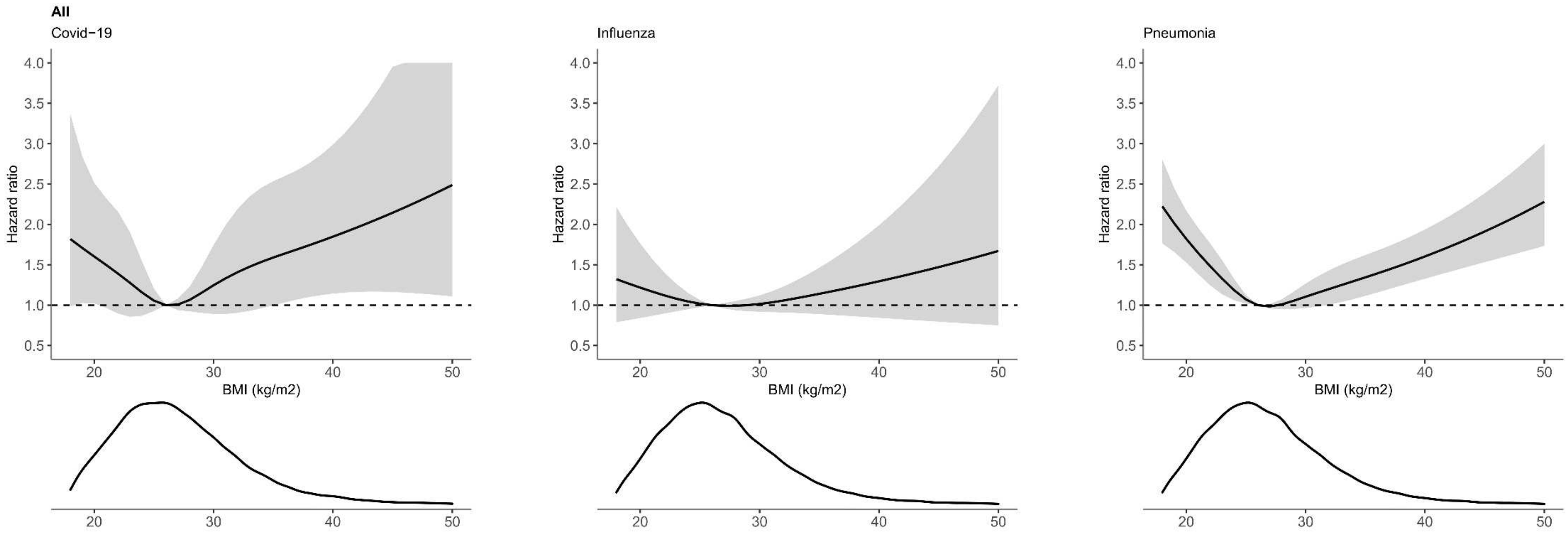

Supplemental table 8. Baseline characteristics and Covid-19, influenza, and pneumonia hospitalisations in type 1 diabetes, restricting hospitalisation definition to primary diagnosis in HES.

	2020 cohort	Covid-19 hospitalisations (in 2020 cohort)	2016 cohort	Influenza hospitalisations (in 2016 cohort)	Pneumonia hospitalisations (in 2016 cohort)
**Number of individuals**	43033	142	42488	130	803
**Mean (SD) follow-up, days**		267.2 (32.6)		917.9 (222.4)	912.1 (230.3)
**Sex**					
Female	18172 (42.2)	45 (31.7)	17889 (42.1)	68 (52.3)	353 (44.0)
Male	24861 (57.8)	97 (68.3)	24599 (57.9)	62 (47.7)	450 (56.0)
**Age, years**					
<18	4458 (10.4)	0 (0.0)	4502 (10.6)	6 (4.6)	9 (1.1)
18-39	14454 (33.6)	20 (14.1)	13996 (32.9)	35 (26.9)	102 (12.7)
40-49	7480 (17.4)	21 (14.8)	7995 (18.8)	22 (16.9)	115 (14.3)
50-59	8056 (18.7)	31 (21.8)	7911 (18.6)	21 (16.2)	154 (19.2)
60-69	5079 (11.8)	31 (21.8)	4731 (11.1)	25 (19.2)	157 (19.6)
70-79	2689 (6.2)	24 (16.9)	2460 (5.8)	17 (13.1)	148 (18.4)
80+	817 (1.9)	15 (10.6)	893 (2.1)	4 (3.1)	118 (14.7)
**Ethnicity**					
White	38478 (89.4)	111 (78.2)	38337 (90.2)	121 (93.1)	754 (93.9)
South Asian	1576 (3.7)	12 (8.5)	1494 (3.5)	8 (6.2)	17 (2.1)
Black	1385 (3.2)	12 (8.5)	1253 (2.9)	0 (0.0)	20 (2.5)
Other	464 (1.1)	2 (1.4)	373 (0.9)	0 (0.0)	3 (0.4)
Mixed	525 (1.2)	5 (3.5)	476 (1.1)	1 (0.8)	9 (1.1)
Unknown	605 (1.4)	0 (0.0)	555 (1.3)	0 (0.0)	0 (0.0)
**Index of multiple deprivation quintile**					
1 (least deprived)	9395 (21.8)	26 (18.3)	9251 (21.8)	22 (16.9)	157 (19.6)
2	8710 (20.2)	20 (14.1)	8652 (20.4)	21 (16.2)	143 (17.8)
3	8334 (19.4)	28 (19.7)	8347 (19.6)	27 (20.8)	163 (20.3)
4	8444 (19.6)	29 (20.4)	8342 (19.6)	26 (20.0)	141 (17.6)
5 (most deprived)	8128 (18.9)	39 (27.5)	7864 (18.5)	34 (26.2)	199 (24.8)
Missing	22 (0.1)	0 (0.0)	32 (0.1)	0 (0.0)	0 (0.0)
**Duration of diagnosed diabetes, years**					
<5	5655 (13.1)	4 (2.8)	6556 (15.4)	7 (5.4)	55 (6.8)
5-9	6194 (14.4)	10 (7.0)	6077 (14.3)	14 (10.8)	58 (7.2)
10-14	5609 (13.0)	18 (12.7)	5899 (13.9)	14 (10.8)	72 (9.0)
15-19	5466 (12.7)	21 (14.8)	5355 (12.6)	17 (13.1)	105 (13.1)
20-24	4621 (10.7)	11 (7.7)	4612 (10.9)	11 (8.5)	95 (11.8)
25-29	4101 (9.5)	22 (15.5)	3747 (8.8)	22 (16.9)	83 (10.3)
30+	11387 (26.5)	56 (39.4)	10242 (24.1)	45 (34.6)	335 (41.7)
**HbA1c, mmol/mol**					
<48	3104 (7.2)	8 (5.6)	2796 (6.6)	9 (6.9)	67 (8.3)
48-53	3232 (7.5)	8 (5.6)	2967 (7.0)	11 (8.5)	66 (8.2)
53-64	10995 (25.6)	35 (24.6)	10340 (24.3)	25 (19.2)	159 (19.8)
64-75	9951 (23.1)	28 (19.7)	10098 (23.8)	32 (24.6)	167 (20.8)
75-86	5891 (13.7)	22 (15.5)	5979 (14.1)	21 (16.2)	117 (14.6)
86+	6097 (14.2)	33 (23.2)	6351 (14.9)	22 (16.9)	179 (22.3)
Missing	3763 (8.7)	8 (5.6)	3957 (9.3)	10 (7.7)	48 (6.0)
**BMI, kg/m2**					
<18.5	1462 (3.4)	2 (1.4)	1267 (3.0)	1 (0.8)	23 (2.9)
18.5-24.9	13478 (31.3)	39 (27.5)	13132 (30.9)	45 (34.6)	267 (33.3)
25-29.9	12966 (30.1)	40 (28.2)	12606 (29.7)	35 (26.9)	215 (26.8)
30-34.9	6258 (14.5)	24 (16.9)	5704 (13.4)	16 (12.3)	95 (11.8)
35-39.9	1934 (4.5)	11 (7.7)	1735 (4.1)	7 (5.4)	53 (6.6)
40+	949 (2.2)	8 (5.6)	782 (1.8)	5 (3.8)	35 (4.4)
Missing	5986 (13.9)	18 (12.7)	7262 (17.1)	21 (16.2)	115 (14.3)

Hopkins R, *et al. BMJ Open* 2024; 14:e078135. doi: 10.1136/bmjopen-2023-078135

Supplemental figure 7. Association of potential risk factors with hospitalisation for Covid-19 (2020), influenza (2016-2019), and pneumonia (2016-2019) in type 1 diabetes, restricting hospitalisation definition to primary diagnosis in HES.

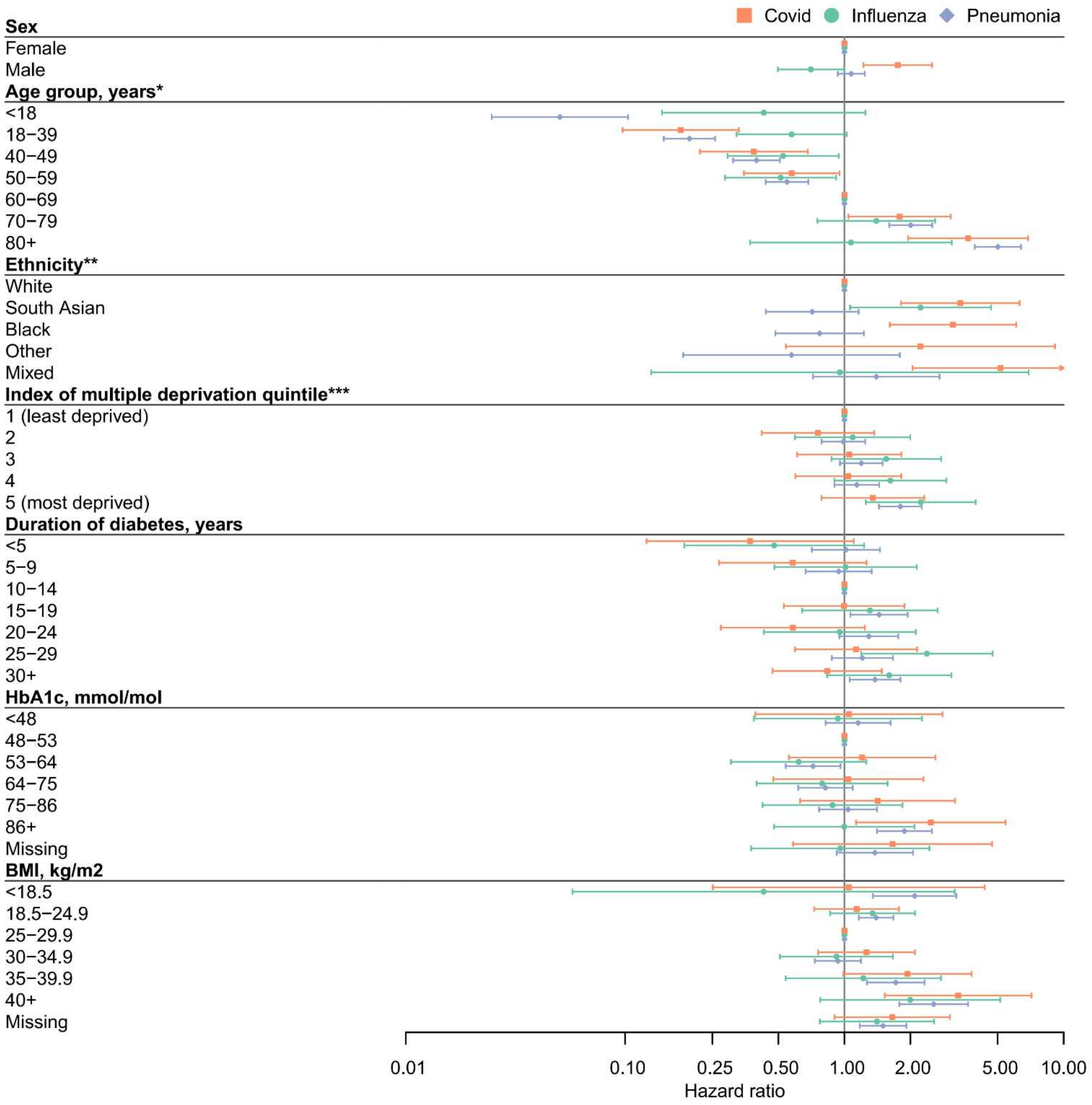

*No Covid-19 hospitalisations occurred in <18 group. **No influenza hospitalisation occurred in Black or other ethnicity groups. No Covid-19, influenza or pneumonia hospitalisation occurred in the unknown ethnicity group. *** No Covid-19, influenza or pneumonia hospitalisations occurred in missing IMD group.

Hopkins R, *et al. BMJ Open* 2024; 14:e078135. doi: 10.1136/bmjopen-2023-078135

Supplemental table 9. Baseline characteristics and Covid-19, influenza, and pneumonia hospitalisations in type 2 diabetes, restricting hospitalisation definition to primary diagnosis in HES.

	2020 cohort	Covid-19 hospitalisations (in 2020 cohort)	2016 cohort	Influenza hospitalisations (in 2016 cohort)	Pneumonia hospitalisations (in 2016 cohort)
**Number of individuals**	584854	4781	585289	2189	24944
**Mean (SD) follow-up, days**		265.3 (37.7)		915.7 (224.3)	903.4 (239.5)
**Sex**					
Female	253360 (43.3)	1802 (37.7)	256656 (43.9)	1068 (48.8)	11337 (45.4)
Male	331494 (56.7)	2979 (62.3)	328633 (56.1)	1121 (51.2)	13607 (54.6)
**Age group, years**					
<40	12898 (2.2)	61 (1.3)	13468 (2.3)	28 (1.3)	111 (0.4)
40-49	46075 (7.9)	220 (4.6)	49649 (8.5)	93 (4.2)	453 (1.8)
50-59	114537 (19.6)	625 (13.1)	113285 (19.4)	216 (9.9)	1599 (6.4)
60-69	151493 (25.9)	976 (20.4)	153782 (26.3)	456 (20.8)	3900 (15.6)
70-79	155424 (26.6)	1312 (27.4)	152401 (26.0)	732 (33.4)	7893 (31.6)
80-89	89871 (15.4)	1309 (27.4)	88492 (15.1)	559 (25.5)	8788 (35.2)
90+	14556 (2.5)	278 (5.8)	14212 (2.4)	105 (4.8)	2200 (8.8)
**Ethnicity**					
White	445160 (76.1)	3240 (67.8)	457714 (78.2)	1746 (79.8)	21923 (87.9)
South Asian	77253 (13.2)	766 (16.0)	71050 (12.1)	282 (12.9)	1871 (7.5)
Black	35144 (6.0)	575 (12.0)	33175 (5.7)	105 (4.8)	771 (3.1)
Other	9886 (1.7)	132 (2.8)	8178 (1.4)	25 (1.1)	190 (0.8)
Mixed	6267 (1.1)	56 (1.2)	5654 (1.0)	25 (1.1)	149 (0.6)
Unknown	11144 (1.9)	12 (0.3)	9518 (1.6)	6 (0.3)	40 (0.2)
**Index of multiple deprivation quintile**					
1 (least deprived)	102950 (17.6)	607 (12.7)	103184 (17.6)	332 (15.2)	4085 (16.4)
2	106968 (18.3)	715 (15.0)	109006 (18.6)	368 (16.8)	4559 (18.3)
3	113454 (19.4)	861 (18.0)	113661 (19.4)	422 (19.3)	4765 (19.1)
4	127048 (21.7)	1186 (24.8)	125492 (21.4)	477 (21.8)	5330 (21.4)
5 (most deprived)	134146 (22.9)	1410 (29.5)	133563 (22.8)	589 (26.9)	6190 (24.8)
Missing	288 (0.0)	2 (0.0)	383 (0.1)	1 (0.0)	15 (0.1)
**Duration of diagnosed diabetes, years**					
<1	14311 (2.4)	60 (1.3)	36503 (6.2)	98 (4.5)	953 (3.8)
1-2	60219 (10.3)	337 (7.0)	77510 (13.2)	216 (9.9)	2247 (9.0)
3-5	105094 (18.0)	652 (13.6)	111199 (19.0)	334 (15.3)	3429 (13.7)
6-9	128817 (22.0)	927 (19.4)	125083 (21.4)	428 (19.6)	4686 (18.8)
10-14	124471 (21.3)	967 (20.2)	126421 (21.6)	526 (24.0)	6378 (25.6)
15-19	89704 (15.3)	972 (20.3)	62373 (10.7)	302 (13.8)	3652 (14.6)
20+	62238 (10.6)	866 (18.1)	46200 (7.9)	285 (13.0)	3599 (14.4)
**HbA1c, mmol/mol**					
<48	167739 (28.7)	1327 (27.8)	175659 (30.0)	634 (29.0)	8049 (32.3)
48-53	116015 (19.8)	840 (17.6)	116131 (19.8)	392 (17.9)	4701 (18.8)
53-64	147209 (25.2)	1093 (22.9)	142662 (24.4)	532 (24.3)	5861 (23.5)
64-75	66071 (11.3)	618 (12.9)	62291 (10.6)	289 (13.2)	2606 (10.4)
75-86	33970 (5.8)	335 (7.0)	32257 (5.5)	127 (5.8)	1422 (5.7)
86+	39727 (6.8)	467 (9.8)	37470 (6.4)	165 (7.5)	1676 (6.7)
Missing	14123 (2.4)	101 (2.1)	18819 (3.2)	50 (2.3)	629 (2.5)
**Number of microvascular complications**					
0	264011 (45.1)	1550 (32.4)	281811 (48.1)	761 (34.8)	7915 (31.7)
1	216115 (37.0)	1718 (35.9)	204550 (34.9)	836 (38.2)	9536 (38.2)
2	96948 (16.6)	1261 (26.4)	91671 (15.7)	526 (24.0)	6617 (26.5)
3	7780 (1.3)	252 (5.3)	7257 (1.2)	66 (3.0)	876 (3.5)
**BMI, kg/m2**					
<18.5	3026 (0.5)	48 (1.0)	2801 (0.5)	12 (0.5)	362 (1.5)
18.5-24.9	86199 (14.7)	752 (15.7)	79018 (13.5)	310 (14.2)	4694 (18.8)
25-29.9	184626 (31.6)	1418 (29.7)	178501 (30.5)	614 (28.0)	7025 (28.2)
30-34.9	151732 (25.9)	1100 (23.0)	151161 (25.8)	551 (25.2)	5329 (21.4)
35-39.9	74766 (12.8)	612 (12.8)	75339 (12.9)	289 (13.2)	2674 (10.7)
40+	48455 (8.3)	441 (9.2)	48685 (8.3)	195 (8.9)	1857 (7.4)
Missing	36050 (6.2)	410 (8.6)	49784 (8.5)	218 (10.0)	3003 (12.0)
**Smoking status**					
Active smoker	87220 (14.9)	403 (8.4)	90218 (15.4)	364 (16.6)	4168 (16.7)
Ex-smoker	336719 (57.6)	3124 (65.3)	316723 (54.1)	1266 (57.8)	14903 (59.7)
Non-smoker	138840 (23.7)	1035 (21.6)	149524 (25.5)	436 (19.9)	4558 (18.3)
Unknown	22075 (3.8)	219 (4.6)	28824 (4.9)	123 (5.6)	1315 (5.3)
**Comorbidities**					
**Cardiovascular**					
Hypertension	425278 (72.7)	4017 (84.0)	419645 (71.7)	1818 (83.1)	21575 (86.5)
Atrial fibrillation	70633 (12.1)	1078 (22.5)	63872 (10.9)	473 (21.6)	6940 (27.8)
Angina	87970 (15.0)	1215 (25.4)	91026 (15.6)	596 (27.2)	7284 (29.2)
Previous myocardial infarction	63738 (10.9)	983 (20.6)	60576 (10.3)	422 (19.3)	5472 (21.9)
Previous cardiac revascularisation	57332 (9.8)	742 (15.5)	54961 (9.4)	317 (14.5)	3853 (15.4)
Other ischaemic heart disease	121776 (20.8)	1669 (34.9)	121753 (20.8)	766 (35.0)	9695 (38.9)

	2020 cohort	Covid-19 hospitalisations (in 2020 cohort)	2016 cohort	Influenza hospitalisations (in 2016 cohort)	Pneumonia hospitalisations (in 2016 cohort)
Heart failure	61014 (10.4)	1243 (26.0)	53579 (9.2)	469 (21.4)	6932 (27.8)
Peripheral arterial disease	50045 (8.6)	895 (18.7)	48489 (8.3)	301 (13.8)	4970 (19.9)
**Recent hospitalisation**					
Respiratory infection	25535 (4.4)	817 (17.1)	21353 (3.6)	280 (12.8)	4469 (17.9)
Anything else	96421 (16.5)	1890 (39.5)	95232 (16.3)	794 (36.3)	10039 (40.2)
**Respiratory**					
Asthma	125582 (21.5)	1275 (26.7)	118207 (20.2)	701 (32.0)	7866 (31.5)
Chronic obstructive pulmonary disease	65615 (11.2)	1022 (21.4)	60108 (10.3)	534 (24.4)	8088 (32.4)
**Neurological**					
Previous transient ischaemic attack	31024 (5.3)	463 (9.7)	29997 (5.1)	203 (9.3)	2812 (11.3)
Previous stroke	49873 (8.5)	763 (16.0)	47721 (8.2)	341 (15.6)	4574 (18.3)
Dementia	20629 (3.5)	573 (12.0)	19548 (3.3)	149 (6.8)	2611 (10.5)
Other neurological condition	35000 (6.0)	584 (12.2)	33569 (5.7)	240 (11.0)	3309 (13.3)
**Oncological**					
Haematological cancer	11751 (2.0)	162 (3.4)	10352 (1.8)	97 (4.4)	990 (4.0)
Solid cancer	79179 (13.5)	855 (17.9)	72001 (12.3)	341 (15.6)	5206 (20.9)
**Other**					
Solid organ transplant	3682 (0.6)	82 (1.7)	3013 (0.5)	32 (1.5)	332 (1.3)
Chronic liver disease	69136 (11.8)	670 (14.0)	48011 (8.2)	210 (9.6)	2142 (8.6)
**Chronic Kidney Disease (CKD) stage**					
Stage 1	165845 (28.4)	790 (16.5)	147798 (25.3)	299 (13.7)	2502 (10.0)
Stage 2	286696 (49.0)	1940 (40.6)	290821 (49.7)	971 (44.4)	10121 (40.6)
Stage 3a	72539 (12.4)	841 (17.6)	80222 (13.7)	453 (20.7)	5273 (21.1)
Stage 3b	33246 (5.7)	544 (11.4)	37256 (6.4)	237 (10.8)	3818 (15.3)
Stage 4	9006 (1.5)	230 (4.8)	10490 (1.8)	88 (4.0)	1473 (5.9)
Stage 5	12871 (2.2)	421 (8.8)	13365 (2.3)	130 (5.9)	1629 (6.5)
Missing	4651 (0.8)	15 (0.3)	5337 (0.9)	11 (0.5)	128 (0.5)
**Albumin creatinine ratio (ACR) category**					
A1	402847 (68.9)	2655 (55.5)	388839 (66.4)	1252 (57.2)	13184 (52.9)
A2	121002 (20.7)	1339 (28.0)	106879 (18.3)	565 (25.8)	7450 (29.9)
A3	21899 (3.7)	495 (10.4)	18817 (3.2)	153 (7.0)	2033 (8.2)
Missing	39106 (6.7)	292 (6.1)	70754 (12.1)	219 (10.0)	2277 (9.1)
**Diabetes treatment (last 6 months)**					
Insulin prescription	79286 (13.6)	1190 (24.9)	78755 (13.5)	468 (21.4)	5380 (21.6)
OHA prescription only	362651 (62.0)	2584 (54.0)	349629 (59.7)	1215 (55.5)	12993 (52.1)
No treatment	142917 (24.4)	1007 (21.1)	156905 (26.8)	506 (23.1)	6571 (26.3)

Supplemental figure 8. Association of potential risk factors with hospitalisation for Covid-19 (2020), influenza (2016-2019), and pneumonia (2016-2019) in type 2 diabetes, restricting hospitalisation definition to primary diagnosis in HES.

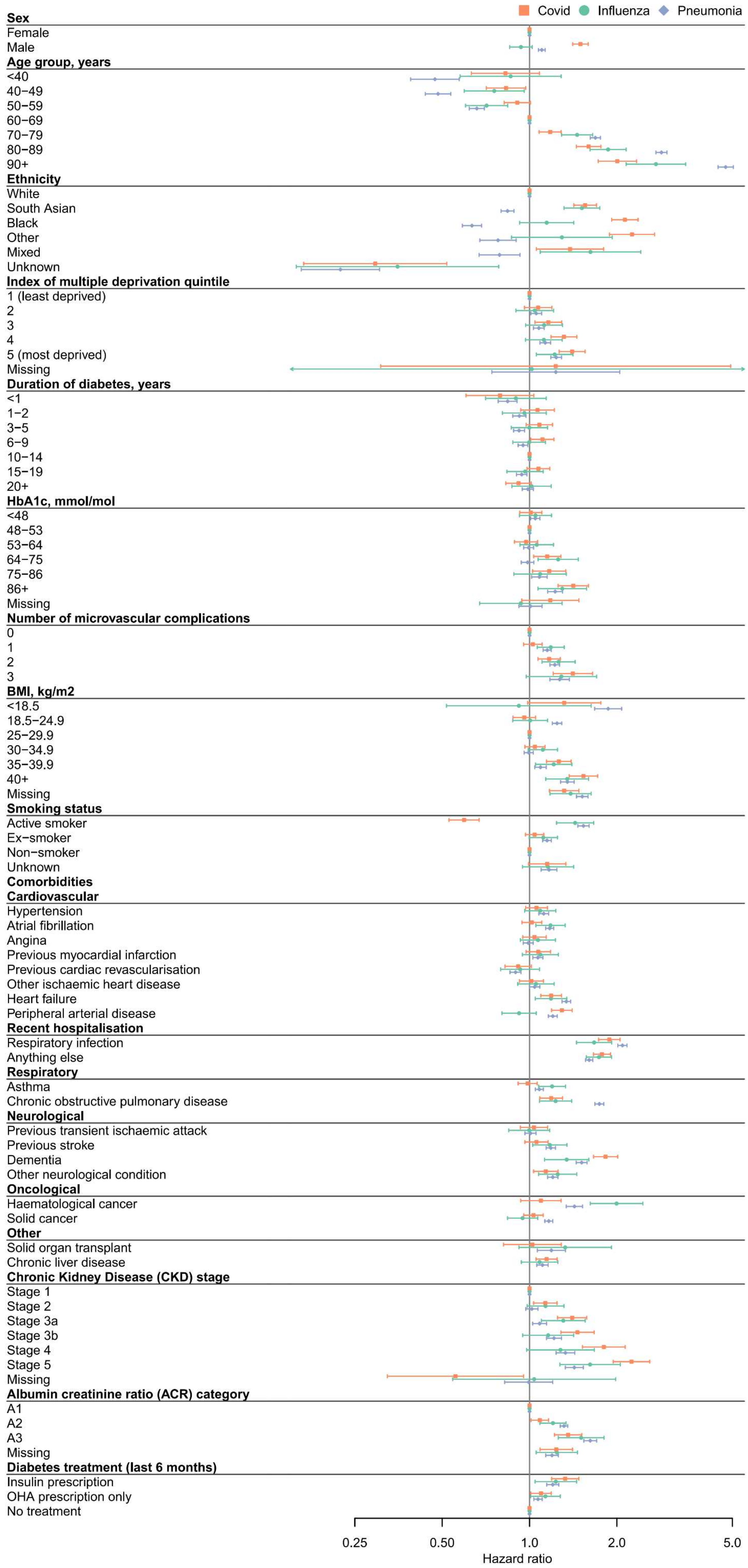

Hopkins R, *et al. BMJ Open* 2024; 14:e078135. doi: 10.1136/bmjopen-2023-078135

Supplemental table 10. Baseline characteristics and Covid-19 and pneumonia deaths in type 2 diabetes, restricting mortality definition to primary cause in ONS.

	2020 cohort	Covid-19 deaths (in 2020 cohort)	2016 cohort	Pneumonia deaths (in 2016 cohort)
**Number of individuals**	584854	2086	585289	2653
**Mean (SD) follow-up, days**		266.1 (35.5)		917.0 (223.2)
**Sex**				
Female	253360 (43.3)	796 (38.2)	256656 (43.9)	1268 (47.8)
Male	331494 (56.7)	1290 (61.8)	328633 (56.1)	1385 (52.2)
**Age group, years**				
<40	12898 (2.2)	6 (0.3)	13468 (2.3)	1 (0.0)
40-49	46075 (7.9)	30 (1.4)	49649 (8.5)	23 (0.9)
50-59	114537 (19.6)	118 (5.7)	113285 (19.4)	52 (2.0)
60-69	151493 (25.9)	299 (14.3)	153782 (26.3)	199 (7.5)
70-79	155424 (26.6)	563 (27.0)	152401 (26.0)	566 (21.3)
80-89	89871 (15.4)	847 (40.6)	88492 (15.1)	1227 (46.2)
90+	14556 (2.5)	223 (10.7)	14212 (2.4)	585 (22.1)
**Ethnicity**				
White	445160 (76.1)	1514 (72.6)	457714 (78.2)	2399 (90.4)
South Asian	77253 (13.2)	289 (13.9)	71050 (12.1)	160 (6.0)
Black	35144 (6.0)	216 (10.4)	33175 (5.7)	55 (2.1)
Other	9886 (1.7)	42 (2.0)	8178 (1.4)	20 (0.8)
Mixed	6267 (1.1)	18 (0.9)	5654 (1.0)	10 (0.4)
Unknown	11144 (1.9)	7 (0.3)	9518 (1.6)	9 (0.3)
**Index of multiple deprivation quintile**				
1 (least deprived)	102950 (17.6)	281 (13.5)	103184 (17.6)	493 (18.6)
2	106968 (18.3)	330 (15.8)	109006 (18.6)	490 (18.5)
3	113454 (19.4)	397 (19.0)	113661 (19.4)	525 (19.8)
4	127048 (21.7)	475 (22.8)	125492 (21.4)	550 (20.7)
5 (most deprived)	134146 (22.9)	602 (28.9)	133563 (22.8)	595 (22.4)
Missing	288 (0.0)	1 (0.0)	383 (0.1)	0 (0.0)
**Duration of diagnosed diabetes, years**				
<1	14311 (2.4)	10 (0.5)	36503 (6.2)	53 (2.0)
1-2	60219 (10.3)	93 (4.5)	77510 (13.2)	157 (5.9)
3-5	105094 (18.0)	257 (12.3)	111199 (19.0)	267 (10.1)
6-9	128817 (22.0)	343 (16.4)	125083 (21.4)	452 (17.0)
10-14	124471 (21.3)	443 (21.2)	126421 (21.6)	732 (27.6)
15-19	89704 (15.3)	490 (23.5)	62373 (10.7)	498 (18.8)
20+	62238 (10.6)	450 (21.6)	46200 (7.9)	494 (18.6)
**HbA1c, mmol/mol**				
<48	167739 (28.7)	655 (31.4)	175659 (30.0)	913 (34.4)
48-53	116015 (19.8)	351 (16.8)	116131 (19.8)	498 (18.8)
53-64	147209 (25.2)	462 (22.1)	142662 (24.4)	612 (23.1)
64-75	66071 (11.3)	266 (12.8)	62291 (10.6)	259 (9.8)
75-86	33970 (5.8)	134 (6.4)	32257 (5.5)	135 (5.1)
86+	39727 (6.8)	156 (7.5)	37470 (6.4)	150 (5.7)
Missing	14123 (2.4)	62 (3.0)	18819 (3.2)	86 (3.2)
**Number of microvascular complications**				
0	264011 (45.1)	564 (27.0)	281811 (48.1)	641 (24.2)
1	216115 (37.0)	772 (37.0)	204550 (34.9)	1009 (38.0)
2	96948 (16.6)	629 (30.2)	91671 (15.7)	884 (33.3)
3	7780 (1.3)	121 (5.8)	7257 (1.2)	119 (4.5)
**BMI, kg/m2**				
<18.5	3026 (0.5)	36 (1.7)	2801 (0.5)	55 (2.1)
18.5-24.9	86199 (14.7)	466 (22.3)	79018 (13.5)	664 (25.0)
25-29.9	184626 (31.6)	597 (28.6)	178501 (30.5)	726 (27.4)
30-34.9	151732 (25.9)	418 (20.0)	151161 (25.8)	395 (14.9)
35-39.9	74766 (12.8)	197 (9.4)	75339 (12.9)	183 (6.9)
40+	48455 (8.3)	125 (6.0)	48685 (8.3)	138 (5.2)
Missing	36050 (6.2)	247 (11.8)	49784 (8.5)	492 (18.5)
**Smoking status**				
Active smoker	87220 (14.9)	179 (8.6)	90218 (15.4)	320 (12.1)
Ex-smoker	336719 (57.6)	1377 (66.0)	316723 (54.1)	1566 (59.0)
Non-smoker	138840 (23.7)	441 (21.1)	149524 (25.5)	630 (23.7)
Unknown	22075 (3.8)	89 (4.3)	28824 (4.9)	137 (5.2)
**Comorbidities**				
**Cardiovascular**				
Hypertension	425278 (72.7)	1853 (88.8)	419645 (71.7)	2402 (90.5)
Atrial fibrillation	70633 (12.1)	612 (29.3)	63872 (10.9)	946 (35.7)
Angina	87970 (15.0)	599 (28.7)	91026 (15.6)	812 (30.6)
Previous myocardial infarction	63738 (10.9)	497 (23.8)	60576 (10.3)	650 (24.5)
Previous cardiac revascularisation	57332 (9.8)	363 (17.4)	54961 (9.4)	435 (16.4)
Other ischaemic heart disease	121776 (20.8)	799 (38.3)	121753 (20.8)	1099 (41.4)
Heart failure	61014 (10.4)	658 (31.5)	53579 (9.2)	965 (36.4)

	2020 cohort	Covid-19 deaths (in 2020 cohort)	2016 cohort	Pneumonia deaths (in 2016 cohort)
Peripheral arterial disease	50045 (8.6)	492 (23.6)	48489 (8.3)	693 (26.1)
**Recent hospitalisation**				
Respiratory infection	25535 (4.4)	439 (21.0)	21353 (3.6)	483 (18.2)
Anything else	96421 (16.5)	911 (43.7)	95232 (16.3)	1117 (42.1)
**Respiratory**				
Asthma	125582 (21.5)	543 (26.0)	118207 (20.2)	507 (19.1)
Chronic obstructive pulmonary disease	65615 (11.2)	502 (24.1)	60108 (10.3)	458 (17.3)
**Neurological**				
Previous transient ischaemic attack	31024 (5.3)	265 (12.7)	29997 (5.1)	325 (12.3)
Previous stroke	49873 (8.5)	457 (21.9)	47721 (8.2)	476 (17.9)
Dementia	20629 (3.5)	477 (22.9)	19548 (3.3)	274 (10.3)
Other neurological condition	35000 (6.0)	362 (17.4)	33569 (5.7)	395 (14.9)
**Oncological**				
Haematological cancer	11751 (2.0)	89 (4.3)	10352 (1.8)	62 (2.3)
Solid cancer	79179 (13.5)	412 (19.8)	72001 (12.3)	488 (18.4)
**Other**				
Solid organ transplant	3682 (0.6)	27 (1.3)	3013 (0.5)	47 (1.8)
Chronic liver disease	69136 (11.8)	261 (12.5)	48011 (8.2)	140 (5.3)
**Chronic Kidney Disease (CKD) stage**				
Stage 1	165845 (28.4)	191 (9.2)	147798 (25.3)	128 (4.8)
Stage 2	286696 (49.0)	760 (36.4)	290821 (49.7)	791 (29.8)
Stage 3a	72539 (12.4)	445 (21.3)	80222 (13.7)	595 (22.4)
Stage 3b	33246 (5.7)	336 (16.1)	37256 (6.4)	561 (21.1)
Stage 4	9006 (1.5)	147 (7.0)	10490 (1.8)	295 (11.1)
Stage 5	12871 (2.2)	195 (9.3)	13365 (2.3)	260 (9.8)
Missing	4651 (0.8)	12 (0.6)	5337 (0.9)	23 (0.9)
**Albumin creatinine ratio (ACR) category**				
A1	402847 (68.9)	1013 (48.6)	388839 (66.4)	1197 (45.1)
A2	121002 (20.7)	647 (31.0)	106879 (18.3)	961 (36.2)
A3	21899 (3.7)	251 (12.0)	18817 (3.2)	314 (11.8)
Missing	39106 (6.7)	175 (8.4)	70754 (12.1)	181 (6.8)
**Diabetes treatment (last 6 months)**				
Insulin prescription	79286 (13.6)	536 (25.7)	78755 (13.5)	581 (21.9)
OHA prescription only	362651 (62.0)	1012 (48.5)	349629 (59.7)	1307 (49.3)
No treatment	142917 (24.4)	538 (25.8)	156905 (26.8)	765 (28.8)

Hopkins R, *et al. BMJ Open* 2024; 14:e078135. doi: 10.1136/bmjopen-2023-078135

Supplemental figure 9. Association of potential risk factors with Covid-19 (2020), and pneumonia (2016-2019) mortality in type 2 diabetes, restricting mortality definition to primary cause in ONS.

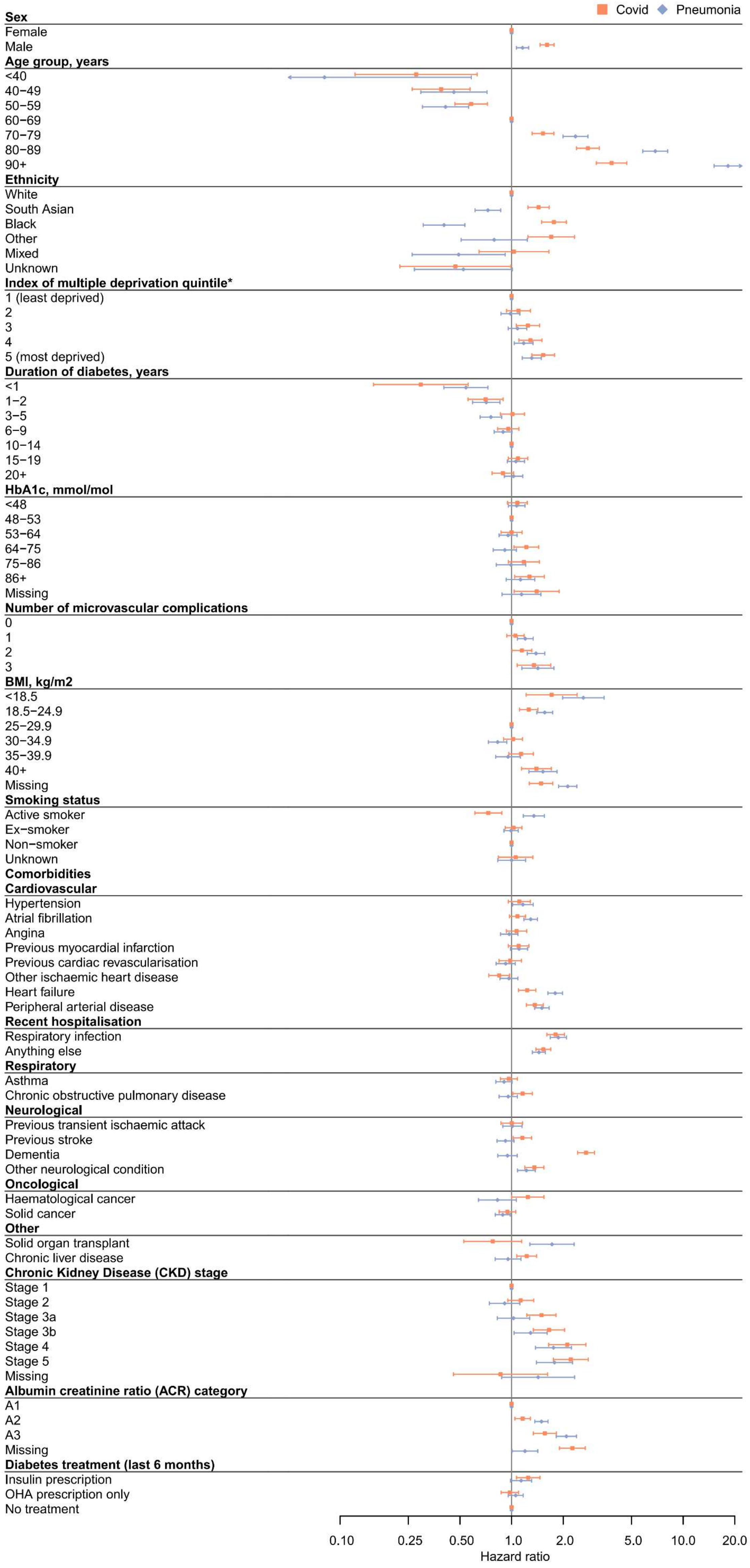

* No pneumonia deaths occurred in missing IMD group. 1 Covid-19 death occurred in missing IMD group.

Supplemental figure 10. Association of potential risk factors with pneumonia hospitalisation (2016-2019) in those with a pneumococcal vaccination and those without.

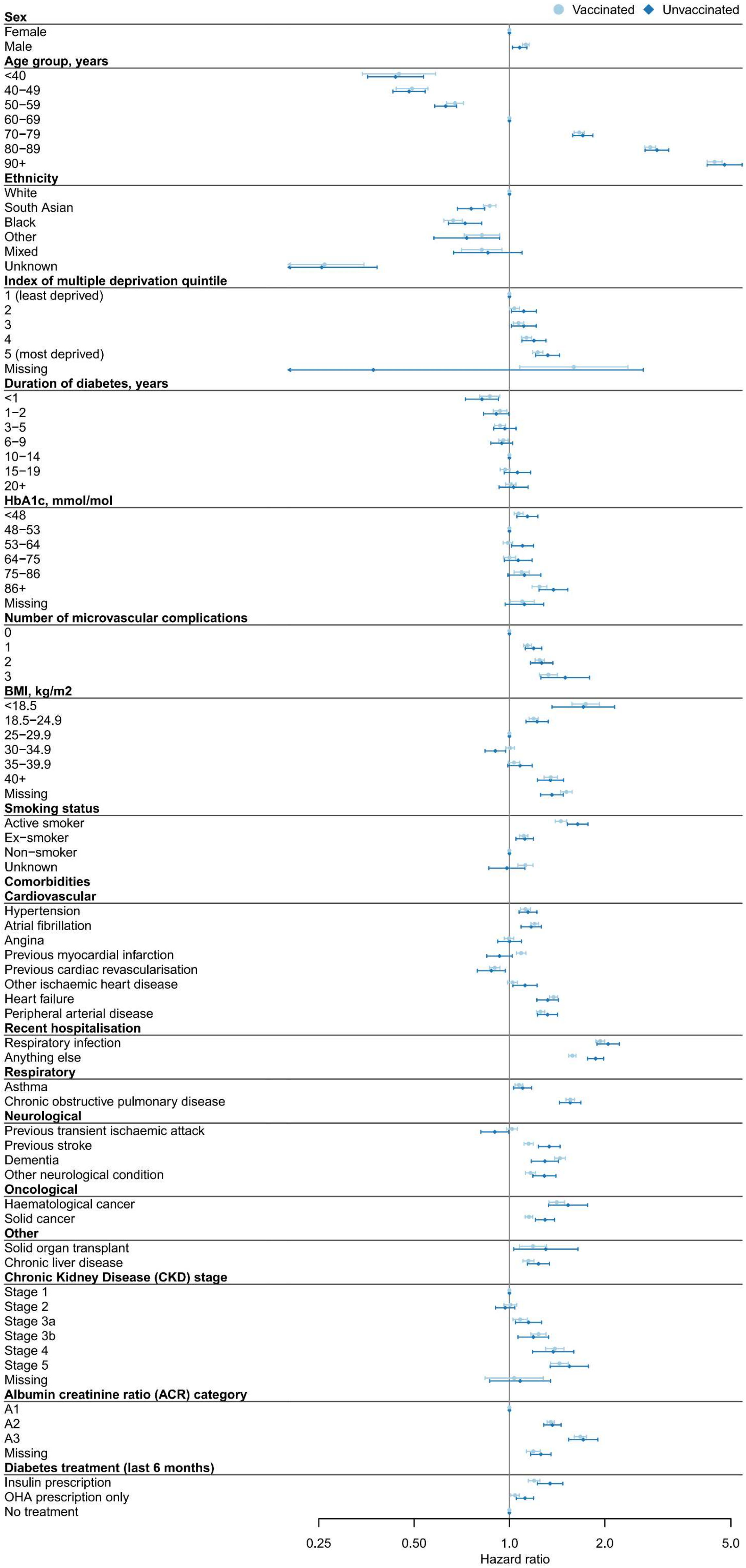

Hopkins R, *et al. BMJ Open* 2024; 14:e078135. doi: 10.1136/bmjopen-2023-078135

Supplemental figure 11. Association of potential risk factors with influenza hospitalisation (2016-2019) in those with an influenza vaccination and those without.

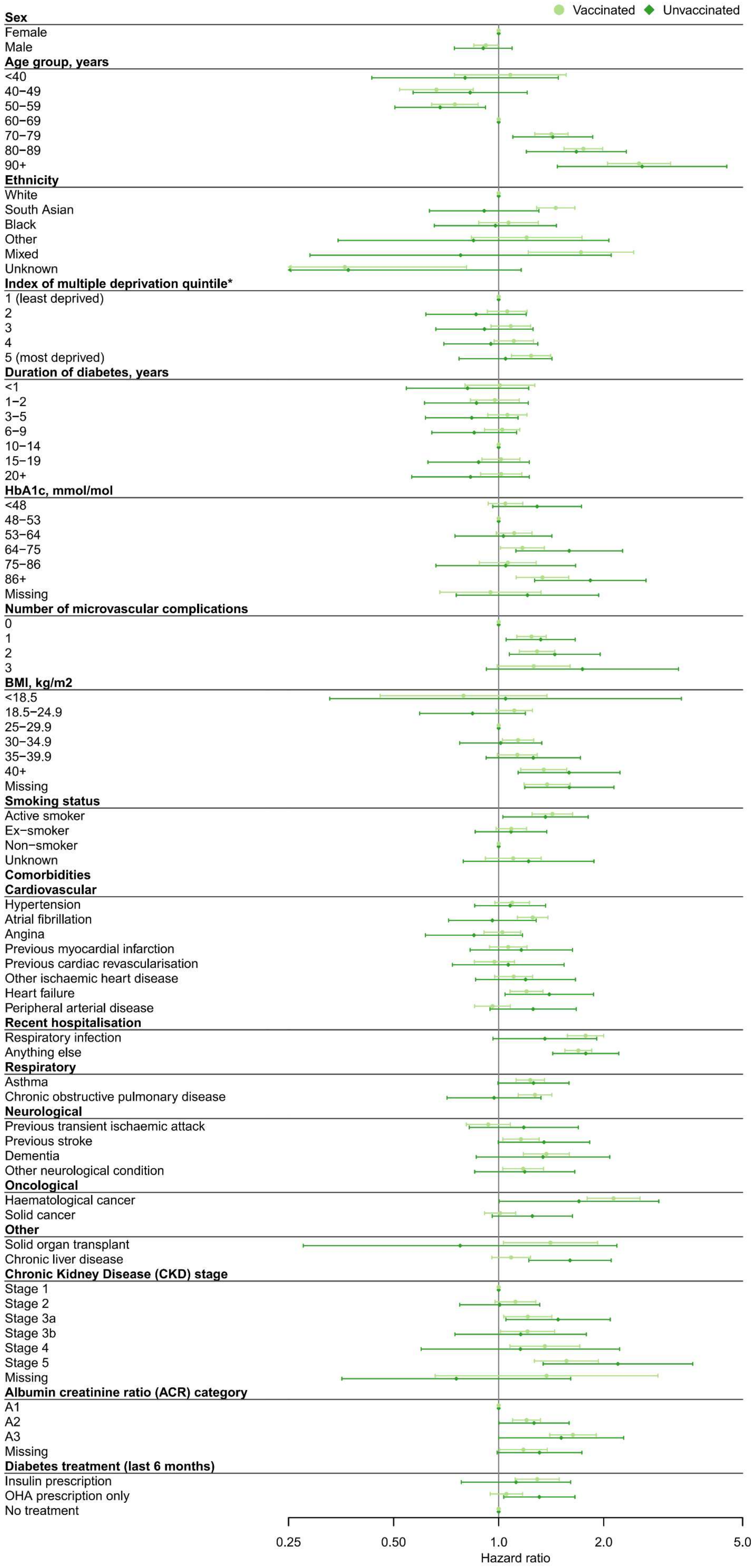

* No influenza hospitalisations occurred in the missing IMD group.

Hopkins R, *et al. BMJ Open* 2024; 14:e078135. doi: 10.1136/bmjopen-2023-078135

References

1. Mathur R, Bhaskaran K, Chaturvedi N, et al. Completeness and usability of ethnicity data in UK-based primary care and hospital databases. J Public Health (Oxf) 2014;36(4): 684-92. doi: 10.1093/pubmed/fdt116

2. GOV.UK. English indices of deprivation 2015. Available: https://www.gov.uk/government/statistics/english-indices-of-deprivation-2015 [Accessed 4 January 2023]

3. World Health Organisation. A healthy lifestyle - WHO recommendations. 2010. Available from https://www.who.int/europe/news-room/fact-sheets/item/a-healthy-lifestyle---who-recommendations [Accessed 4 January 2023]

4. Rodgers LR, Weedon MN, Henley WE, et al. Cohort profile for the MASTERMIND study: using the Clinical Practice Research Datalink (CPRD) to investigate stratification of response to treatment in patients with type 2 diabetes. BMJ Open 2017;7(10): e017989. doi: 10.1136/bmjopen-2017-017989

5. KDIGO. CKD Evaluation and Management. 2012. Available from https://kdigo.org/guidelines/ckd-evaluation-and-management/ [Accessed 4 January 2023]

6. Clift AK, Coupland CAC, Keogh RH, et al. Living risk prediction algorithm (QCOVID) for risk of hospital admission and mortality from coronavirus 19 in adults: national derivation and validation cohort study. BMJ 2020;371: m3731. doi: 10.1136/bmj.m3731

Hopkins R, *et al. BMJ Open* 2024; 14:e078135. doi: 10.1136/bmjopen-2023-078135
