# Supplementary material for: Risk factor associations for severe COVID-19, influenza and pneumonia in people with diabetes to inform future pandemic preparations: UK population-based cohort study
Source: BMJ Open. 2024 Jan 31;14(1):e078135. doi: 10.1136/bmjopen-2023-078135 (PMC10831438; doi:10.1136/bmjopen-2023-078135)
Supplement: Author's manuscript [file bmjopen-2023-078135.draft_revisions.pdf]

BMJ Open

BMJ Open is committed to open peer review. As part of this commitment we make the peer review history of every article we publish publicly available.

When an article is published we post the peer reviewers’ comments and the authors’ responses online. We also post the versions of the paper that were used during peer review. These are the versions that the peer review comments apply to.

The versions of the paper that follow are the versions that were submitted during the peer review process. They are not the versions of record or the final published versions. They should not be cited or distributed as the published version of this manuscript.

BMJ Open is an open access journal and the full, final, typeset and author-corrected version of record of the manuscript is available on our site with no access controls, subscription charges or pay-per-view fees (http://bmjopen.bmj.com).

If you have any questions on BMJ Open’s open peer review process please email info.bmjopen@bmj.com

BMJ Open

Risk factor associations for severe Covid-19, influenza, and pneumonia in people with diabetes to inform future pandemic preparations: UK population-based cohort study

Journal:	*BMJ Open*
Manuscript ID	bmjopen-2023-078135
Article Type:	Original research
Date Submitted by the Author:	25-Jul-2023
Complete List of Authors:	Hopkins, Rhian; University of Exeter Medical School, Institute of Biomedical & Clinical Science<br>Young, Katherine; University of Exeter Medical School, Institute of Biomedical & Clinical Science<br>Thomas, Nick; University of Exeter Medical School, Institute of Biomedical & Clinical Science<br>Godwin, James; University of Exeter Medical School, Institute of Biomedical & Clinical Science<br>Raja, Daniyal; University of Exeter Medical School, Institute of Biomedical & Clinical Science<br>Mateen, Bilal; The Alan Turing Institute; University College London Institute of Health Informatics<br>Challen, Robert; University of Bristol, Engineering Mathematics; NIHR Applied Research Collaboration South West Peninsula<br>Vollmer, Sebastian; University of Warwick Department of Statistics<br>Shields, Beverley; University of Exeter Medical School, Institute of Biomedical & Clinical Science<br>McGovern, Andrew; University of Exeter Medical School, Institute of Biomedical & Clinical Science<br>Dennis, John; University of Exeter Medical School, Institute of Biomedical & Clinical Science
Keywords:	COVID-19, Risk Factors, Electronic Health Records, DIABETES & ENDOCRINOLOGY

SCHOLARONE™
Manuscripts

I, the Submitting Author has the right to grant and does grant on behalf of all authors of the Work (as defined in the below author licence), an exclusive licence and/or a non-exclusive licence for contributions from authors who are: i) UK Crown employees; ii) where BMJ has agreed a CC-BY licence shall apply, and/or iii) in accordance with the terms applicable for US Federal Government officers or employees acting as part of their official duties; on a worldwide, perpetual, irrevocable, royalty-free basis to BMJ Publishing Group Ltd ("BMJ") its licensees and where the relevant Journal is co-owned by BMJ to the co-owners of the Journal, to publish the Work in this journal and any other BMJ products and to exploit all rights, as set out in our licence.

The Submitting Author accepts and understands that any supply made under these terms is made by BMJ to the Submitting Author unless you are acting as an employee on behalf of your employer or a postgraduate student of an affiliated institution which is paying any applicable article publishing charge ("APC") for Open Access articles. Where the Submitting Author wishes to make the Work available on an Open Access basis (and intends to pay the relevant APC), the terms of reuse of such Open Access shall be governed by a Creative Commons licence – details of these licences and which Creative Commons licence will apply to this Work are set out in our licence referred to above.

Other than as permitted in any relevant BMJ Author's Self Archiving Policies, I confirm this Work has not been accepted for publication elsewhere, is not being considered for publication elsewhere and does not duplicate material already published. I confirm all authors consent to publication of this Work and authorise the granting of this licence.

For peer review only

Risk factor associations for severe Covid-19, influenza, and pneumonia in people with diabetes to inform future pandemic preparations: UK population-based cohort study

Word count: 3803

Authors: Rhian Hopkins[1], Katherine G Young[1], Nicholas J Thomas[1], James Godwin[1], Daniyal Raja[1], Bilal A Mateen[2,3], Robert J Challen[4,5], Sebastian J Vollmer[6], Beverley M Shields[1], Andrew P McGovern[1]*, John M Dennis[1]*

*Joint senior

[1]University of Exeter Medical School. Address: Institute of Biomedical & Clinical Science, RILD Building, Royal Devon & Exeter Hospital, Barrack Road, Exeter EX2 5DW, UK

[2]The Alan Turing Institute. Address: British Library, 96 Euston Road, London, NW1 2DB, UK

[3]University College London, Institute of Health Informatics. Address: University College London, 222 Euston Rd, London, NW1 2DA, London, UK

[4]Engineering Mathematics, University of Bristol. Address: Department of Engineering and Mathematics, University of Bristol, Bristol, BS8 1TW, UK

[5]University of Exeter Medical School and the National Institute for Health Research (NIHR) Applied Research Collaboration South West Peninsula (PenARC). Address: University of Exeter, Exeter, EX1 2LU, UK

[6]University of Warwick, Department of Statistics. Address: Department of Statistics, University of Warwick, Coventry, CV4 7AL, UK

Corresponding Author:

Rhian Hopkins

rh530@exeter.ac.uk

For peer review only

Abstract

Objective This study aimed to compare clinical and sociodemographic risk factors for severe Covid-19, influenza, and pneumonia, in people with diabetes.

Design Population-based cohort study.

Setting UK primary care records (Clinical Practice Research Datalink) linked to mortality and hospital records.

Methods We followed individuals with type 1 and type 2 diabetes from 1st February 2020-31st October 2020 (pre-Covid-19 vaccination roll-out, type 2 diabetes: N=584,854, type 1 diabetes: N=43,033) to study Covid-19 hospitalisation (primary outcome) and mortality, and from 1st September 2016-31st May 2019 (pre-Covid-19 pandemic, type 2 diabetes: N=585,289, type 1 diabetes: N=42,488) to study influenza and pneumonia outcomes. Associations between clinical and sociodemographic risk factors and each outcome were assessed using multivariable Cox proportional hazards models. In people with type 2 diabetes, we explored modifying effects of HbA1c and BMI by age, sex, and ethnicity.

Results In type 2 diabetes, poor glycaemic control and severe obesity were consistently associated with increased risk of hospitalisation for Covid-19, influenza, and pneumonia. The highest HbA1c and BMI-associated relative risks were observed in people under 70 years. Sociodemographic-associated risk differed markedly by respiratory infection, particularly for ethnicity. Compared to people of white ethnicity, black and south Asian groups had a greater risk of Covid-19 hospitalisation, but a lesser risk of pneumonia hospitalisation. Risk factor associations for type 1 diabetes and for type 2 diabetes mortality, were broadly consistent with the primary analysis.

Conclusions Clinical risk factors of high HbA1c and severe obesity are consistently associated with severe outcomes from Covid-19, influenza, and pneumonia, especially in younger people. In contrast, associations with sociodemographic risk factors differed by type of respiratory infection. This emphasises that risk stratification should be specific to individual respiratory infections.

For peer review only

Strengths and limitations of this study

- First systematic comparison of risk factors for severe respiratory infection in a large primary care dataset of people with both type 1 and type 2 diabetes with linked hospital records and mortality datasets used to robustly define infection outcomes
- Many potential risk factors, including potentially modifiable factors such as HbA1c and BMI, assessed and included in models guided by previous studies and clinical knowledge
- Sociodemographic risk factor associations, in particular ethnicity differences, may be influenced by factors unique to the Covid-19 pandemic, and knowledge of these differences could inform future risk stratification
- Limitations include the potential for misclassification of infection outcomes, however associations using different outcome definitions were robust in sensitivity analyses
- Associations do not have a causal interpretation but are still useful for identifying those at high risk

For peer review only

Introduction

People with diabetes are twice as likely to die from infection compared to the general population [1]. Infections are also a major cause of morbidity in people with diabetes, with 1 in 4 hospitalisations in men and 1 in 3 hospitalisations in women now being attributable to infections [2]. The burden of respiratory infections is increasing, comprising at least 10% of hospitalisations in people with diabetes in 2018, compared to less than 4% in 2003 [2]. During the Covid-19 pandemic, diabetes was one of the most common comorbidities associated with severe infection and poor outcomes [3-11]. People with type 2 diabetes were twice as likely to die with Covid-19 in hospital as the general population, with the risk being even higher in those with type 1 diabetes [11]. This is concordant with other severe respiratory infections including pneumonia, for which an excess risk of hospitalisation has been observed in people with type 2 diabetes [12], and influenza, for which an increased risk of severe infection has been observed in older adults with diabetes [13].

Despite the high burden of respiratory infection in people with diabetes, there has been no previous population-based comparison of risk factor associations across major respiratory infections. Previous studies have identified clear heterogeneity in the risk of severe Covid-19 in people with diabetes [14-17], with a higher risk in those of older age, male sex, non-white ethnicity, and higher social deprivation [16, 17], and differences by potentially modifiable risk factors, for example increased risk with higher HbA1c and higher BMI [14, 16-19]. Although broadly similar differences in the risk of other severe respiratory infections by sex [20-24], age [20-22], deprivation [20, 22, 25], ethnicity [26], glycaemic control [12, 27] and BMI [28] have been suggested in studies of people with and without diabetes, the consistency of these risk factor associations across respiratory infections has not been robustly established. The limited studies to-date comparing Covid-19 risk factors to influenza and pneumonia have used selectively recruited cohorts, focused on the specific risk factors of sex and obesity [29, 30], or used positive Covid-19 tests/incident infection (influenced by country-specific testing policies) as the outcome [31]. As recently highlighted by the Chief Medical Officer for England, research to provide a robust understanding of the comparability of risk factors for major respiratory infections is urgently needed [32], and could inform future risk stratification and targeted intervention.

In this study in people with diabetes, we aimed to compare clinical and sociodemographic risk factors for Covid-19 hospitalisation and mortality, with risk factors for influenza and pneumonia. We also aimed to assess heterogeneity in potentially modifiable HbA1c and BMI associated risk by age, sex, and ethnicity.

For peer review only

Methods

Data source

We used UK population-based data from the Clinical Practice Research Datalink (CPRD) Aurum, a large database of longitudinal, routinely collected medical records covering demographics, diagnoses, prescriptions, and test results [33]. CPRD Aurum data is sourced from over 2,000 primary care general practitioner (GP) practices from across the UK [34], representing 13% of the population and is largely representative of the broader UK population [33]. We extracted data on all individuals with diabetes actively registered with a GP practice from 1st September 2016 to 31st October 2020. Primary care records were linked to national records of hospital admissions (Hospital Episode Statistics (HES)) and deaths (Office for National Statistics (ONS)). This combined dataset is one of the largest primary and secondary care datasets in the world and gave us a near-complete healthcare and mortality record for over half a million people with diabetes. We also included linked Index of Multiple Deprivation data (the official national measure of deprivation).

Study population

Inclusion and Exclusion Criteria

We included people with type 1 diabetes and type 2 diabetes with valid linkage data (Supplemental figure 1). We classified type 1 diabetes and type 2 diabetes using an algorithm based on diabetes clinical code counts, insulin prescriptions, oral hypoglycaemic agent (OHA) prescriptions, and diagnosis age (algorithm available at https://github.com/Exeter-Diabetes/CPRD-Codelists). The accuracy of this classification approach has recently been confirmed against the use of genetic and biochemical biomarkers of diabetes type [35]. Only those diagnosed with type 2 diabetes aged 20 or over were included to reduce the impact of potential misclassification in young adults. No age restrictions were applied for people with type 1 diabetes.

Cohorts

To study Covid-19, we identified people with diabetes actively registered on 1st February 2020 (Covid-19 baseline date), who were followed up until 31st October 2020 (pre-Covid-19 vaccination roll-out, covering the first wave of Covid-19 and start of the second wave). To study influenza and pneumonia infection outcomes, we defined a pre-Covid-19 pandemic study period, including individuals with diabetes actively registered on 1st September 2016 (influenza/pneumonia baseline date) followed up until 31st May 2019. This study period enabled assessment of influenza and pneumonia outcomes over multiple recent UK influenza seasons prior to Covid-19.

Outcomes

Primary outcomes were first hospitalisation for Covid-19, influenza, or pneumonia during the respective study periods. Outcomes were identified using ICD-10 codes for the infection of interest (Supplemental table 1) recorded as a diagnosis during hospital admission in HES. Secondary outcomes were cause-specific mortality, defined as an ICD-10 code for the

For peer review only - http://bmjopen.bmj.com/site/about/guidelines.xhtml

infection of interest recorded as any cause of death, or hospitalisation for the infection of interest with the discharge method or location recorded as death.

Risk factors and covariates

The risk factors of interest comprised baseline sociodemographic features (sex, age, ethnicity, deprivation), diabetes features (duration of diabetes, HbA1c, microvascular complications (diabetic nephropathy, neuropathy, and retinopathy)), and BMI. We also included a wide range of covariates in our analysis: smoking status, major comorbidities (cardiovascular, respiratory, neurological, and oncological conditions, as well as chronic kidney disease, and recent hospitalisation (respiratory infection, any other cause)), diabetes treatments (no treatment, OHA prescription only, insulin), medications affecting the immune response (immunosuppressants, oral steroids), medications used for long term respiratory conditions (leukotrienes, long acting β agonists), and geographical region. Each comorbidity and medication were included as individual binary variables and missing data was treated as the absence of that covariate. All other covariates were included as categorical variables, and any missing values were grouped into a separate category. Full definitions of each variable are provided in Supplemental table 2, all were defined using primary care and, where appropriate, HES records (codes available at https://github.com/Exeter-Diabetes/CPRD-Codelists).

Statistical analysis

Primary analysis

Multivariable Cox proportional hazards models were used to identify risk factor associations for Covid-19, influenza, and pneumonia hospitalisation, with proportional hazards assumptions for each variable assessed by visual inspection of Schoenfeld residuals. People were followed up until the earliest of; the outcome of interest, deregistration from GP, death, or the end of each study period. Hazard ratios for the potential risk factors in the model were calculated for each infection separately. For people with type 2 diabetes, we fitted separate multivariable models for each infection outcome including all potential risk factors (sex, age, ethnicity, deprivation, diabetes duration, HbA1c, number of microvascular complications, BMI) and covariates (smoking status, comorbidities, diabetes treatment, other medications, and region [full analysis set] (Supplemental table 2)). As the cohort of people with type 1 diabetes was much smaller, we used a reduced risk factor set comprising sex, age, ethnicity, deprivation, diabetes duration, HbA1c, and BMI, with adjustment for region only (restricted analysis set). Analysis of mortality outcomes in people with type 1 diabetes, and mortality from influenza in people with type 2 diabetes, was not performed due to a limited number of deaths in these groups.

HbA1c and BMI specific associations

In people with type 2 diabetes, we assessed the associations of continuous HbA1c and BMI with hospitalisation for each infection outcome, adjusted for the full analysis set. To allow for non-linearity, restricted cubic splines were fitted with 5 knots for Covid-19 and pneumonia and with 3 knots for influenza, with knot selection informed by Akaike information criterion. To explore heterogeneity in risk across sociodemographic subgroups, we refitted the same

For peer review only

models including an interaction between continuous HbA1c/ BMI and subgroups defined by age (over 70 years old/ under 70 years old), sex (male/ female), and ethnicity (white/ black/ south Asian ethnicity). Statistical significance of subgroup interactions were tested using ANOVA. In people with type 1 diabetes, the same approach as for type 2 diabetes was used to assess continuous associations of HbA1c and BMI with hospitalisation outcomes, adjusted for the restricted analysis set. Subgroup analysis was not performed due to low numbers.

Sensitivity analysis

We repeated the primary analysis with restricted outcome definitions where the infection of interest was recorded as a primary diagnosis during the hospital admission or the primary cause of death. To assess the potential impact of influenza and pneumonia vaccination on our findings, we repeated the primary analysis in subgroups with and without a history of vaccination for influenza in the 2 years prior to baseline, and pneumococcal vaccination prior to baseline.

Patient and public involvement

Patients and the public were not involved in this study.

For peer review only

Results

Type 2 diabetes

584,854 people with type 2 diabetes were evaluated for Covid-19 outcomes, of whom 5,965 (1.02%) were hospitalised for Covid-19 from 1st February 2020 to 31st October 2020. 585,289 people with type 2 diabetes were evaluated for pneumonia and influenza outcomes, of whom 38,088 (6.51%) were hospitalised for pneumonia, and 3,226 (0.55%) were hospitalised with influenza from 1st September 2016 to 31st May 2019. Supplemental table 3 details the frequency of ICD10 codes recorded for each infection hospitalisation outcome. The baseline characteristics of the two cohorts were similar (Table 1 and Supplemental table 4). The majority of hospitalisations occurred in the older age groups for all three infections (Table 1). The median age of those hospitalised was 74.8 years for Covid-19, 75.6 years for influenza, and 79.4 years for pneumonia.

Table 1. Baseline clinical and sociodemographic characteristics in each cohort and Covid-19, influenza, and pneumonia hospitalisations in type 2 diabetes.

	2020 cohort	Covid-19 hospitalisations	2016 cohort	Influenza hospitalisations	Pneumonia hospitalisations
**Number of individuals**	584854	5965	585289	3226	38088
**Mean (SD) follow-up, days**		265.0 (38.3)		915.2 (224.8)	897.4 (246.3)
**Sex**					
Female	253360 (43.3)	2317 (38.8)	256656 (43.9)	1581 (49.0)	17140 (45.0)
Male	331494 (56.7)	3648 (61.2)	328633 (56.1)	1645 (51.0)	20948 (55.0)
**Age group, years**					
<40	12898 (2.2)	70 (1.2)	13468 (2.3)	46 (1.4)	164 (0.4)
40-49	46075 (7.9)	258 (4.3)	49649 (8.5)	128 (4.0)	717 (1.9)
50-59	114537 (19.6)	729 (12.2)	113285 (19.4)	329 (10.2)	2495 (6.6)
60-69	151493 (25.9)	1198 (20.1)	153782 (26.3)	679 (21.0)	6068 (15.9)
70-79	155424 (26.6)	1660 (27.8)	152401 (26.0)	1078 (33.4)	12073 (31.7)
80-89	89871 (15.4)	1671 (28.0)	88492 (15.1)	811 (25.1)	13324 (35.0)
90+	14556 (2.5)	379 (6.4)	14212 (2.4)	155 (4.8)	3247 (8.5)
**Ethnicity**					
White	445160 (76.1)	4200 (70.4)	457714 (78.2)	2586 (80.2)	33206 (87.2)
South Asian	77253 (13.2)	891 (14.9)	71050 (12.1)	403 (12.5)	2935 (7.7)
Black	35144 (6.0)	645 (10.8)	33175 (5.7)	156 (4.8)	1324 (3.5)
Other	9886 (1.7)	144 (2.4)	8178 (1.4)	35 (1.1)	306 (0.8)
Mixed	6267 (1.1)	69 (1.2)	5654 (1.0)	37 (1.1)	246 (0.6)
Unknown	11144 (1.9)	16 (0.3)	9518 (1.6)	9 (0.3)	71 (0.2)
**Index of multiple deprivation quintile**					
1 (least deprived)	102950 (17.6)	761 (12.8)	103184 (17.6)	502 (15.6)	6296 (16.5)
2	106968 (18.3)	921 (15.4)	109006 (18.6)	548 (17.0)	6923 (18.2)
3	113454 (19.4)	1088 (18.2)	113661 (19.4)	601 (18.6)	7264 (19.1)
4	127048 (21.7)	1446 (24.2)	125492 (21.4)	698 (21.6)	8199 (21.5)
5 (most deprived)	134146 (22.9)	1747 (29.3)	133563 (22.8)	873 (27.1)	9380 (24.6)
Missing	288 (0.0)	2 (0.0)	383 (0.1)	4 (0.1)	26 (0.1)
**Duration of diagnosed diabetes, years**					
<1	14311 (2.4)	67 (1.1)	36503 (6.2)	146 (4.5)	1401 (3.7)
1-2	60219 (10.3)	397 (6.7)	77510 (13.2)	306 (9.5)	3324 (8.7)
3-5	105094 (18.0)	796 (13.3)	111199 (19.0)	491 (15.2)	5218 (13.7)

For peer review only

	2020 cohort	Covid-19 hospitalisations	2016 cohort	Influenza hospitalisations	Pneumonia hospitalisations
6-9	128817 (22.0)	1116 (18.7)	125083 (21.4)	621 (19.2)	7088 (18.6)
10-14	124471 (21.3)	1250 (21.0)	126421 (21.6)	771 (23.9)	9620 (25.3)
15-19	89704 (15.3)	1225 (20.5)	62373 (10.7)	465 (14.4)	5764 (15.1)
20+	62238 (10.6)	1114 (18.7)	46200 (7.9)	426 (13.2)	5673 (14.9)
**HbA1c, mmol/mol**					
<48	167739 (28.7)	1679 (28.1)	175659 (30.0)	934 (29.0)	12271 (32.2)
48-53	116015 (19.8)	1054 (17.7)	116131 (19.8)	557 (17.3)	7015 (18.4)
53-64	147209 (25.2)	1352 (22.7)	142662 (24.4)	791 (24.5)	8912 (23.4)
64-75	66071 (11.3)	767 (12.9)	62291 (10.6)	412 (12.8)	4062 (10.7)
75-86	33970 (5.8)	408 (6.8)	32257 (5.5)	184 (5.7)	2191 (5.8)
86+	39727 (6.8)	580 (9.7)	37470 (6.4)	270 (8.4)	2646 (6.9)
Missing	14123 (2.4)	125 (2.1)	18819 (3.2)	78 (2.4)	991 (2.6)
**Number of microvascular complications**					
0	264011 (45.1)	1889 (31.7)	281811 (48.1)	1077 (33.4)	11930 (31.3)
1	216115 (37.0)	2140 (35.9)	204550 (34.9)	1256 (38.9)	14444 (37.9)
2	96948 (16.6)	1621 (27.2)	91671 (15.7)	789 (24.5)	10288 (27.0)
3	7780 (1.3)	315 (5.3)	7257 (1.2)	104 (3.2)	1426 (3.7)
**BMI, kg/m2**					
<18.5	3026 (0.5)	66 (1.1)	2801 (0.5)	16 (0.5)	497 (1.3)
18.5-24.9	86199 (14.7)	1009 (16.9)	79018 (13.5)	476 (14.8)	6985 (18.3)
25-29.9	184626 (31.6)	1747 (29.3)	178501 (30.5)	885 (27.4)	10950 (28.7)
30-34.9	151732 (25.9)	1350 (22.6)	151161 (25.8)	809 (25.1)	8249 (21.7)
35-39.9	74766 (12.8)	752 (12.6)	75339 (12.9)	405 (12.6)	3976 (10.4)
40+	48455 (8.3)	517 (8.7)	48685 (8.3)	302 (9.4)	2877 (7.6)
Missing	36050 (6.2)	524 (8.8)	49784 (8.5)	333 (10.3)	4554 (12.0)

Poor glycaemic control and severe obesity are consistent risk factors for Covid-19, influenza, and pneumonia in type 2 diabetes

High HbA1c >86 mmol/mol (>10%), was consistently associated with increased hospitalisation for Covid-19, influenza, and pneumonia (Figure 1). When assessed continuously, there was an increasing risk of Covid-19 and influenza hospitalisation above HbA1c 53 mmol/mol (7%), but a more gradual risk increase for pneumonia (Figure 2). Compared to a HbA1c of 53 mmol/mol (7%), a HbA1c of 75 mmol/mol (9%) was associated with a 15% increased risk of Covid-19 (HR 1.15 95%CI 1.06-1.26), 12% increased risk of influenza (HR 1.12 95%CI 1.07-1.18), and 6% increased risk of pneumonia hospitalisation (HR 1.06 95%CI 1.02-1.10).

There was a U-shaped association with BMI for Covid-19 and pneumonia, and obesity was associated with an increased risk of hospitalisation for all three respiratory infections (Figure 1). There was a stronger association between increasing BMI and Covid-19 hospitalisation than hospitalisation for influenza or pneumonia (Figure 3). Compared to a BMI of 30 kg/m^2, a BMI of 35 kg/m^2 was associated with an 19% increased risk of Covid-19 (HR 1.19 95%CI 1.10-1.27), 8% increased risk of influenza (HR 1.08 95%CI 1.05-1.11), and a 6% increased risk of pneumonia hospitalisation (HR 1.06 95%CI 1.03-1.09).

An increasing number of microvascular complications was also associated with increased hospitalisation for all three respiratory infections (Figure 1). Diabetes duration was not associated with hospitalisation for all 3 infections, except in those with short duration (< 1

For peer review only

year) where there was reduced risk for Covid-19 and pneumonia (Figure 2). Supplemental Figure 2 and Supplemental table 5 report the full set of risk factors studied.

HbA1c and BMI associated risk differs by age and sex

In subgroup analysis, we observed a difference in HbA1c associations by age but not sex; there was a stronger association between higher HbA1c and hospitalisation in those aged under 70 compared to those over 70 for Covid-19 and pneumonia, but not influenza (Figure 2). There was little evidence for a difference by sex (Figure 2). For BMI, there was a stronger association with increasing BMI and hospitalisation in those aged under 70 and in females for all three infections (Figure 3). We observed no clear difference in HbA1c or BMI associations by ethnicity, although numbers were limited (Supplemental Figure 3A, Supplemental Figure 3B).

Non-white ethnicity, male sex, and increased deprivation are more important risk factors for Covid-19 hospitalisation than pneumonia or influenza hospitalisation in type 2 diabetes

Across the three respiratory infections, we observed differential effects of ethnicity, male sex, and higher deprivation on hospitalisation risk. Compared to people of white ethnicity, black and south Asian groups had a higher risk of Covid-19 hospitalisation, but a lower risk of pneumonia hospitalisation (Figure 1). For influenza, a higher risk was seen in the south Asian group only. Male sex was more strongly associated with increased Covid-19 hospitalisation than pneumonia hospitalisation, and was associated with decreased hospitalisation for influenza (Figure 1). For all three infections higher deprivation was associated with a higher risk of hospitalisation, but there was a stronger association with Covid-19 than influenza or pneumonia (Figure 1). Older age was strongly associated with pneumonia hospitalisation, whereas a lesser age-associated gradients was seen for Covid-19 and influenza (Figure 1).

Risk factors associations are similar for mortality

2,635 of 584,854 (0.45%) died from Covid-19, and 12,652 of 585,289 (2.16%) died from pneumonia in the respective study periods (baseline characteristics: Supplemental table 6). Clinical and sociodemographic risk factor associations for Covid-19 and pneumonia mortality were largely consistent with those for hospitalisation (Supplemental figure 4).

Type 1 diabetes

Of people with type 1 diabetes, 209 of 43,033 (0.49%) were hospitalised with Covid-19 during the 2020 study period. 208 of 42,488 (0.49%) were hospitalised for influenza, and 1,401 of 42,488 (3.30%) were hospitalised with pneumonia during the 2016 to 2019 study period (baseline characteristics: Supplemental table 7). Similarly to type 2 diabetes, higher HbA1c and higher BMI were associated with increased hospitalisation for Covid-19, influenza, and pneumonia in type 1 diabetes (Supplemental figure 5, Supplemental figure 6A, Supplemental figure 6B). Sociodemographic associations were broadly similar to those

For peer review only

in type 2 diabetes, although the association between increasing age and pneumonia hospitalisation was not stronger than for Covid-19, and there was no clear difference in Covid-19 risk by deprivation quintile in type 1 diabetes. We observed an increased risk of Covid-19 hospitalisation in people of non-white ethnicities, and in people of south Asian ethnicity for influenza, but no difference in risk by ethnicity for pneumonia (Supplemental figure 5).

Sensitivity analyses

Results were largely consistent with the primary analyses when outcomes were restricted to primary hospitalisation diagnoses (type 1 diabetes: Supplemental table 8, Supplemental figure 7; type 2 diabetes: Supplemental table 9, Supplemental figure 8), and primary cause of death in type 2 diabetes (Supplemental table 10, Supplemental figure 9). When repeating the primary analysis in those vaccinated and unvaccinated, associations with pneumonia hospitalisation were consistent in those with and without a pneumococcal vaccination (Supplemental figure 10). There were very low numbers of people without an influenza vaccination, however, associations with influenza hospitalisation appeared to be consistent in those with and without vaccination for influenza (Supplemental figure 11).

For peer review only

Discussion

In a large-scale UK population-based analysis, we provide a comprehensive comparison of risk factors for Covid-19, influenza, and pneumonia in people with type 1 and type 2 diabetes. We show that although clinical risk factor associations for HbA1c, BMI, and microvascular complications are similar for all three respiratory infections, there is evidence that risk varies meaningfully by age and sex. Notably, whilst poor glycaemic control and marked obesity are consistently associated with an increased risk of hospitalisation and death across all three infections, in relative terms these risk increases are greater in those aged under 70. For BMI, the association between higher BMI (>30) and a severe outcome is consistently higher for females than males for all three infections.

We also demonstrate clear differences in sociodemographic risk factor associations by respiratory infection type. The previously reported higher risk of severe Covid-19 in people of non-white ethnicity [10, 17, 36] is not consistent with other respiratory infections and is the opposite pattern of association seen for pneumonia. Additionally, male sex and higher deprivation appear to be more important risk factors for Covid-19 than influenza and pneumonia. Findings have important implications for the management of modifiable infection risk factors in people with diabetes as well as current and future resource planning to manage severe respiratory infection.

Whilst Covid-19 risk factors in people with diabetes have previously been robustly described [16, 17] previous population-based studies have not compared risk factor associations with those seen for other major respiratory infections. To our knowledge, only one general population (with and without diabetes) study has compared a range of risk factors for Covid-19 to influenza and pneumonia. However, this study used a selected cohort (UK Biobank) and a positive test result/ incident infection as the outcome measure which means findings are likely to be influenced by UK testing policies. Due to the under-representation of non-white ethnicities in the UK Biobank, this study also had limited power to describe associations by ethnicity [31].

The associations between higher HbA1c and BMI and severe Covid-19 outcomes identified in this study are consistent with previous studies of people with diabetes [14, 16-19]. Whilst UK population-based data have suggested that associations between poor glycaemic control and obesity and Covid-19 mortality were greater in people younger than 70 [17], our study extends this previous work by evaluating non-linear continuous associations for HbA1c and BMI for severe Covid-19, and showing associations are not specific to Covid-19. For pneumonia, poor glycaemic control has been associated with increased hospitalisation [12] but risk heterogeneity by patient phenotype has not been previously evaluated. Our finding of a markedly increased risk of severe Covid-19, influenza, and pneumonia in females with higher BMI is supported by prospective cohort data on the whole UK Biobank population, including individuals with and without diabetes [29].

Notably, we show that previously described risk factor associations between male sex, older age, non-white ethnicity, increasing deprivation, and severe Covid-19 outcomes in people with diabetes [16, 17] are not generalisable to influenza and pneumonia. Previous studies describing associations between sex and pneumonia have not reported consistent effects, [37] and evidence is also lacking for risk factors associated with severe influenza with the exception of increasing age [38]. Previous studies have found evidence of increased risk of severe Covid-19 outcomes in those of non-white ethnicity [10, 17, 36] consistent with our

findings. Whilst these studies have mainly been based on UK data, ethnic disparities in rates of influenza-associated hospitalisation have been observed in US data, with certain ethnic groups having poorer outcomes compared to those of white ethnicity [26].

Strengths and Limitations

Strengths of our study include the large size of the dataset, representativeness to the UK population, and linkage to longitudinal hospital admission and mortality data allowing us to robustly define infection outcomes and systematically compare Covid-19, influenza, and pneumonia risk factor associations. The large dataset allowed us to study both type 1 diabetes and type 2 diabetes. As with all studies of this nature, misclassification or misdiagnosis of our outcomes is possible and relies on correct coding in the records. Evaluating both hospitalisation and mortality outcomes, and analysis to evaluate the sensitivity of results to our outcome definitions, mitigated against this limitation. Despite this, we were unable to completely differentiate between infection being the reason for admission or death or being contracted during admission, or only contributing to hospitalisation/death. We have also noted some differences in the coding of the studied respiratory infections in the hospital records; Covid-19 and influenza were mostly confirmed by a positive test whereas pneumonia coding was much broader and less specific, which should be considered when interpreting the results. The associations we have found do not have a causal interpretation and we cannot rule out potential confounding by unmeasured variables, which is an inherent limitation of many observational studies, however, we have adjusted for a wide range of covariates. There is also potential for misclassification of diabetes type in our cohorts, but we have used comprehensive algorithms considering prescriptions, age at diagnosis as well as diabetes codes, to define diabetes type as thoroughly as possible. This approach has been validated against a biological diabetes definition using classification biomarkers [35].

Due to our focus on severe infection outcomes, we will have missed milder community cases of infection; to assess risk factors for being infected would have required a different study design. The use of two different study periods for Covid-19 and influenza/ pneumonia, while unavoidable, has a risk of bias and may have influenced the observed associations. In particular, associations found for Covid-19 may reflect behaviours during the first wave of the Covid-19 pandemic, and be influenced by factors unique to this period including marked geographical differences in infection rates, as well interventions such as lockdowns and shielding. The disproportionate impact of these measures on non-white and more deprived groups [39] may have influenced the sociodemographic disparities we have observed. The risks associated with the disruption to routine care in people with diabetes during the Covid-19 pandemic [40] also makes comparisons with a pre-pandemic study period difficult. Despite these limitations, a key strength of our analysis is the use of standardised case and risk factor definitions, and consistent statistical methodology, ensuring associations observed across the two study periods were as comparable as possible. Using this approach, we demonstrate major clinical risk factors are very similar across infections, a finding that is unlikely to be explained by specific impacts of the Covid-19 pandemic.

Clinical implications

For peer review only

This study highlights the potential importance of good glycaemic control and supporting weight loss for managing risk of three major respiratory infections in people with diabetes. While BMI may be hard to modify at an individual level, a population-level investment in effective weight reduction measures may help reduce severe respiratory infection. The substantial differences in sociodemographic risk factor associations across the different respiratory infections suggest we cannot assume high-risk groups for one respiratory infection are the same as another. This means existing risk models for respiratory infection, such as those developed for Covid-19 [5, 41], may not be applicable to new respiratory infections and so in the event of a new respiratory pathogen, early analysis of risk factors would be needed to correctly target interventions. The finding that the increased risk of severe Covid-19 in those of non-white ethnicity is largely not seen for other respiratory infections (except for an increase in severe influenza risk in people of south Asian ethnicity) highlights the importance of further exploration as to why ethnicity-associated infection risk appears to vary by infection type.

Conclusion

Poor glycaemic control and obesity are associated with a consistently increased risk of severe outcomes from Covid-19, influenza, and pneumonia, especially in those aged under 70. In contrast, even in a single country setting, sociodemographic risk factor associations differ by respiratory infection. This highlights the need for the development of population-specific risk stratification approaches for individual respiratory infections to ensure accurate identification of people with diabetes at the highest risk of severe outcomes.

For peer review only

Acknowledgements

This article is based on data from the Clinical Practice Research Datalink obtained under licence from the UK Medicines and Healthcare products Regulatory Agency. CPRD data is provided by patients and collected by the NHS as part of their care and support. Approval for CPRD data access and the study protocol was granted by the CPRD Independent Scientific Advisory Committee (Project-ID: 20_000101).

This study was supported by the National Institute for Health and Care Research Exeter Biomedical Research Centre. The views expressed are those of the author(s) and not necessarily those of the NIHR or the Department of Health and Social Care or Diabetes UK. RH and KGY are supported by Research England's Expanding Excellence in England (E3) fund. NJT is funded by a Wellcome Trust–funded GW4 PhD fellowship. RC is funded through the AvonCAP study which is an investigator-led University of Bristol study funded by Pfizer and further supported by the National Institute for Health and Care Research Applied Research Collaboration South West Peninsula. BMS is supported by the NIHR Exeter Clinical Research Facility. JMD is supported by an Independent Fellowship funded by Research England's Expanding Excellence in England (E3) fund. The funders had no role in the study design, data collection, data analysis, data interpretation, writing of the article, or the decision to submit for publication.

Funding

This work was supported by Diabetes UK (20/0006220).

Conflict of Interest

BAM is an employee of Wellcome Trust; holds an honorary post at the Alan Turing Institute and University College London; and declares payments from the Medical Research Council, Health Data Research UK, British Heart Foundation, and Engineering and Physical Sciences Research Council (grant EP/N510129/). RC declares research funding from Pfizer. SJV declares support from the University of Warwick, University of Kaiserslautern, and German Research Center for Artificial Intelligence; consulting fees from PUMAS; stock from Freshflow; and grant funding from The Alan Turing Institute (EP/N510129), Engineering and Physical Sciences Research Council, and Massachusetts Institute of Technology. APM received research funding from Eli Lilly and Company, Pfizer, and AstraZeneca. For all authors these are outside the submitted work; there are no other relationships or activities that could appear to have influenced the submitted work.

Author Contributions and Guarantor Statement

The study concept and design were conceived and developed by RH, JMD, APM, BAM, and NJT. JMD, KGY, RH, and BMS had access to all the raw data sets used for the study. RH, KGY, DR, and RC prepared the data for analysis. RH undertook the analysis, with support from JMD, BMS, JG, and APM. All authors provided support for the interpretation of results, critically revised the manuscript, and saw and approved the final article. JMD and APM attest that all listed authors meet authorship criteria, that no others meeting the criteria have been

For peer review only

omitted. JMD and APM were responsible for the decision to submit for publication, and are the guarantors of this work and, as such, had full access to all the data in the study and takes responsibility for the integrity of the data and the accuracy of the data analysis.

Data availability

No additional data are available from the authors although CPRD data are available by application to CPRD Independent Scientific Advisory Committee.

Prior presentation

Oral presentation of preliminary results from this study have been given at the Diabetes UK Professional Conference 2022 and the European Association for the Study of Diabetes (EASD) Annual Meeting 2022.

For peer review only

References

1. Carey IM, Critchley JA, DeWilde S, et al. Risk of Infection in Type 1 and Type 2 Diabetes Compared With the General Population: A Matched Cohort Study. Diabetes Care 2018;41(3):513-21. doi: 10.2337/dc17-2131

2. Pearson-Stuttard J, Cheng YJ, Bennett J, et al. Trends in leading causes of hospitalisation of adults with diabetes in England from 2003 to 2018: an epidemiological analysis of linked primary care records. Lancet Diabetes Endocrinol 2022;10(1):46-57. doi: 10.1016/S2213-8587(21)00288-6

3. Grasselli G, Greco M, Zanella A, et al. Risk Factors Associated With Mortality Among Patients With COVID-19 in Intensive Care Units in Lombardy, Italy. JAMA Intern Med 2020;180(10):1345-55. doi: 10.1001/jamainternmed.2020.3539

4. Zhou F, Yu T, Du R, et al. Clinical course and risk factors for mortality of adult inpatients with COVID-19 in Wuhan, China: a retrospective cohort study. Lancet 2020;395(10229):1054-62. doi: 10.1016/S0140-6736(20)30566-3

5. Clift AK, Coupland CAC, Keogh RH, et al. Living risk prediction algorithm (QCOVID) for risk of hospital admission and mortality from coronavirus 19 in adults: national derivation and validation cohort study. BMJ 2020;371:m3731. doi: 10.1136/bmj.m3731

6. Roncon L, Zuin M, Rigatelli G, et al. Diabetic patients with COVID-19 infection are at higher risk of ICU admission and poor short-term outcome. J Clin Virol 2020;127:104354. doi: 10.1016/j.jcv.2020.104354

7. Richardson S, Hirsch JS, Narasimhan M, et al. Presenting Characteristics, Comorbidities, and Outcomes Among 5700 Patients Hospitalized With COVID-19 in the New York City Area. JAMA 2020;323(20):2052-59. doi: 10.1001/jama.2020.6775

8. Docherty AB, Harrison EM, Green CA, et al. Features of 20 133 UK patients in hospital with covid-19 using the ISARIC WHO Clinical Characterisation Protocol: prospective observational cohort study. BMJ 2020;369:m1985. doi: 10.1136/bmj.m1985

9. Dennis JM, Mateen BA, Sonabend R, et al. Type 2 Diabetes and COVID-19-Related Mortality in the Critical Care Setting: A National Cohort Study in England, March-July 2020. Diabetes Care 2021;44(1):50-57. doi: 10.2337/dc20-1444

10. Williamson EJ, Walker AJ, Bhaskaran K, et al. Factors associated with COVID-19-related death using OpenSAFELY. Nature 2020;584(7821):430-36. doi: 10.1038/s41586-020-2521-4

11. Barron E, Bakhai C, Kar P, et al. Associations of type 1 and type 2 diabetes with COVID-19-related mortality in England: a whole-population study. Lancet Diabetes Endocrinol 2020;8(10):813-22. doi: 10.1016/S2213-8587(20)30272-2

12. Kornum JB, Thomsen RW, Riis A, et al. Diabetes, glycemic control, and risk of hospitalization with pneumonia: a population-based case-control study. Diabetes Care 2008;31(8):1541-5. doi: 10.2337/dc08-0138

13. Owusu D, Rolfes MA, Arriola CS, et al. Rates of Severe Influenza-Associated Outcomes Among Older Adults Living With Diabetes-Influenza Hospitalization Surveillance Network

For peer review only

(FluSurv-NET), 2012-2017. Open Forum Infect Dis 2022;9(5):ofac131. doi: 10.1093/ofid/ofac131

14. Cariou B, Hadjadj S, Wargny M, et al. Phenotypic characteristics and prognosis of inpatients with COVID-19 and diabetes: the CORONADO study. Diabetologia 2020;63(8):1500-15. doi: 10.1007/s00125-020-05180-x

15. Shi Q, Zhang X, Jiang F, et al. Clinical Characteristics and Risk Factors for Mortality of COVID-19 Patients With Diabetes in Wuhan, China: A Two-Center, Retrospective Study. Diabetes Care 2020;43(7):1382-91. doi: 10.2337/dc20-0598

16. McGurnaghan SJ, Weir A, Bishop J, et al. Risks of and risk factors for COVID-19 disease in people with diabetes: a cohort study of the total population of Scotland. Lancet Diabetes Endocrinol 2021;9(2):82-93. doi: 10.1016/S2213-8587(20)30405-8

17. Holman N, Knighton P, Kar P, et al. Risk factors for COVID-19-related mortality in people with type 1 and type 2 diabetes in England: a population-based cohort study. Lancet Diabetes Endocrinol 2020;8(10):823-33. doi: 10.1016/S2213-8587(20)30271-0

18. Hamer M, Gale CR, Batty GD. Diabetes, glycaemic control, and risk of COVID-19 hospitalisation: Population-based, prospective cohort study. Metabolism 2020;112:154344. doi: 10.1016/j.metabol.2020.154344

19. Zhu L, She ZG, Cheng X, et al. Association of Blood Glucose Control and Outcomes in Patients with COVID-19 and Pre-existing Type 2 Diabetes. Cell Metab 2020;31(6):1068-77 e3. doi: 10.1016/j.cmet.2020.04.021

20. Jordan R, Verlander N, Olowokure B, et al. Age, sex, material deprivation and respiratory mortality. Respir Med 2006;100(7):1282-5. doi: 10.1016/j.rmed.2005.10.014

21. Thomsen RW, Riis A, Norgaard M, et al. Rising incidence and persistently high mortality of hospitalized pneumonia: a 10-year population-based study in Denmark. J Intern Med 2006;259(4):410-7. doi: 10.1111/j.1365-2796.2006.01629.x

22. Myles PR, Hubbard RB, Gibson JE, et al. Pneumonia mortality in a UK general practice population cohort. Eur J Public Health 2009;19(5):521-6. doi: 10.1093/eurpub/ckp081

23. Millett ER, De Stavola BL, Quint JK, et al. Risk factors for hospital admission in the 28 days following a community-acquired pneumonia diagnosis in older adults, and their contribution to increasing hospitalisation rates over time: a cohort study. BMJ Open 2015;5(12):e008737. doi: 10.1136/bmjopen-2015-008737

24. Lopez-de-Andres A, Jimenez-Garcia R, Hernandez-Barrera V, et al. Sex-Related Disparities in the Incidence and Outcomes of Community-Acquired Pneumonia among Type 2 Diabetes Patients: A Propensity Score-Matching Analysis Using the Spanish National Hospital Discharge Database for the Period 2016-2019. J Clin Med 2021;10(17) doi: 10.3390/jcm10173975

25. Hungerford D, Ibarz-Pavon A, Cleary P, et al. Influenza-associated hospitalisation, vaccine uptake and socioeconomic deprivation in an English city region: an ecological study. BMJ Open 2018;8(12):e023275. doi: 10.1136/bmjopen-2018-023275

For peer review only

26. O'Halloran AC, Holstein R, Cummings C, et al. Rates of Influenza-Associated Hospitalization, Intensive Care Unit Admission, and In-Hospital Death by Race and Ethnicity in the United States From 2009 to 2019. JAMA Netw Open 2021;4(8):e2121880. doi: 10.1001/jamanetworkopen.2021.21880

27. Breitling LP. Evidence of non-linearity in the association of glycemic control with influenza/pneumonia mortality: a study of 19 000 adults from the US general population. Diabetes Metab Res Rev 2016;32(1):111-20. doi: 10.1002/dmrr.2681

28. Kornum JB, Norgaard M, Dethlefsen C, et al. Obesity and risk of subsequent hospitalisation with pneumonia. Eur Respir J 2010;36(6):1330-6. doi: 10.1183/09031936.00184209

29. Peters SAE, MacMahon S, Woodward M. Obesity as a risk factor for COVID-19 mortality in women and men in the UK biobank: Comparisons with influenza/pneumonia and coronary heart disease. Diabetes Obes Metab 2021;23(1):258-62. doi: 10.1111/dom.14199

30. de Jong M, Woodward M, Peters SAE. Diabetes and COVID-19-Related Mortality in Women and Men in the UK Biobank: Comparisons With Influenza/Pneumonia and Coronary Heart Disease. Diabetes Care 2021;44(2):e22-e24. doi: 10.2337/dc20-2378

31. Ho FK, Celis-Morales CA, Gray SR, et al. Modifiable and non-modifiable risk factors for COVID-19, and comparison to risk factors for influenza and pneumonia: results from a UK Biobank prospective cohort study. BMJ Open 2020;10(11):e040402. doi: 10.1136/bmjopen-2020-040402

32. Health Data Research UK. HDR UK and NIHR issue a rapid funding opportunity for Data Science to inform NHS compound winter pressure policy response. 2022. Available: https://www.hdruk.ac.uk/funding-opportunities/rapid-funding-winter-pressure/ [Accessed 4 January 2023]

33. Wolf A, Dedman D, Campbell J, et al. Data resource profile: Clinical Practice Research Datalink (CPRD) Aurum. Int J Epidemiol 2019;48(6):1740-40g. doi: 10.1093/ije/dyz034

34. Clinical Practice Research Datalink. CPRD Data. Available: https://cprd.com/data [Accessed 11 April 2023]

35. Thomas NJ, McGovern A, Young KG, et al. Identifying type 1 and 2 diabetes in research datasets where classification biomarkers are unavailable: assessing the accuracy of published approaches. J Clin Epidemiol 2022;153:34-44. doi: 10.1016/j.jclinepi.2022.10.022

36. Mathur R, Rentsch CT, Morton CE, et al. Ethnic differences in SARS-CoV-2 infection and COVID-19-related hospitalisation, intensive care unit admission, and death in 17 million adults in England: an observational cohort study using the OpenSAFELY platform. Lancet 2021;397(10286):1711-24. doi: 10.1016/S0140-6736(21)00634-6

37. Almirall J, Serra-Prat M, Bolibar I, et al. Risk Factors for Community-Acquired Pneumonia in Adults: A Systematic Review of Observational Studies. Respiration 2017;94(3):299-311. doi: 10.1159/000479089

38. Mertz D, Kim TH, Johnstone J, et al. Populations at risk for severe or complicated influenza illness: systematic review and meta-analysis. BMJ 2013;347:f5061. doi: 10.1136/bmj.f5061

For peer review only

39. Khunti K, Feldman EL, Laiteerapong N, et al. The Impact of the COVID-19 Pandemic on Ethnic Minority Groups With Diabetes. Diabetes Care 2022 doi: 10.2337/dc21-2495

40. Valabhji J, Barron E, Gorton T, et al. Associations between reductions in routine care delivery and non-COVID-19-related mortality in people with diabetes in England during the COVID-19 pandemic: a population-based parallel cohort study. Lancet Diabetes Endocrinol 2022;10(8):561-70. doi: 10.1016/S2213-8587(22)00131-0

41. Wynants L, Van Calster B, Collins GS, et al. Prediction models for diagnosis and prognosis of covid-19: systematic review and critical appraisal. BMJ 2020;369:m1328. doi: 10.1136/bmj.m1328

For peer review only

Figure legends

Figure 1: Hazard ratios and 95% confidence intervals for the association of clinical and sociodemographic risk factors with hospitalisation for Covid-19 (orange squares), influenza (green circles), and pneumonia (blue diamonds) in type 2 diabetes.

Figure 2: Hazard ratios and 95% confidence intervals for the association of continuous HbA1c with hospitalisation for Covid-19, influenza, and pneumonia in type 2 diabetes. Overall, and by age and sex. Statistical significance of age subgroup interactions with HbA1c: p=0.0190 for Covid-19, p=0.2214 for influenza, and p<.0001 for pneumonia. Statistical significance of sex subgroup interactions with HbA1c: p=0.5488 for Covid-19, p=0.0737 for influenza, and p=0.0037 for pneumonia. Density plots show the distribution of HbA1c in each group.

Figure 3: Hazard ratios and 95% confidence intervals for the association of continuous BMI with hospitalisation for Covid-19, influenza, and pneumonia in type 2 diabetes. Overall, and by age and sex. Statistical significance of age subgroup interactions with BMI: p=0.0046 for Covid-19, p=0.0934 for influenza, and p<.0001 for pneumonia. Statistical significance of sex subgroup interactions with BMI: p=0.2780 for Covid-19, p=0.0008 for influenza, and p=<.0001 for pneumonia. Density plots show the distribution of BMI in each group.

For peer review only

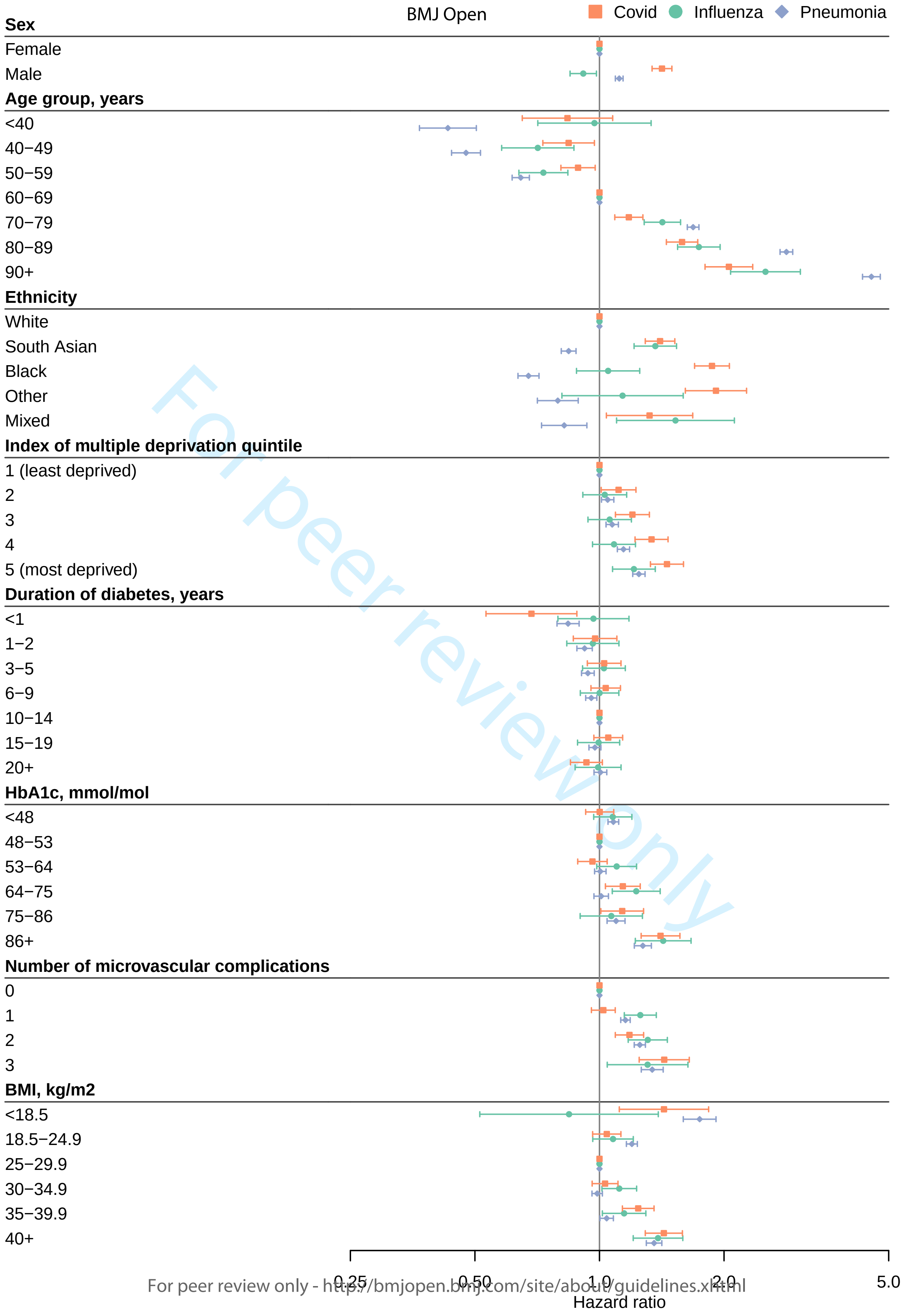

For peer review only

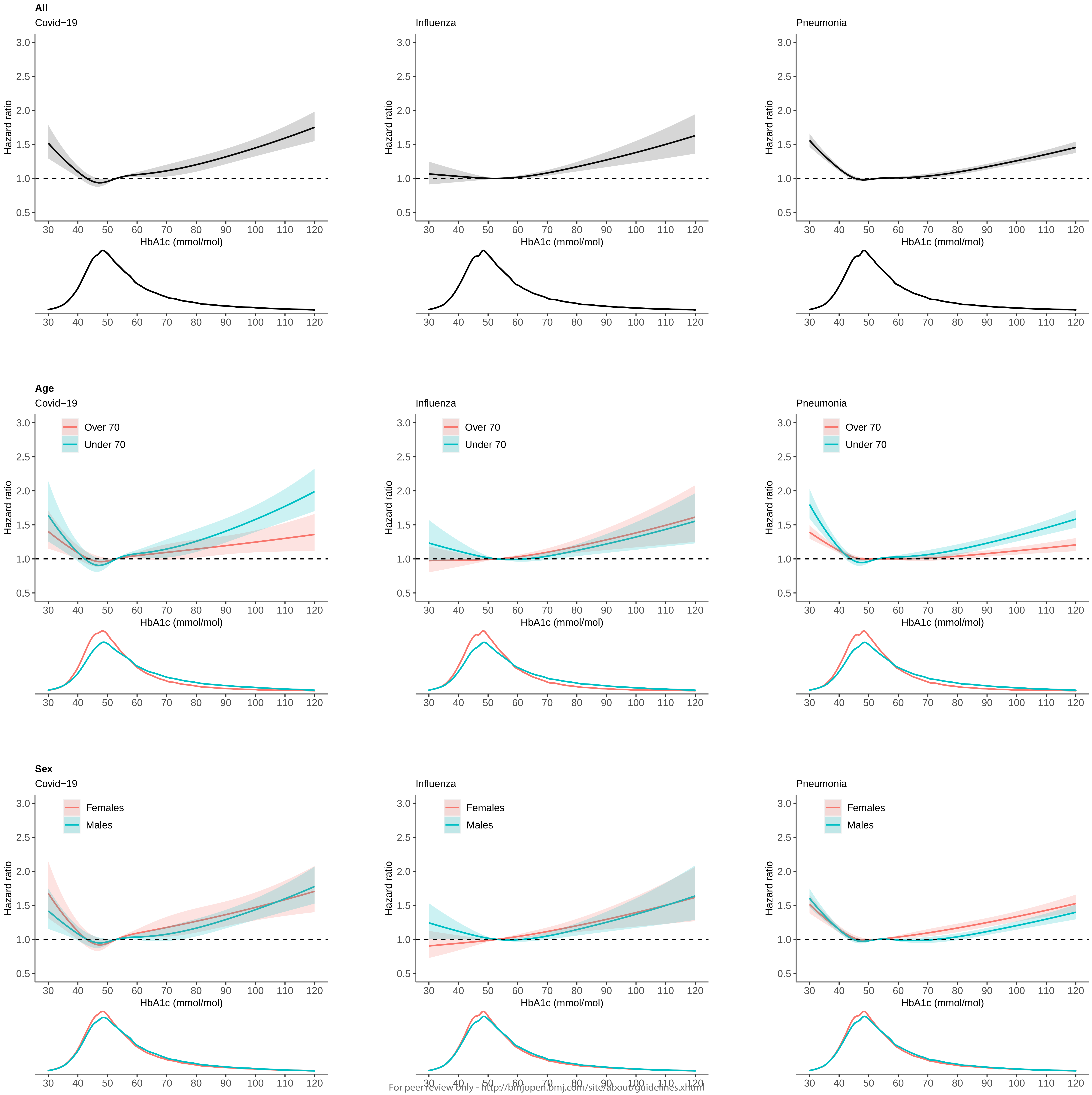
All
Covid−19
Influenza
Pneumonia
Hazard ratio
HbA1c (mmol/mol)
Age
Over 70
Under 70
Sex
Females
Males

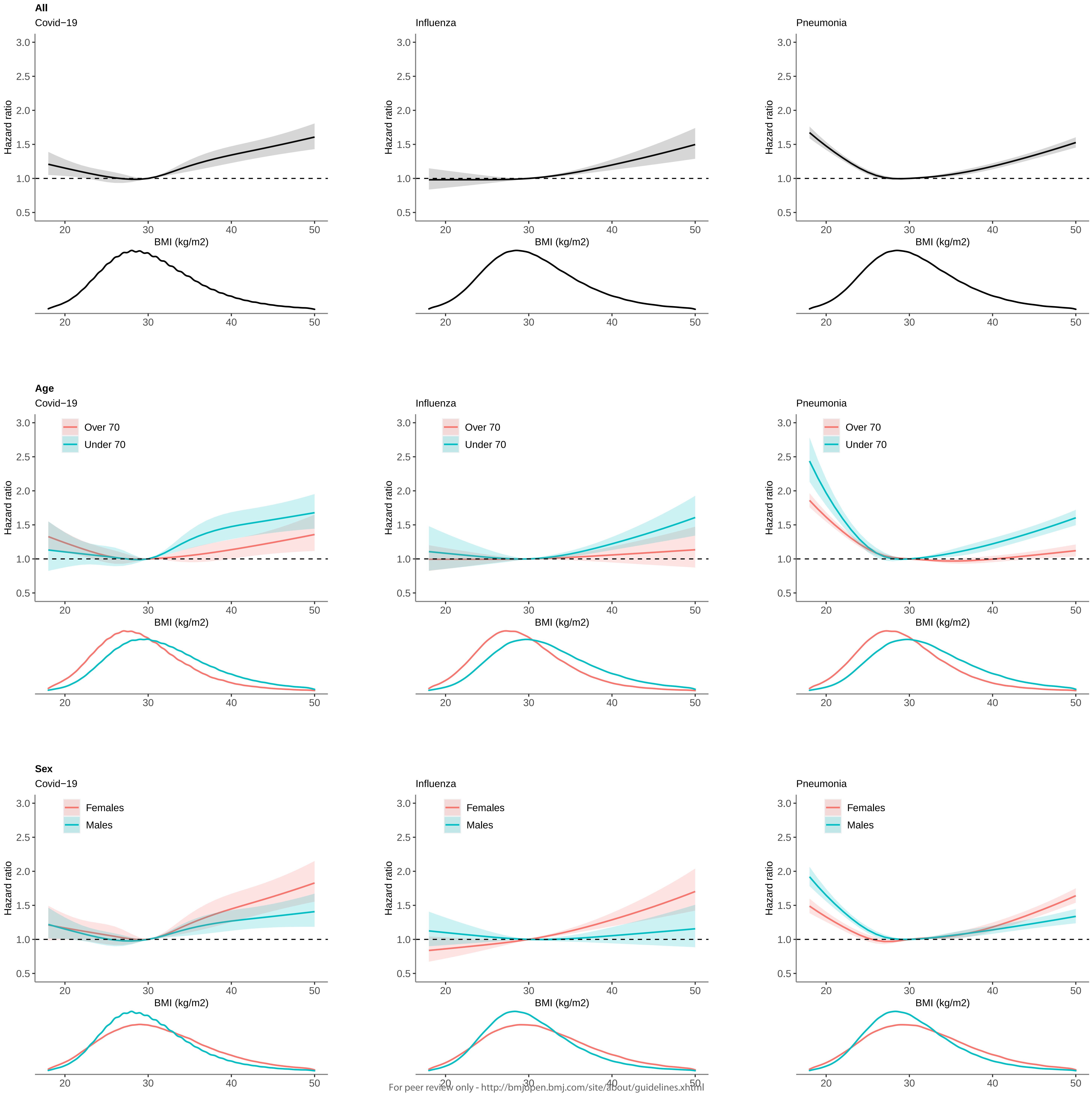
All
Covid−19
Influenza
Pneumonia
Hazard ratio
3.0
2.5
2.0
1.5
1.0
0.5
20
30
40
50
BMI (kg/m2)
Age
Over 70
Under 70
Sex
Females
Males

Supplemental material

Table of Contents

**Page**	
**2**	Supplemental figure 1. Flow diagram of study participants and exclusions.
**3**	Supplemental table 1. ICD-10 codes used to define Covid-19, influenza, and pneumonia outcomes.
**4**	Supplemental table 2. Definitions of exposure variables.
	**Type 2 diabetes (hospitalisation)**
**5**	Supplemental table 3. Frequency of ICD-10 codes recorded in hospitalisation outcomes in type 2 diabetes for Covid-19, influenza, and pneumonia.
**6-7**	Supplemental table 4. Full baseline characteristics and Covid-19, influenza, and pneumonia hospitalisations in type 2 diabetes.
**8**	Supplemental figure 2. Association of potential risk factors with hospitalisation for Covid-19, influenza, and pneumonia in type 2 diabetes.
**9-10**	Supplemental figure 5. Hazard ratio values for the association of potential risk factors with hospitalisation for Covid-19, influenza, and pneumonia in type 2 diabetes.
**11**	Supplemental figure 3A. Association of continuous HbA1c with hospitalisation for Covid-19, influenza, and pneumonia in type 2 diabetes by ethnicity subgroup.
**11**	Supplemental figure 3B. Association of continuous BMI with hospitalisation for Covid-19, influenza, and pneumonia in type 2 diabetes by ethnicity subgroup.
	**Type 2 diabetes (mortality)**
**12-13**	Supplemental table 6. Baseline characteristics and Covid-19 and pneumonia deaths in type 2 diabetes.
**14**	Supplemental figure 4. Association of potential risk factors with Covid-19, and pneumonia mortality in type 2 diabetes.
	**Type 1 diabetes**
**15**	Supplemental table 7. Baseline characteristics and Covid-19, influenza, and pneumonia hospitalisations in type 1 diabetes.
**16**	Supplemental figure 5. Association of potential risk factors with hospitalisation for Covid-19, influenza, and pneumonia in type 1 diabetes.
**17**	Supplemental figure 6A. Association of continuous HbA1c with hospitalisation for Covid-19, influenza, and pneumonia in type 1 diabetes.
**17**	Supplemental figure 6B. Association of continuous BMI with hospitalisation for Covid-19, influenza, and pneumonia in type 1 diabetes.
	**Sensitivity analyses**
**18**	Supplemental table 8. Baseline characteristics and Covid-19, influenza, and pneumonia hospitalisations in type 1 diabetes, restricting hospitalisation definition to primary diagnosis in HES.
**19**	Supplemental figure 7. Association of potential risk factors with hospitalisation for Covid-19, influenza, and pneumonia in type 1 diabetes, restricting hospitalisation definition to primary diagnosis in HES.
**20-21**	Supplemental table 9. Baseline characteristics and Covid-19, influenza, and pneumonia hospitalisations in type 2 diabetes, restricting hospitalisation definition to primary diagnosis in HES.
**22**	Supplemental figure 8. Association of potential risk factors with hospitalisation for Covid-19, influenza, and pneumonia in type 2 diabetes, restricting hospitalisation definition to primary diagnosis in HES.
**23-24**	Supplemental table 10. Baseline characteristics and Covid-19 and pneumonia deaths in type 2 diabetes, restricting mortality definition to primary cause in ONS.
**25**	Supplemental figure 9. Association of potential risk factors with Covid-19, and pneumonia mortality in type 2 diabetes, restricting mortality definition to primary cause in ONS.
**26**	Supplemental figure 10. Association of potential risk factors with pneumonia hospitalisation in those with a pneumococcal vaccination and those without.
**27**	Supplemental figure 11. Association of potential risk factors with influenza hospitalisation in those with an influenza vaccination and those without.
**28**	References

For peer review only

Supplemental figure 1. Flow diagram of study participants and exclusions.

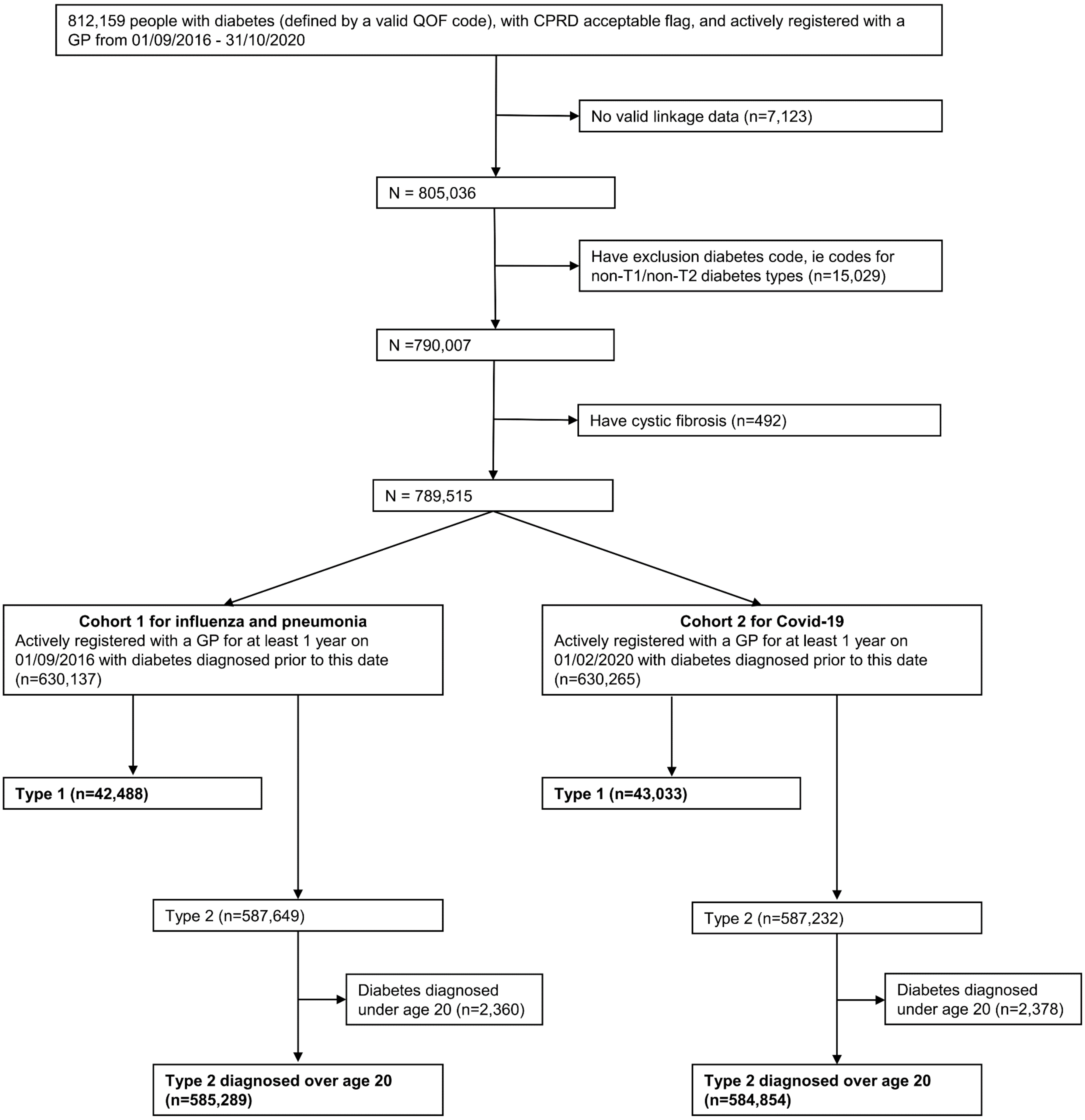

For peer review only - http://bmjopen.bmj.com/site/about/guidelines.xhtml

Supplemental table 1. ICD-10 codes used to define Covid-19, influenza, and pneumonia hospitalisation and mortality outcomes.

**Infection**	**ICD-10 codes**
Covid-19	U07.1, U07.2
Influenza	J09-J11
Pneumonia	J12-J18

For peer review only

Supplemental table 2. Definitions of exposure variables.

Variable	Definition	Data source	Categories	Missing data method
Sex	Sex recorded in CPRD	Primary care	Male, or female	No missing data
Age	Age at baseline date, calculated from date of birth	Primary care	<40, 40-49, 50-59, 60-69, 70-79, 80-89 or 90+ years for type 2 diabetes cohorts, and <18, 18-39, 40-49, 50-59, 60-69, 70-79 or 80+ years for type 1 diabetes cohorts	No missing data
Ethnicity	Major UK ethnic group based on a previous study [1]	Primary care and HES	White, south Asian, black, mixed, other, or unknown	Missing indicator method
Deprivation	Index of Multiple Deprivation (official national measure) [2]	Linked Index of Multiple Deprivation dataset	1, 2, 3, 4, 5, or missing (Quintile of national distribution)	Missing indicator method
Smoking status	Smoking status at baseline date	Primary care	Non-smoker, active smoker, ex-smoker, or unknown	Missing indicator method
Region	UK strategic health region based on GP practice location	Primary care	East Midlands, East of England, London, North East, North West, South Central, South East Coast, South West, West Midlands, Yorkshire and the Humber, or missing	Missing indicator method
HbA1c	Latest recorded HbA1c measurement in the last two years prior to baseline date	Primary care	<48 mmol/ mol (<6.5%), 48-53 mmol/mol (6.5-7%), 53-64 mmol/mol (7-8%), 64-75 mmol/mol (8-9%), 75-86 mmol/mol (9-10%), >86 mmol/mol (>10%), or missing	Missing indicator method
BMI	Latest recorded BMI measurement in the last two years prior to baseline date, grouped using the World Health Organisation classification [3]	Primary care	<18.5 kg/m², 18.5-24.9 kg/m², 25-29.9 kg/m², 30-34.5 kg/m², 35-39.9 kg/m², 40+ kg/m², or missing	Missing indicator method
Diabetes duration	Years since diabetes diagnosis, with diagnosis date defined using an algorithm adapted from a previous study [4] taking the earliest date of first diabetes clinical code, first HbA1c measurement of 48 mmol/mol (6.5%) or above, first oral hypoglycaemic agent (OHA) prescription or first insulin prescription	Primary care	<1 year, 1-2 years, 3-5 years, 6-9 years, 10-14 years, 15-19 years or 20+ years for the type 2 diabetes cohorts, and <5 years, 5-9 years, 10-14 years, 15-19 years, 20-24 years, 25-29 years or 30+ years for type 1 diabetes cohorts	No missing data
Microvascular complications	Total number of coded microvascular complications (diabetic nephropathy, neuropathy, and retinopathy) recorded prior to baseline date	Primary care and HES	0, 1, 2, or 3 complications	Non by definition
Diabetes treatment	Diabetes treatment recorded within last 6 months prior to baseline date	Primary care	Insulin prescription, oral hypoglycaemic agent (OHA) prescriptions only, or no treatment	Non by definition
Comorbidities	Record of comorbidity with diagnosis date prior to baseline date. Comorbidities studied covered cardiovascular (hypertension, atrial fibrillation, angina, previous myocardial infarction, previous cardiac revascularisation, other ischaemic heart disease, heart failure, peripheral vascular disease), respiratory (asthma, chronic obstructive pulmonary disease), neurological (previous transient ischaemic attack, previous stroke, dementia, or other neurological condition), oncological (haematological cancer, solid cancer), and other conditions (chronic liver disease, solid organ transplant)	Primary care and HES	Yes, or no	Non by definition
Chronic kidney disease stage	Stage defined from estimated glomerular filtration rate (eGFR), stage 5 also ascertained by coded record prior to baseline [5]	Primary care and HES	Stage 1, stage 2, stage 3a, stage 3b, stage 4, stage 5, or missing	Missing indicator method
Albumin creatinine ratio	Defined from latest albumin creatinine ratio measurement prior to baseline	Primary care	A1, A2, A3, or missing	Missing indicator method
Recent hospitalisation for respiratory infection	A hospitalisation recorded with a code for respiratory infection in the 2 years prior to baseline	HES	Yes, or no	Non by definition
Recent hospitalisation for other causes	A hospitalisation recorded for any other cause (excluding respiratory infection) in the 2 years prior to baseline	HES	Yes, or no	Non by definition
Additional respiratory infections	Respiratory conditions previously included in the 2020 QCOVID risk prediction algorithm [6] (pulmonary fibrosis, pulmonary hypertension, bronchiectasis) with diagnosis occurring before the baseline date	Primary care and HES	Yes, or no	Non by definition
Other medications	Medications previously included in 2020 QCOVID risk prediction algorithm [6] (immunosuppressants, leukotrienes, long acting β agonists, oral steroids) with a prescription issued within 6 months prior to baseline	Primary care	Yes, or no	Non by definition

For peer review only

Supplemental table 3. Frequency of ICD-10 codes recorded in hospitalisation outcomes in type 2 diabetes for Covid-19 (2020), influenza (2016-2019), and pneumonia (2016-2019).

**Covid-19**		
**ICD10 code**	**Description**	**Frequency**
U07.1	COVID-19, confirmed by lab testing	4972
U07.2	COVID-19, not confirmed by lab testing (includes clinically-epidemiologically diagnosed, probable and suspected)	1073
**Influenza**		
**ICD10 code**	**Description**	**Frequency**
J10.1	Influenza with other respiratory manifestations, seasonal influenza virus identified	1645
J10.0	Influenza with pneumonia, seasonal influenza virus identified	1158
J11.1	Influenza with other respiratory manifestations, virus not identified	250
J10.8	Influenza with other manifestations, seasonal influenza virus identified	134
J11.0	Influenza with pneumonia, virus not identified	101
J09	Influenza due to identified zoonotic or pandemic influenza virus	11
J11.8	Influenza with other manifestations, virus not identified	10
**Pneumonia**		
**ICD10 code**	**Description**	**Frequency**
J18.1	Lobar pneumonia, unspecified	20367
J18.9	Pneumonia, unspecified	16382
J18.0	Bronchopneumonia, unspecified	885
J13	Pneumonia due to Streptococcus pneumoniae	407
J14	Pneumonia due to Haemophilus influenzae	138
J15.0	Pneumonia due to Klebsiella pneumoniae	137
J15.1	Pneumonia due to Pseudomonas	116
J15.2	Pneumonia due to staphylococcus	88
J15.4	Pneumonia due to other streptococci	73
J12.1	Respiratory syncytial virus pneumonia	71
J15.6	Pneumonia due to other Gram-negative bacteria	53
J15.9	Bacterial pneumonia, unspecified	53
J12.3	Human metapneumovirus pneumonia	46
J15.5	Pneumonia due to Escherichia coli	43
J17.2	Pneumonia in mycoses	41
J12.2	Parainfluenza virus pneumonia	35
J12.9	Viral pneumonia, unspecified	34
J17.3	Pneumonia in parasitic diseases	34
J18.8	Other pneumonia, organism unspecified	29
J15.8	Other bacterial pneumonia	24
J18.2	Hypostatic pneumonia, unspecified	22
J12.8	Other viral pneumonia	21
J15.3	Pneumonia due to streptococcus, group B	14
J15.7	Pneumonia due to Mycoplasma pneumoniae	14
J17.1	Pneumonia in viral diseases classified elsewhere	9
J12.0	Adenoviral pneumonia	6
J17.0	Pneumonia in bacterial diseases classified elsewhere	2
J16.0	Chlamydial pneumonia	1
J16.8	Pneumonia due to other specified infectious organisms	1

For peer review only

Supplemental table 4. Full baseline characteristics and Covid-19 (2020), influenza (2016-2019), and pneumonia (2016-2019) hospitalisations in type 2 diabetes.

	2020 cohort	Covid-19 hospitalisations (in 2020 cohort)	2016 cohort	Influenza hospitalisations (in 2016 cohort)	Pneumonia hospitalisations (in 2016 cohort)
**Number of individuals**	584854	5965	585289	3226	38088
**Mean (SD) follow-up, days**		265.0 (38.3)		915.2 (224.8)	897.4 (246.3)
**Sex**					
Female	253360 (43.3)	2317 (38.8)	256656 (43.9)	1581 (49.0)	17140 (45.0)
Male	331494 (56.7)	3648 (61.2)	328633 (56.1)	1645 (51.0)	20948 (55.0)
**Age group, years**					
<40	12898 (2.2)	70 (1.2)	13468 (2.3)	46 (1.4)	164 (0.4)
40-49	46075 (7.9)	258 (4.3)	49649 (8.5)	128 (4.0)	717 (1.9)
50-59	114537 (19.6)	729 (12.2)	113285 (19.4)	329 (10.2)	2495 (6.6)
60-69	151493 (25.9)	1198 (20.1)	153782 (26.3)	679 (21.0)	6068 (15.9)
70-79	155424 (26.6)	1660 (27.8)	152401 (26.0)	1078 (33.4)	12073 (31.7)
80-89	89871 (15.4)	1671 (28.0)	88492 (15.1)	811 (25.1)	13324 (35.0)
90+	14556 (2.5)	379 (6.4)	14212 (2.4)	155 (4.8)	3247 (8.5)
**Ethnicity**					
White	445160 (76.1)	4200 (70.4)	457714 (78.2)	2586 (80.2)	33206 (87.2)
South Asian	77253 (13.2)	891 (14.9)	71050 (12.1)	403 (12.5)	2935 (7.7)
Black	35144 (6.0)	645 (10.8)	33175 (5.7)	156 (4.8)	1324 (3.5)
Other	9886 (1.7)	144 (2.4)	8178 (1.4)	35 (1.1)	306 (0.8)
Mixed	6267 (1.1)	69 (1.2)	5654 (1.0)	37 (1.1)	246 (0.6)
Unknown	11144 (1.9)	16 (0.3)	9518 (1.6)	9 (0.3)	71 (0.2)
**Index of multiple deprivation quintile**					
1 (least deprived)	102950 (17.6)	761 (12.8)	103184 (17.6)	502 (15.6)	6296 (16.5)
2	106968 (18.3)	921 (15.4)	109006 (18.6)	548 (17.0)	6923 (18.2)
3	113454 (19.4)	1088 (18.2)	113661 (19.4)	601 (18.6)	7264 (19.1)
4	127048 (21.7)	1446 (24.2)	125492 (21.4)	698 (21.6)	8199 (21.5)
5 (most deprived)	134146 (22.9)	1747 (29.3)	133563 (22.8)	873 (27.1)	9380 (24.6)
Missing	288 (0.0)	2 (0.0)	383 (0.1)	4 (0.1)	26 (0.1)
**Duration of diagnosed diabetes, years**					
<1	14311 (2.4)	67 (1.1)	36503 (6.2)	146 (4.5)	1401 (3.7)
1-2	60219 (10.3)	397 (6.7)	77510 (13.2)	306 (9.5)	3324 (8.7)
3-5	105094 (18.0)	796 (13.3)	111199 (19.0)	491 (15.2)	5218 (13.7)
6-9	128817 (22.0)	1116 (18.7)	125083 (21.4)	621 (19.2)	7088 (18.6)
10-14	124471 (21.3)	1250 (21.0)	126421 (21.6)	771 (23.9)	9620 (25.3)
15-19	89704 (15.3)	1225 (20.5)	62373 (10.7)	465 (14.4)	5764 (15.1)
20+	62238 (10.6)	1114 (18.7)	46200 (7.9)	426 (13.2)	5673 (14.9)
**HbA1c, mmol/mol**					
<48	167739 (28.7)	1679 (28.1)	175659 (30.0)	934 (29.0)	12271 (32.2)
48-53	116015 (19.8)	1054 (17.7)	116131 (19.8)	557 (17.3)	7015 (18.4)
53-64	147209 (25.2)	1352 (22.7)	142662 (24.4)	791 (24.5)	8912 (23.4)
64-75	66071 (11.3)	767 (12.9)	62291 (10.6)	412 (12.8)	4062 (10.7)
75-86	33970 (5.8)	408 (6.8)	32257 (5.5)	184 (5.7)	2191 (5.8)
86+	39727 (6.8)	580 (9.7)	37470 (6.4)	270 (8.4)	2646 (6.9)
Missing	14123 (2.4)	125 (2.1)	18819 (3.2)	78 (2.4)	991 (2.6)
**Number of microvascular complications**					
0	264011 (45.1)	1889 (31.7)	281811 (48.1)	1077 (33.4)	11930 (31.3)
1	216115 (37.0)	2140 (35.9)	204550 (34.9)	1256 (38.9)	14444 (37.9)
2	96948 (16.6)	1621 (27.2)	91671 (15.7)	789 (24.5)	10288 (27.0)
3	7780 (1.3)	315 (5.3)	7257 (1.2)	104 (3.2)	1426 (3.7)
**BMI, kg/m2**					
<18.5	3026 (0.5)	66 (1.1)	2801 (0.5)	16 (0.5)	497 (1.3)
18.5-24.9	86199 (14.7)	1009 (16.9)	79018 (13.5)	476 (14.8)	6985 (18.3)
25-29.9	184626 (31.6)	1747 (29.3)	178501 (30.5)	885 (27.4)	10950 (28.7)
30-34.9	151732 (25.9)	1350 (22.6)	151161 (25.8)	809 (25.1)	8249 (21.7)
35-39.9	74766 (12.8)	752 (12.6)	75339 (12.9)	405 (12.6)	3976 (10.4)
40+	48455 (8.3)	517 (8.7)	48685 (8.3)	302 (9.4)	2877 (7.6)
Missing	36050 (6.2)	524 (8.8)	49784 (8.5)	333 (10.3)	4554 (12.0)
**Smoking status**					
Active smoker	87220 (14.9)	556 (9.3)	90218 (15.4)	548 (17.0)	6259 (16.4)
Ex-smoker	336719 (57.6)	3847 (64.5)	316723 (54.1)	1852 (57.4)	22587 (59.3)
Non-smoker	138840 (23.7)	1288 (21.6)	149524 (25.5)	651 (20.2)	7305 (19.2)
Unknown	22075 (3.8)	274 (4.6)	28824 (4.9)	175 (5.4)	1937 (5.1)
**Comorbidities**					
**Cardiovascular**					
Hypertension	425278 (72.7)	5055 (84.7)	419645 (71.7)	2692 (83.4)	33013 (86.7)
Atrial fibrillation	70633 (12.1)	1413 (23.7)	63872 (10.9)	727 (22.5)	10486 (27.5)
Angina	87970 (15.0)	1526 (25.6)	91026 (15.6)	883 (27.4)	10973 (28.8)
Previous myocardial infarction	63738 (10.9)	1249 (20.9)	60576 (10.3)	645 (20.0)	8239 (21.6)
Previous cardiac revascularisation	57332 (9.8)	949 (15.9)	54961 (9.4)	497 (15.4)	5926 (15.6)
Other ischaemic heart disease	121776 (20.8)	2102 (35.2)	121753 (20.8)	1170 (36.3)	14645 (38.5)

For peer review only - http://bmjopen.bmj.com/site/about/guidelines.xhtml

	2020 cohort	Covid-19 hospitalisations (in 2020 cohort)	2016 cohort	Influenza hospitalisations (in 2016 cohort)	Pneumonia hospitalisations (in 2016 cohort)
Heart failure	61014 (10.4)	1573 (26.4)	53579 (9.2)	735 (22.8)	10402 (27.3)
Peripheral arterial disease	50045 (8.6)	1157 (19.4)	48489 (8.3)	481 (14.9)	7700 (20.2)
**Recent hospitalisation**					
Respiratory infection	25535 (4.4)	1049 (17.6)	21353 (3.6)	436 (13.5)	6127 (16.1)
Anything else	96421 (16.5)	2426 (40.7)	95232 (16.3)	1195 (37.0)	15007 (39.4)
**Respiratory**					
Asthma	125582 (21.5)	1567 (26.3)	118207 (20.2)	1063 (33.0)	11229 (29.5)
Chronic obstructive pulmonary disease	65615 (11.2)	1273 (21.3)	60108 (10.3)	806 (25.0)	10948 (28.7)
**Neurological**					
Previous transient ischaemic attack	31024 (5.3)	598 (10.0)	29997 (5.1)	294 (9.1)	4200 (11.0)
Previous stroke	49873 (8.5)	987 (16.5)	47721 (8.2)	504 (15.6)	6839 (18.0)
Dementia	20629 (3.5)	718 (12.0)	19548 (3.3)	226 (7.0)	3710 (9.7)
Other neurological condition	35000 (6.0)	741 (12.4)	33569 (5.7)	352 (10.9)	4967 (13.0)
**Oncological**					
Haematological cancer	11751 (2.0)	212 (3.6)	10352 (1.8)	155 (4.8)	1499 (3.9)
Solid cancer	79179 (13.5)	1130 (18.9)	72001 (12.3)	544 (16.9)	7887 (20.7)
**Other**					
Solid organ transplant	3682 (0.6)	99 (1.7)	3013 (0.5)	49 (1.5)	509 (1.3)
Chronic liver disease	69136 (11.8)	860 (14.4)	48011 (8.2)	333 (10.3)	3387 (8.9)
**Chronic Kidney Disease (CKD) stage**					
Stage 1	165845 (28.4)	933 (15.6)	147798 (25.3)	448 (13.9)	3873 (10.2)
Stage 2	286696 (49.0)	2438 (40.9)	290821 (49.7)	1399 (43.4)	15243 (40.0)
Stage 3a	72539 (12.4)	1079 (18.1)	80222 (13.7)	642 (19.9)	8056 (21.2)
Stage 3b	33246 (5.7)	699 (11.7)	37256 (6.4)	372 (11.5)	5844 (15.3)
Stage 4	9006 (1.5)	301 (5.0)	10490 (1.8)	143 (4.4)	2323 (6.1)
Stage 5	12871 (2.2)	496 (8.3)	13365 (2.3)	205 (6.4)	2543 (6.7)
Missing	4651 (0.8)	19 (0.3)	5337 (0.9)	17 (0.5)	206 (0.5)
**Albumin creatinine ratio (ACR) category**					
A1	402847 (68.9)	3284 (55.1)	388839 (66.4)	1812 (56.2)	19926 (52.3)
A2	121002 (20.7)	1719 (28.8)	106879 (18.3)	845 (26.2)	11550 (30.3)
A3	21899 (3.7)	614 (10.3)	18817 (3.2)	252 (7.8)	3219 (8.5)
Missing	39106 (6.7)	348 (5.8)	70754 (12.1)	317 (9.8)	3393 (8.9)
**Diabetes treatment (last 6 months)**					
Insulin prescription	79286 (13.6)	1490 (25.0)	78755 (13.5)	742 (23.0)	8481 (22.3)
OHA prescription only	362651 (62.0)	3167 (53.1)	349629 (59.7)	1739 (53.9)	19713 (51.8)
No treatment	142917 (24.4)	1308 (21.9)	156905 (26.8)	745 (23.1)	9894 (26.0)

For peer review only

Supplemental figure 2. Association of potential risk factors with hospitalisation for Covid-19 (2020), influenza (2016-2019), and pneumonia (2016-2019) in type 2 diabetes.

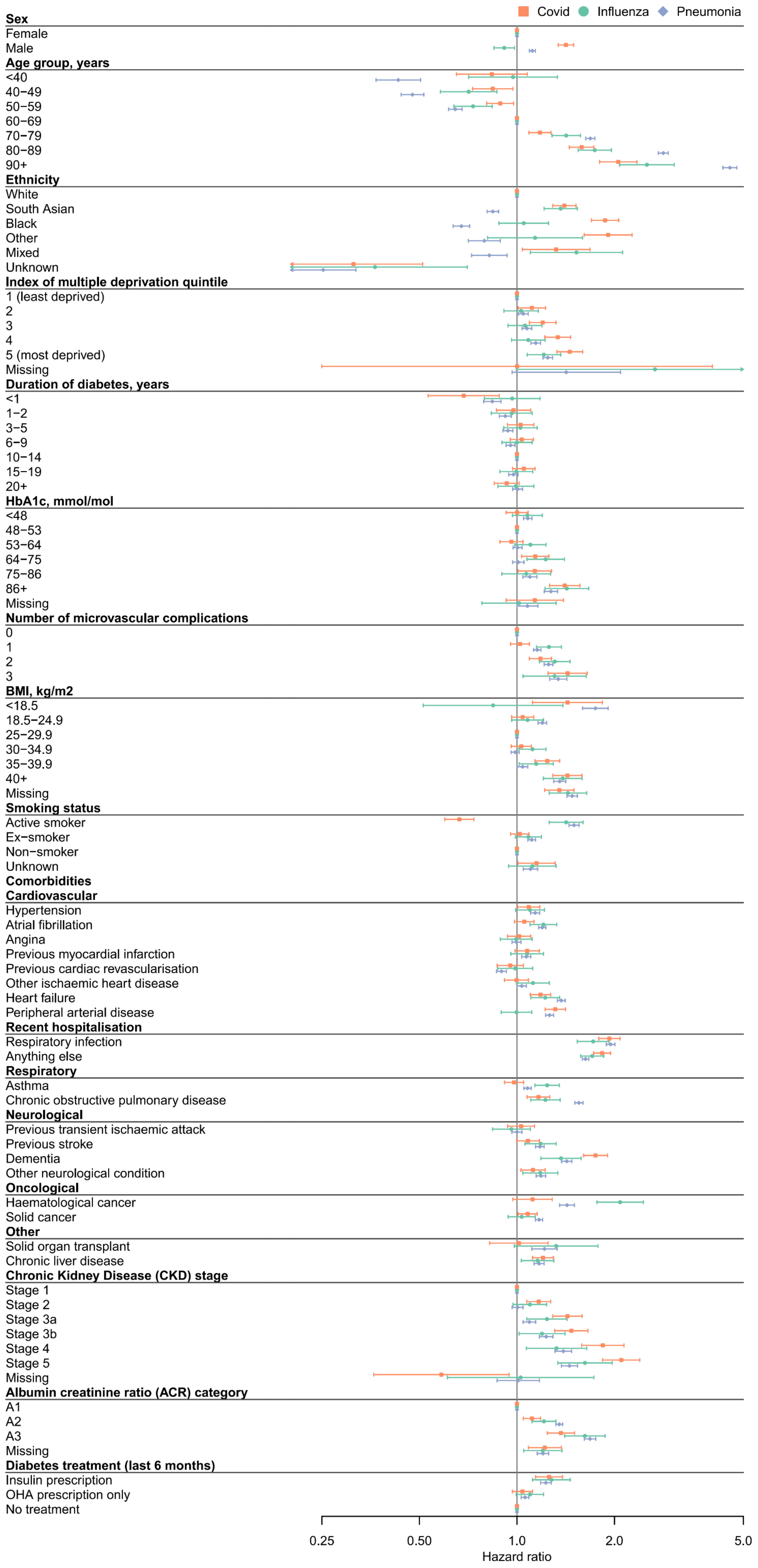

For peer review only - http://bmjopen.bmj.com/site/about/guidelines.xhtml

Supplemental table 5. Hazard ratio (HR) values and 95% confidence intervals (95% CI) for the association of potential risk factors with hospitalisation for Covid-19 (2020), influenza (2016-2019), and pneumonia (2016-2019) in type 2 diabetes.

	Covid-19		Influenza		Pneumonia	
	**HR (95% CI)**	**p value**	**HR (95% CI)**	**p value**	**HR (95% CI)**	**p value**
**Sex**						
Female	1 (ref)		1 (ref)		1 (ref)	
Male	1.42 (1.34-1.5)	<0.001	0.91 (0.85-0.98)	0.016	1.12 (1.09-1.14)	<0.001
**Age group, years**						
<40	0.84 (0.65-1.08)	0.165	0.97 (0.71-1.33)	0.862	0.43 (0.37-0.5)	<0.001
40-49	0.84 (0.73-0.97)	0.019	0.71 (0.58-0.87)	<0.001	0.48 (0.44-0.52)	<0.001
50-59	0.89 (0.81-0.98)	0.014	0.73 (0.64-0.84)	<0.001	0.65 (0.62-0.68)	<0.001
60-69	1 (ref)		1 (ref)		1 (ref)	
70-79	1.18 (1.09-1.27)	<0.001	1.42 (1.28-1.57)	<0.001	1.68 (1.63-1.74)	<0.001
80-89	1.58 (1.45-1.73)	<0.001	1.74 (1.54-1.96)	<0.001	2.83 (2.73-2.93)	<0.001
90+	2.05 (1.8-2.35)	<0.001	2.52 (2.07-3.06)	<0.001	4.54 (4.32-4.77)	<0.001
**Ethnicity**						
White	1 (ref)		1 (ref)		1 (ref)	
South Asian	1.4 (1.29-1.52)	<0.001	1.36 (1.21-1.54)	<0.001	0.84 (0.81-0.88)	<0.001
Black	1.87 (1.7-2.06)	<0.001	1.05 (0.88-1.25)	0.593	0.67 (0.64-0.71)	<0.001
Other	1.91 (1.61-2.27)	<0.001	1.14 (0.81-1.59)	0.456	0.79 (0.71-0.89)	<0.001
Mixed	1.32 (1.04-1.68)	0.023	1.53 (1.1-2.12)	0.011	0.82 (0.72-0.93)	0.002
Unknown	0.31 (0.19-0.51)	<0.001	0.36 (0.19-0.7)	0.003	0.25 (0.2-0.32)	<0.001
**Index of multiple deprivation quintile**						
1 (least deprived)	1 (ref)		1 (ref)		1 (ref)	
2	1.11 (1.01-1.23)	0.031	1.03 (0.91-1.16)	0.637	1.05 (1.01-1.08)	0.009
3	1.2 (1.09-1.32)	<0.001	1.06 (0.94-1.19)	0.357	1.07 (1.04-1.11)	<0.001
4	1.34 (1.22-1.46)	<0.001	1.08 (0.96-1.22)	0.185	1.14 (1.1-1.18)	<0.001
5 (most deprived)	1.46 (1.33-1.6)	<0.001	1.21 (1.08-1.36)	0.002	1.25 (1.2-1.29)	<0.001
Missing	1 (0.25-4.01)	0.999	2.67 (1-7.14)	0.051	1.42 (0.97-2.09)	0.075
**Duration of diagnosed diabetes, years**						
<1	0.68 (0.53-0.88)	0.003	0.97 (0.79-1.18)	0.739	0.84 (0.79-0.89)	<0.001
1-2	0.98 (0.86-1.1)	0.693	0.96 (0.83-1.11)	0.619	0.92 (0.88-0.96)	<0.001
3-5	1.03 (0.93-1.13)	0.585	1.03 (0.91-1.15)	0.683	0.94 (0.9-0.97)	<0.001
6-9	1.04 (0.95-1.12)	0.408	1 (0.9-1.11)	0.992	0.96 (0.93-0.99)	0.004
10-14	1 (ref)		1 (ref)		1 (ref)	
15-19	1.05 (0.97-1.14)	0.232	1 (0.89-1.12)	0.935	0.97 (0.94-1.01)	0.135
20+	0.93 (0.85-1.02)	0.108	0.99 (0.87-1.13)	0.906	1.01 (0.97-1.04)	0.774
**HbA1c, mmol/mol**						
<48	1 (0.93-1.08)	0.975	1.08 (0.97-1.2)	0.172	1.08 (1.05-1.11)	<0.001
48-53	1 (ref)		1 (ref)		1 (ref)	
53-64	0.96 (0.89-1.04)	0.349	1.1 (0.99-1.23)	0.09	1 (0.97-1.04)	0.776
64-75	1.14 (1.03-1.25)	0.009	1.23 (1.07-1.4)	0.003	1.01 (0.97-1.05)	0.642
75-86	1.13 (1.01-1.28)	0.037	1.07 (0.9-1.27)	0.457	1.1 (1.04-1.15)	<0.001
86+	1.4 (1.26-1.56)	<0.001	1.43 (1.22-1.66)	<0.001	1.27 (1.21-1.33)	<0.001
Missing	1.14 (0.93-1.39)	0.22	1.01 (0.78-1.32)	0.913	1.08 (1-1.16)	0.048
**Number of microvascular complications**						
0	1 (ref)		1 (ref)		1 (ref)	
1	1.02 (0.96-1.09)	0.527	1.26 (1.15-1.37)	<0.001	1.16 (1.13-1.19)	<0.001
2	1.18 (1.09-1.28)	<0.001	1.31 (1.17-1.46)	<0.001	1.25 (1.21-1.29)	<0.001
3	1.43 (1.25-1.65)	<0.001	1.31 (1.04-1.64)	0.019	1.34 (1.26-1.43)	<0.001
**BMI, kg/m2**						
<18.5	1.43 (1.12-1.84)	0.005	0.84 (0.51-1.39)	0.504	1.75 (1.59-1.91)	<0.001
18.5-24.9	1.04 (0.96-1.13)	0.312	1.08 (0.96-1.21)	0.19	1.2 (1.16-1.23)	<0.001
25-29.9	1 (ref)		1 (ref)		1 (ref)	
30-34.9	1.03 (0.96-1.11)	0.397	1.12 (1.01-1.23)	0.025	0.99 (0.96-1.02)	0.383
35-39.9	1.24 (1.14-1.35)	<0.001	1.15 (1.02-1.29)	0.026	1.04 (1-1.08)	0.035
40+	1.43 (1.29-1.59)	<0.001	1.39 (1.21-1.59)	<0.001	1.35 (1.3-1.41)	<0.001
Missing	1.35 (1.22-1.5)	<0.001	1.44 (1.26-1.64)	<0.001	1.48 (1.43-1.54)	<0.001
**Smoking status**						
Active smoker	0.66 (0.6-0.74)	<0.001	1.42 (1.26-1.6)	<0.001	1.5 (1.45-1.55)	<0.001
Ex-smoker	1.02 (0.96-1.09)	0.552	1.09 (0.99-1.19)	0.083	1.11 (1.08-1.14)	<0.001
Non-smoker	1 (ref)		1 (ref)		1 (ref)	
Unknown	1.15 (1.01-1.31)	0.039	1.12 (0.94-1.32)	0.204	1.1 (1.05-1.16)	<0.001
**Comorbidities**						
**Cardiovascular**						
Hypertension	1.09 (1.01-1.18)	0.037	1.1 (0.99-1.22)	0.077	1.14 (1.1-1.17)	<0.001
Atrial fibrillation	1.05 (0.98-1.13)	0.139	1.21 (1.1-1.33)	<0.001	1.2 (1.17-1.23)	<0.001
Angina	1.02 (0.93-1.1)	0.714	0.99 (0.89-1.11)	0.927	1 (0.97-1.03)	0.836
Previous myocardial infarction	1.08 (0.99-1.17)	0.099	1.08 (0.96-1.21)	0.221	1.07 (1.03-1.1)	<0.001
Previous cardiac revascularisation	0.95 (0.87-1.05)	0.315	0.99 (0.87-1.12)	0.841	0.9 (0.86-0.93)	<0.001
Other ischaemic heart disease	1 (0.92-1.09)	0.938	1.12 (1-1.26)	0.057	1.03 (1-1.07)	0.041
Heart failure	1.18 (1.1-1.27)	<0.001	1.22 (1.1-1.35)	<0.001	1.37 (1.33-1.41)	<0.001
Peripheral arterial disease	1.31 (1.22-1.41)	<0.001	1 (0.89-1.11)	0.957	1.26 (1.23-1.3)	<0.001
**Recent hospitalisation**						
Respiratory infection	1.93 (1.79-2.08)	<0.001	1.72 (1.54-1.92)	<0.001	1.95 (1.89-2.01)	<0.001
Anything else	1.83 (1.73-1.95)	<0.001	1.71 (1.57-1.85)	<0.001	1.63 (1.59-1.67)	<0.001

For peer review only - http://bmjopen.bmj.com/site/about/guidelines.xhtml

	Covid-19		Influenza		Pneumonia	
	HR (95% CI)	p value	HR (95% CI)	p value	HR (95% CI)	p value
**Respiratory**						
Asthma	0.98 (0.92-1.05)	0.547	1.24 (1.14-1.35)	<0.001	1.08 (1.05-1.11)	<0.001
Chronic obstructive pulmonary disease	1.16 (1.07-1.26)	<0.001	1.22 (1.1-1.36)	<0.001	1.55 (1.51-1.6)	<0.001
**Neurological**						
Previous transient ischaemic attack	1.03 (0.94-1.13)	0.543	0.96 (0.84-1.1)	0.568	1 (0.96-1.04)	0.991
Previous stroke	1.08 (1-1.17)	0.06	1.18 (1.06-1.32)	0.003	1.18 (1.14-1.21)	<0.001
Dementia	1.75 (1.61-1.9)	<0.001	1.37 (1.19-1.58)	<0.001	1.42 (1.37-1.48)	<0.001
Other neurological condition	1.12 (1.03-1.22)	0.009	1.18 (1.04-1.34)	0.009	1.19 (1.15-1.23)	<0.001
**Oncological**						
Haematological cancer	1.12 (0.97-1.29)	0.121	2.08 (1.76-2.46)	<0.001	1.43 (1.35-1.5)	<0.001
Solid cancer	1.08 (1.01-1.15)	0.027	1.03 (0.94-1.14)	0.49	1.17 (1.14-1.2)	<0.001
**Other**						
Solid organ transplant	1.01 (0.82-1.25)	0.897	1.32 (0.98-1.78)	0.066	1.22 (1.11-1.33)	<0.001
Chronic liver disease	1.2 (1.12-1.3)	<0.001	1.16 (1.03-1.3)	0.013	1.17 (1.13-1.21)	<0.001
**Chronic Kidney Disease (CKD) stage**						
Stage 1	1 (ref)		1 (ref)		1 (ref)	
Stage 2	1.17 (1.07-1.27)	<0.001	1.1 (0.97-1.24)	0.132	1 (0.97-1.04)	0.827
Stage 3a	1.43 (1.29-1.59)	<0.001	1.24 (1.07-1.43)	0.004	1.09 (1.04-1.14)	<0.001
Stage 3b	1.47 (1.31-1.66)	<0.001	1.19 (1.01-1.41)	0.033	1.23 (1.17-1.29)	<0.001
Stage 4	1.84 (1.58-2.14)	<0.001	1.33 (1.07-1.64)	0.01	1.39 (1.31-1.48)	<0.001
Stage 5	2.1 (1.84-2.39)	<0.001	1.62 (1.34-1.97)	<0.001	1.45 (1.37-1.54)	<0.001
Missing	0.58 (0.36-0.95)	0.029	1.03 (0.61-1.73)	0.921	1.01 (0.87-1.17)	0.903
**Albumin creatinine ratio (ACR) category**						
A1	1 (ref)		1 (ref)		1 (ref)	
A2	1.11 (1.05-1.18)	<0.001	1.21 (1.11-1.32)	<0.001	1.35 (1.32-1.38)	<0.001
A3	1.37 (1.24-1.51)	<0.001	1.62 (1.4-1.87)	<0.001	1.68 (1.61-1.75)	<0.001
Missing	1.22 (1.08-1.37)	<0.001	1.2 (1.05-1.38)	0.008	1.2 (1.15-1.25)	<0.001
**Diabetes treatment (last 6 months)**						
Insulin prescription	1.26 (1.14-1.38)	<0.001	1.28 (1.12-1.46)	<0.001	1.23 (1.18-1.28)	<0.001
OHA prescription only	1.04 (0.97-1.12)	0.286	1.1 (1-1.21)	0.062	1.06 (1.03-1.09)	<0.001
No treatment	1 (ref)		1 (ref)		1 (ref)	

For peer review only

Supplemental figure 3A. Association of continuous HbA1c with hospitalisation for Covid-19 (2020), influenza (2016-2019), and pneumonia (2016-2019) in type 2 diabetes by ethnicity subgroup. Density plots show the distribution of HbA1c in each group.

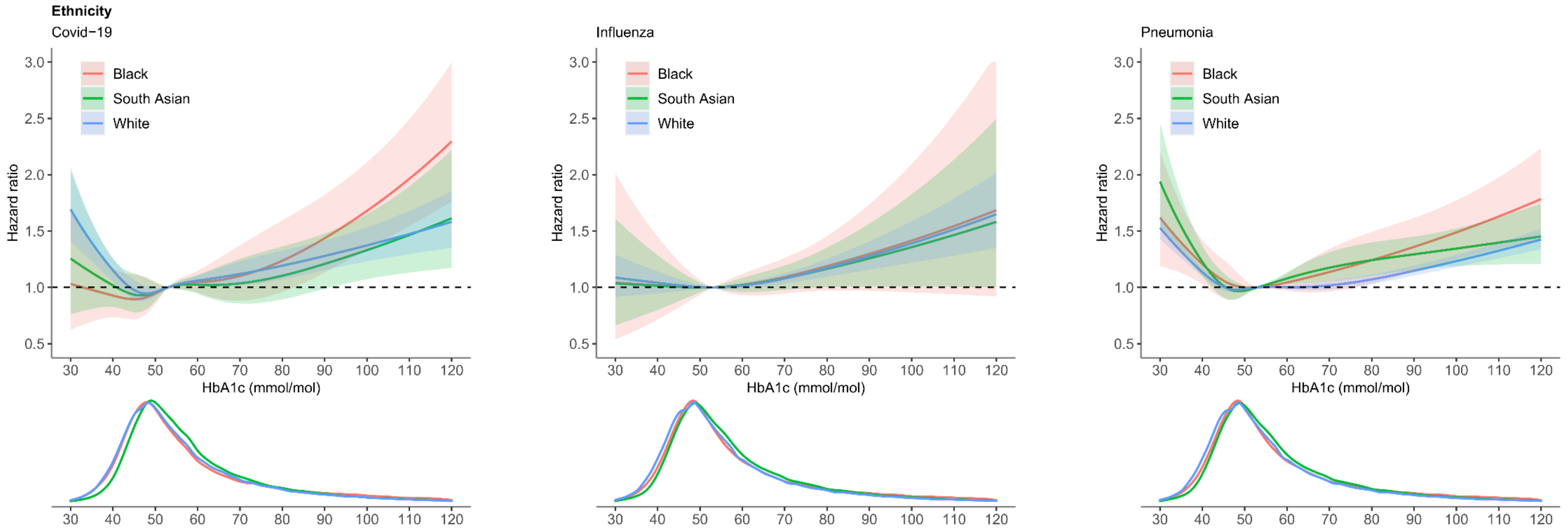

Supplemental figure 3B. Association of continuous BMI with hospitalisation for Covid-19 (2020), influenza (2016-2019), and pneumonia (2016-2019) in type 2 diabetes by ethnicity subgroup. Density plots show the distribution of BMI in each group.

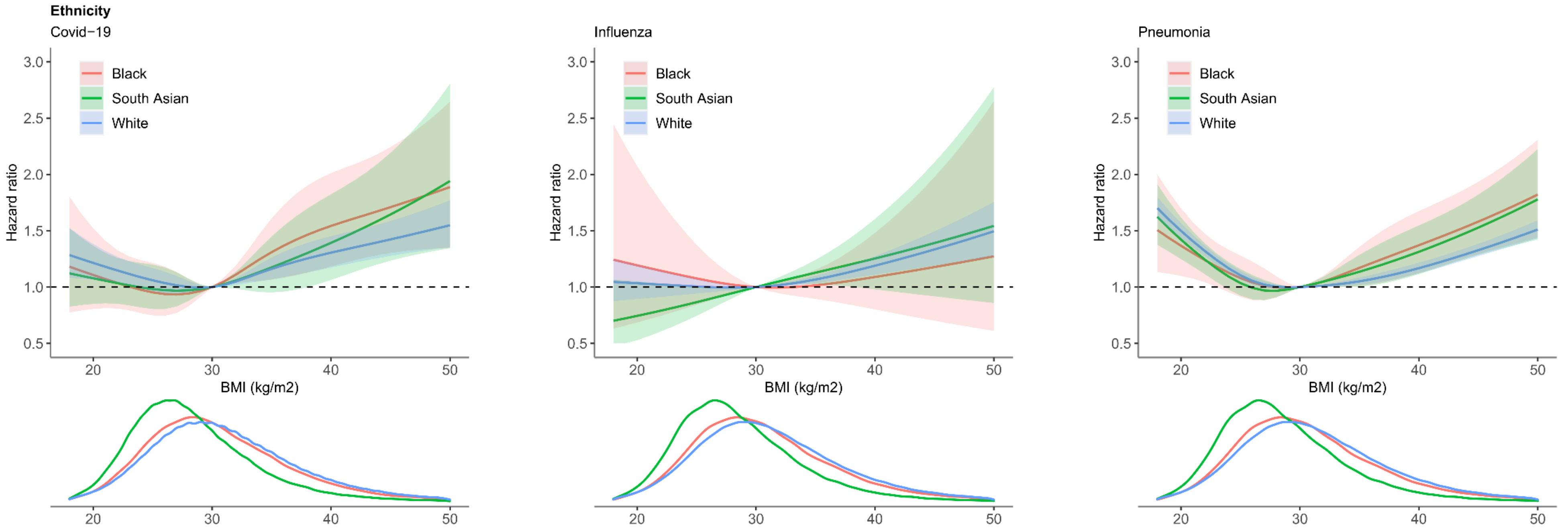

For peer review only - http://bmjopen.bmj.com/site/about/guidelines.xhtml

Supplemental table 6. Baseline characteristics and Covid-19 (2020) and pneumonia (2016-2019) deaths in type 2 diabetes.

	2020 cohort	Covid-19 deaths (in 2020 cohort)	2016 cohort	Pneumonia deaths (in 2016 cohort)
**Number of individuals**	584854	2635	585289	12652
**Mean (SD) follow-up, days**		266.1 (35.5)		917.0 (223.2)
**Sex**				
Female	253360 (43.3)	1023 (38.8)	256656 (43.9)	5499 (43.5)
Male	331494 (56.7)	1612 (61.2)	328633 (56.1)	7153 (56.5)
**Age group, years**				
<40	12898 (2.2)	7 (0.3)	13468 (2.3)	9 (0.1)
40-49	46075 (7.9)	38 (1.4)	49649 (8.5)	91 (0.7)
50-59	114537 (19.6)	150 (5.7)	113285 (19.4)	404 (3.2)
60-69	151493 (25.9)	381 (14.5)	153782 (26.3)	1401 (11.1)
70-79	155424 (26.6)	729 (27.7)	152401 (26.0)	3667 (29.0)
80-89	89871 (15.4)	1047 (39.7)	88492 (15.1)	5370 (42.4)
90+	14556 (2.5)	283 (10.7)	14212 (2.4)	1710 (13.5)
**Ethnicity**				
White	445160 (76.1)	1944 (73.8)	457714 (78.2)	11398 (90.1)
South Asian	77253 (13.2)	341 (12.9)	71050 (12.1)	748 (5.9)
Black	35144 (6.0)	267 (10.1)	33175 (5.7)	326 (2.6)
Other	9886 (1.7)	52 (2.0)	8178 (1.4)	86 (0.7)
Mixed	6267 (1.1)	21 (0.8)	5654 (1.0)	66 (0.5)
Unknown	11144 (1.9)	10 (0.4)	9518 (1.6)	28 (0.2)
**Index of multiple deprivation quintile**				
1 (least deprived)	102950 (17.6)	371 (14.1)	103184 (17.6)	2177 (17.2)
2	106968 (18.3)	418 (15.9)	109006 (18.6)	2378 (18.8)
3	113454 (19.4)	488 (18.5)	113661 (19.4)	2481 (19.6)
4	127048 (21.7)	607 (23.0)	125492 (21.4)	2708 (21.4)
5 (most deprived)	134146 (22.9)	749 (28.4)	133563 (22.8)	2904 (23.0)
Missing	288 (0.0)	2 (0.1)	383 (0.1)	4 (0.0)
**Duration of diagnosed diabetes, years**				
<1	14311 (2.4)	13 (0.5)	36503 (6.2)	331 (2.6)
1-2	60219 (10.3)	120 (4.6)	77510 (13.2)	845 (6.7)
3-5	105094 (18.0)	315 (12.0)	111199 (19.0)	1553 (12.3)
6-9	128817 (22.0)	438 (16.6)	125083 (21.4)	2294 (18.1)
10-14	124471 (21.3)	562 (21.3)	126421 (21.6)	3349 (26.5)
15-19	89704 (15.3)	604 (22.9)	62373 (10.7)	2112 (16.7)
20+	62238 (10.6)	583 (22.1)	46200 (7.9)	2168 (17.1)
**HbA1c, mmol/mol**				
<48	167739 (28.7)	838 (31.8)	175659 (30.0)	4411 (34.9)
48-53	116015 (19.8)	453 (17.2)	116131 (19.8)	2370 (18.7)
53-64	147209 (25.2)	567 (21.5)	142662 (24.4)	2862 (22.6)
64-75	66071 (11.3)	349 (13.2)	62291 (10.6)	1269 (10.0)
75-86	33970 (5.8)	168 (6.4)	32257 (5.5)	642 (5.1)
86+	39727 (6.8)	189 (7.2)	37470 (6.4)	756 (6.0)
Missing	14123 (2.4)	71 (2.7)	18819 (3.2)	342 (2.7)
**Number of microvascular complications**				
0	264011 (45.1)	706 (26.8)	281811 (48.1)	3447 (27.2)
1	216115 (37.0)	957 (36.3)	204550 (34.9)	4804 (38.0)
2	96948 (16.6)	804 (30.5)	91671 (15.7)	3868 (30.6)
3	7780 (1.3)	168 (6.4)	7257 (1.2)	533 (4.2)
**BMI, kg/m2**				
<18.5	3026 (0.5)	47 (1.8)	2801 (0.5)	319 (2.5)
18.5-24.9	86199 (14.7)	587 (22.3)	79018 (13.5)	2900 (22.9)
25-29.9	184626 (31.6)	755 (28.7)	178501 (30.5)	3616 (28.6)
30-34.9	151732 (25.9)	526 (20.0)	151161 (25.8)	2236 (17.7)
35-39.9	74766 (12.8)	246 (9.3)	75339 (12.9)	968 (7.7)
40+	48455 (8.3)	166 (6.3)	48685 (8.3)	656 (5.2)
Missing	36050 (6.2)	308 (11.7)	49784 (8.5)	1957 (15.5)
**Smoking status**				
Active smoker	87220 (14.9)	234 (8.9)	90218 (15.4)	1937 (15.3)
Ex-smoker	336719 (57.6)	1742 (66.1)	316723 (54.1)	7553 (59.7)
Non-smoker	138840 (23.7)	551 (20.9)	149524 (25.5)	2478 (19.6)
Unknown	22075 (3.8)	108 (4.1)	28824 (4.9)	684 (5.4)
**Comorbidities**				
**Cardiovascular**				
Hypertension	425278 (72.7)	2334 (88.6)	419645 (71.7)	11173 (88.3)
Atrial fibrillation	70633 (12.1)	769 (29.2)	63872 (10.9)	4175 (33.0)
Angina	87970 (15.0)	749 (28.4)	91026 (15.6)	3842 (30.4)
Previous myocardial infarction	63738 (10.9)	633 (24.0)	60576 (10.3)	3064 (24.2)
Previous cardiac revascularisation	57332 (9.8)	462 (17.5)	54961 (9.4)	2035 (16.1)
Other ischaemic heart disease	121776 (20.8)	1022 (38.8)	121753 (20.8)	5196 (41.1)
Heart failure	61014 (10.4)	848 (32.2)	53579 (9.2)	4195 (33.2)
Peripheral arterial disease	50045 (8.6)	635 (24.1)	48489 (8.3)	3060 (24.2)

For peer review only - http://bmjopen.bmj.com/site/about/guidelines.xhtml

	2020 cohort	Covid-19 deaths (in 2020 cohort)	2016 cohort	Pneumonia deaths (in 2016 cohort)
**Recent hospitalisation**				
Respiratory infection	25535 (4.4)	563 (21.4)	21353 (3.6)	2387 (18.9)
Anything else	96421 (16.5)	1171 (44.4)	95232 (16.3)	5409 (42.8)
**Respiratory**				
Asthma	125582 (21.5)	683 (25.9)	118207 (20.2)	3395 (26.8)
Chronic obstructive pulmonary disease	65615 (11.2)	642 (24.4)	60108 (10.3)	3755 (29.7)
**Neurological**				
Previous transient ischaemic attack	31024 (5.3)	334 (12.7)	29997 (5.1)	1597 (12.6)
Previous stroke	49873 (8.5)	570 (21.6)	47721 (8.2)	2591 (20.5)
Dementia	20629 (3.5)	572 (21.7)	19548 (3.3)	1831 (14.5)
Other neurological condition	35000 (6.0)	447 (17.0)	33569 (5.7)	1974 (15.6)
**Oncological**				
Haematological cancer	11751 (2.0)	119 (4.5)	10352 (1.8)	550 (4.3)
Solid cancer	79179 (13.5)	555 (21.1)	72001 (12.3)	2965 (23.4)
**Other**				
Solid organ transplant	3682 (0.6)	41 (1.6)	3013 (0.5)	193 (1.5)
Chronic liver disease	69136 (11.8)	322 (12.2)	48011 (8.2)	1034 (8.2)
**Chronic Kidney Disease (CKD) stage**				
Stage 1	165845 (28.4)	239 (9.1)	147798 (25.3)	988 (7.8)
Stage 2	286696 (49.0)	968 (36.7)	290821 (49.7)	4481 (35.4)
Stage 3a	72539 (12.4)	553 (21.0)	80222 (13.7)	2787 (22.0)
Stage 3b	33246 (5.7)	415 (15.7)	37256 (6.4)	2279 (18.0)
Stage 4	9006 (1.5)	186 (7.1)	10490 (1.8)	999 (7.9)
Stage 5	12871 (2.2)	261 (9.9)	13365 (2.3)	1043 (8.2)
Missing	4651 (0.8)	13 (0.5)	5337 (0.9)	75 (0.6)
**Albumin creatinine ratio (ACR) category**				
A1	402847 (68.9)	1271 (48.2)	388839 (66.4)	6044 (47.8)
A2	121002 (20.7)	845 (32.1)	106879 (18.3)	4334 (34.3)
A3	21899 (3.7)	322 (12.2)	18817 (3.2)	1281 (10.1)
Missing	39106 (6.7)	197 (7.5)	70754 (12.1)	993 (7.8)
**Diabetes treatment (last 6 months)**				
Insulin prescription	79286 (13.6)	682 (25.9)	78755 (13.5)	2772 (21.9)
OHA prescription only	362651 (62.0)	1289 (48.9)	349629 (59.7)	6309 (49.9)
No treatment	142917 (24.4)	664 (25.2)	156905 (26.8)	3571 (28.2)

For peer review only

Supplemental figure 4. Association of potential risk factors with Covid-19 (2020), and pneumonia (2016-2019) mortality in type 2 diabetes.

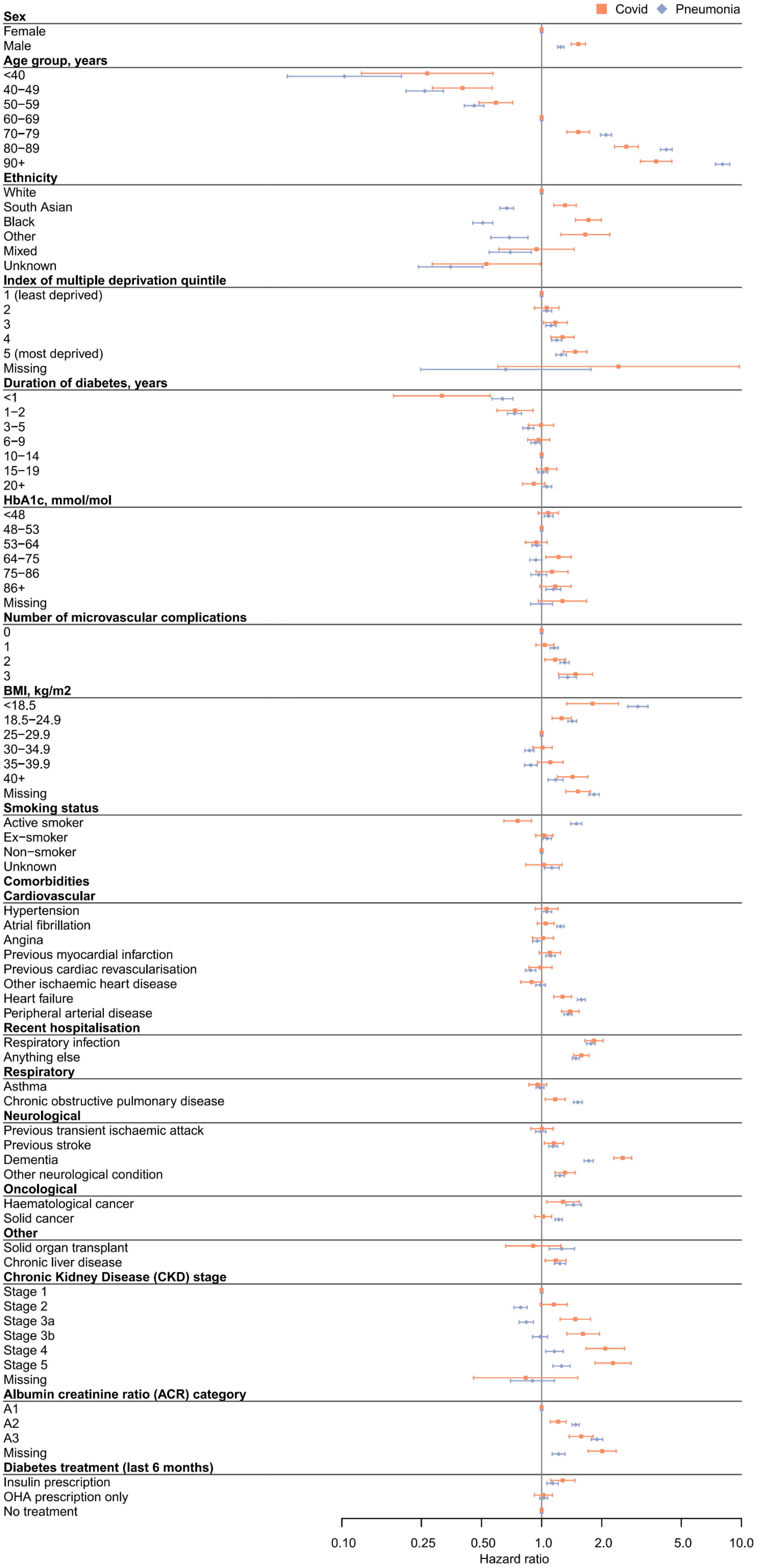

For peer review only - http://bmjopen.bmj.com/site/about/guidelines.xhtml

Supplemental table 7. Baseline characteristics and Covid-19 (2020), influenza (2016-2019), and pneumonia (2016-2019) hospitalisations in type 1 diabetes.

	2020 cohort	Covid-19 hospitalisations (in 2020 cohort)	2016 cohort	Influenza hospitalisations (in 2016 cohort)	Pneumonia hospitalisations (in 2016 cohort)
**Number of individuals**	43033	209	42488	208	1401
**Mean (SD) follow-up, days**		267.0 (33.2)		917.3 (223.0)	907.1 (236.2)
**Sex**					
Female	18172 (42.2)	76 (36.4)	17889 (42.1)	103 (49.5)	611 (43.6)
Male	24861 (57.8)	133 (63.6)	24599 (57.9)	105 (50.5)	790 (56.4)
**Age, years**					
<18	4458 (10.4)	1 (0.5)	4502 (10.6)	12 (5.8)	13 (0.9)
18-39	14454 (33.6)	34 (16.3)	13996 (32.9)	59 (28.4)	191 (13.6)
40-49	7480 (17.4)	31 (14.8)	7995 (18.8)	43 (20.7)	208 (14.8)
50-59	8056 (18.7)	42 (20.1)	7911 (18.6)	31 (14.9)	283 (20.2)
60-69	5079 (11.8)	43 (20.6)	4731 (11.1)	34 (16.3)	281 (20.1)
70-79	2689 (6.2)	37 (17.7)	2460 (5.8)	23 (11.1)	239 (17.1)
80+	817 (1.9)	21 (10.0)	893 (2.1)	6 (2.9)	186 (13.3)
**Ethnicity**					
White	38478 (89.4)	170 (81.3)	38337 (90.2)	187 (89.9)	1311 (93.6)
South Asian	1576 (3.7)	14 (6.7)	1494 (3.5)	14 (6.7)	37 (2.6)
Black	1385 (3.2)	16 (7.7)	1253 (2.9)	3 (1.4)	33 (2.4)
Other	464 (1.1)	3 (1.4)	373 (0.9)	0 (0.0)	5 (0.4)
Mixed	525 (1.2)	6 (2.9)	476 (1.1)	4 (1.9)	15 (1.1)
Unknown	605 (1.4)	0 (0.0)	555 (1.3)	0 (0.0)	0 (0.0)
**Index of multiple deprivation quintile**					
1 (least deprived)	9395 (21.8)	43 (20.6)	9251 (21.8)	28 (13.5)	254 (18.1)
2	8710 (20.2)	30 (14.4)	8652 (20.4)	37 (17.8)	237 (16.9)
3	8334 (19.4)	33 (15.8)	8347 (19.6)	44 (21.2)	276 (19.7)
4	8444 (19.6)	38 (18.2)	8342 (19.6)	45 (21.6)	292 (20.8)
5 (most deprived)	8128 (18.9)	65 (31.1)	7864 (18.5)	54 (26.0)	342 (24.4)
Missing	22 (0.1)	0 (0.0)	32 (0.1)	0 (0.0)	0 (0.0)
**Duration of diagnosed diabetes, years**					
<5	5655 (13.1)	8 (3.8)	6556 (15.4)	17 (8.2)	81 (5.8)
5-9	6194 (14.4)	13 (6.2)	6077 (14.3)	21 (10.1)	101 (7.2)
10-14	5609 (13.0)	23 (11.0)	5899 (13.9)	28 (13.5)	144 (10.3)
15-19	5466 (12.7)	27 (12.9)	5355 (12.6)	28 (13.5)	174 (12.4)
20-24	4621 (10.7)	25 (12.0)	4612 (10.9)	22 (10.6)	176 (12.6)
25-29	4101 (9.5)	27 (12.9)	3747 (8.8)	27 (13.0)	149 (10.6)
30+	11387 (26.5)	86 (41.1)	10242 (24.1)	65 (31.2)	576 (41.1)
**HbA1c, mmol/mol**					
<48	3104 (7.2)	12 (5.7)	2796 (6.6)	13 (6.2)	109 (7.8)
48-53	3232 (7.5)	13 (6.2)	2967 (7.0)	13 (6.2)	111 (7.9)
53-64	10995 (25.6)	50 (23.9)	10340 (24.3)	37 (17.8)	263 (18.8)
64-75	9951 (23.1)	36 (17.2)	10098 (23.8)	46 (22.1)	283 (20.2)
75-86	5891 (13.7)	30 (14.4)	5979 (14.1)	32 (15.4)	228 (16.3)
86+	6097 (14.2)	57 (27.3)	6351 (14.9)	45 (21.6)	327 (23.3)
Missing	3763 (8.7)	11 (5.3)	3957 (9.3)	22 (10.6)	80 (5.7)
**BMI, kg/m2**					
<18.5	1462 (3.4)	6 (2.9)	1267 (3.0)	1 (0.5)	37 (2.6)
18.5-24.9	13478 (31.3)	61 (29.2)	13132 (30.9)	70 (33.7)	465 (33.2)
25-29.9	12966 (30.1)	57 (27.3)	12606 (29.7)	54 (26.0)	372 (26.6)
30-34.9	6258 (14.5)	37 (17.7)	5704 (13.4)	24 (11.5)	181 (12.9)
35-39.9	1934 (4.5)	16 (7.7)	1735 (4.1)	11 (5.3)	78 (5.6)
40+	949 (2.2)	8 (3.8)	782 (1.8)	8 (3.8)	57 (4.1)
Missing	5986 (13.9)	24 (11.5)	7262 (17.1)	40 (19.2)	211 (15.1)

For peer review only

For peer review only - http://bmjopen.bmj.com/site/about/guidelines.xhtml

Supplemental figure 5. Association of potential risk factors with hospitalisation for Covid-19 (2020), influenza (2016-2019), and pneumonia (2016-2019) in type 1 diabetes.

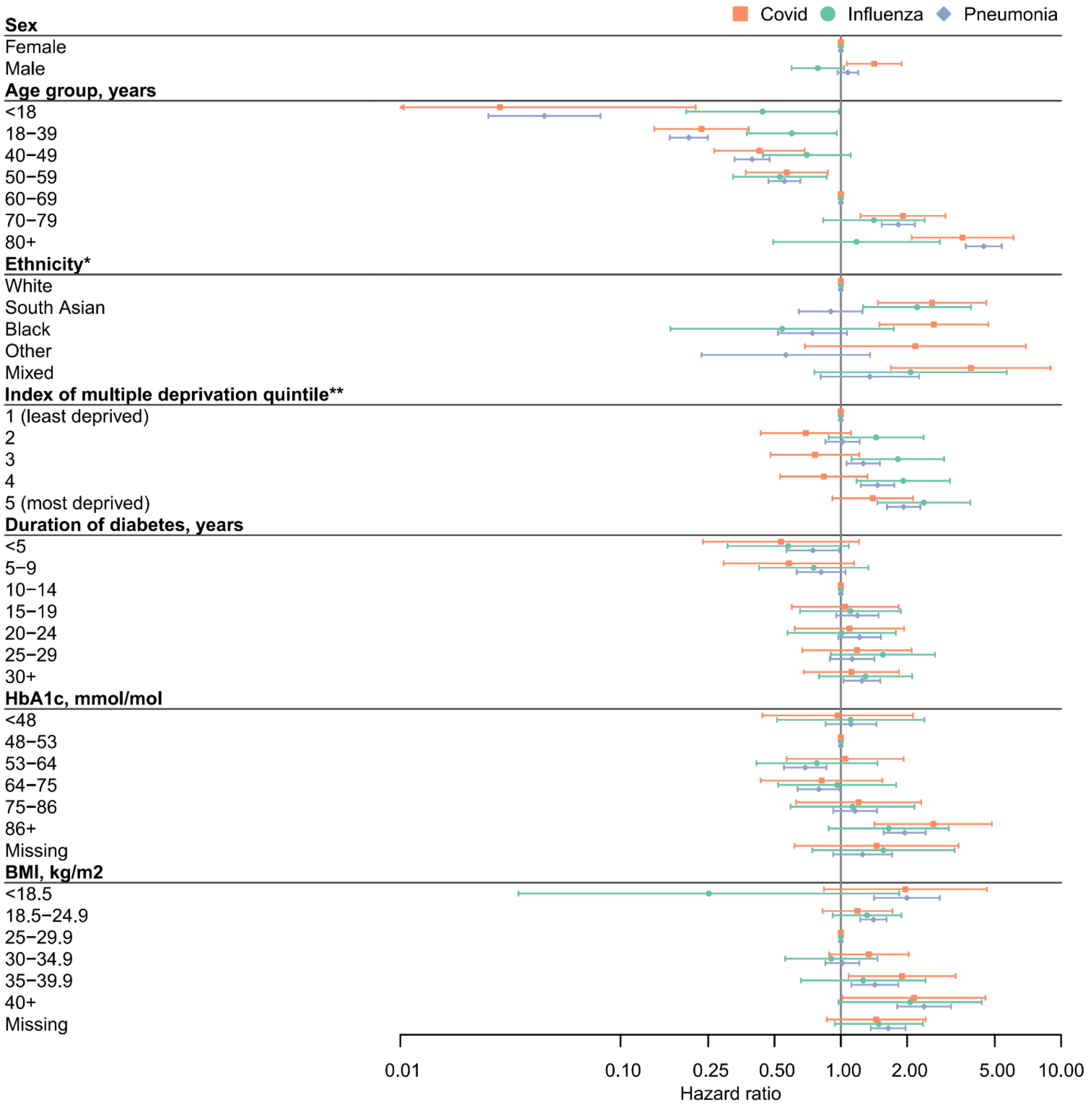

*No influenza hospitalisations occurred in other ethnicity group. No Covid-19, influenza or pneumonia hospitalisations occurred in unknown ethnicity group. ** No Covid-19, influenza or pneumonia hospitalisations occurred in missing IMD group.

For peer review only - http://bmjopen.bmj.com/site/about/guidelines.xhtml

Supplemental figure 6A. Association of continuous HbA1c with hospitalisation for Covid-19 (2020), influenza (2016-2019), and pneumonia (2016-2019) in type 1 diabetes. Density plots show the distribution of HbA1c in each group.

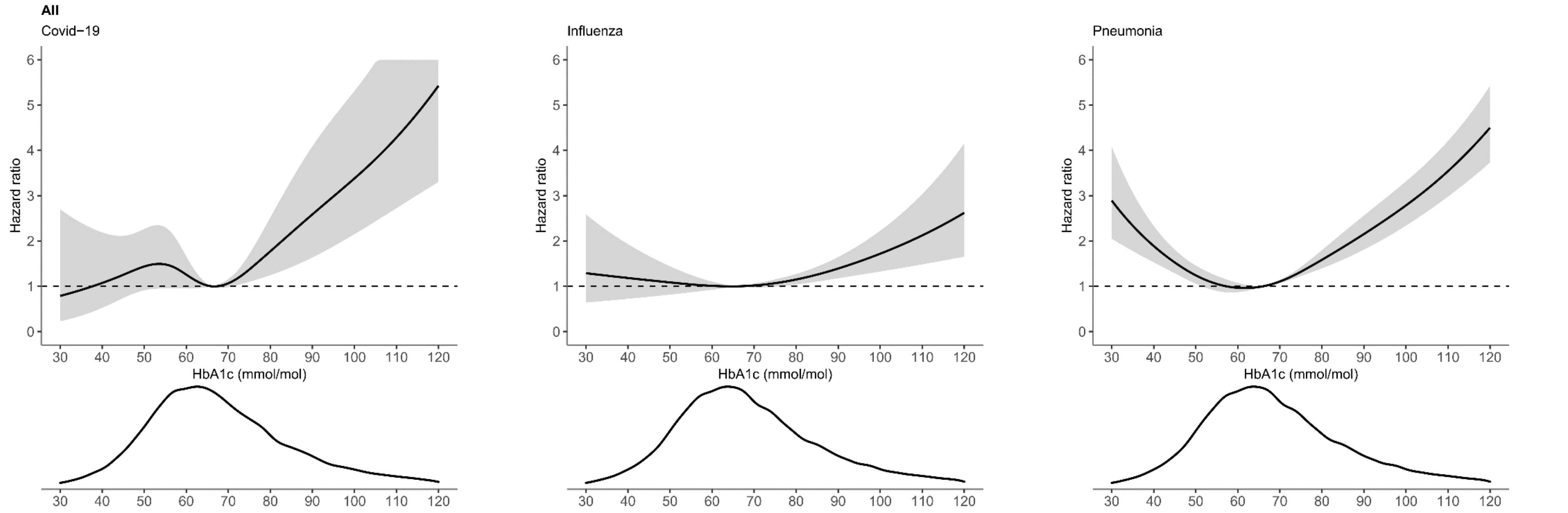

Supplemental figure 6B. Association of continuous BMI with hospitalisation for Covid-19 (2020), influenza (2016-2019), and pneumonia (2016-2019) in type 1 diabetes. Density plots show the distribution of BMI in each group.

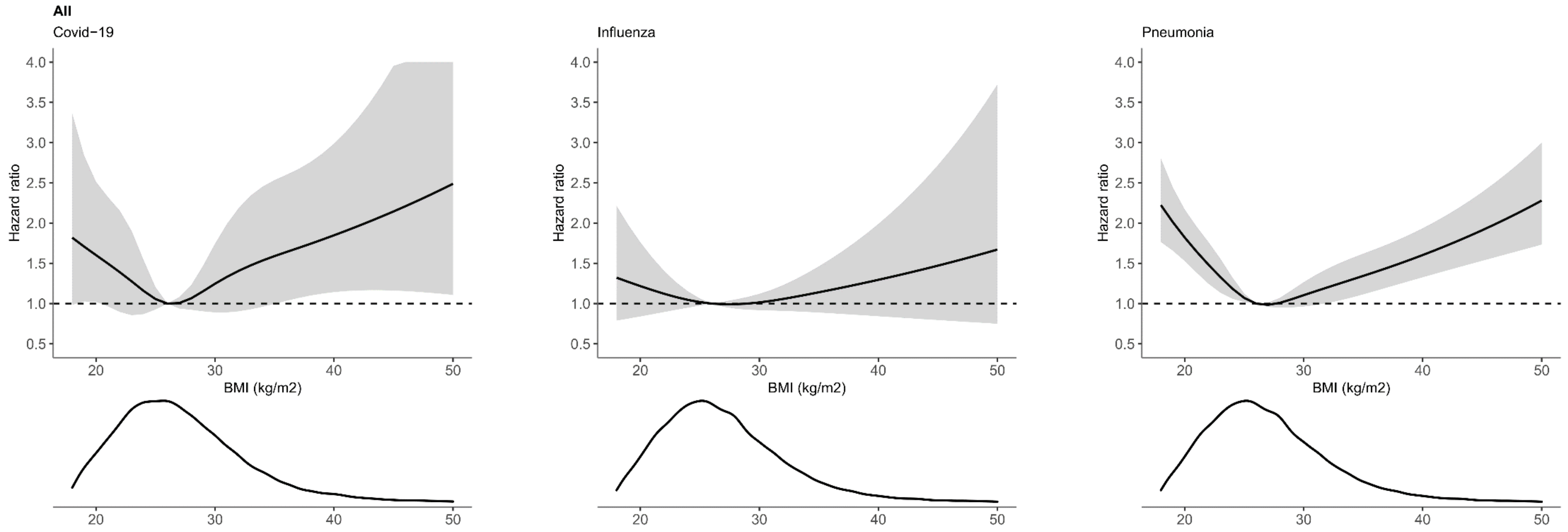

Supplemental table 8. Baseline characteristics and Covid-19, influenza, and pneumonia hospitalisations in type 1 diabetes, restricting hospitalisation definition to primary diagnosis in HES.

	2020 cohort	Covid-19 hospitalisations (in 2020 cohort)	2016 cohort	Influenza hospitalisations (in 2016 cohort)	Pneumonia hospitalisations (in 2016 cohort)
**Number of individuals**	43033	142	42488	130	803
**Mean (SD) follow-up, days**		267.2 (32.6)		917.9 (222.4)	912.1 (230.3)
**Sex**					
Female	18172 (42.2)	45 (31.7)	17889 (42.1)	68 (52.3)	353 (44.0)
Male	24861 (57.8)	97 (68.3)	24599 (57.9)	62 (47.7)	450 (56.0)
**Age, years**					
<18	4458 (10.4)	0 (0.0)	4502 (10.6)	6 (4.6)	9 (1.1)
18-39	14454 (33.6)	20 (14.1)	13996 (32.9)	35 (26.9)	102 (12.7)
40-49	7480 (17.4)	21 (14.8)	7995 (18.8)	22 (16.9)	115 (14.3)
50-59	8056 (18.7)	31 (21.8)	7911 (18.6)	21 (16.2)	154 (19.2)
60-69	5079 (11.8)	31 (21.8)	4731 (11.1)	25 (19.2)	157 (19.6)
70-79	2689 (6.2)	24 (16.9)	2460 (5.8)	17 (13.1)	148 (18.4)
80+	817 (1.9)	15 (10.6)	893 (2.1)	4 (3.1)	118 (14.7)
**Ethnicity**					
White	38478 (89.4)	111 (78.2)	38337 (90.2)	121 (93.1)	754 (93.9)
South Asian	1576 (3.7)	12 (8.5)	1494 (3.5)	8 (6.2)	17 (2.1)
Black	1385 (3.2)	12 (8.5)	1253 (2.9)	0 (0.0)	20 (2.5)
Other	464 (1.1)	2 (1.4)	373 (0.9)	0 (0.0)	3 (0.4)
Mixed	525 (1.2)	5 (3.5)	476 (1.1)	1 (0.8)	9 (1.1)
Unknown	605 (1.4)	0 (0.0)	555 (1.3)	0 (0.0)	0 (0.0)
**Index of multiple deprivation quintile**					
1 (least deprived)	9395 (21.8)	26 (18.3)	9251 (21.8)	22 (16.9)	157 (19.6)
2	8710 (20.2)	20 (14.1)	8652 (20.4)	21 (16.2)	143 (17.8)
3	8334 (19.4)	28 (19.7)	8347 (19.6)	27 (20.8)	163 (20.3)
4	8444 (19.6)	29 (20.4)	8342 (19.6)	26 (20.0)	141 (17.6)
5 (most deprived)	8128 (18.9)	39 (27.5)	7864 (18.5)	34 (26.2)	199 (24.8)
Missing	22 (0.1)	0 (0.0)	32 (0.1)	0 (0.0)	0 (0.0)
**Duration of diagnosed diabetes, years**					
<5	5655 (13.1)	4 (2.8)	6556 (15.4)	7 (5.4)	55 (6.8)
5-9	6194 (14.4)	10 (7.0)	6077 (14.3)	14 (10.8)	58 (7.2)
10-14	5609 (13.0)	18 (12.7)	5899 (13.9)	14 (10.8)	72 (9.0)
15-19	5466 (12.7)	21 (14.8)	5355 (12.6)	17 (13.1)	105 (13.1)
20-24	4621 (10.7)	11 (7.7)	4612 (10.9)	11 (8.5)	95 (11.8)
25-29	4101 (9.5)	22 (15.5)	3747 (8.8)	22 (16.9)	83 (10.3)
30+	11387 (26.5)	56 (39.4)	10242 (24.1)	45 (34.6)	335 (41.7)
**HbA1c, mmol/mol**					
<48	3104 (7.2)	8 (5.6)	2796 (6.6)	9 (6.9)	67 (8.3)
48-53	3232 (7.5)	8 (5.6)	2967 (7.0)	11 (8.5)	66 (8.2)
53-64	10995 (25.6)	35 (24.6)	10340 (24.3)	25 (19.2)	159 (19.8)
64-75	9951 (23.1)	28 (19.7)	10098 (23.8)	32 (24.6)	167 (20.8)
75-86	5891 (13.7)	22 (15.5)	5979 (14.1)	21 (16.2)	117 (14.6)
86+	6097 (14.2)	33 (23.2)	6351 (14.9)	22 (16.9)	179 (22.3)
Missing	3763 (8.7)	8 (5.6)	3957 (9.3)	10 (7.7)	48 (6.0)
**BMI, kg/m2**					
<18.5	1462 (3.4)	2 (1.4)	1267 (3.0)	1 (0.8)	23 (2.9)
18.5-24.9	13478 (31.3)	39 (27.5)	13132 (30.9)	45 (34.6)	267 (33.3)
25-29.9	12966 (30.1)	40 (28.2)	12606 (29.7)	35 (26.9)	215 (26.8)
30-34.9	6258 (14.5)	24 (16.9)	5704 (13.4)	16 (12.3)	95 (11.8)
35-39.9	1934 (4.5)	11 (7.7)	1735 (4.1)	7 (5.4)	53 (6.6)
40+	949 (2.2)	8 (5.6)	782 (1.8)	5 (3.8)	35 (4.4)
Missing	5986 (13.9)	18 (12.7)	7262 (17.1)	21 (16.2)	115 (14.3)

For peer review only - http://bmjopen.bmj.com/site/about/guidelines.xhtml

Supplemental figure 7. Association of potential risk factors with hospitalisation for Covid-19 (2020), influenza (2016-2019), and pneumonia (2016-2019) in type 1 diabetes, restricting hospitalisation definition to primary diagnosis in HES.

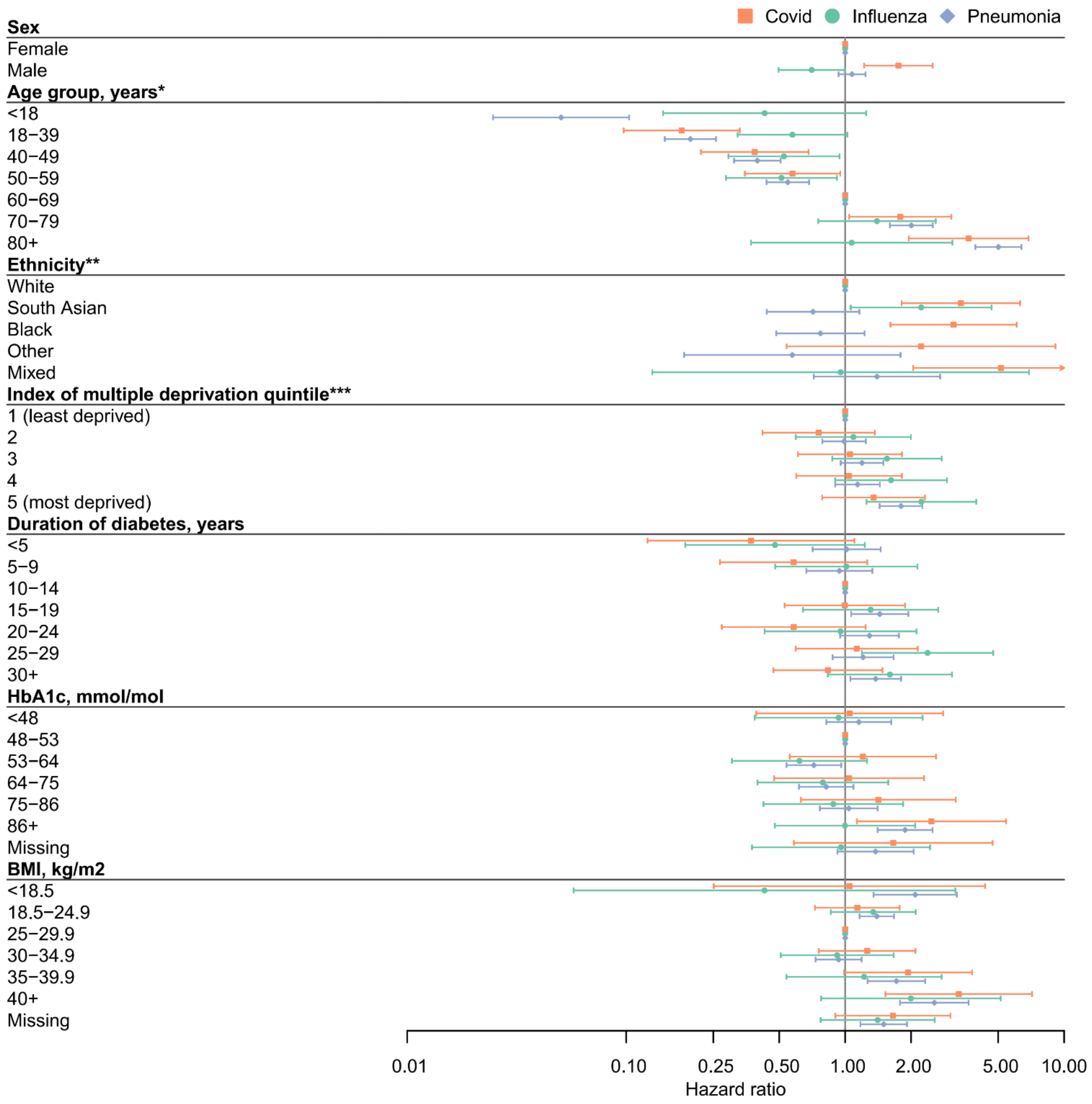

*No Covid-19 hospitalisations occurred in <18 group. **No influenza hospitalisation occurred in Black or other ethnicity groups. No Covid-19, influenza or pneumonia hospitalisation occurred in the unknown ethnicity group. *** No Covid-19, influenza or pneumonia hospitalisations occurred in missing IMD group.

For peer review only - http://bmjopen.bmj.com/site/about/guidelines.xhtml

Supplemental table 9. Baseline characteristics and Covid-19, influenza, and pneumonia hospitalisations in type 2 diabetes, restricting hospitalisation definition to primary diagnosis in HES.

	2020 cohort	Covid-19 hospitalisations (in 2020 cohort)	2016 cohort	Influenza hospitalisations (in 2016 cohort)	Pneumonia hospitalisations (in 2016 cohort)
**Number of individuals**	584854	4781	585289	2189	24944
**Mean (SD) follow-up, days**		265.3 (37.7)		915.7 (224.3)	903.4 (239.5)
**Sex**					
Female	253360 (43.3)	1802 (37.7)	256656 (43.9)	1068 (48.8)	11337 (45.4)
Male	331494 (56.7)	2979 (62.3)	328633 (56.1)	1121 (51.2)	13607 (54.6)
**Age group, years**					
<40	12898 (2.2)	61 (1.3)	13468 (2.3)	28 (1.3)	111 (0.4)
40-49	46075 (7.9)	220 (4.6)	49649 (8.5)	93 (4.2)	453 (1.8)
50-59	114537 (19.6)	625 (13.1)	113285 (19.4)	216 (9.9)	1599 (6.4)
60-69	151493 (25.9)	976 (20.4)	153782 (26.3)	456 (20.8)	3900 (15.6)
70-79	155424 (26.6)	1312 (27.4)	152401 (26.0)	732 (33.4)	7893 (31.6)
80-89	89871 (15.4)	1309 (27.4)	88492 (15.1)	559 (25.5)	8788 (35.2)
90+	14556 (2.5)	278 (5.8)	14212 (2.4)	105 (4.8)	2200 (8.8)
**Ethnicity**					
White	445160 (76.1)	3240 (67.8)	457714 (78.2)	1746 (79.8)	21923 (87.9)
South Asian	77253 (13.2)	766 (16.0)	71050 (12.1)	282 (12.9)	1871 (7.5)
Black	35144 (6.0)	575 (12.0)	33175 (5.7)	105 (4.8)	771 (3.1)
Other	9886 (1.7)	132 (2.8)	8178 (1.4)	25 (1.1)	190 (0.8)
Mixed	6267 (1.1)	56 (1.2)	5654 (1.0)	25 (1.1)	149 (0.6)
Unknown	11144 (1.9)	12 (0.3)	9518 (1.6)	6 (0.3)	40 (0.2)
**Index of multiple deprivation quintile**					
1 (least deprived)	102950 (17.6)	607 (12.7)	103184 (17.6)	332 (15.2)	4085 (16.4)
2	106968 (18.3)	715 (15.0)	109006 (18.6)	368 (16.8)	4559 (18.3)
3	113454 (19.4)	861 (18.0)	113661 (19.4)	422 (19.3)	4765 (19.1)
4	127048 (21.7)	1186 (24.8)	125492 (21.4)	477 (21.8)	5330 (21.4)
5 (most deprived)	134146 (22.9)	1410 (29.5)	133563 (22.8)	589 (26.9)	6190 (24.8)
Missing	288 (0.0)	2 (0.0)	383 (0.1)	1 (0.0)	15 (0.1)
**Duration of diagnosed diabetes, years**					
<1	14311 (2.4)	60 (1.3)	36503 (6.2)	98 (4.5)	953 (3.8)
1-2	60219 (10.3)	337 (7.0)	77510 (13.2)	216 (9.9)	2247 (9.0)
3-5	105094 (18.0)	652 (13.6)	111199 (19.0)	334 (15.3)	3429 (13.7)
6-9	128817 (22.0)	927 (19.4)	125083 (21.4)	428 (19.6)	4686 (18.8)
10-14	124471 (21.3)	967 (20.2)	126421 (21.6)	526 (24.0)	6378 (25.6)
15-19	89704 (15.3)	972 (20.3)	62373 (10.7)	302 (13.8)	3652 (14.6)
20+	62238 (10.6)	866 (18.1)	46200 (7.9)	285 (13.0)	3599 (14.4)
**HbA1c, mmol/mol**					
<48	167739 (28.7)	1327 (27.8)	175659 (30.0)	634 (29.0)	8049 (32.3)
48-53	116015 (19.8)	840 (17.6)	116131 (19.8)	392 (17.9)	4701 (18.8)
53-64	147209 (25.2)	1093 (22.9)	142662 (24.4)	532 (24.3)	5861 (23.5)
64-75	66071 (11.3)	618 (12.9)	62291 (10.6)	289 (13.2)	2606 (10.4)
75-86	33970 (5.8)	335 (7.0)	32257 (5.5)	127 (5.8)	1422 (5.7)
86+	39727 (6.8)	467 (9.8)	37470 (6.4)	165 (7.5)	1676 (6.7)
Missing	14123 (2.4)	101 (2.1)	18819 (3.2)	50 (2.3)	629 (2.5)
**Number of microvascular complications**					
0	264011 (45.1)	1550 (32.4)	281811 (48.1)	761 (34.8)	7915 (31.7)
1	216115 (37.0)	1718 (35.9)	204550 (34.9)	836 (38.2)	9536 (38.2)
2	96948 (16.6)	1261 (26.4)	91671 (15.7)	526 (24.0)	6617 (26.5)
3	7780 (1.3)	252 (5.3)	7257 (1.2)	66 (3.0)	876 (3.5)
**BMI, kg/m2**					
<18.5	3026 (0.5)	48 (1.0)	2801 (0.5)	12 (0.5)	362 (1.5)
18.5-24.9	86199 (14.7)	752 (15.7)	79018 (13.5)	310 (14.2)	4694 (18.8)
25-29.9	184626 (31.6)	1418 (29.7)	178501 (30.5)	614 (28.0)	7025 (28.2)
30-34.9	151732 (25.9)	1100 (23.0)	151161 (25.8)	551 (25.2)	5329 (21.4)
35-39.9	74766 (12.8)	612 (12.8)	75339 (12.9)	289 (13.2)	2674 (10.7)
40+	48455 (8.3)	441 (9.2)	48685 (8.3)	195 (8.9)	1857 (7.4)
Missing	36050 (6.2)	410 (8.6)	49784 (8.5)	218 (10.0)	3003 (12.0)
**Smoking status**					
Active smoker	87220 (14.9)	403 (8.4)	90218 (15.4)	364 (16.6)	4168 (16.7)
Ex-smoker	336719 (57.6)	3124 (65.3)	316723 (54.1)	1266 (57.8)	14903 (59.7)
Non-smoker	138840 (23.7)	1035 (21.6)	149524 (25.5)	436 (19.9)	4558 (18.3)
Unknown	22075 (3.8)	219 (4.6)	28824 (4.9)	123 (5.6)	1315 (5.3)
**Comorbidities**					
**Cardiovascular**					
Hypertension	425278 (72.7)	4017 (84.0)	419645 (71.7)	1818 (83.1)	21575 (86.5)
Atrial fibrillation	70633 (12.1)	1078 (22.5)	63872 (10.9)	473 (21.6)	6940 (27.8)
Angina	87970 (15.0)	1215 (25.4)	91026 (15.6)	596 (27.2)	7284 (29.2)
Previous myocardial infarction	63738 (10.9)	983 (20.6)	60576 (10.3)	422 (19.3)	5472 (21.9)
Previous cardiac revascularisation	57332 (9.8)	742 (15.5)	54961 (9.4)	317 (14.5)	3853 (15.4)
Other ischaemic heart disease	121776 (20.8)	1669 (34.9)	121753 (20.8)	766 (35.0)	9695 (38.9)

For peer review only - http://bmjopen.bmj.com/site/about/guidelines.xhtml

	2020 cohort	Covid-19 hospitalisations (in 2020 cohort)	2016 cohort	Influenza hospitalisations (in 2016 cohort)	Pneumonia hospitalisations (in 2016 cohort)
Heart failure	61014 (10.4)	1243 (26.0)	53579 (9.2)	469 (21.4)	6932 (27.8)
Peripheral arterial disease	50045 (8.6)	895 (18.7)	48489 (8.3)	301 (13.8)	4970 (19.9)
**Recent hospitalisation**					
Respiratory infection	25535 (4.4)	817 (17.1)	21353 (3.6)	280 (12.8)	4469 (17.9)
Anything else	96421 (16.5)	1890 (39.5)	95232 (16.3)	794 (36.3)	10039 (40.2)
**Respiratory**					
Asthma	125582 (21.5)	1275 (26.7)	118207 (20.2)	701 (32.0)	7866 (31.5)
Chronic obstructive pulmonary disease	65615 (11.2)	1022 (21.4)	60108 (10.3)	534 (24.4)	8088 (32.4)
**Neurological**					
Previous transient ischaemic attack	31024 (5.3)	463 (9.7)	29997 (5.1)	203 (9.3)	2812 (11.3)
Previous stroke	49873 (8.5)	763 (16.0)	47721 (8.2)	341 (15.6)	4574 (18.3)
Dementia	20629 (3.5)	573 (12.0)	19548 (3.3)	149 (6.8)	2611 (10.5)
Other neurological condition	35000 (6.0)	584 (12.2)	33569 (5.7)	240 (11.0)	3309 (13.3)
**Oncological**					
Haematological cancer	11751 (2.0)	162 (3.4)	10352 (1.8)	97 (4.4)	990 (4.0)
Solid cancer	79179 (13.5)	855 (17.9)	72001 (12.3)	341 (15.6)	5206 (20.9)
**Other**					
Solid organ transplant	3682 (0.6)	82 (1.7)	3013 (0.5)	32 (1.5)	332 (1.3)
Chronic liver disease	69136 (11.8)	670 (14.0)	48011 (8.2)	210 (9.6)	2142 (8.6)
**Chronic Kidney Disease (CKD) stage**					
Stage 1	165845 (28.4)	790 (16.5)	147798 (25.3)	299 (13.7)	2502 (10.0)
Stage 2	286696 (49.0)	1940 (40.6)	290821 (49.7)	971 (44.4)	10121 (40.6)
Stage 3a	72539 (12.4)	841 (17.6)	80222 (13.7)	453 (20.7)	5273 (21.1)
Stage 3b	33246 (5.7)	544 (11.4)	37256 (6.4)	237 (10.8)	3818 (15.3)
Stage 4	9006 (1.5)	230 (4.8)	10490 (1.8)	88 (4.0)	1473 (5.9)
Stage 5	12871 (2.2)	421 (8.8)	13365 (2.3)	130 (5.9)	1629 (6.5)
Missing	4651 (0.8)	15 (0.3)	5337 (0.9)	11 (0.5)	128 (0.5)
**Albumin creatinine ratio (ACR) category**					
A1	402847 (68.9)	2655 (55.5)	388839 (66.4)	1252 (57.2)	13184 (52.9)
A2	121002 (20.7)	1339 (28.0)	106879 (18.3)	565 (25.8)	7450 (29.9)
A3	21899 (3.7)	495 (10.4)	18817 (3.2)	153 (7.0)	2033 (8.2)
Missing	39106 (6.7)	292 (6.1)	70754 (12.1)	219 (10.0)	2277 (9.1)
**Diabetes treatment (last 6 months)**					
Insulin prescription	79286 (13.6)	1190 (24.9)	78755 (13.5)	468 (21.4)	5380 (21.6)
OHA prescription only	362651 (62.0)	2584 (54.0)	349629 (59.7)	1215 (55.5)	12993 (52.1)
No treatment	142917 (24.4)	1007 (21.1)	156905 (26.8)	506 (23.1)	6571 (26.3)

For peer review only
For peer review only - http://bmjopen.bmj.com/site/about/guidelines.xhtml

Supplemental figure 8. Association of potential risk factors with hospitalisation for Covid-19 (2020), influenza (2016-2019), and pneumonia (2016-2019) in type 2 diabetes, restricting hospitalisation definition to primary diagnosis in HES.

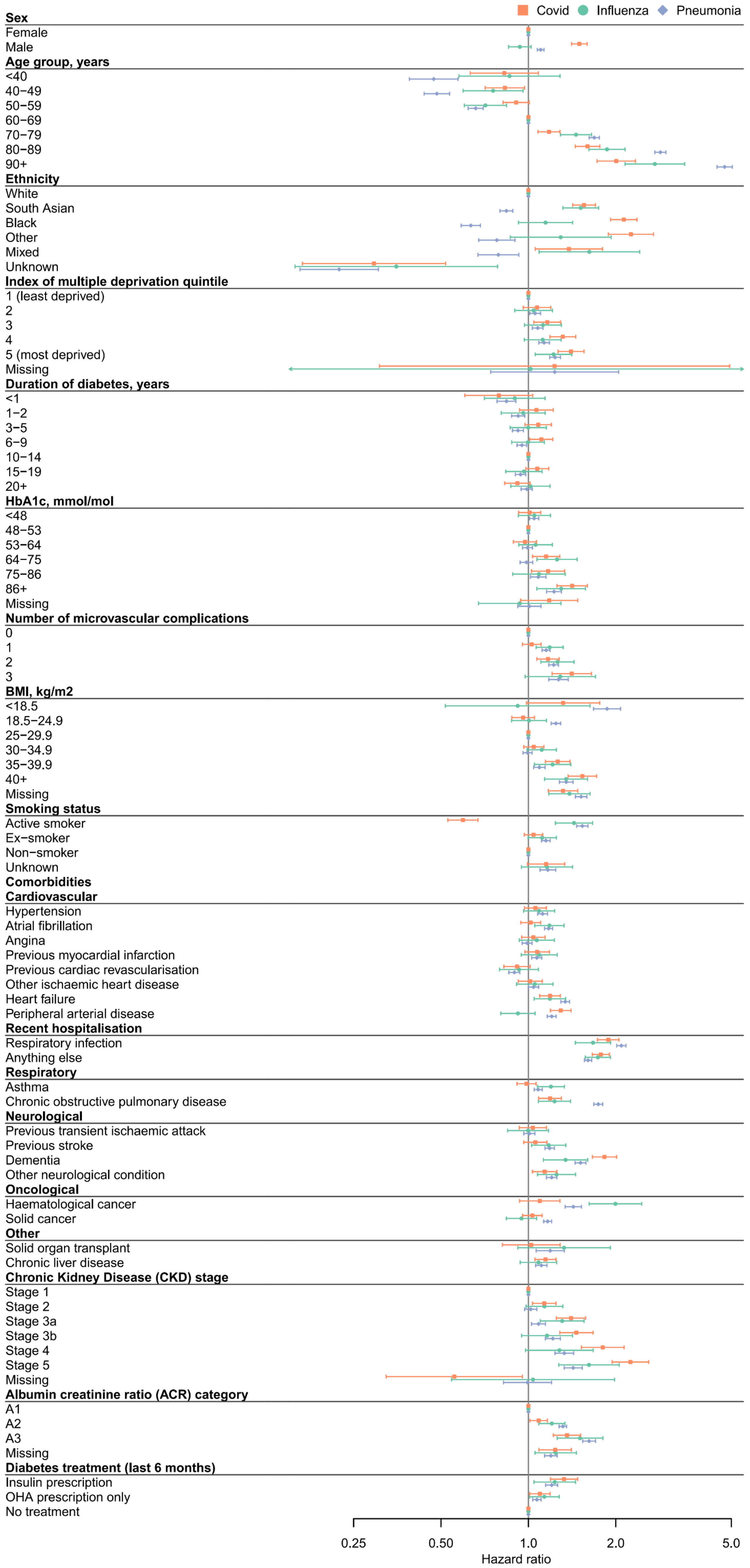

For peer review only - http://bmjopen.bmj.com/site/about/guidelines.xhtml

Supplemental table 10. Baseline characteristics and Covid-19 and pneumonia deaths in type 2 diabetes, restricting mortality definition to primary cause in ONS.

	2020 cohort	Covid-19 deaths (in 2020 cohort)	2016 cohort	Pneumonia deaths (in 2016 cohort)
**Number of individuals**	584854	2086	585289	2653
**Mean (SD) follow-up, days**		266.1 (35.5)		917.0 (223.2)
**Sex**				
Female	253360 (43.3)	796 (38.2)	256656 (43.9)	1268 (47.8)
Male	331494 (56.7)	1290 (61.8)	328633 (56.1)	1385 (52.2)
**Age group, years**				
<40	12898 (2.2)	6 (0.3)	13468 (2.3)	1 (0.0)
40-49	46075 (7.9)	30 (1.4)	49649 (8.5)	23 (0.9)
50-59	114537 (19.6)	118 (5.7)	113285 (19.4)	52 (2.0)
60-69	151493 (25.9)	299 (14.3)	153782 (26.3)	199 (7.5)
70-79	155424 (26.6)	563 (27.0)	152401 (26.0)	566 (21.3)
80-89	89871 (15.4)	847 (40.6)	88492 (15.1)	1227 (46.2)
90+	14556 (2.5)	223 (10.7)	14212 (2.4)	585 (22.1)
**Ethnicity**				
White	445160 (76.1)	1514 (72.6)	457714 (78.2)	2399 (90.4)
South Asian	77253 (13.2)	289 (13.9)	71050 (12.1)	160 (6.0)
Black	35144 (6.0)	216 (10.4)	33175 (5.7)	55 (2.1)
Other	9886 (1.7)	42 (2.0)	8178 (1.4)	20 (0.8)
Mixed	6267 (1.1)	18 (0.9)	5654 (1.0)	10 (0.4)
Unknown	11144 (1.9)	7 (0.3)	9518 (1.6)	9 (0.3)
**Index of multiple deprivation quintile**				
1 (least deprived)	102950 (17.6)	281 (13.5)	103184 (17.6)	493 (18.6)
2	106968 (18.3)	330 (15.8)	109006 (18.6)	490 (18.5)
3	113454 (19.4)	397 (19.0)	113661 (19.4)	525 (19.8)
4	127048 (21.7)	475 (22.8)	125492 (21.4)	550 (20.7)
5 (most deprived)	134146 (22.9)	602 (28.9)	133563 (22.8)	595 (22.4)
Missing	288 (0.0)	1 (0.0)	383 (0.1)	0 (0.0)
**Duration of diagnosed diabetes, years**				
<1	14311 (2.4)	10 (0.5)	36503 (6.2)	53 (2.0)
1-2	60219 (10.3)	93 (4.5)	77510 (13.2)	157 (5.9)
3-5	105094 (18.0)	257 (12.3)	111199 (19.0)	267 (10.1)
6-9	128817 (22.0)	343 (16.4)	125083 (21.4)	452 (17.0)
10-14	124471 (21.3)	443 (21.2)	126421 (21.6)	732 (27.6)
15-19	89704 (15.3)	490 (23.5)	62373 (10.7)	498 (18.8)
20+	62238 (10.6)	450 (21.6)	46200 (7.9)	494 (18.6)
**HbA1c, mmol/mol**				
<48	167739 (28.7)	655 (31.4)	175659 (30.0)	913 (34.4)
48-53	116015 (19.8)	351 (16.8)	116131 (19.8)	498 (18.8)
53-64	147209 (25.2)	462 (22.1)	142662 (24.4)	612 (23.1)
64-75	66071 (11.3)	266 (12.8)	62291 (10.6)	259 (9.8)
75-86	33970 (5.8)	134 (6.4)	32257 (5.5)	135 (5.1)
86+	39727 (6.8)	156 (7.5)	37470 (6.4)	150 (5.7)
Missing	14123 (2.4)	62 (3.0)	18819 (3.2)	86 (3.2)
**Number of microvascular complications**				
0	264011 (45.1)	564 (27.0)	281811 (48.1)	641 (24.2)
1	216115 (37.0)	772 (37.0)	204550 (34.9)	1009 (38.0)
2	96948 (16.6)	629 (30.2)	91671 (15.7)	884 (33.3)
3	7780 (1.3)	121 (5.8)	7257 (1.2)	119 (4.5)
**BMI, kg/m2**				
<18.5	3026 (0.5)	36 (1.7)	2801 (0.5)	55 (2.1)
18.5-24.9	86199 (14.7)	466 (22.3)	79018 (13.5)	664 (25.0)
25-29.9	184626 (31.6)	597 (28.6)	178501 (30.5)	726 (27.4)
30-34.9	151732 (25.9)	418 (20.0)	151161 (25.8)	395 (14.9)
35-39.9	74766 (12.8)	197 (9.4)	75339 (12.9)	183 (6.9)
40+	48455 (8.3)	125 (6.0)	48685 (8.3)	138 (5.2)
Missing	36050 (6.2)	247 (11.8)	49784 (8.5)	492 (18.5)
**Smoking status**				
Active smoker	87220 (14.9)	179 (8.6)	90218 (15.4)	320 (12.1)
Ex-smoker	336719 (57.6)	1377 (66.0)	316723 (54.1)	1566 (59.0)
Non-smoker	138840 (23.7)	441 (21.1)	149524 (25.5)	630 (23.7)
Unknown	22075 (3.8)	89 (4.3)	28824 (4.9)	137 (5.2)
**Comorbidities**				
**Cardiovascular**				
Hypertension	425278 (72.7)	1853 (88.8)	419645 (71.7)	2402 (90.5)
Atrial fibrillation	70633 (12.1)	612 (29.3)	63872 (10.9)	946 (35.7)
Angina	87970 (15.0)	599 (28.7)	91026 (15.6)	812 (30.6)
Previous myocardial infarction	63738 (10.9)	497 (23.8)	60576 (10.3)	650 (24.5)
Previous cardiac revascularisation	57332 (9.8)	363 (17.4)	54961 (9.4)	435 (16.4)
Other ischaemic heart disease	121776 (20.8)	799 (38.3)	121753 (20.8)	1099 (41.4)
Heart failure	61014 (10.4)	658 (31.5)	53579 (9.2)	965 (36.4)

For peer review only

	2020 cohort	Covid-19 deaths (in 2020 cohort)	2016 cohort	Pneumonia deaths (in 2016 cohort)
Peripheral arterial disease	50045 (8.6)	492 (23.6)	48489 (8.3)	693 (26.1)
**Recent hospitalisation**				
Respiratory infection	25535 (4.4)	439 (21.0)	21353 (3.6)	483 (18.2)
Anything else	96421 (16.5)	911 (43.7)	95232 (16.3)	1117 (42.1)
**Respiratory**				
Asthma	125582 (21.5)	543 (26.0)	118207 (20.2)	507 (19.1)
Chronic obstructive pulmonary disease	65615 (11.2)	502 (24.1)	60108 (10.3)	458 (17.3)
**Neurological**				
Previous transient ischaemic attack	31024 (5.3)	265 (12.7)	29997 (5.1)	325 (12.3)
Previous stroke	49873 (8.5)	457 (21.9)	47721 (8.2)	476 (17.9)
Dementia	20629 (3.5)	477 (22.9)	19548 (3.3)	274 (10.3)
Other neurological condition	35000 (6.0)	362 (17.4)	33569 (5.7)	395 (14.9)
**Oncological**				
Haematological cancer	11751 (2.0)	89 (4.3)	10352 (1.8)	62 (2.3)
Solid cancer	79179 (13.5)	412 (19.8)	72001 (12.3)	488 (18.4)
**Other**				
Solid organ transplant	3682 (0.6)	27 (1.3)	3013 (0.5)	47 (1.8)
Chronic liver disease	69136 (11.8)	261 (12.5)	48011 (8.2)	140 (5.3)
**Chronic Kidney Disease (CKD) stage**				
Stage 1	165845 (28.4)	191 (9.2)	147798 (25.3)	128 (4.8)
Stage 2	286696 (49.0)	760 (36.4)	290821 (49.7)	791 (29.8)
Stage 3a	72539 (12.4)	445 (21.3)	80222 (13.7)	595 (22.4)
Stage 3b	33246 (5.7)	336 (16.1)	37256 (6.4)	561 (21.1)
Stage 4	9006 (1.5)	147 (7.0)	10490 (1.8)	295 (11.1)
Stage 5	12871 (2.2)	195 (9.3)	13365 (2.3)	260 (9.8)
Missing	4651 (0.8)	12 (0.6)	5337 (0.9)	23 (0.9)
**Albumin creatinine ratio (ACR) category**				
A1	402847 (68.9)	1013 (48.6)	388839 (66.4)	1197 (45.1)
A2	121002 (20.7)	647 (31.0)	106879 (18.3)	961 (36.2)
A3	21899 (3.7)	251 (12.0)	18817 (3.2)	314 (11.8)
Missing	39106 (6.7)	175 (8.4)	70754 (12.1)	181 (6.8)
**Diabetes treatment (last 6 months)**				
Insulin prescription	79286 (13.6)	536 (25.7)	78755 (13.5)	581 (21.9)
OHA prescription only	362651 (62.0)	1012 (48.5)	349629 (59.7)	1307 (49.3)
No treatment	142917 (24.4)	538 (25.8)	156905 (26.8)	765 (28.8)

For peer review only

Supplemental figure 9. Association of potential risk factors with Covid-19 (2020), and pneumonia (2016-2019) mortality in type 2 diabetes, restricting mortality definition to primary cause in ONS.

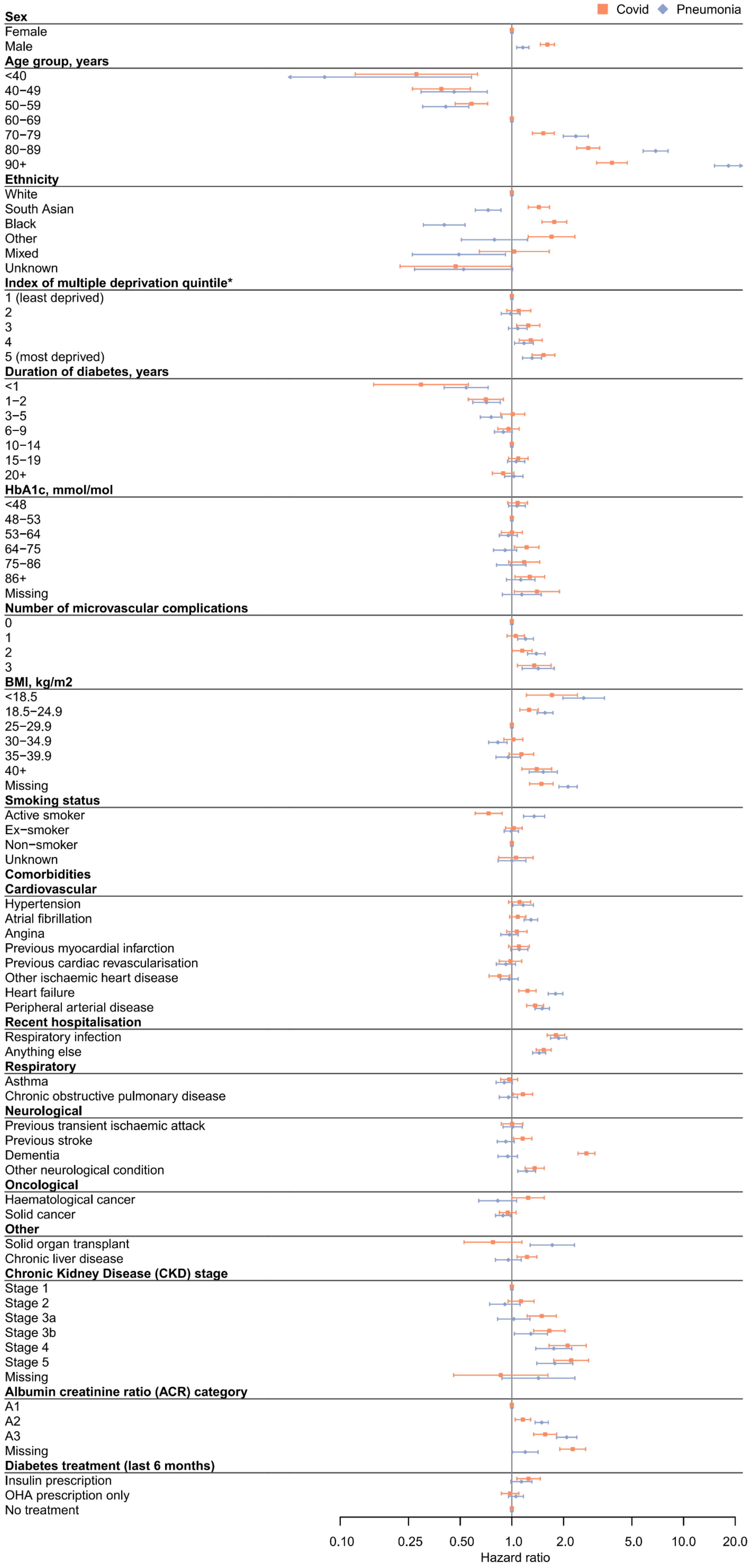

* No pneumonia deaths occurred in missing IMD group. 1 Covid-19 death occurred in missing IMD group.

For peer review only - http://bmjopen.bmj.com/site/about/guidelines.xhtml

Supplemental figure 10. Association of potential risk factors with pneumonia hospitalisation (2016-2019) in those with a pneumococcal vaccination and those without.

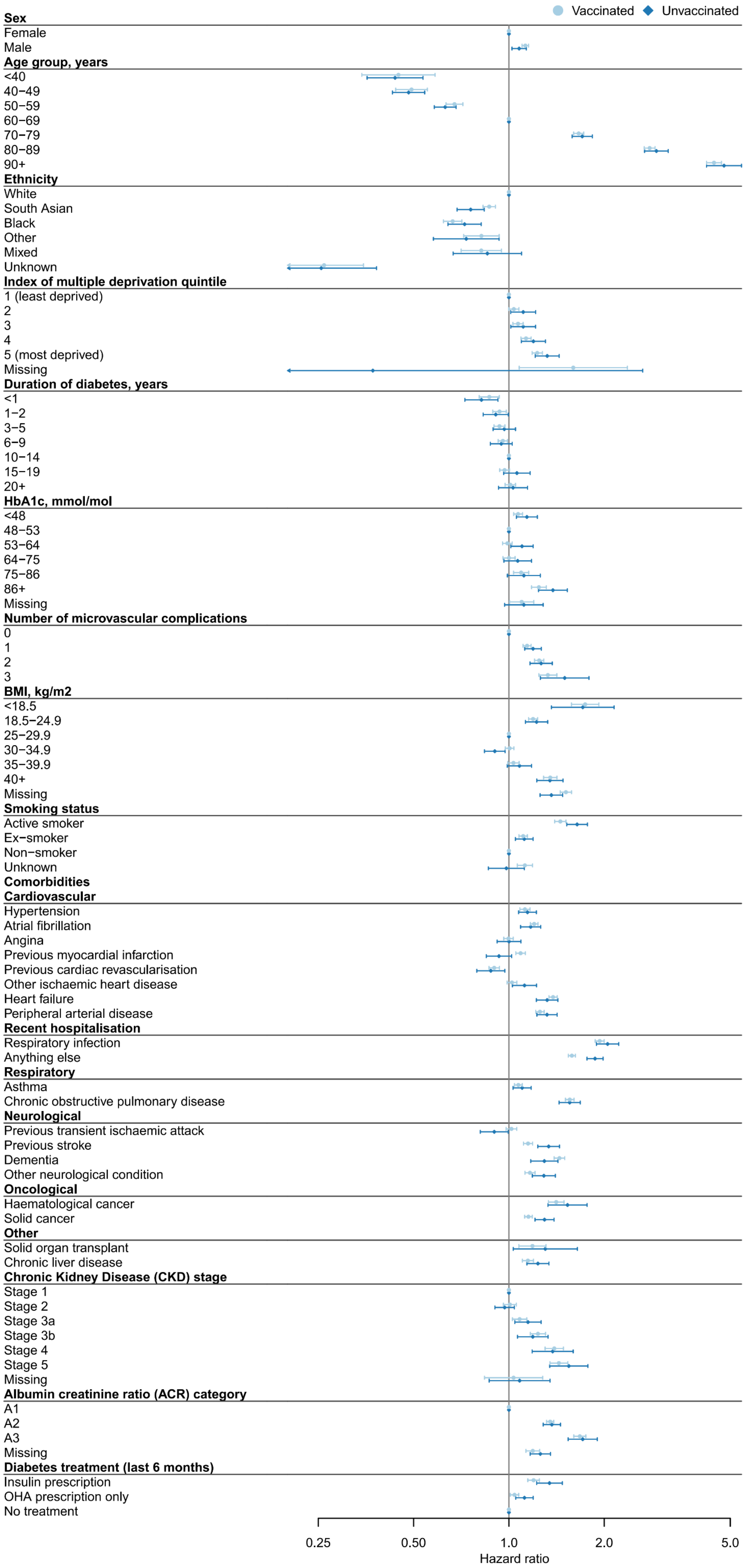

26

Supplemental figure 11. Association of potential risk factors with influenza hospitalisation (2016-2019) in those with an influenza vaccination and those without.

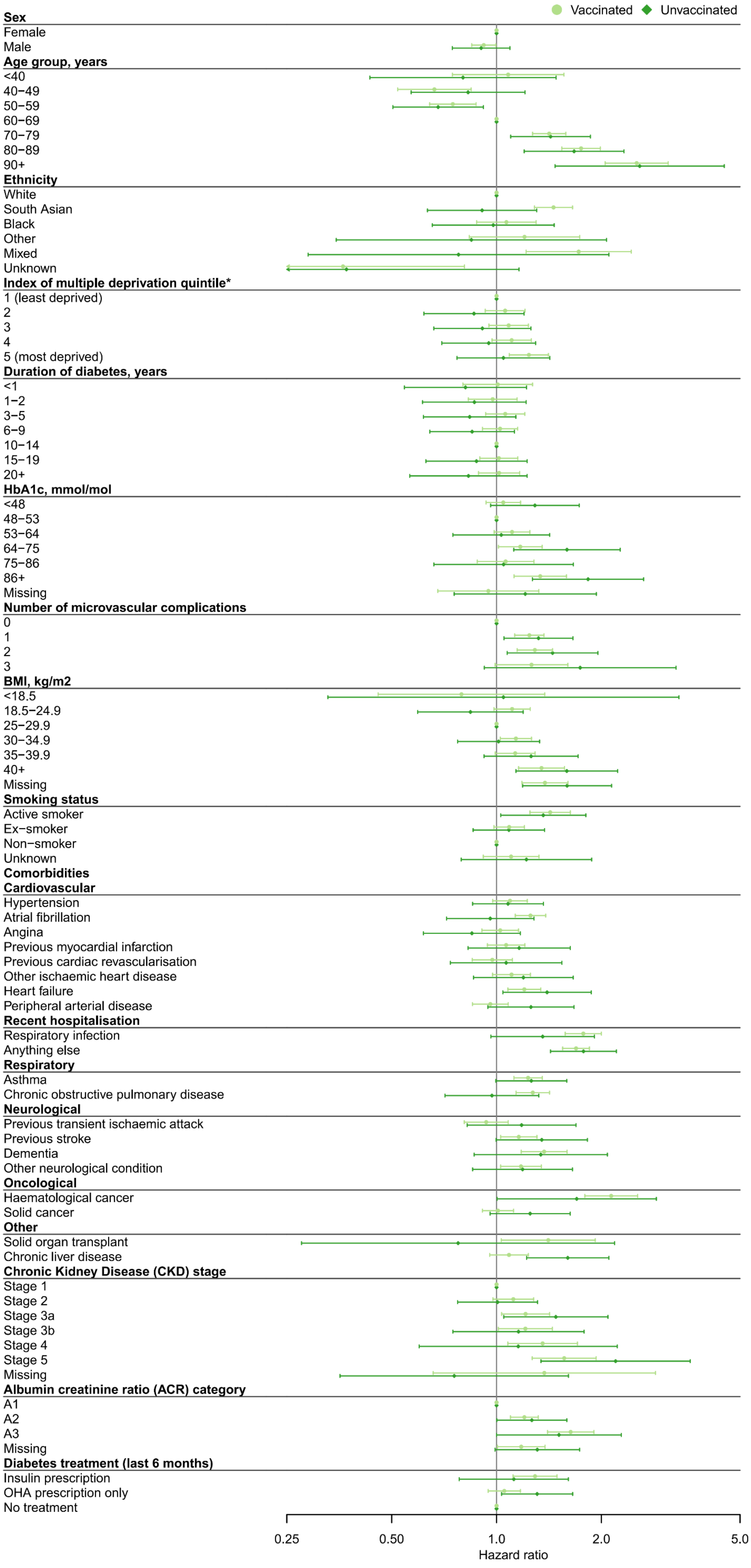

* No influenza hospitalisations occurred in the missing IMD group.

References

1. Mathur R, Bhaskaran K, Chaturvedi N, et al. Completeness and usability of ethnicity data in UK-based primary care and hospital databases. J Public Health (Oxf) 2014;36(4): 684-92. doi: 10.1093/pubmed/fdt116

2. GOV.UK. English indices of deprivation 2015. Available: https://www.gov.uk/government/statistics/english-indices-of-deprivation-2015 [Accessed 4 January 2023]

3. World Health Organisation. A healthy lifestyle - WHO recommendations. 2010. Available from https://www.who.int/europe/news-room/fact-sheets/item/a-healthy-lifestyle---who-recommendations [Accessed 4 January 2023]

4. Rodgers LR, Weedon MN, Henley WE, et al. Cohort profile for the MASTERMIND study: using the Clinical Practice Research Datalink (CPRD) to investigate stratification of response to treatment in patients with type 2 diabetes. BMJ Open 2017;7(10): e017989. doi: 10.1136/bmjopen-2017-017989

5. KDIGO. CKD Evaluation and Management. 2012. Available from https://kdigo.org/guidelines/ckd-evaluation-and-management/ [Accessed 4 January 2023]

6. Clift AK, Coupland CAC, Keogh RH, et al. Living risk prediction algorithm (QCOVID) for risk of hospital admission and mortality from coronavirus 19 in adults: national derivation and validation cohort study. BMJ 2020;371: m3731. doi: 10.1136/bmj.m3731

For peer review only

For peer review only - http://bmjopen.bmj.com/site/about/guidelines.xhtml

The RECORD statement – checklist of items, extended from the STROBE statement, that should be reported in observational studies using routinely collected health data.

	Item No.	STROBE items	Location in manuscript where items are reported	RECORD items	Location in manuscript where items are reported
**Title and abstract**					
	1	(a) Indicate the study's design with a commonly used term in the title or the abstract (b) Provide in the abstract an informative and balanced summary of what was done and what was found	a) Study design given in the title and abstract methods section.<br>b) Summary of the study given in abstract methods section.	RECORD 1.1: The type of data used should be specified in the title or abstract. When possible, the name of the databases used should be included.<br><br>RECORD 1.2: If applicable, the geographic region and timeframe within which the study took place should be reported in the title or abstract.<br><br>RECORD 1.3: If linkage between databases was conducted for the study, this should be clearly stated in the title or abstract.	Type of data and dataset name given in abstract methods section.<br><br>Study periods given in abstract methods section.<br><br>Linkage stated in abstract methods section.
**Introduction**					
Background rationale	2	Explain the scientific background and rationale for the investigation being reported	Background and rationale are covered in the introduction.		
Objectives	3	State specific objectives, including any prespecified hypotheses	Aims are stated at the end of introduction.		
**Methods**					
Study Design	4	Present key elements of study design early in the paper	Study design is reported in the methods.		
Setting	5	Describe the setting, locations, and relevant dates, including periods of recruitment, exposure, follow-up, and data collection	Data source and study population sections of methods cover the		

			setting/location and periods of follow-up.		
Participants	6	*(a) Cohort study* - Give the eligibility criteria, and the sources and methods of selection of participants. Describe methods of follow-up<br>*Case-control study* - Give the eligibility criteria, and the sources and methods of case ascertainment and control selection. Give the rationale for the choice of cases and controls<br>*Cross-sectional study* - Give the eligibility criteria, and the sources and methods of selection of participants<br><br>*(b) Cohort study* - For matched studies, give matching criteria and number of exposed and unexposed<br>*Case-control study* - For matched studies, give matching criteria and the number of controls per case	a) Study population section of methods gives inclusion criteria for participants (flow diagram included as Supplemental figure 1) and follow-up described.	RECORD 6.1: The methods of study population selection (such as codes or algorithms used to identify subjects) should be listed in detail. If this is not possible, an explanation should be provided.<br><br>RECORD 6.2: Any validation studies of the codes or algorithms used to select the population should be referenced. If validation was conducted for this study and not published elsewhere, detailed methods and results should be provided.<br><br>RECORD 6.3: If the study involved linkage of databases, consider use of a flow diagram or other graphical display to demonstrate the data linkage process, including the number of individuals with linked data at each stage.	Algorithm for selection of diabetes population briefly described in study population section of methods and full details available on our github (https://github.com/Exeter-Diabetes/CPRD-Codelists).<br><br>Study confirming accuracy of algorithm referenced.<br><br>CPRD performed data linkage, number of participants with linked data given in flow diagram (Supplemental figure 1)
Variables	7	Clearly define all outcomes, exposures, predictors, potential confounders, and effect modifiers. Give diagnostic criteria, if applicable.	All outcomes defined in outcomes section of methods. All exposures defined in risk factors and covariates section	RECORD 7.1: A complete list of codes and algorithms used to classify exposures, outcomes, confounders, and effect modifiers should be provided. If these cannot be reported, an explanation should be provided.	Codes and algorithms used for all exposures can be found on our github (https://github.com/Exeter-Diabetes/CPRD-

For peer review only

			and Supplemental table 2.		Codelists), codes used to define outcomes in Supplemental table 1.
Data sources/ measurement	8	For each variable of interest, give sources of data and details of methods of assessment (measurement).<br>Describe comparability of assessment methods if there is more than one group	Sources of data and measurement of variables given in Supplemental table 2.		
Bias	9	Describe any efforts to address potential sources of bias	Covered in methods.		
Study size	10	Explain how the study size was arrived at	Not applicable.		
Quantitative variables	11	Explain how quantitative variables were handled in the analyses. If applicable, describe which groupings were chosen, and why	Supplemental table 2 describes how quantitative variables were handled/ grouped.		
Statistical methods	12	(a) Describe all statistical methods, including those used to control for confounding<br>(b) Describe any methods used to examine subgroups and interactions<br>(c) Explain how missing data were addressed<br>(d) *Cohort study* - If applicable, explain how loss to follow-up was addressed<br>*Case-control study* - If applicable, explain how matching of cases and controls was addressed<br>*Cross-sectional study* - If applicable, describe analytical	Statistical analysis section of methods describes all statistical methods used and methods used for subgroup analysis and sensitivity analysis. Missing data methods described in methods and Supplemental table 2.		

For peer review only

		methods taking account of sampling strategy<br>(e) Describe any sensitivity analyses			
Data access and cleaning methods		..		RECORD 12.1: Authors should describe the extent to which the investigators had access to the database population used to create the study population.<br><br>RECORD 12.2: Authors should provide information on the data cleaning methods used in the study.	Some authors had access to raw data (stated in acknowledgments)<br><br>Algorithms used for cleaning and deriving variables given on github
Linkage		..		RECORD 12.3: State whether the study included person-level, institutional-level, or other data linkage across two or more databases. The methods of linkage and methods of linkage quality evaluation should be provided.	CPRD performed linkage of datasets.
**Results**					
Participants	13	(a) Report the numbers of individuals at each stage of the study (*e.g.*, numbers potentially eligible, examined for eligibility, confirmed eligible, included in the study, completing follow-up, and analysed)<br>(b) Give reasons for non-participation at each stage.<br>(c) Consider use of a flow diagram	Study participation flow diagram given as Supplemental figure 1.	RECORD 13.1: Describe in detail the selection of the persons included in the study (*i.e.,* study population selection) including filtering based on data quality, data availability and linkage. The selection of included persons can be described in the text and/or by means of the study flow diagram.	Study flow diagram (Supplemental figure 1) gives detailed summary of selection of participants for study.
Descriptive data	14	(a) Give characteristics of study participants (*e.g.*, demographic, clinical, social) and information on exposures and potential confounders	Baseline characteristics of study participants and mean follow-up time given in Table		

For peer review only

		(b) Indicate the number of participants with missing data for each variable of interest<br>(c) *Cohort study* - summarise follow-up time (*e.g.*, average and total amount)	1 and Supplemental table 4. Missing data category included for each variable where applicable.		
Outcome data	15	*Cohort study* - Report numbers of outcome events or summary measures over time<br>*Case-control study* - Report numbers in each exposure category, or summary measures of exposure<br>*Cross-sectional study* - Report numbers of outcome events or summary measures	Numbers of outcome events reported in results section of text and in Table 1.		
Main results	16	(a) Give unadjusted estimates and, if applicable, confounder-adjusted estimates and their precision (e.g., 95% confidence interval). Make clear which confounders were adjusted for and why they were included<br>(b) Report category boundaries when continuous variables were categorized<br>(c) If relevant, consider translating estimates of relative risk into absolute risk for a meaningful time period	Confounder adjusted estimates with 95% confidence intervals for all risk factors given in Supplemental table 5. Category boundaries given in all relevant tables and plots.		
Other analyses	17	Report other analyses done—e.g., analyses of subgroups and interactions, and sensitivity analyses	Results from subgroup analysis and sensitivity analyses reported.		
**Discussion**					
Key results	18	Summarise key results with reference to study objectives	Key results are summarised at the		

For peer review only

			beginning of the discussion section.		
Limitations	19	Discuss limitations of the study, taking into account sources of potential bias or imprecision. Discuss both direction and magnitude of any potential bias	Limitations of this study were discussed in the strengths and limitations section of the discussion.	RECORD 19.1: Discuss the implications of using data that were not created or collected to answer the specific research question(s). Include discussion of misclassification bias, unmeasured confounding, missing data, and changing eligibility over time, as they pertain to the study being reported.	Implications of using this data discussed in limitations.
Interpretation	20	Give a cautious overall interpretation of results considering objectives, limitations, multiplicity of analyses, results from similar studies, and other relevant evidence	Interpretation of results is given in the clinical implications section.		
Generalisability	21	Discuss the generalisability (external validity) of the study results	Study data largely representative of UK population.		
**Other Information**					
Funding	22	Give the source of funding and the role of the funders for the present study and, if applicable, for the original study on which the present article is based	Details of funding given in acknowledgments.		
Accessibility of protocol, raw data, and programming code		..		RECORD 22.1: Authors should provide information on how to access any supplemental information such as the study protocol, raw data, or programming code.	'CPRD data are available by application to CPRD Independent Scientific Advisory Committee' Code will be available on github.

For peer review only

*Reference: Benchimol EI, Smeeth L, Guttmann A, Harron K, Moher D, Petersen I, Sørensen HT, von Elm E, Langan SM, the RECORD Working Committee. The REporting of studies Conducted using Observational Routinely-collected health Data (RECORD) Statement. *PLoS Medicine* 2015; in press.

*Checklist is protected under Creative Commons Attribution (CC BY) license.

For peer review only

BMJ Open

Risk factor associations for severe Covid-19, influenza, and pneumonia in people with diabetes to inform future pandemic preparations: UK population-based cohort study

Journal:	*BMJ Open*
Manuscript ID	bmjopen-2023-078135.R1
Article Type:	Original research
Date Submitted by the Author:	11-Dec-2023
Complete List of Authors:	Hopkins, Rhian; University of Exeter Medical School, Institute of Biomedical & Clinical Science<br>Young, Katherine; University of Exeter Medical School, Institute of Biomedical & Clinical Science<br>Thomas, Nick; University of Exeter Medical School, Institute of Biomedical & Clinical Science<br>Godwin, James; University of Exeter Medical School, Institute of Biomedical & Clinical Science<br>Raja, Daniyal; University of Exeter Medical School, Institute of Biomedical & Clinical Science<br>Mateen, Bilal; The Alan Turing Institute; University College London Institute of Health Informatics<br>Challen, Robert; University of Bristol, Engineering Mathematics; NIHR Applied Research Collaboration South West Peninsula<br>Vollmer, Sebastian; University of Warwick Department of Statistics<br>Shields, Beverley; University of Exeter Medical School, Institute of Biomedical & Clinical Science<br>McGovern, Andrew; University of Exeter Medical School, Institute of Biomedical & Clinical Science<br>Dennis, John; University of Exeter Medical School, Institute of Biomedical & Clinical Science
**Primary Subject Heading**:	Diabetes and endocrinology
Secondary Subject Heading:	Epidemiology
Keywords:	COVID-19, Risk Factors, Electronic Health Records, DIABETES & ENDOCRINOLOGY

SCHOLARONE™
Manuscripts

I, the Submitting Author has the right to grant and does grant on behalf of all authors of the Work (as defined in the below author licence), an exclusive licence and/or a non-exclusive licence for contributions from authors who are: i) UK Crown employees; ii) where BMJ has agreed a CC-BY licence shall apply, and/or iii) in accordance with the terms applicable for US Federal Government officers or employees acting as part of their official duties; on a worldwide, perpetual, irrevocable, royalty-free basis to BMJ Publishing Group Ltd ("BMJ") its licensees and where the relevant Journal is co-owned by BMJ to the co-owners of the Journal, to publish the Work in this journal and any other BMJ products and to exploit all rights, as set out in our licence.

The Submitting Author accepts and understands that any supply made under these terms is made by BMJ to the Submitting Author unless you are acting as an employee on behalf of your employer or a postgraduate student of an affiliated institution which is paying any applicable article publishing charge ("APC") for Open Access articles. Where the Submitting Author wishes to make the Work available on an Open Access basis (and intends to pay the relevant APC), the terms of reuse of such Open Access shall be governed by a Creative Commons licence – details of these licences and which Creative Commons licence will apply to this Work are set out in our licence referred to above.

Other than as permitted in any relevant BMJ Author's Self Archiving Policies, I confirm this Work has not been accepted for publication elsewhere, is not being considered for publication elsewhere and does not duplicate material already published. I confirm all authors consent to publication of this Work and authorise the granting of this licence.

For peer review only

Risk factor associations for severe Covid-19, influenza, and pneumonia in people with diabetes to inform future pandemic preparations: UK population-based cohort study

Word count: 3856

Authors: Rhian Hopkins[1], Katherine G Young[1], Nicholas J Thomas[1], James Godwin[1], Daniyal Raja[1], Bilal A Mateen[2,3], Robert J Challen[4,5], Sebastian J Vollmer[6], Beverley M Shields[1], Andrew P McGovern[1]*, John M Dennis[1]*

*Joint senior

[1]University of Exeter Medical School. Address: Institute of Biomedical & Clinical Science, RILD Building, Royal Devon & Exeter Hospital, Barrack Road, Exeter EX2 5DW, UK

[2]The Alan Turing Institute. Address: British Library, 96 Euston Road, London, NW1 2DB, UK

[3]University College London, Institute of Health Informatics. Address: University College London, 222 Euston Rd, London, NW1 2DA, London, UK

[4]Engineering Mathematics, University of Bristol. Address: Department of Engineering and Mathematics, University of Bristol, Bristol, BS8 1TW, UK

[5]University of Exeter Medical School and the National Institute for Health Research (NIHR) Applied Research Collaboration South West Peninsula (PenARC). Address: University of Exeter, Exeter, EX1 2LU, UK

[6]University of Warwick, Department of Statistics. Address: Department of Statistics, University of Warwick, Coventry, CV4 7AL, UK

Corresponding Author:

Rhian Hopkins

rh530@exeter.ac.uk

For peer review only - http://bmjopen.bmj.com/site/about/guidelines.xhtml

Abstract

Objective This study aimed to compare clinical and sociodemographic risk factors for severe Covid-19, influenza, and pneumonia, in people with diabetes.

Design Population-based cohort study.

Setting UK primary care records (Clinical Practice Research Datalink) linked to mortality and hospital records.

Participants Individuals with type 1 and type 2 diabetes (Covid-19 cohort: N=43,033 type 1 diabetes and N=584,854 type 2 diabetes, influenza and pneumonia cohort: N=42,488 type 1 diabetes and N=585,289 type 2 diabetes).

Primary and secondary outcome measures Covid-19 hospitalisation from 1st February 2020-31st October 2020 (pre-Covid-19 vaccination roll-out), and influenza and pneumonia hospitalisation from 1st September 2016-31st May 2019 (pre-Covid-19 pandemic). Secondary outcomes were Covid-19 and pneumonia mortality. Associations between clinical and sociodemographic risk factors and each outcome were assessed using multivariable Cox proportional hazards models. In people with type 2 diabetes, we explored modifying effects of HbA1c and BMI by age, sex, and ethnicity.

Results In type 2 diabetes, poor glycaemic control and severe obesity were consistently associated with increased risk of hospitalisation for Covid-19, influenza, and pneumonia. The highest HbA1c and BMI-associated relative risks were observed in people under 70 years. Sociodemographic-associated risk differed markedly by respiratory infection, particularly for ethnicity. Compared to people of white ethnicity, black and south Asian groups had a greater risk of Covid-19 hospitalisation, but a lesser risk of pneumonia hospitalisation. Risk factor associations for type 1 diabetes and for type 2 diabetes mortality, were broadly consistent with the primary analysis.

Conclusions Clinical risk factors of high HbA1c and severe obesity are consistently associated with severe outcomes from Covid-19, influenza, and pneumonia, especially in younger people. In contrast, associations with sociodemographic risk factors differed by type of respiratory infection. This emphasises that risk stratification should be specific to individual respiratory infections.

For peer review only

Strengths and limitations of this study

- Uses a large primary care dataset of people with both type 1 and type 2 diabetes with linked hospital and mortality records
- Many potential risk factors, including potentially modifiable factors such as HbA1c and BMI, assessed and included in models guided by previous studies and clinical knowledge
- Sociodemographic risk factor associations, in particular ethnicity differences, may be influenced by factors unique to the Covid-19 pandemic, and knowledge of these differences could inform future risk stratification
- Limitations include the potential for misclassification of infection outcomes, however associations using different outcome definitions were robust in sensitivity analyses
- Associations do not have a causal interpretation but are still useful for identifying those at high risk

For peer review only

Introduction

People with diabetes are twice as likely to die from infection compared to the general population [1]. Infections are also a major cause of morbidity in people with diabetes, with 1 in 4 hospitalisations in men and 1 in 3 hospitalisations in women now being attributable to infections [2]. The burden of respiratory infections is increasing, comprising at least 10% of hospitalisations in people with diabetes in 2018, compared to less than 4% in 2003 [2]. During the Covid-19 pandemic, diabetes was one of the most common comorbidities associated with severe infection and poor outcomes [3-11]. People with type 2 diabetes were twice as likely to die with Covid-19 in hospital as the general population, with the risk being even higher in those with type 1 diabetes [11]. This is concordant with other severe respiratory infections including pneumonia, for which an excess risk of hospitalisation has been observed in people with type 2 diabetes [12], and influenza, for which an increased risk of severe infection has been observed in older adults with diabetes [13].

Despite the high burden of respiratory infection in people with diabetes, there has been no previous population-based comparison of risk factor associations across major respiratory infections. Previous studies have identified clear heterogeneity in the risk of severe Covid-19 in people with diabetes [14-17], with a higher risk in those of older age, male sex, non-white ethnicity, and higher social deprivation [16, 17], and differences by potentially modifiable risk factors, for example increased risk with higher HbA1c and higher BMI [14, 16-19]. Although broadly similar differences in the risk of other severe respiratory infections by sex [20-24], age [20-22], deprivation [20, 22, 25], ethnicity [26], glycaemic control [12, 27] and BMI [28] have been suggested in studies of people with and without diabetes, the consistency of these risk factor associations across respiratory infections has not been robustly established. The limited studies to-date comparing Covid-19 risk factors to influenza and pneumonia have used selectively recruited cohorts, focused on the specific risk factors of sex and obesity [29, 30], or used positive Covid-19 tests/incident infection (influenced by country-specific testing policies) as the outcome [31]. As recently highlighted by the Chief Medical Officer for England, research to provide a robust understanding of the comparability of risk factors for major respiratory infections is urgently needed [32], and could inform future risk stratification and targeted intervention.

In this study in people with diabetes, we aimed to compare clinical and sociodemographic risk factors for Covid-19 hospitalisation and mortality, with risk factors for influenza and pneumonia. We also aimed to assess heterogeneity in potentially modifiable HbA1c and BMI associated risk by age, sex, and ethnicity.

For peer review only - http://bmjopen.bmj.com/site/about/guidelines.xhtml

Methods

Data source

We used UK population-based data from the Clinical Practice Research Datalink (CPRD) Aurum, a large database of longitudinal, routinely collected medical records covering demographics, diagnoses, prescriptions, and test results [33]. CPRD Aurum data is sourced from over 2,000 primary care general practitioner (GP) practices from across the UK [34], representing 13% of the population and is largely representative of the broader UK population [33]. We extracted data on all individuals with diabetes actively registered with a GP practice from 1st September 2016 to 31st October 2020. Primary care records were linked to national records of hospital admissions (Hospital Episode Statistics (HES)) and deaths (Office for National Statistics (ONS)). This combined dataset is one of the largest primary and secondary care datasets in the world and gave us a near-complete healthcare and mortality record for over half a million people with diabetes. We also included linked Index of Multiple Deprivation data (the official national measure of deprivation).

Study population

Inclusion and Exclusion Criteria

We included people with type 1 diabetes and type 2 diabetes with valid linkage data (Supplemental figure 1). We classified type 1 diabetes and type 2 diabetes using an algorithm based on diabetes clinical code counts, insulin prescriptions, oral hypoglycaemic agent (OHA) prescriptions, and diagnosis age (algorithm available at https://github.com/Exeter-Diabetes/CPRD-Codelists). The accuracy of this classification approach has recently been confirmed against the use of genetic and biochemical biomarkers of diabetes type [35]. Only those diagnosed with type 2 diabetes aged 20 or over were included to reduce the impact of potential misclassification in young adults. No age restrictions were applied for people with type 1 diabetes.

Cohorts

To study Covid-19, we identified people with diabetes actively registered on 1st February 2020 (Covid-19 baseline date), who were followed up until 31st October 2020 (pre-Covid-19 vaccination roll-out, covering the first wave of Covid-19 and start of the second wave). To study influenza and pneumonia infection outcomes, we defined a pre-Covid-19 pandemic study period, including individuals with diabetes actively registered on 1st September 2016 (influenza/pneumonia baseline date) followed up until 31st May 2019. This study period enabled assessment of influenza and pneumonia outcomes over multiple recent UK influenza seasons prior to Covid-19.

Outcomes

Primary outcomes were first hospitalisation for Covid-19, influenza, or pneumonia during the respective study periods. Outcomes were identified using ICD-10 codes for the infection of interest (Supplemental table 1) recorded as a diagnosis during hospital admission in HES. Secondary outcomes were cause-specific mortality, defined as an ICD-10 code for the

For peer review only - http://bmjopen.bmj.com/site/about/guidelines.xhtml

infection of interest recorded as any cause of death, or hospitalisation for the infection of interest with the discharge method or location recorded as death.

Risk factors and covariates

The risk factors of interest comprised baseline sociodemographic features (sex, age, ethnicity, deprivation), diabetes features (duration of diabetes, HbA1c, microvascular complications (diabetic nephropathy, neuropathy, and retinopathy)), and BMI. We also included a wide range of covariates in our analysis: smoking status, major comorbidities (cardiovascular, respiratory, neurological, and oncological conditions, as well as chronic kidney disease, and recent hospitalisation (respiratory infection, any other cause)), diabetes treatments (no treatment, OHA prescription only, insulin), medications affecting the immune response (immunosuppressants, oral steroids), medications used for long term respiratory conditions (leukotrienes, long acting β agonists), and geographical region. Each comorbidity and medication were included as individual binary variables and missing data was treated as the absence of that covariate. All other covariates were included as categorical variables, and any missing values were grouped into a separate category. Full definitions of each variable are provided in Supplemental table 2, all were defined using primary care and, where appropriate, HES records (codes available at https://github.com/Exeter-Diabetes/CPRD-Codelists).

Statistical analysis

Primary analysis

Multivariable Cox proportional hazards models were used to identify risk factor associations for Covid-19, influenza, and pneumonia hospitalisation, with proportional hazards assumptions for each variable assessed by visual inspection of Schoenfeld residuals. People were followed up until the earliest of; the outcome of interest, deregistration from GP, death, or the end of each study period. Hazard ratios for the potential risk factors in the model were calculated for each infection separately. For people with type 2 diabetes, we fitted separate multivariable models for each infection outcome including all potential risk factors (sex, age, ethnicity, deprivation, diabetes duration, HbA1c, number of microvascular complications, BMI) and covariates (smoking status, comorbidities, diabetes treatment, other medications, and region [full analysis set] (Supplemental table 2)). As the cohort of people with type 1 diabetes was much smaller, we used a reduced risk factor set comprising sex, age, ethnicity, deprivation, diabetes duration, HbA1c, and BMI, with adjustment for region only (restricted analysis set). Analysis of mortality outcomes in people with type 1 diabetes, and mortality from influenza in people with type 2 diabetes, was not performed due to a limited number of deaths in these groups.

HbA1c and BMI specific associations

In people with type 2 diabetes, we assessed the associations of continuous HbA1c and BMI with hospitalisation for each infection outcome, adjusted for the full analysis set. To allow for non-linearity, restricted cubic splines were fitted with 5 knots for Covid-19 and pneumonia and with 3 knots for influenza, with knot selection informed by Akaike information criterion. To explore heterogeneity in risk across sociodemographic subgroups, we refitted the same

For peer review only - http://bmjopen.bmj.com/site/about/guidelines.xhtml

models including an interaction between continuous HbA1c/ BMI and subgroups defined by age (over 70 years old/ under 70 years old), sex (male/ female), and ethnicity (white/ black/ south Asian ethnicity). We grouped age into over and under 70 years in order to be consistent with previous research of Covid-19 risk factors in people with diabetes [17]. Statistical significance of subgroup interactions were tested using ANOVA. In people with type 1 diabetes, the same approach as for type 2 diabetes was used to assess continuous associations of HbA1c and BMI with hospitalisation outcomes, adjusted for the restricted analysis set. Subgroup analysis was not performed due to low numbers.

Sensitivity analysis

We repeated the primary analysis with restricted outcome definitions where the infection of interest was recorded as a primary diagnosis during the hospital admission or the primary cause of death. To assess the potential impact of influenza and pneumonia vaccination on our findings, we repeated the primary analysis in subgroups with and without a history of vaccination for influenza in the 2 years prior to baseline, and pneumococcal vaccination prior to baseline.

Patient and public involvement

Patients and the public were not involved in this study.

For peer review only

Results

Type 2 diabetes

584,854 people with type 2 diabetes were evaluated for Covid-19 outcomes, of whom 5,965 (1.02%) were hospitalised for Covid-19 from 1st February 2020 to 31st October 2020. 585,289 people with type 2 diabetes were evaluated for pneumonia and influenza outcomes, of whom 38,088 (6.51%) were hospitalised for pneumonia, and 3,226 (0.55%) were hospitalised with influenza from 1st September 2016 to 31st May 2019. Supplemental table 3 details the frequency of ICD10 codes recorded for each infection hospitalisation outcome. The baseline characteristics of the two cohorts were similar (Table 1 and Supplemental table 4). The majority of hospitalisations occurred in the older age groups for all three infections (Table 1). The median age of those hospitalised was 74.8 years for Covid-19, 75.6 years for influenza, and 79.4 years for pneumonia.

Table 1. Baseline clinical and sociodemographic characteristics in each cohort and Covid-19, influenza, and pneumonia hospitalisations in type 2 diabetes.

	2020 cohort	Covid-19 hospitalisations	2016 cohort	Influenza hospitalisations	Pneumonia hospitalisations
**Number of individuals**	584854	5965	585289	3226	38088
**Mean (SD) follow-up, days**		265.0 (38.3)		915.2 (224.8)	897.4 (246.3)
**Sex**					
Female	253360 (43.3)	2317 (38.8)	256656 (43.9)	1581 (49.0)	17140 (45.0)
Male	331494 (56.7)	3648 (61.2)	328633 (56.1)	1645 (51.0)	20948 (55.0)
**Age group, years**					
<40	12898 (2.2)	70 (1.2)	13468 (2.3)	46 (1.4)	164 (0.4)
40-49	46075 (7.9)	258 (4.3)	49649 (8.5)	128 (4.0)	717 (1.9)
50-59	114537 (19.6)	729 (12.2)	113285 (19.4)	329 (10.2)	2495 (6.6)
60-69	151493 (25.9)	1198 (20.1)	153782 (26.3)	679 (21.0)	6068 (15.9)
70-79	155424 (26.6)	1660 (27.8)	152401 (26.0)	1078 (33.4)	12073 (31.7)
80-89	89871 (15.4)	1671 (28.0)	88492 (15.1)	811 (25.1)	13324 (35.0)
90+	14556 (2.5)	379 (6.4)	14212 (2.4)	155 (4.8)	3247 (8.5)
**Ethnicity**					
White	445160 (76.1)	4200 (70.4)	457714 (78.2)	2586 (80.2)	33206 (87.2)
South Asian	77253 (13.2)	891 (14.9)	71050 (12.1)	403 (12.5)	2935 (7.7)
Black	35144 (6.0)	645 (10.8)	33175 (5.7)	156 (4.8)	1324 (3.5)
Other	9886 (1.7)	144 (2.4)	8178 (1.4)	35 (1.1)	306 (0.8)
Mixed	6267 (1.1)	69 (1.2)	5654 (1.0)	37 (1.1)	246 (0.6)
Unknown	11144 (1.9)	16 (0.3)	9518 (1.6)	9 (0.3)	71 (0.2)
**Index of multiple deprivation quintile**					
1 (least deprived)	102950 (17.6)	761 (12.8)	103184 (17.6)	502 (15.6)	6296 (16.5)
2	106968 (18.3)	921 (15.4)	109006 (18.6)	548 (17.0)	6923 (18.2)
3	113454 (19.4)	1088 (18.2)	113661 (19.4)	601 (18.6)	7264 (19.1)
4	127048 (21.7)	1446 (24.2)	125492 (21.4)	698 (21.6)	8199 (21.5)
5 (most deprived)	134146 (22.9)	1747 (29.3)	133563 (22.8)	873 (27.1)	9380 (24.6)
Missing	288 (0.0)	2 (0.0)	383 (0.1)	4 (0.1)	26 (0.1)
**Duration of diagnosed diabetes, years**					
<1	14311 (2.4)	67 (1.1)	36503 (6.2)	146 (4.5)	1401 (3.7)
1-2	60219 (10.3)	397 (6.7)	77510 (13.2)	306 (9.5)	3324 (8.7)
3-5	105094 (18.0)	796 (13.3)	111199 (19.0)	491 (15.2)	5218 (13.7)

For peer review only - http://bmjopen.bmj.com/site/about/guidelines.xhtml

	2020 cohort	Covid-19 hospitalisations	2016 cohort	Influenza hospitalisations	Pneumonia hospitalisations
6-9	128817 (22.0)	1116 (18.7)	125083 (21.4)	621 (19.2)	7088 (18.6)
10-14	124471 (21.3)	1250 (21.0)	126421 (21.6)	771 (23.9)	9620 (25.3)
15-19	89704 (15.3)	1225 (20.5)	62373 (10.7)	465 (14.4)	5764 (15.1)
20+	62238 (10.6)	1114 (18.7)	46200 (7.9)	426 (13.2)	5673 (14.9)
**HbA1c, mmol/mol**					
<48	167739 (28.7)	1679 (28.1)	175659 (30.0)	934 (29.0)	12271 (32.2)
48-53	116015 (19.8)	1054 (17.7)	116131 (19.8)	557 (17.3)	7015 (18.4)
53-64	147209 (25.2)	1352 (22.7)	142662 (24.4)	791 (24.5)	8912 (23.4)
64-75	66071 (11.3)	767 (12.9)	62291 (10.6)	412 (12.8)	4062 (10.7)
75-86	33970 (5.8)	408 (6.8)	32257 (5.5)	184 (5.7)	2191 (5.8)
86+	39727 (6.8)	580 (9.7)	37470 (6.4)	270 (8.4)	2646 (6.9)
Missing	14123 (2.4)	125 (2.1)	18819 (3.2)	78 (2.4)	991 (2.6)
**Number of microvascular complications**					
0	264011 (45.1)	1889 (31.7)	281811 (48.1)	1077 (33.4)	11930 (31.3)
1	216115 (37.0)	2140 (35.9)	204550 (34.9)	1256 (38.9)	14444 (37.9)
2	96948 (16.6)	1621 (27.2)	91671 (15.7)	789 (24.5)	10288 (27.0)
3	7780 (1.3)	315 (5.3)	7257 (1.2)	104 (3.2)	1426 (3.7)
**BMI, kg/m2**					
<18.5	3026 (0.5)	66 (1.1)	2801 (0.5)	16 (0.5)	497 (1.3)
18.5-24.9	86199 (14.7)	1009 (16.9)	79018 (13.5)	476 (14.8)	6985 (18.3)
25-29.9	184626 (31.6)	1747 (29.3)	178501 (30.5)	885 (27.4)	10950 (28.7)
30-34.9	151732 (25.9)	1350 (22.6)	151161 (25.8)	809 (25.1)	8249 (21.7)
35-39.9	74766 (12.8)	752 (12.6)	75339 (12.9)	405 (12.6)	3976 (10.4)
40+	48455 (8.3)	517 (8.7)	48685 (8.3)	302 (9.4)	2877 (7.6)
Missing	36050 (6.2)	524 (8.8)	49784 (8.5)	333 (10.3)	4554 (12.0)

Poor glycaemic control and severe obesity are consistent risk factors for Covid-19, influenza, and pneumonia in type 2 diabetes

High HbA1c >86 mmol/mol (>10%), was consistently associated with increased hospitalisation for Covid-19, influenza, and pneumonia (Figure 1). When assessed continuously, there was an increasing risk of Covid-19 and influenza hospitalisation above HbA1c 53 mmol/mol (7%), but a more gradual risk increase for pneumonia (Figure 2). Compared to a HbA1c of 53 mmol/mol (7%), a HbA1c of 75 mmol/mol (9%) was associated with a 15% increased risk of Covid-19 (HR 1.15 95%CI 1.06-1.26), 12% increased risk of influenza (HR 1.12 95%CI 1.07-1.18), and 6% increased risk of pneumonia hospitalisation (HR 1.06 95%CI 1.02-1.10).

There was a U-shaped association with BMI for Covid-19 and pneumonia, and obesity was associated with an increased risk of hospitalisation for all three respiratory infections (Figure 1). There was a stronger association between increasing BMI and Covid-19 hospitalisation than hospitalisation for influenza or pneumonia (Figure 3). Compared to a BMI of 30 kg/m^2, a BMI of 35 kg/m^2 was associated with an 19% increased risk of Covid-19 (HR 1.19 95%CI 1.10-1.27), 8% increased risk of influenza (HR 1.08 95%CI 1.05-1.11), and a 6% increased risk of pneumonia hospitalisation (HR 1.06 95%CI 1.03-1.09).

An increasing number of microvascular complications was also associated with increased hospitalisation for all three respiratory infections (Figure 1). Diabetes duration was not associated with hospitalisation for all 3 infections, except in those with short duration (< 1

For peer review only - http://bmjopen.bmj.com/site/about/guidelines.xhtml

year) where there was reduced risk for Covid-19 and pneumonia (Figure 2). Supplemental Figure 2 and Supplemental table 5 report the full set of risk factors studied.

HbA1c and BMI associated risk differs by age and sex

In subgroup analysis, we observed a difference in HbA1c associations by age but not sex; there was a stronger association between higher HbA1c and hospitalisation in those aged under 70 compared to those over 70 for Covid-19 and pneumonia, but not influenza (Figure 2). There was little evidence for a difference by sex (Figure 2). For BMI, there was a stronger association with increasing BMI and hospitalisation in those aged under 70 and in females for all three infections (Figure 3). We observed no clear difference in HbA1c or BMI associations by ethnicity, although numbers were limited (Supplemental Figure 3A, Supplemental Figure 3B).

Non-white ethnicity, male sex, and increased deprivation are more important risk factors for Covid-19 hospitalisation than pneumonia or influenza hospitalisation in type 2 diabetes

Across the three respiratory infections, we observed differential effects of ethnicity, male sex, and higher deprivation on hospitalisation risk. Compared to people of white ethnicity, black and south Asian groups had a higher risk of Covid-19 hospitalisation, but a lower risk of pneumonia hospitalisation (Figure 1). For influenza, a higher risk was seen in the south Asian group only. Male sex was more strongly associated with increased Covid-19 hospitalisation than pneumonia hospitalisation, and was associated with decreased hospitalisation for influenza (Figure 1). For all three infections higher deprivation was associated with a higher risk of hospitalisation, but there was a stronger association with Covid-19 than influenza or pneumonia (Figure 1). Older age was strongly associated with pneumonia hospitalisation, whereas a lesser age-associated gradients was seen for Covid-19 and influenza (Figure 1).

Risk factors associations are similar for mortality

2,635 of 584,854 (0.45%) died from Covid-19, and 12,652 of 585,289 (2.16%) died from pneumonia in the respective study periods (baseline characteristics: Supplemental table 6). Clinical and sociodemographic risk factor associations for Covid-19 and pneumonia mortality were largely consistent with those for hospitalisation (Supplemental figure 4).

Type 1 diabetes

Of people with type 1 diabetes, 209 of 43,033 (0.49%) were hospitalised with Covid-19 during the 2020 study period. 208 of 42,488 (0.49%) were hospitalised for influenza, and 1,401 of 42,488 (3.30%) were hospitalised with pneumonia during the 2016 to 2019 study period (baseline characteristics: Supplemental table 7). Similarly to type 2 diabetes, higher HbA1c and higher BMI were associated with increased hospitalisation for Covid-19, influenza, and pneumonia in type 1 diabetes (Supplemental figure 5, Supplemental figure 6A, Supplemental figure 6B). Sociodemographic associations were broadly similar to those

For peer review only - http://bmjopen.bmj.com/site/about/guidelines.xhtml

in type 2 diabetes, although the association between increasing age and pneumonia hospitalisation was not stronger than for Covid-19, and there was no clear difference in Covid-19 risk by deprivation quintile in type 1 diabetes. We observed an increased risk of Covid-19 hospitalisation in people of non-white ethnicities, and in people of south Asian ethnicity for influenza, but no difference in risk by ethnicity for pneumonia (Supplemental figure 5).

Sensitivity analyses

Results were largely consistent with the primary analyses when outcomes were restricted to primary hospitalisation diagnoses (type 1 diabetes: Supplemental table 8, Supplemental figure 7; type 2 diabetes: Supplemental table 9, Supplemental figure 8), and primary cause of death in type 2 diabetes (Supplemental table 10, Supplemental figure 9). When repeating the primary analysis in those vaccinated and unvaccinated, associations with pneumonia hospitalisation were consistent in those with and without a pneumococcal vaccination (Supplemental figure 10). There were very low numbers of people without an influenza vaccination, however, associations with influenza hospitalisation appeared to be consistent in those with and without vaccination for influenza (Supplemental figure 11).

For peer review only

For peer review only - http://bmjopen.bmj.com/site/about/guidelines.xhtml

Discussion

In a large-scale UK population-based analysis, we provide a comprehensive comparison of risk factors for Covid-19, influenza, and pneumonia in people with type 1 and type 2 diabetes. We show that although clinical risk factor associations for HbA1c, BMI, and microvascular complications are similar for all three respiratory infections, there is evidence that risk varies meaningfully by age and sex. Notably, whilst poor glycaemic control and marked obesity are consistently associated with an increased risk of hospitalisation and death across all three infections, in relative terms these risk increases are greater in those aged under 70. For BMI, the association between higher BMI (>30) and a severe outcome is consistently higher for females than males for all three infections.

We also demonstrate clear differences in sociodemographic risk factor associations by respiratory infection type. The previously reported higher risk of severe Covid-19 in people of non-white ethnicity [10, 17, 36] is not consistent with other respiratory infections and is the opposite pattern of association seen for pneumonia. Additionally, male sex and higher deprivation appear to be more important risk factors for Covid-19 than influenza and pneumonia. Findings have important implications for the management of modifiable infection risk factors in people with diabetes as well as current and future resource planning to manage severe respiratory infection.

Whilst Covid-19 risk factors in people with diabetes have previously been robustly described [16, 17] previous population-based studies have not compared risk factor associations with those seen for other major respiratory infections. To our knowledge, only one general population (with and without diabetes) study has compared a range of risk factors for Covid-19 to influenza and pneumonia. However, this study used a selected cohort (UK Biobank) and a positive test result/ incident infection as the outcome measure which means findings are likely to be influenced by UK testing policies. Due to the under-representation of non-white ethnicities in the UK Biobank, this study also had limited power to describe associations by ethnicity [31].

The associations between higher HbA1c and BMI and severe Covid-19 outcomes identified in this study are consistent with previous studies of people with diabetes [14, 16-19]. Whilst UK population-based data have suggested that associations between poor glycaemic control and obesity and Covid-19 mortality were greater in people younger than 70 [17], our study extends this previous work by evaluating non-linear continuous associations for HbA1c and BMI for severe Covid-19, and showing associations are not specific to Covid-19. For pneumonia, poor glycaemic control has been associated with increased hospitalisation [12] but risk heterogeneity by patient phenotype has not been previously evaluated. Our finding of a markedly increased risk of severe Covid-19, influenza, and pneumonia in females with higher BMI is supported by prospective cohort data on the whole UK Biobank population, including individuals with and without diabetes [29].

Notably, we show that previously described risk factor associations between male sex, older age, non-white ethnicity, increasing deprivation, and severe Covid-19 outcomes in people with diabetes [16, 17] are not generalisable to influenza and pneumonia. Previous studies describing associations between sex and pneumonia have not reported consistent effects, [37] and evidence is also lacking for risk factors associated with severe influenza with the exception of increasing age [38]. Previous studies have found evidence of increased risk of severe Covid-19 outcomes in those of non-white ethnicity [10, 17, 36] consistent with our

For peer review only - http://bmjopen.bmj.com/site/about/guidelines.xhtml

findings. Whilst these studies have mainly been based on UK data, ethnic disparities in rates of influenza-associated hospitalisation have been observed in US data, with certain ethnic groups having poorer outcomes compared to those of white ethnicity [26].

Strengths and Limitations

Strengths of our study include the large size of the dataset, representativeness to the UK population, and linkage to longitudinal hospital admission and mortality data allowing us to robustly define infection outcomes and systematically compare Covid-19, influenza, and pneumonia risk factor associations. The large dataset allowed us to study both type 1 diabetes and type 2 diabetes. We evaluated associations for a range of risk factors and across different subgroups, however low numbers in certain groups may limit the precision of those estimates. As with all studies of this nature, misclassification or misdiagnosis of our outcomes is possible and relies on correct coding in the records. Evaluating both hospitalisation and mortality outcomes, and analysis to evaluate the sensitivity of results to our outcome definitions, mitigated against this limitation. Despite this, we were unable to completely differentiate between infection being the reason for admission or death or being contracted during admission, or only contributing to hospitalisation/death. We have also noted some differences in the coding of the studied respiratory infections in the hospital records; Covid-19 and influenza were mostly confirmed by a positive test whereas pneumonia coding was much broader and less specific, which should be considered when interpreting the results. The associations we have found do not have a causal interpretation and we cannot rule out potential confounding by unmeasured variables, which is an inherent limitation of many observational studies, however, we have adjusted for a wide range of covariates. There is also potential for misclassification of diabetes type in our cohorts, but we have used comprehensive algorithms considering prescriptions, age at diagnosis as well as diabetes codes, to define diabetes type as thoroughly as possible. This approach has been validated against a biological diabetes definition using classification biomarkers [35].

Due to our focus on severe infection outcomes, we will have missed milder community cases of infection; to assess risk factors for being infected would have required a different study design. The use of two different study periods for Covid-19 and influenza/ pneumonia, while unavoidable, has a risk of bias and may have influenced the observed associations. The associations found may reflect differences in behaviour, in particular during the first wave of the Covid-19 pandemic, and be influenced by factors unique to this period including marked geographical differences in infection rates, as well interventions such as lockdowns and shielding. The disproportionate impact of these measures on non-white and more deprived groups [39] may have influenced the sociodemographic disparities we have observed. The risks associated with the disruption to routine care in people with diabetes during the Covid-19 pandemic [40] also makes comparisons with a pre-pandemic study period difficult. Despite these limitations, a key strength of our analysis is the use of standardised case and risk factor definitions, and consistent statistical methodology, ensuring associations observed across the two study periods were as comparable as possible. Using this approach, we demonstrate major clinical risk factors are very similar across infections, a finding that is unlikely to be explained by specific impacts of the Covid-19 pandemic.

For peer review only - http://bmjopen.bmj.com/site/about/guidelines.xhtml

Clinical implications

This study highlights the potential importance of good glycaemic control and supporting weight loss for managing risk of three major respiratory infections in people with diabetes. While BMI may be hard to modify at an individual level, a population-level investment in effective weight reduction measures may help reduce severe respiratory infection. The substantial differences in sociodemographic risk factor associations across the different respiratory infections suggest we cannot assume high-risk groups for one respiratory infection are the same as another. This means existing risk models for respiratory infection, such as those developed for Covid-19 [5, 41], may not be applicable to new respiratory infections and so in the event of a new respiratory pathogen, early analysis of risk factors would be needed to correctly target interventions. The finding that the increased risk of severe Covid-19 in those of non-white ethnicity is largely not seen for other respiratory infections (except for an increase in severe influenza risk in people of south Asian ethnicity) highlights the importance of further exploration as to why ethnicity-associated infection risk appears to vary by infection type.

Conclusion

Poor glycaemic control and obesity are associated with a consistently increased risk of severe outcomes from Covid-19, influenza, and pneumonia, especially in those aged under 70. In contrast, even in a single country setting, sociodemographic risk factor associations differ by respiratory infection. This highlights the need for the development of population-specific risk stratification approaches for individual respiratory infections to ensure accurate identification of people with diabetes at the highest risk of severe outcomes.

For peer review only

Acknowledgements

This article is based on data from the Clinical Practice Research Datalink obtained under licence from the UK Medicines and Healthcare products Regulatory Agency. CPRD data is provided by patients and collected by the NHS as part of their care and support. Approval for CPRD data access and the study protocol was granted by the CPRD Independent Scientific Advisory Committee (Project-ID: 20_000101).

This study was supported by the National Institute for Health and Care Research Exeter Biomedical Research Centre. The views expressed are those of the author(s) and not necessarily those of the NIHR or the Department of Health and Social Care or Diabetes UK. RH and KGY are supported by Research England's Expanding Excellence in England (E3) fund. NJT is funded by a Wellcome Trust–funded GW4 PhD fellowship. RC is funded through the AvonCAP study which is an investigator-led University of Bristol study funded by Pfizer and further supported by the National Institute for Health and Care Research Applied Research Collaboration South West Peninsula. BMS is supported by the NIHR Exeter Clinical Research Facility. JMD is supported by an Independent Fellowship funded by Research England's Expanding Excellence in England (E3) fund. The funders had no role in the study design, data collection, data analysis, data interpretation, writing of the article, or the decision to submit for publication.

Funding

This work was supported by Diabetes UK (20/0006220).

Conflict of Interest

BAM is an employee of Wellcome Trust; holds an honorary post at the Alan Turing Institute and University College London; and declares payments from the Medical Research Council, Health Data Research UK, British Heart Foundation, and Engineering and Physical Sciences Research Council (grant EP/N510129/). RC declares research funding from Pfizer. SJV declares support from the University of Warwick, University of Kaiserslautern, and German Research Center for Artificial Intelligence; consulting fees from PUMAS; stock from Freshflow; and grant funding from The Alan Turing Institute (EP/N510129), Engineering and Physical Sciences Research Council, and Massachusetts Institute of Technology. APM received research funding from Eli Lilly and Company, Pfizer, and AstraZeneca. For all authors these are outside the submitted work; there are no other relationships or activities that could appear to have influenced the submitted work.

Author Contributions and Guarantor Statement

The study concept and design were conceived and developed by RH, JMD, APM, BAM, SJV, and NJT. JMD, KGY, RH, and BMS had access to all the raw data sets used for the study. RH, KGY, DR, and RC prepared the data for analysis. RH undertook the analysis, with support from JMD, BMS, JG, and APM. All authors provided support for the interpretation of results, critically revised the manuscript, and saw and approved the final article. JMD and APM attest that all listed authors meet authorship criteria, that no others

For peer review only - http://bmjopen.bmj.com/site/about/guidelines.xhtml

meeting the criteria have been omitted. JMD and APM were responsible for the decision to submit for publication, and are the guarantors of this work and, as such, had full access to all the data in the study and takes responsibility for the integrity of the data and the accuracy of the data analysis.

Data availability

No additional data are available from the authors although CPRD data are available by application to CPRD Independent Scientific Advisory Committee.

Prior presentation

Oral presentation of preliminary results from this study have been given at the Diabetes UK Professional Conference 2022 and the European Association for the Study of Diabetes (EASD) Annual Meeting 2022.

Ethics Approval

The study protocol was approved by the CPRD Independent Scientific Advisory Committee (Project-ID: 20_000101).

For peer review only

References

1. Carey IM, Critchley JA, DeWilde S, et al. Risk of Infection in Type 1 and Type 2 Diabetes Compared With the General Population: A Matched Cohort Study. Diabetes Care 2018;41(3):513-21. doi: 10.2337/dc17-2131

2. Pearson-Stuttard J, Cheng YJ, Bennett J, et al. Trends in leading causes of hospitalisation of adults with diabetes in England from 2003 to 2018: an epidemiological analysis of linked primary care records. Lancet Diabetes Endocrinol 2022;10(1):46-57. doi: 10.1016/S2213-8587(21)00288-6

3. Grasselli G, Greco M, Zanella A, et al. Risk Factors Associated With Mortality Among Patients With COVID-19 in Intensive Care Units in Lombardy, Italy. JAMA Intern Med 2020;180(10):1345-55. doi: 10.1001/jamainternmed.2020.3539

4. Zhou F, Yu T, Du R, et al. Clinical course and risk factors for mortality of adult inpatients with COVID-19 in Wuhan, China: a retrospective cohort study. Lancet 2020;395(10229):1054-62. doi: 10.1016/S0140-6736(20)30566-3

5. Clift AK, Coupland CAC, Keogh RH, et al. Living risk prediction algorithm (QCOVID) for risk of hospital admission and mortality from coronavirus 19 in adults: national derivation and validation cohort study. BMJ 2020;371:m3731. doi: 10.1136/bmj.m3731

6. Roncon L, Zuin M, Rigatelli G, et al. Diabetic patients with COVID-19 infection are at higher risk of ICU admission and poor short-term outcome. J Clin Virol 2020;127:104354. doi: 10.1016/j.jcv.2020.104354

7. Richardson S, Hirsch JS, Narasimhan M, et al. Presenting Characteristics, Comorbidities, and Outcomes Among 5700 Patients Hospitalized With COVID-19 in the New York City Area. JAMA 2020;323(20):2052-59. doi: 10.1001/jama.2020.6775

8. Docherty AB, Harrison EM, Green CA, et al. Features of 20 133 UK patients in hospital with covid-19 using the ISARIC WHO Clinical Characterisation Protocol: prospective observational cohort study. BMJ 2020;369:m1985. doi: 10.1136/bmj.m1985

9. Dennis JM, Mateen BA, Sonabend R, et al. Type 2 Diabetes and COVID-19-Related Mortality in the Critical Care Setting: A National Cohort Study in England, March-July 2020. Diabetes Care 2021;44(1):50-57. doi: 10.2337/dc20-1444

10. Williamson EJ, Walker AJ, Bhaskaran K, et al. Factors associated with COVID-19-related death using OpenSAFELY. Nature 2020;584(7821):430-36. doi: 10.1038/s41586-020-2521-4

11. Barron E, Bakhai C, Kar P, et al. Associations of type 1 and type 2 diabetes with COVID-19-related mortality in England: a whole-population study. Lancet Diabetes Endocrinol 2020;8(10):813-22. doi: 10.1016/S2213-8587(20)30272-2

12. Kornum JB, Thomsen RW, Riis A, et al. Diabetes, glycemic control, and risk of hospitalization with pneumonia: a population-based case-control study. Diabetes Care 2008;31(8):1541-5. doi: 10.2337/dc08-0138

13. Owusu D, Rolfes MA, Arriola CS, et al. Rates of Severe Influenza-Associated Outcomes Among Older Adults Living With Diabetes-Influenza Hospitalization Surveillance Network

For peer review only - http://bmjopen.bmj.com/site/about/guidelines.xhtml

(FluSurv-NET), 2012-2017. Open Forum Infect Dis 2022;9(5):ofac131. doi: 10.1093/ofid/ofac131

14. Cariou B, Hadjadj S, Wargny M, et al. Phenotypic characteristics and prognosis of inpatients with COVID-19 and diabetes: the CORONADO study. Diabetologia 2020;63(8):1500-15. doi: 10.1007/s00125-020-05180-x

15. Shi Q, Zhang X, Jiang F, et al. Clinical Characteristics and Risk Factors for Mortality of COVID-19 Patients With Diabetes in Wuhan, China: A Two-Center, Retrospective Study. Diabetes Care 2020;43(7):1382-91. doi: 10.2337/dc20-0598

16. McGurnaghan SJ, Weir A, Bishop J, et al. Risks of and risk factors for COVID-19 disease in people with diabetes: a cohort study of the total population of Scotland. Lancet Diabetes Endocrinol 2021;9(2):82-93. doi: 10.1016/S2213-8587(20)30405-8

17. Holman N, Knighton P, Kar P, et al. Risk factors for COVID-19-related mortality in people with type 1 and type 2 diabetes in England: a population-based cohort study. Lancet Diabetes Endocrinol 2020;8(10):823-33. doi: 10.1016/S2213-8587(20)30271-0

18. Hamer M, Gale CR, Batty GD. Diabetes, glycaemic control, and risk of COVID-19 hospitalisation: Population-based, prospective cohort study. Metabolism 2020;112:154344. doi: 10.1016/j.metabol.2020.154344

19. Zhu L, She ZG, Cheng X, et al. Association of Blood Glucose Control and Outcomes in Patients with COVID-19 and Pre-existing Type 2 Diabetes. Cell Metab 2020;31(6):1068-77 e3. doi: 10.1016/j.cmet.2020.04.021

20. Jordan R, Verlander N, Olowokure B, et al. Age, sex, material deprivation and respiratory mortality. Respir Med 2006;100(7):1282-5. doi: 10.1016/j.rmed.2005.10.014

21. Thomsen RW, Riis A, Norgaard M, et al. Rising incidence and persistently high mortality of hospitalized pneumonia: a 10-year population-based study in Denmark. J Intern Med 2006;259(4):410-7. doi: 10.1111/j.1365-2796.2006.01629.x

22. Myles PR, Hubbard RB, Gibson JE, et al. Pneumonia mortality in a UK general practice population cohort. Eur J Public Health 2009;19(5):521-6. doi: 10.1093/eurpub/ckp081

23. Millett ER, De Stavola BL, Quint JK, et al. Risk factors for hospital admission in the 28 days following a community-acquired pneumonia diagnosis in older adults, and their contribution to increasing hospitalisation rates over time: a cohort study. BMJ Open 2015;5(12):e008737. doi: 10.1136/bmjopen-2015-008737

24. Lopez-de-Andres A, Jimenez-Garcia R, Hernandez-Barrera V, et al. Sex-Related Disparities in the Incidence and Outcomes of Community-Acquired Pneumonia among Type 2 Diabetes Patients: A Propensity Score-Matching Analysis Using the Spanish National Hospital Discharge Database for the Period 2016-2019. J Clin Med 2021;10(17) doi: 10.3390/jcm10173975

25. Hungerford D, Ibarz-Pavon A, Cleary P, et al. Influenza-associated hospitalisation, vaccine uptake and socioeconomic deprivation in an English city region: an ecological study. BMJ Open 2018;8(12):e023275. doi: 10.1136/bmjopen-2018-023275

For peer review only - http://bmjopen.bmj.com/site/about/guidelines.xhtml

26. O'Halloran AC, Holstein R, Cummings C, et al. Rates of Influenza-Associated Hospitalization, Intensive Care Unit Admission, and In-Hospital Death by Race and Ethnicity in the United States From 2009 to 2019. JAMA Netw Open 2021;4(8):e2121880. doi: 10.1001/jamanetworkopen.2021.21880

27. Breitling LP. Evidence of non-linearity in the association of glycemic control with influenza/pneumonia mortality: a study of 19 000 adults from the US general population. Diabetes Metab Res Rev 2016;32(1):111-20. doi: 10.1002/dmrr.2681

28. Kornum JB, Norgaard M, Dethlefsen C, et al. Obesity and risk of subsequent hospitalisation with pneumonia. Eur Respir J 2010;36(6):1330-6. doi: 10.1183/09031936.00184209

29. Peters SAE, MacMahon S, Woodward M. Obesity as a risk factor for COVID-19 mortality in women and men in the UK biobank: Comparisons with influenza/pneumonia and coronary heart disease. Diabetes Obes Metab 2021;23(1):258-62. doi: 10.1111/dom.14199

30. de Jong M, Woodward M, Peters SAE. Diabetes and COVID-19-Related Mortality in Women and Men in the UK Biobank: Comparisons With Influenza/Pneumonia and Coronary Heart Disease. Diabetes Care 2021;44(2):e22-e24. doi: 10.2337/dc20-2378

31. Ho FK, Celis-Morales CA, Gray SR, et al. Modifiable and non-modifiable risk factors for COVID-19, and comparison to risk factors for influenza and pneumonia: results from a UK Biobank prospective cohort study. BMJ Open 2020;10(11):e040402. doi: 10.1136/bmjopen-2020-040402

32. Health Data Research UK. HDR UK and NIHR issue a rapid funding opportunity for Data Science to inform NHS compound winter pressure policy response. 2022. Available: https://www.hdruk.ac.uk/funding-opportunities/rapid-funding-winter-pressure/ [Accessed 4 January 2023]

33. Wolf A, Dedman D, Campbell J, et al. Data resource profile: Clinical Practice Research Datalink (CPRD) Aurum. Int J Epidemiol 2019;48(6):1740-40g. doi: 10.1093/ije/dyz034

34. Clinical Practice Research Datalink. CPRD Data. Available: https://cprd.com/data [Accessed 11 April 2023]

35. Thomas NJ, McGovern A, Young KG, et al. Identifying type 1 and 2 diabetes in research datasets where classification biomarkers are unavailable: assessing the accuracy of published approaches. J Clin Epidemiol 2022;153:34-44. doi: 10.1016/j.jclinepi.2022.10.022

36. Mathur R, Rentsch CT, Morton CE, et al. Ethnic differences in SARS-CoV-2 infection and COVID-19-related hospitalisation, intensive care unit admission, and death in 17 million adults in England: an observational cohort study using the OpenSAFELY platform. Lancet 2021;397(10286):1711-24. doi: 10.1016/S0140-6736(21)00634-6

37. Almirall J, Serra-Prat M, Bolibar I, et al. Risk Factors for Community-Acquired Pneumonia in Adults: A Systematic Review of Observational Studies. Respiration 2017;94(3):299-311. doi: 10.1159/000479089

38. Mertz D, Kim TH, Johnstone J, et al. Populations at risk for severe or complicated influenza illness: systematic review and meta-analysis. BMJ 2013;347:f5061. doi: 10.1136/bmj.f5061

For peer review only - http://bmjopen.bmj.com/site/about/guidelines.xhtml

39. Khunti K, Feldman EL, Laiteerapong N, et al. The Impact of the COVID-19 Pandemic on Ethnic Minority Groups With Diabetes. Diabetes Care 2022 doi: 10.2337/dc21-2495

40. Valabhji J, Barron E, Gorton T, et al. Associations between reductions in routine care delivery and non-COVID-19-related mortality in people with diabetes in England during the COVID-19 pandemic: a population-based parallel cohort study. Lancet Diabetes Endocrinol 2022;10(8):561-70. doi: 10.1016/S2213-8587(22)00131-0

41. Wynants L, Van Calster B, Collins GS, et al. Prediction models for diagnosis and prognosis of covid-19: systematic review and critical appraisal. BMJ 2020;369:m1328. doi: 10.1136/bmj.m1328

For peer review only

For peer review only - http://bmjopen.bmj.com/site/about/guidelines.xhtml

Figure legends

Figure 1: Hazard ratios and 95% confidence intervals for the association of clinical and sociodemographic risk factors with hospitalisation for Covid-19 (orange squares), influenza (green circles), and pneumonia (blue diamonds) in type 2 diabetes.

Figure 2: Hazard ratios and 95% confidence intervals for the association of continuous HbA1c with hospitalisation for Covid-19, influenza, and pneumonia in type 2 diabetes. Overall, and by age and sex. Statistical significance of age subgroup interactions with HbA1c: $p=0.0190$ for Covid-19, $p=0.2214$ for influenza, and $p<.0001$ for pneumonia. Statistical significance of sex subgroup interactions with HbA1c: $p=0.5488$ for Covid-19, $p=0.0737$ for influenza, and $p=0.0037$ for pneumonia. Density plots show the distribution of HbA1c in each group.

Figure 3: Hazard ratios and 95% confidence intervals for the association of continuous BMI with hospitalisation for Covid-19, influenza, and pneumonia in type 2 diabetes. Overall, and by age and sex. Statistical significance of age subgroup interactions with BMI: $p=0.0046$ for Covid-19, $p=0.0934$ for influenza, and $p<.0001$ for pneumonia. Statistical significance of sex subgroup interactions with BMI: $p=0.2780$ for Covid-19, $p=0.0008$ for influenza, and $p=<.0001$ for pneumonia. Density plots show the distribution of BMI in each group.

For peer review only - http://bmjopen.bmj.com/site/about/guidelines.xhtml

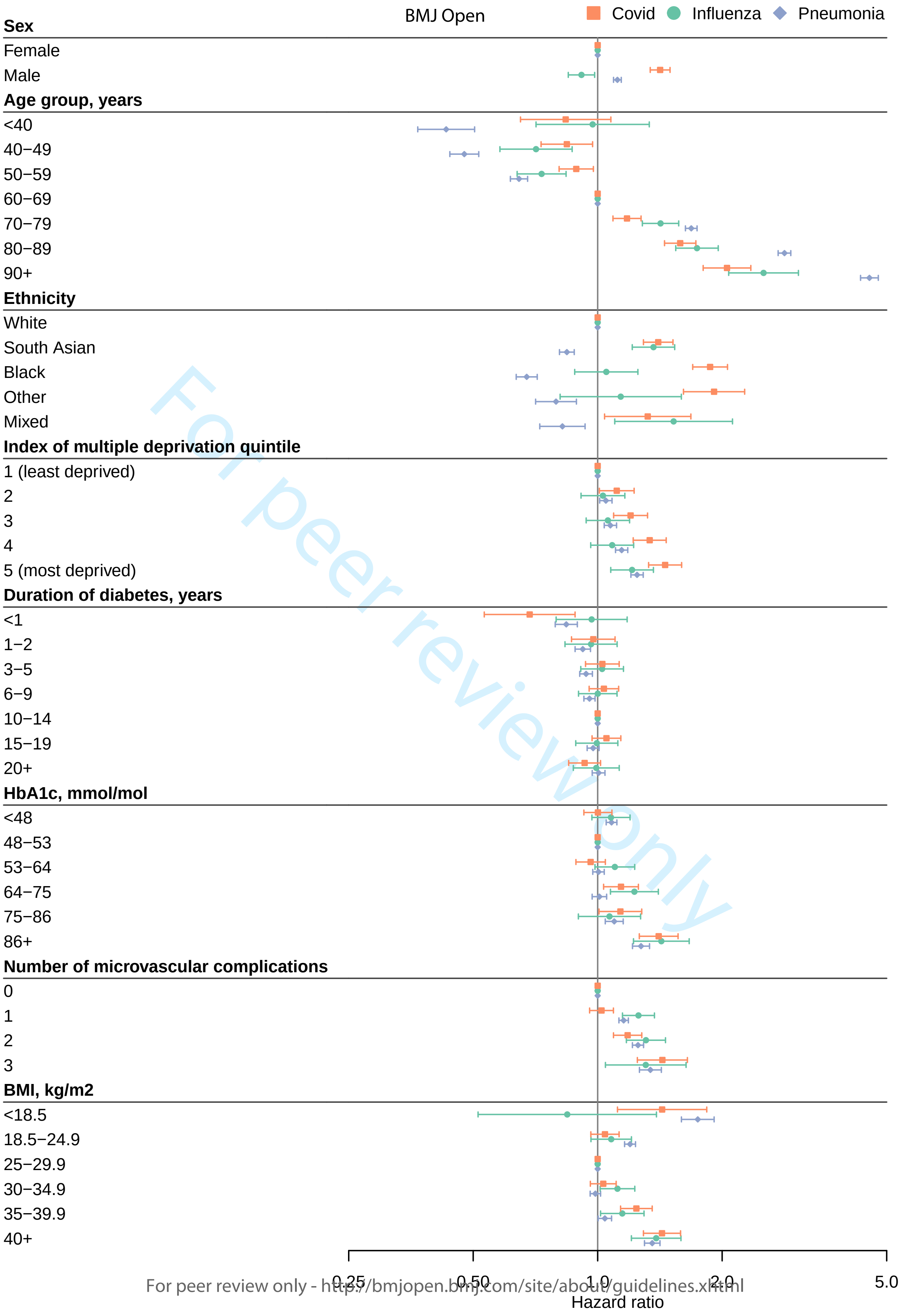

For peer review only

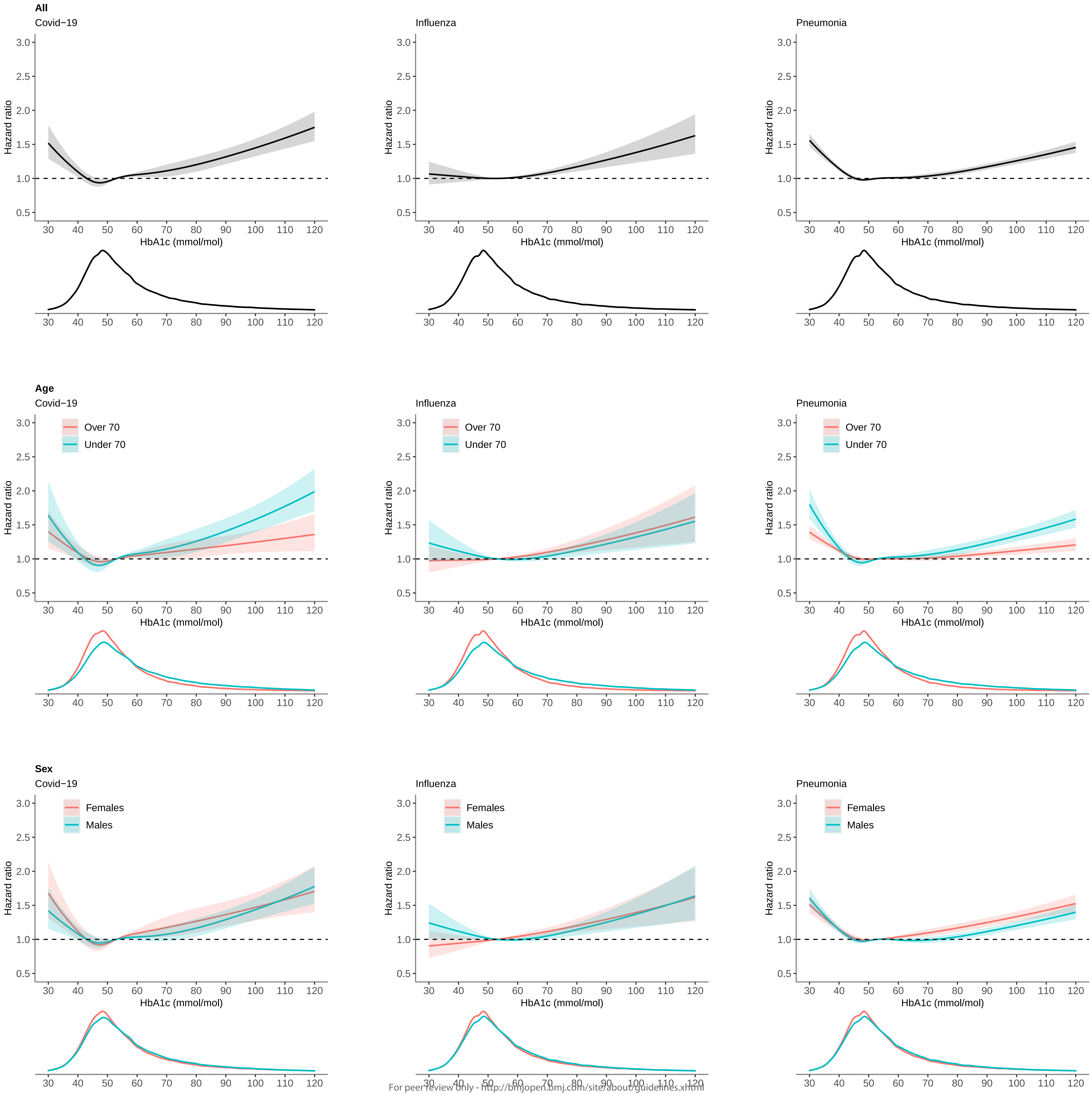
All
Covid−19
Influenza
Pneumonia
Hazard ratio
HbA1c (mmol/mol)
Age
Over 70
Under 70
Sex
Females
Males

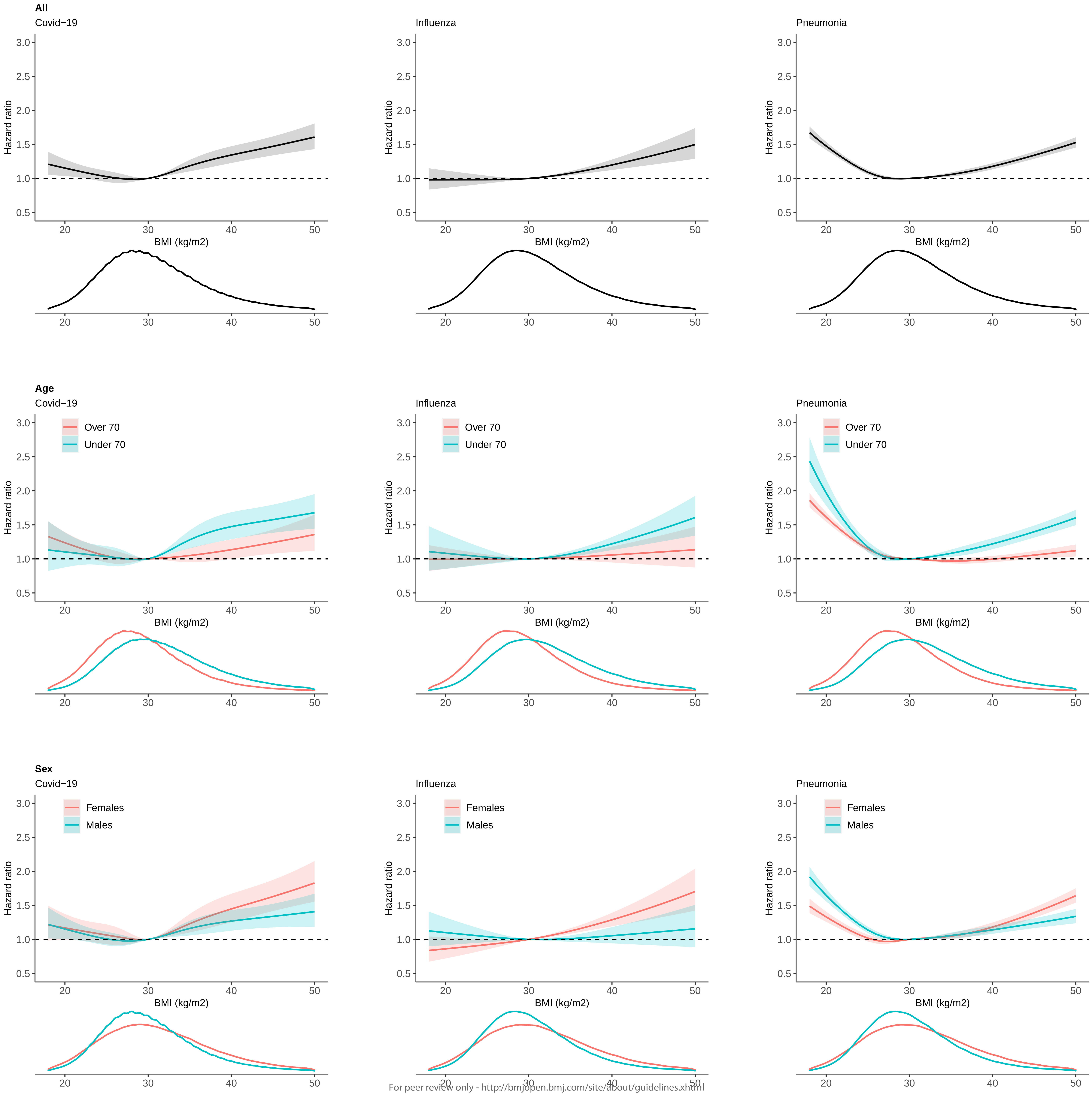
All
Covid−19
Influenza
Pneumonia
Hazard ratio
3.0
2.5
2.0
1.5
1.0
0.5
20
30
40
50
BMI (kg/m2)
Age
Over 70
Under 70
Sex
Females
Males

Supplemental material

Table of Contents

Page	
**2**	Supplemental figure 1. Flow diagram of study participants and exclusions.
**3**	Supplemental table 1. ICD-10 codes used to define Covid-19, influenza, and pneumonia outcomes.
**4**	Supplemental table 2. Definitions of exposure variables.
	**Type 2 diabetes (hospitalisation)**
**5**	Supplemental table 3. Frequency of ICD-10 codes recorded in hospitalisation outcomes in type 2 diabetes for Covid-19, influenza, and pneumonia.
**6-7**	Supplemental table 4. Full baseline characteristics and Covid-19, influenza, and pneumonia hospitalisations in type 2 diabetes.
**8**	Supplemental figure 2. Association of potential risk factors with hospitalisation for Covid-19, influenza, and pneumonia in type 2 diabetes.
**9-10**	Supplemental figure 5. Hazard ratio values for the association of potential risk factors with hospitalisation for Covid-19, influenza, and pneumonia in type 2 diabetes.
**11**	Supplemental figure 3A. Association of continuous HbA1c with hospitalisation for Covid-19, influenza, and pneumonia in type 2 diabetes by ethnicity subgroup.
**11**	Supplemental figure 3B. Association of continuous BMI with hospitalisation for Covid-19, influenza, and pneumonia in type 2 diabetes by ethnicity subgroup.
	**Type 2 diabetes (mortality)**
**12-13**	Supplemental table 6. Baseline characteristics and Covid-19 and pneumonia deaths in type 2 diabetes.
**14**	Supplemental figure 4. Association of potential risk factors with Covid-19, and pneumonia mortality in type 2 diabetes.
	**Type 1 diabetes**
**15**	Supplemental table 7. Baseline characteristics and Covid-19, influenza, and pneumonia hospitalisations in type 1 diabetes.
**16**	Supplemental figure 5. Association of potential risk factors with hospitalisation for Covid-19, influenza, and pneumonia in type 1 diabetes.
**17**	Supplemental figure 6A. Association of continuous HbA1c with hospitalisation for Covid-19, influenza, and pneumonia in type 1 diabetes.
**17**	Supplemental figure 6B. Association of continuous BMI with hospitalisation for Covid-19, influenza, and pneumonia in type 1 diabetes.
	**Sensitivity analyses**
**18**	Supplemental table 8. Baseline characteristics and Covid-19, influenza, and pneumonia hospitalisations in type 1 diabetes, restricting hospitalisation definition to primary diagnosis in HES.
**19**	Supplemental figure 7. Association of potential risk factors with hospitalisation for Covid-19, influenza, and pneumonia in type 1 diabetes, restricting hospitalisation definition to primary diagnosis in HES.
**20-21**	Supplemental table 9. Baseline characteristics and Covid-19, influenza, and pneumonia hospitalisations in type 2 diabetes, restricting hospitalisation definition to primary diagnosis in HES.
**22**	Supplemental figure 8. Association of potential risk factors with hospitalisation for Covid-19, influenza, and pneumonia in type 2 diabetes, restricting hospitalisation definition to primary diagnosis in HES.
**23-24**	Supplemental table 10. Baseline characteristics and Covid-19 and pneumonia deaths in type 2 diabetes, restricting mortality definition to primary cause in ONS.
**25**	Supplemental figure 9. Association of potential risk factors with Covid-19, and pneumonia mortality in type 2 diabetes, restricting mortality definition to primary cause in ONS.
**26**	Supplemental figure 10. Association of potential risk factors with pneumonia hospitalisation in those with a pneumococcal vaccination and those without.
**27**	Supplemental figure 11. Association of potential risk factors with influenza hospitalisation in those with an influenza vaccination and those without.
**28**	References

For peer review only

Supplemental figure 1. Flow diagram of study participants and exclusions.

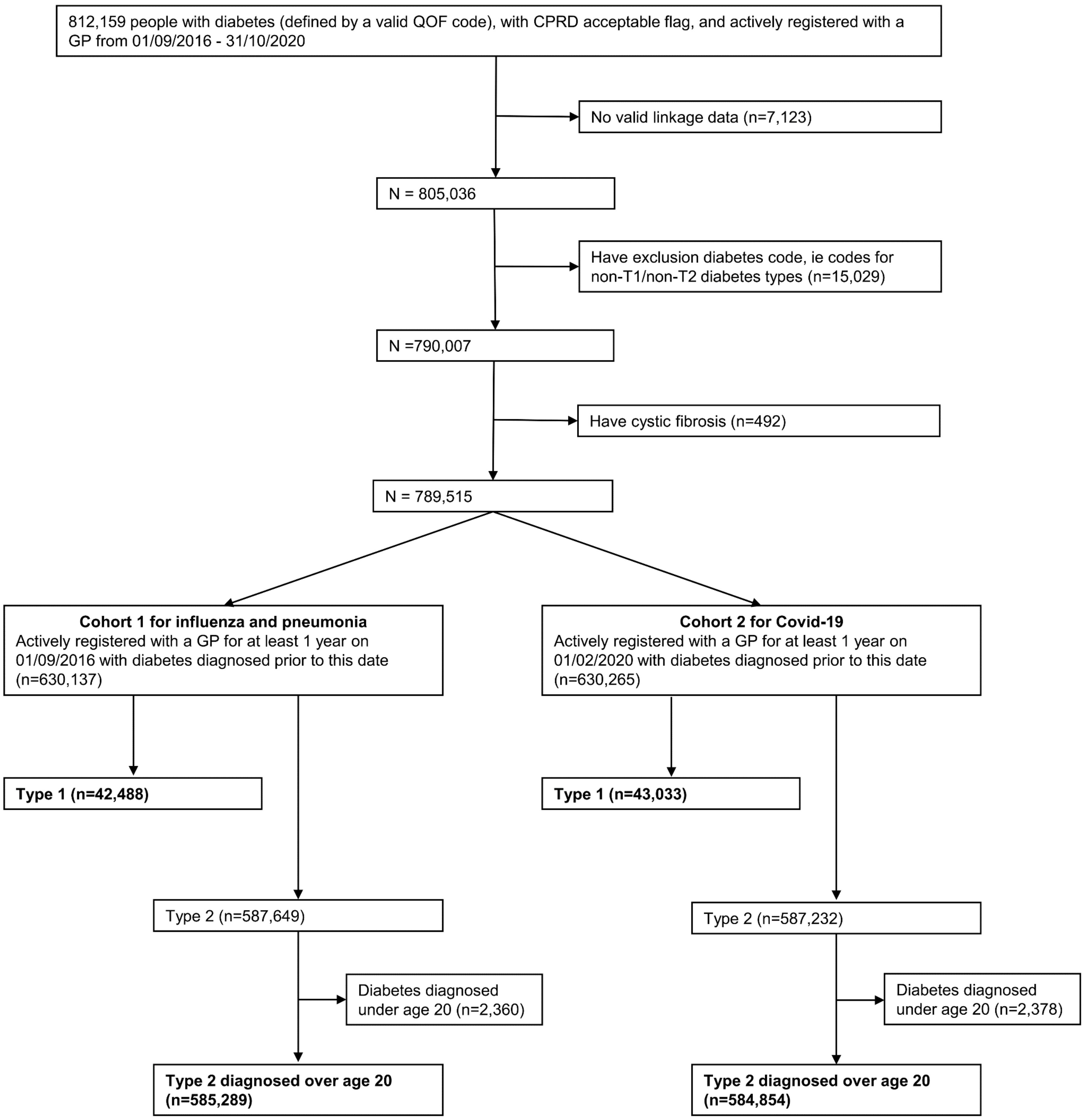

Supplemental table 1. ICD-10 codes used to define Covid-19, influenza, and pneumonia hospitalisation and mortality outcomes.

**Infection**	**ICD-10 codes**
Covid-19	U07.1, U07.2
Influenza	J09-J11
Pneumonia	J12-J18

For peer review only

Supplemental table 2. Definitions of exposure variables.

Variable	Definition	Data source	Categories	Missing data method
Sex	Sex recorded in CPRD	Primary care	Male, or female	No missing data
Age	Age at baseline date, calculated from date of birth	Primary care	<40, 40-49, 50-59, 60-69, 70-79, 80-89 or 90+ years for type 2 diabetes cohorts, and <18, 18-39, 40-49, 50-59, 60-69, 70-79 or 80+ years for type 1 diabetes cohorts	No missing data
Ethnicity	Major UK ethnic group based on a previous study [1]	Primary care and HES	White, south Asian, black, mixed, other, or unknown	Missing indicator method
Deprivation	Index of Multiple Deprivation (official national measure) [2]	Linked Index of Multiple Deprivation dataset	1, 2, 3, 4, 5, or missing (Quintile of national distribution)	Missing indicator method
Smoking status	Smoking status at baseline date	Primary care	Non-smoker, active smoker, ex-smoker, or unknown	Missing indicator method
Region	UK strategic health region based on GP practice location	Primary care	East Midlands, East of England, London, North East, North West, South Central, South East Coast, South West, West Midlands, Yorkshire and the Humber, or missing	Missing indicator method
HbA1c	Latest recorded HbA1c measurement in the last two years prior to baseline date	Primary care	<48 mmol/ mol (<6.5%), 48-53 mmol/mol (6.5-7%), 53-64 mmol/mol (7-8%), 64-75 mmol/mol (8-9%), 75-86 mmol/mol (9-10%), >86 mmol/mol (>10%), or missing	Missing indicator method
BMI	Latest recorded BMI measurement in the last two years prior to baseline date, grouped using the World Health Organisation classification [3]	Primary care	<18.5 kg/m², 18.5-24.9 kg/m², 25-29.9 kg/m², 30-34.5 kg/m², 35-39.9 kg/m², 40+ kg/m², or missing	Missing indicator method
Diabetes duration	Years since diabetes diagnosis, with diagnosis date defined using an algorithm adapted from a previous study [4] taking the earliest date of first diabetes clinical code, first HbA1c measurement of 48 mmol/mol (6.5%) or above, first oral hypoglycaemic agent (OHA) prescription or first insulin prescription	Primary care	<1 year, 1-2 years, 3-5 years, 6-9 years, 10-14 years, 15-19 years or 20+ years for the type 2 diabetes cohorts, and <5 years, 5-9 years, 10-14 years, 15-19 years, 20-24 years, 25-29 years or 30+ years for type 1 diabetes cohorts	No missing data
Microvascular complications	Total number of coded microvascular complications (diabetic nephropathy, neuropathy, and retinopathy) recorded prior to baseline date	Primary care and HES	0, 1, 2, or 3 complications	Non by definition
Diabetes treatment	Diabetes treatment recorded within last 6 months prior to baseline date	Primary care	Insulin prescription, oral hypoglycaemic agent (OHA) prescriptions only, or no treatment	Non by definition
Comorbidities	Record of comorbidity with diagnosis date prior to baseline date. Comorbidities studied covered cardiovascular (hypertension, atrial fibrillation, angina, previous myocardial infarction, previous cardiac revascularisation, other ischaemic heart disease, heart failure, peripheral vascular disease), respiratory (asthma, chronic obstructive pulmonary disease), neurological (previous transient ischaemic attack, previous stroke, dementia, or other neurological condition), oncological (haematological cancer, solid cancer), and other conditions (chronic liver disease, solid organ transplant)	Primary care and HES	Yes, or no	Non by definition
Chronic kidney disease stage	Stage defined from estimated glomerular filtration rate (eGFR), stage 5 also ascertained by coded record prior to baseline [5]	Primary care and HES	Stage 1, stage 2, stage 3a, stage 3b, stage 4, stage 5, or missing	Missing indicator method
Albumin creatinine ratio	Defined from latest albumin creatinine ratio measurement prior to baseline	Primary care	A1, A2, A3, or missing	Missing indicator method
Recent hospitalisation for respiratory infection	A hospitalisation recorded with a code for respiratory infection in the 2 years prior to baseline	HES	Yes, or no	Non by definition
Recent hospitalisation for other causes	A hospitalisation recorded for any other cause (excluding respiratory infection) in the 2 years prior to baseline	HES	Yes, or no	Non by definition
Additional respiratory infections	Respiratory conditions previously included in the 2020 QCOVID risk prediction algorithm [6] (pulmonary fibrosis, pulmonary hypertension, bronchiectasis) with diagnosis occurring before the baseline date	Primary care and HES	Yes, or no	Non by definition
Other medications	Medications previously included in 2020 QCOVID risk prediction algorithm [6] (immunosuppressants, leukotrienes, long acting β agonists, oral steroids) with a prescription issued within 6 months prior to baseline	Primary care	Yes, or no	Non by definition

For peer review only

Supplemental table 3. Frequency of ICD-10 codes recorded in hospitalisation outcomes in type 2 diabetes for Covid-19 (2020), influenza (2016-2019), and pneumonia (2016-2019).

**Covid-19**		
**ICD10 code**	**Description**	**Frequency**
U07.1	COVID-19, confirmed by lab testing	4972
U07.2	COVID-19, not confirmed by lab testing (includes clinically-epidemiologically diagnosed, probable and suspected)	1073
**Influenza**		
**ICD10 code**	**Description**	**Frequency**
J10.1	Influenza with other respiratory manifestations, seasonal influenza virus identified	1645
J10.0	Influenza with pneumonia, seasonal influenza virus identified	1158
J11.1	Influenza with other respiratory manifestations, virus not identified	250
J10.8	Influenza with other manifestations, seasonal influenza virus identified	134
J11.0	Influenza with pneumonia, virus not identified	101
J09	Influenza due to identified zoonotic or pandemic influenza virus	11
J11.8	Influenza with other manifestations, virus not identified	10
**Pneumonia**		
**ICD10 code**	**Description**	**Frequency**
J18.1	Lobar pneumonia, unspecified	20367
J18.9	Pneumonia, unspecified	16382
J18.0	Bronchopneumonia, unspecified	885
J13	Pneumonia due to Streptococcus pneumoniae	407
J14	Pneumonia due to Haemophilus influenzae	138
J15.0	Pneumonia due to Klebsiella pneumoniae	137
J15.1	Pneumonia due to Pseudomonas	116
J15.2	Pneumonia due to staphylococcus	88
J15.4	Pneumonia due to other streptococci	73
J12.1	Respiratory syncytial virus pneumonia	71
J15.6	Pneumonia due to other Gram-negative bacteria	53
J15.9	Bacterial pneumonia, unspecified	53
J12.3	Human metapneumovirus pneumonia	46
J15.5	Pneumonia due to Escherichia coli	43
J17.2	Pneumonia in mycoses	41
J12.2	Parainfluenza virus pneumonia	35
J12.9	Viral pneumonia, unspecified	34
J17.3	Pneumonia in parasitic diseases	34
J18.8	Other pneumonia, organism unspecified	29
J15.8	Other bacterial pneumonia	24
J18.2	Hypostatic pneumonia, unspecified	22
J12.8	Other viral pneumonia	21
J15.3	Pneumonia due to streptococcus, group B	14
J15.7	Pneumonia due to Mycoplasma pneumoniae	14
J17.1	Pneumonia in viral diseases classified elsewhere	9
J12.0	Adenoviral pneumonia	6
J17.0	Pneumonia in bacterial diseases classified elsewhere	2
J16.0	Chlamydial pneumonia	1
J16.8	Pneumonia due to other specified infectious organisms	1

For peer review only

Supplemental table 4. Full baseline characteristics and Covid-19 (2020), influenza (2016-2019), and pneumonia (2016-2019) hospitalisations in type 2 diabetes.

	2020 cohort	Covid-19 hospitalisations (in 2020 cohort)	2016 cohort	Influenza hospitalisations (in 2016 cohort)	Pneumonia hospitalisations (in 2016 cohort)
**Number of individuals**	584854	5965	585289	3226	38088
**Mean (SD) follow-up, days**		265.0 (38.3)		915.2 (224.8)	897.4 (246.3)
**Sex**					
Female	253360 (43.3)	2317 (38.8)	256656 (43.9)	1581 (49.0)	17140 (45.0)
Male	331494 (56.7)	3648 (61.2)	328633 (56.1)	1645 (51.0)	20948 (55.0)
**Age group, years**					
<40	12898 (2.2)	70 (1.2)	13468 (2.3)	46 (1.4)	164 (0.4)
40-49	46075 (7.9)	258 (4.3)	49649 (8.5)	128 (4.0)	717 (1.9)
50-59	114537 (19.6)	729 (12.2)	113285 (19.4)	329 (10.2)	2495 (6.6)
60-69	151493 (25.9)	1198 (20.1)	153782 (26.3)	679 (21.0)	6068 (15.9)
70-79	155424 (26.6)	1660 (27.8)	152401 (26.0)	1078 (33.4)	12073 (31.7)
80-89	89871 (15.4)	1671 (28.0)	88492 (15.1)	811 (25.1)	13324 (35.0)
90+	14556 (2.5)	379 (6.4)	14212 (2.4)	155 (4.8)	3247 (8.5)
**Ethnicity**					
White	445160 (76.1)	4200 (70.4)	457714 (78.2)	2586 (80.2)	33206 (87.2)
South Asian	77253 (13.2)	891 (14.9)	71050 (12.1)	403 (12.5)	2935 (7.7)
Black	35144 (6.0)	645 (10.8)	33175 (5.7)	156 (4.8)	1324 (3.5)
Other	9886 (1.7)	144 (2.4)	8178 (1.4)	35 (1.1)	306 (0.8)
Mixed	6267 (1.1)	69 (1.2)	5654 (1.0)	37 (1.1)	246 (0.6)
Unknown	11144 (1.9)	16 (0.3)	9518 (1.6)	9 (0.3)	71 (0.2)
**Index of multiple deprivation quintile**					
1 (least deprived)	102950 (17.6)	761 (12.8)	103184 (17.6)	502 (15.6)	6296 (16.5)
2	106968 (18.3)	921 (15.4)	109006 (18.6)	548 (17.0)	6923 (18.2)
3	113454 (19.4)	1088 (18.2)	113661 (19.4)	601 (18.6)	7264 (19.1)
4	127048 (21.7)	1446 (24.2)	125492 (21.4)	698 (21.6)	8199 (21.5)
5 (most deprived)	134146 (22.9)	1747 (29.3)	133563 (22.8)	873 (27.1)	9380 (24.6)
Missing	288 (0.0)	2 (0.0)	383 (0.1)	4 (0.1)	26 (0.1)
**Duration of diagnosed diabetes, years**					
<1	14311 (2.4)	67 (1.1)	36503 (6.2)	146 (4.5)	1401 (3.7)
1-2	60219 (10.3)	397 (6.7)	77510 (13.2)	306 (9.5)	3324 (8.7)
3-5	105094 (18.0)	796 (13.3)	111199 (19.0)	491 (15.2)	5218 (13.7)
6-9	128817 (22.0)	1116 (18.7)	125083 (21.4)	621 (19.2)	7088 (18.6)
10-14	124471 (21.3)	1250 (21.0)	126421 (21.6)	771 (23.9)	9620 (25.3)
15-19	89704 (15.3)	1225 (20.5)	62373 (10.7)	465 (14.4)	5764 (15.1)
20+	62238 (10.6)	1114 (18.7)	46200 (7.9)	426 (13.2)	5673 (14.9)
**HbA1c, mmol/mol**					
<48	167739 (28.7)	1679 (28.1)	175659 (30.0)	934 (29.0)	12271 (32.2)
48-53	116015 (19.8)	1054 (17.7)	116131 (19.8)	557 (17.3)	7015 (18.4)
53-64	147209 (25.2)	1352 (22.7)	142662 (24.4)	791 (24.5)	8912 (23.4)
64-75	66071 (11.3)	767 (12.9)	62291 (10.6)	412 (12.8)	4062 (10.7)
75-86	33970 (5.8)	408 (6.8)	32257 (5.5)	184 (5.7)	2191 (5.8)
86+	39727 (6.8)	580 (9.7)	37470 (6.4)	270 (8.4)	2646 (6.9)
Missing	14123 (2.4)	125 (2.1)	18819 (3.2)	78 (2.4)	991 (2.6)
**Number of microvascular complications**					
0	264011 (45.1)	1889 (31.7)	281811 (48.1)	1077 (33.4)	11930 (31.3)
1	216115 (37.0)	2140 (35.9)	204550 (34.9)	1256 (38.9)	14444 (37.9)
2	96948 (16.6)	1621 (27.2)	91671 (15.7)	789 (24.5)	10288 (27.0)
3	7780 (1.3)	315 (5.3)	7257 (1.2)	104 (3.2)	1426 (3.7)
**BMI, kg/m2**					
<18.5	3026 (0.5)	66 (1.1)	2801 (0.5)	16 (0.5)	497 (1.3)
18.5-24.9	86199 (14.7)	1009 (16.9)	79018 (13.5)	476 (14.8)	6985 (18.3)
25-29.9	184626 (31.6)	1747 (29.3)	178501 (30.5)	885 (27.4)	10950 (28.7)
30-34.9	151732 (25.9)	1350 (22.6)	151161 (25.8)	809 (25.1)	8249 (21.7)
35-39.9	74766 (12.8)	752 (12.6)	75339 (12.9)	405 (12.6)	3976 (10.4)
40+	48455 (8.3)	517 (8.7)	48685 (8.3)	302 (9.4)	2877 (7.6)
Missing	36050 (6.2)	524 (8.8)	49784 (8.5)	333 (10.3)	4554 (12.0)
**Smoking status**					
Active smoker	87220 (14.9)	556 (9.3)	90218 (15.4)	548 (17.0)	6259 (16.4)
Ex-smoker	336719 (57.6)	3847 (64.5)	316723 (54.1)	1852 (57.4)	22587 (59.3)
Non-smoker	138840 (23.7)	1288 (21.6)	149524 (25.5)	651 (20.2)	7305 (19.2)
Unknown	22075 (3.8)	274 (4.6)	28824 (4.9)	175 (5.4)	1937 (5.1)
**Comorbidities**					
**Cardiovascular**					
Hypertension	425278 (72.7)	5055 (84.7)	419645 (71.7)	2692 (83.4)	33013 (86.7)
Atrial fibrillation	70633 (12.1)	1413 (23.7)	63872 (10.9)	727 (22.5)	10486 (27.5)
Angina	87970 (15.0)	1526 (25.6)	91026 (15.6)	883 (27.4)	10973 (28.8)
Previous myocardial infarction	63738 (10.9)	1249 (20.9)	60576 (10.3)	645 (20.0)	8239 (21.6)
Previous cardiac revascularisation	57332 (9.8)	949 (15.9)	54961 (9.4)	497 (15.4)	5926 (15.6)
Other ischaemic heart disease	121776 (20.8)	2102 (35.2)	121753 (20.8)	1170 (36.3)	14645 (38.5)

For peer review only - http://bmjopen.bmj.com/site/about/guidelines.xhtml

	2020 cohort	Covid-19 hospitalisations (in 2020 cohort)	2016 cohort	Influenza hospitalisations (in 2016 cohort)	Pneumonia hospitalisations (in 2016 cohort)
Heart failure	61014 (10.4)	1573 (26.4)	53579 (9.2)	735 (22.8)	10402 (27.3)
Peripheral arterial disease	50045 (8.6)	1157 (19.4)	48489 (8.3)	481 (14.9)	7700 (20.2)
**Recent hospitalisation**					
Respiratory infection	25535 (4.4)	1049 (17.6)	21353 (3.6)	436 (13.5)	6127 (16.1)
Anything else	96421 (16.5)	2426 (40.7)	95232 (16.3)	1195 (37.0)	15007 (39.4)
**Respiratory**					
Asthma	125582 (21.5)	1567 (26.3)	118207 (20.2)	1063 (33.0)	11229 (29.5)
Chronic obstructive pulmonary disease	65615 (11.2)	1273 (21.3)	60108 (10.3)	806 (25.0)	10948 (28.7)
**Neurological**					
Previous transient ischaemic attack	31024 (5.3)	598 (10.0)	29997 (5.1)	294 (9.1)	4200 (11.0)
Previous stroke	49873 (8.5)	987 (16.5)	47721 (8.2)	504 (15.6)	6839 (18.0)
Dementia	20629 (3.5)	718 (12.0)	19548 (3.3)	226 (7.0)	3710 (9.7)
Other neurological condition	35000 (6.0)	741 (12.4)	33569 (5.7)	352 (10.9)	4967 (13.0)
**Oncological**					
Haematological cancer	11751 (2.0)	212 (3.6)	10352 (1.8)	155 (4.8)	1499 (3.9)
Solid cancer	79179 (13.5)	1130 (18.9)	72001 (12.3)	544 (16.9)	7887 (20.7)
**Other**					
Solid organ transplant	3682 (0.6)	99 (1.7)	3013 (0.5)	49 (1.5)	509 (1.3)
Chronic liver disease	69136 (11.8)	860 (14.4)	48011 (8.2)	333 (10.3)	3387 (8.9)
**Chronic Kidney Disease (CKD) stage**					
Stage 1	165845 (28.4)	933 (15.6)	147798 (25.3)	448 (13.9)	3873 (10.2)
Stage 2	286696 (49.0)	2438 (40.9)	290821 (49.7)	1399 (43.4)	15243 (40.0)
Stage 3a	72539 (12.4)	1079 (18.1)	80222 (13.7)	642 (19.9)	8056 (21.2)
Stage 3b	33246 (5.7)	699 (11.7)	37256 (6.4)	372 (11.5)	5844 (15.3)
Stage 4	9006 (1.5)	301 (5.0)	10490 (1.8)	143 (4.4)	2323 (6.1)
Stage 5	12871 (2.2)	496 (8.3)	13365 (2.3)	205 (6.4)	2543 (6.7)
Missing	4651 (0.8)	19 (0.3)	5337 (0.9)	17 (0.5)	206 (0.5)
**Albumin creatinine ratio (ACR) category**					
A1	402847 (68.9)	3284 (55.1)	388839 (66.4)	1812 (56.2)	19926 (52.3)
A2	121002 (20.7)	1719 (28.8)	106879 (18.3)	845 (26.2)	11550 (30.3)
A3	21899 (3.7)	614 (10.3)	18817 (3.2)	252 (7.8)	3219 (8.5)
Missing	39106 (6.7)	348 (5.8)	70754 (12.1)	317 (9.8)	3393 (8.9)
**Diabetes treatment (last 6 months)**					
Insulin prescription	79286 (13.6)	1490 (25.0)	78755 (13.5)	742 (23.0)	8481 (22.3)
OHA prescription only	362651 (62.0)	3167 (53.1)	349629 (59.7)	1739 (53.9)	19713 (51.8)
No treatment	142917 (24.4)	1308 (21.9)	156905 (26.8)	745 (23.1)	9894 (26.0)

For peer review only

Supplemental figure 2. Association of potential risk factors with hospitalisation for Covid-19 (2020), influenza (2016-2019), and pneumonia (2016-2019) in type 2 diabetes.

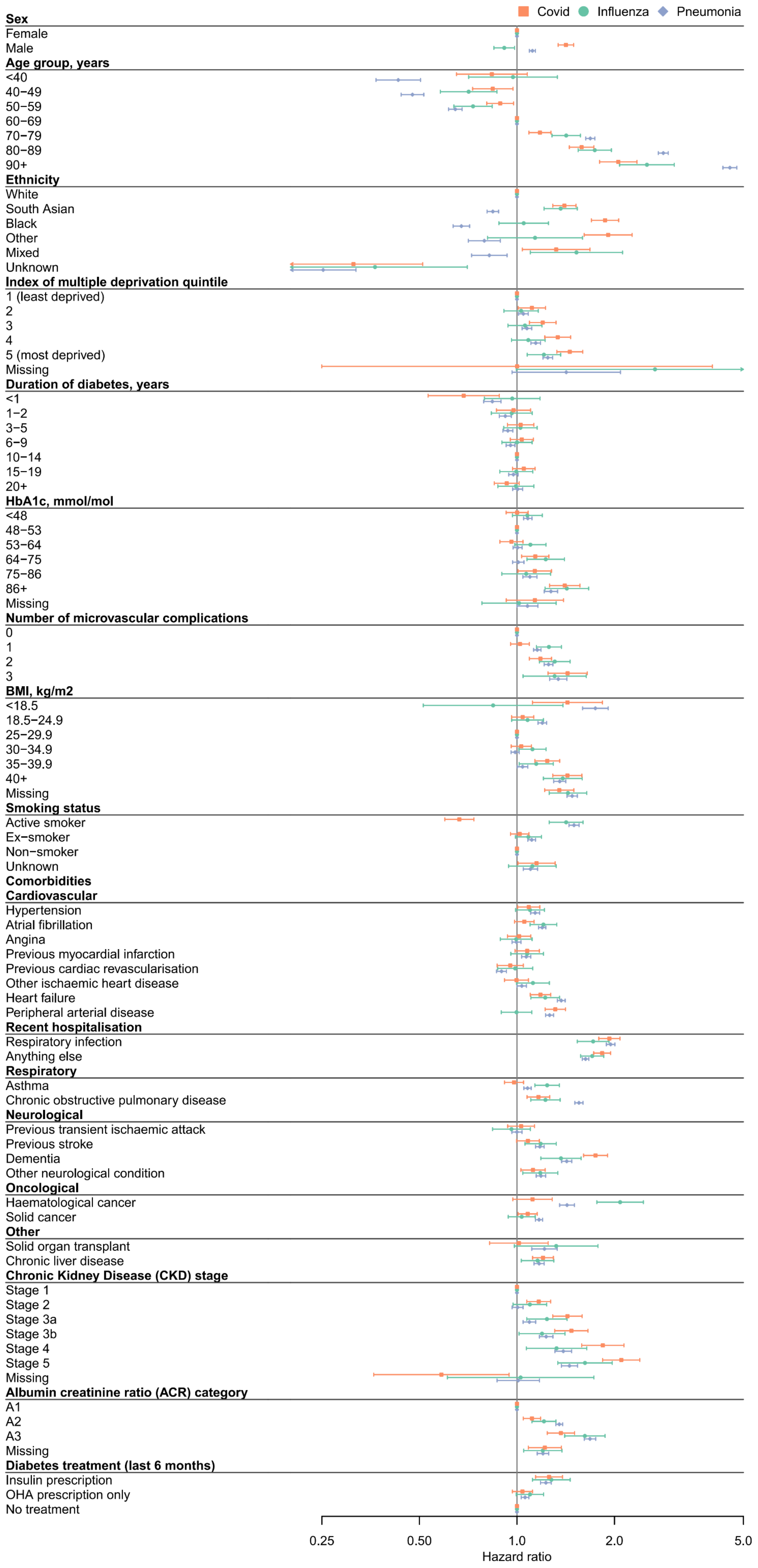

Supplemental table 5. Hazard ratio (HR) values and 95% confidence intervals (95% CI) for the association of potential risk factors with hospitalisation for Covid-19 (2020), influenza (2016-2019), and pneumonia (2016-2019) in type 2 diabetes.

	Covid-19		Influenza		Pneumonia	
	HR (95% CI)	p value	HR (95% CI)	p value	HR (95% CI)	p value
**Sex**						
Female	1 (ref)		1 (ref)		1 (ref)	
Male	1.42 (1.34-1.5)	<0.001	0.91 (0.85-0.98)	0.016	1.12 (1.09-1.14)	<0.001
**Age group, years**						
<40	0.84 (0.65-1.08)	0.165	0.97 (0.71-1.33)	0.862	0.43 (0.37-0.5)	<0.001
40-49	0.84 (0.73-0.97)	0.019	0.71 (0.58-0.87)	<0.001	0.48 (0.44-0.52)	<0.001
50-59	0.89 (0.81-0.98)	0.014	0.73 (0.64-0.84)	<0.001	0.65 (0.62-0.68)	<0.001
60-69	1 (ref)		1 (ref)		1 (ref)	
70-79	1.18 (1.09-1.27)	<0.001	1.42 (1.28-1.57)	<0.001	1.68 (1.63-1.74)	<0.001
80-89	1.58 (1.45-1.73)	<0.001	1.74 (1.54-1.96)	<0.001	2.83 (2.73-2.93)	<0.001
90+	2.05 (1.8-2.35)	<0.001	2.52 (2.07-3.06)	<0.001	4.54 (4.32-4.77)	<0.001
**Ethnicity**						
White	1 (ref)		1 (ref)		1 (ref)	
South Asian	1.4 (1.29-1.52)	<0.001	1.36 (1.21-1.54)	<0.001	0.84 (0.81-0.88)	<0.001
Black	1.87 (1.7-2.06)	<0.001	1.05 (0.88-1.25)	0.593	0.67 (0.64-0.71)	<0.001
Other	1.91 (1.61-2.27)	<0.001	1.14 (0.81-1.59)	0.456	0.79 (0.71-0.89)	<0.001
Mixed	1.32 (1.04-1.68)	0.023	1.53 (1.1-2.12)	0.011	0.82 (0.72-0.93)	0.002
Unknown	0.31 (0.19-0.51)	<0.001	0.36 (0.19-0.7)	0.003	0.25 (0.2-0.32)	<0.001
**Index of multiple deprivation quintile**						
1 (least deprived)	1 (ref)		1 (ref)		1 (ref)	
2	1.11 (1.01-1.23)	0.031	1.03 (0.91-1.16)	0.637	1.05 (1.01-1.08)	0.009
3	1.2 (1.09-1.32)	<0.001	1.06 (0.94-1.19)	0.357	1.07 (1.04-1.11)	<0.001
4	1.34 (1.22-1.46)	<0.001	1.08 (0.96-1.22)	0.185	1.14 (1.1-1.18)	<0.001
5 (most deprived)	1.46 (1.33-1.6)	<0.001	1.21 (1.08-1.36)	0.002	1.25 (1.2-1.29)	<0.001
Missing	1 (0.25-4.01)	0.999	2.67 (1-7.14)	0.051	1.42 (0.97-2.09)	0.075
**Duration of diagnosed diabetes, years**						
<1	0.68 (0.53-0.88)	0.003	0.97 (0.79-1.18)	0.739	0.84 (0.79-0.89)	<0.001
1-2	0.98 (0.86-1.1)	0.693	0.96 (0.83-1.11)	0.619	0.92 (0.88-0.96)	<0.001
3-5	1.03 (0.93-1.13)	0.585	1.03 (0.91-1.15)	0.683	0.94 (0.9-0.97)	<0.001
6-9	1.04 (0.95-1.12)	0.408	1 (0.9-1.11)	0.992	0.96 (0.93-0.99)	0.004
10-14	1 (ref)		1 (ref)		1 (ref)	
15-19	1.05 (0.97-1.14)	0.232	1 (0.89-1.12)	0.935	0.97 (0.94-1.01)	0.135
20+	0.93 (0.85-1.02)	0.108	0.99 (0.87-1.13)	0.906	1.01 (0.97-1.04)	0.774
**HbA1c, mmol/mol**						
<48	1 (0.93-1.08)	0.975	1.08 (0.97-1.2)	0.172	1.08 (1.05-1.11)	<0.001
48-53	1 (ref)		1 (ref)		1 (ref)	
53-64	0.96 (0.89-1.04)	0.349	1.1 (0.99-1.23)	0.09	1 (0.97-1.04)	0.776
64-75	1.14 (1.03-1.25)	0.009	1.23 (1.07-1.4)	0.003	1.01 (0.97-1.05)	0.642
75-86	1.13 (1.01-1.28)	0.037	1.07 (0.9-1.27)	0.457	1.1 (1.04-1.15)	<0.001
86+	1.4 (1.26-1.56)	<0.001	1.43 (1.22-1.66)	<0.001	1.27 (1.21-1.33)	<0.001
Missing	1.14 (0.93-1.39)	0.22	1.01 (0.78-1.32)	0.913	1.08 (1-1.16)	0.048
**Number of microvascular complications**						
0	1 (ref)		1 (ref)		1 (ref)	
1	1.02 (0.96-1.09)	0.527	1.26 (1.15-1.37)	<0.001	1.16 (1.13-1.19)	<0.001
2	1.18 (1.09-1.28)	<0.001	1.31 (1.17-1.46)	<0.001	1.25 (1.21-1.29)	<0.001
3	1.43 (1.25-1.65)	<0.001	1.31 (1.04-1.64)	0.019	1.34 (1.26-1.43)	<0.001
**BMI, kg/m2**						
<18.5	1.43 (1.12-1.84)	0.005	0.84 (0.51-1.39)	0.504	1.75 (1.59-1.91)	<0.001
18.5-24.9	1.04 (0.96-1.13)	0.312	1.08 (0.96-1.21)	0.19	1.2 (1.16-1.23)	<0.001
25-29.9	1 (ref)		1 (ref)		1 (ref)	
30-34.9	1.03 (0.96-1.11)	0.397	1.12 (1.01-1.23)	0.025	0.99 (0.96-1.02)	0.383
35-39.9	1.24 (1.14-1.35)	<0.001	1.15 (1.02-1.29)	0.026	1.04 (1-1.08)	0.035
40+	1.43 (1.29-1.59)	<0.001	1.39 (1.21-1.59)	<0.001	1.35 (1.3-1.41)	<0.001
Missing	1.35 (1.22-1.5)	<0.001	1.44 (1.26-1.64)	<0.001	1.48 (1.43-1.54)	<0.001
**Smoking status**						
Active smoker	0.66 (0.6-0.74)	<0.001	1.42 (1.26-1.6)	<0.001	1.5 (1.45-1.55)	<0.001
Ex-smoker	1.02 (0.96-1.09)	0.552	1.09 (0.99-1.19)	0.083	1.11 (1.08-1.14)	<0.001
Non-smoker	1 (ref)		1 (ref)		1 (ref)	
Unknown	1.15 (1.01-1.31)	0.039	1.12 (0.94-1.32)	0.204	1.1 (1.05-1.16)	<0.001
**Comorbidities**						
**Cardiovascular**						
Hypertension	1.09 (1.01-1.18)	0.037	1.1 (0.99-1.22)	0.077	1.14 (1.1-1.17)	<0.001
Atrial fibrillation	1.05 (0.98-1.13)	0.139	1.21 (1.1-1.33)	<0.001	1.2 (1.17-1.23)	<0.001
Angina	1.02 (0.93-1.1)	0.714	0.99 (0.89-1.11)	0.927	1 (0.97-1.03)	0.836
Previous myocardial infarction	1.08 (0.99-1.17)	0.099	1.08 (0.96-1.21)	0.221	1.07 (1.03-1.1)	<0.001
Previous cardiac revascularisation	0.95 (0.87-1.05)	0.315	0.99 (0.87-1.12)	0.841	0.9 (0.86-0.93)	<0.001
Other ischaemic heart disease	1 (0.92-1.09)	0.938	1.12 (1-1.26)	0.057	1.03 (1-1.07)	0.041
Heart failure	1.18 (1.1-1.27)	<0.001	1.22 (1.1-1.35)	<0.001	1.37 (1.33-1.41)	<0.001
Peripheral arterial disease	1.31 (1.22-1.41)	<0.001	1 (0.89-1.11)	0.957	1.26 (1.23-1.3)	<0.001
**Recent hospitalisation**						
Respiratory infection	1.93 (1.79-2.08)	<0.001	1.72 (1.54-1.92)	<0.001	1.95 (1.89-2.01)	<0.001
Anything else	1.83 (1.73-1.95)	<0.001	1.71 (1.57-1.85)	<0.001	1.63 (1.59-1.67)	<0.001

For peer review only - http://bmjopen.bmj.com/site/about/guidelines.xhtml

	Covid-19		Influenza		Pneumonia	
	HR (95% CI)	p value	HR (95% CI)	p value	HR (95% CI)	p value
**Respiratory**						
Asthma	0.98 (0.92-1.05)	0.547	1.24 (1.14-1.35)	<0.001	1.08 (1.05-1.11)	<0.001
Chronic obstructive pulmonary disease	1.16 (1.07-1.26)	<0.001	1.22 (1.1-1.36)	<0.001	1.55 (1.51-1.6)	<0.001
**Neurological**						
Previous transient ischaemic attack	1.03 (0.94-1.13)	0.543	0.96 (0.84-1.1)	0.568	1 (0.96-1.04)	0.991
Previous stroke	1.08 (1-1.17)	0.06	1.18 (1.06-1.32)	0.003	1.18 (1.14-1.21)	<0.001
Dementia	1.75 (1.61-1.9)	<0.001	1.37 (1.19-1.58)	<0.001	1.42 (1.37-1.48)	<0.001
Other neurological condition	1.12 (1.03-1.22)	0.009	1.18 (1.04-1.34)	0.009	1.19 (1.15-1.23)	<0.001
**Oncological**						
Haematological cancer	1.12 (0.97-1.29)	0.121	2.08 (1.76-2.46)	<0.001	1.43 (1.35-1.5)	<0.001
Solid cancer	1.08 (1.01-1.15)	0.027	1.03 (0.94-1.14)	0.49	1.17 (1.14-1.2)	<0.001
**Other**						
Solid organ transplant	1.01 (0.82-1.25)	0.897	1.32 (0.98-1.78)	0.066	1.22 (1.11-1.33)	<0.001
Chronic liver disease	1.2 (1.12-1.3)	<0.001	1.16 (1.03-1.3)	0.013	1.17 (1.13-1.21)	<0.001
**Chronic Kidney Disease (CKD) stage**						
Stage 1	1 (ref)		1 (ref)		1 (ref)	
Stage 2	1.17 (1.07-1.27)	<0.001	1.1 (0.97-1.24)	0.132	1 (0.97-1.04)	0.827
Stage 3a	1.43 (1.29-1.59)	<0.001	1.24 (1.07-1.43)	0.004	1.09 (1.04-1.14)	<0.001
Stage 3b	1.47 (1.31-1.66)	<0.001	1.19 (1.01-1.41)	0.033	1.23 (1.17-1.29)	<0.001
Stage 4	1.84 (1.58-2.14)	<0.001	1.33 (1.07-1.64)	0.01	1.39 (1.31-1.48)	<0.001
Stage 5	2.1 (1.84-2.39)	<0.001	1.62 (1.34-1.97)	<0.001	1.45 (1.37-1.54)	<0.001
Missing	0.58 (0.36-0.95)	0.029	1.03 (0.61-1.73)	0.921	1.01 (0.87-1.17)	0.903
**Albumin creatinine ratio (ACR) category**						
A1	1 (ref)		1 (ref)		1 (ref)	
A2	1.11 (1.05-1.18)	<0.001	1.21 (1.11-1.32)	<0.001	1.35 (1.32-1.38)	<0.001
A3	1.37 (1.24-1.51)	<0.001	1.62 (1.4-1.87)	<0.001	1.68 (1.61-1.75)	<0.001
Missing	1.22 (1.08-1.37)	<0.001	1.2 (1.05-1.38)	0.008	1.2 (1.15-1.25)	<0.001
**Diabetes treatment (last 6 months)**						
Insulin prescription	1.26 (1.14-1.38)	<0.001	1.28 (1.12-1.46)	<0.001	1.23 (1.18-1.28)	<0.001
OHA prescription only	1.04 (0.97-1.12)	0.286	1.1 (1-1.21)	0.062	1.06 (1.03-1.09)	<0.001
No treatment	1 (ref)		1 (ref)		1 (ref)	

For peer review only

Supplemental figure 3A. Association of continuous HbA1c with hospitalisation for Covid-19 (2020), influenza (2016-2019), and pneumonia (2016-2019) in type 2 diabetes by ethnicity subgroup. Density plots show the distribution of HbA1c in each group.

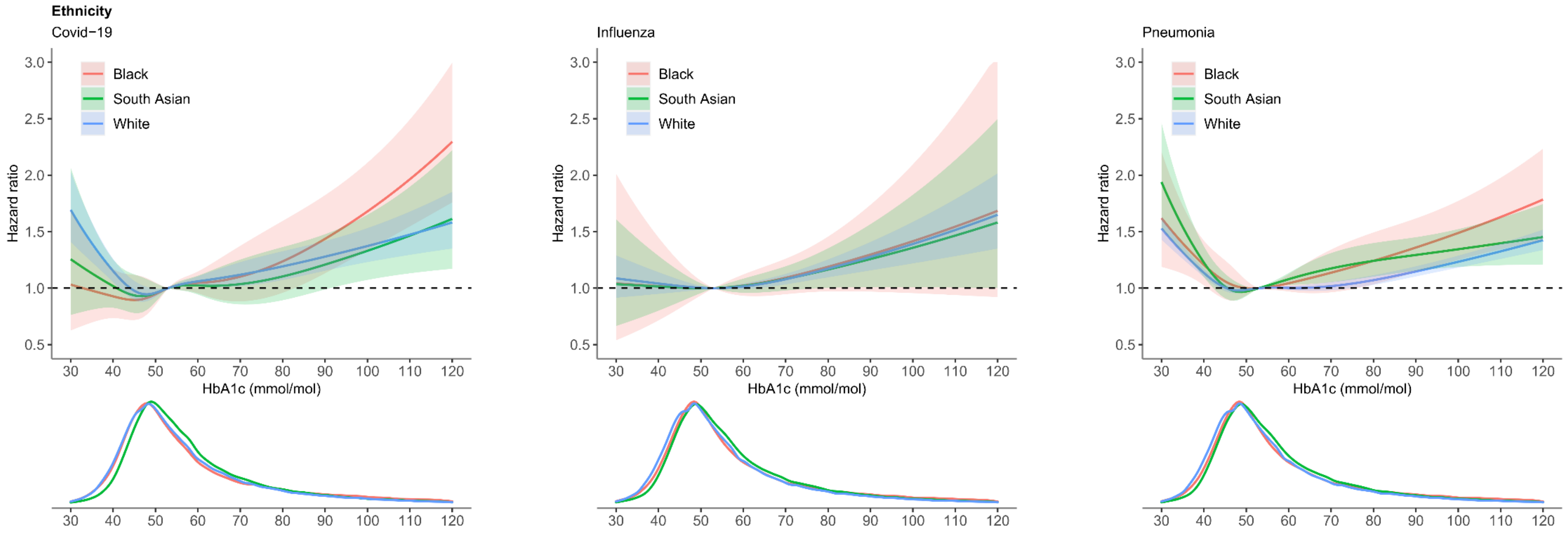

For peer

Supplemental figure 3B. Association of continuous BMI with hospitalisation for Covid-19 (2020), influenza (2016-2019), and pneumonia (2016-2019) in type 2 diabetes by ethnicity subgroup. Density plots show the distribution of BMI in each group.

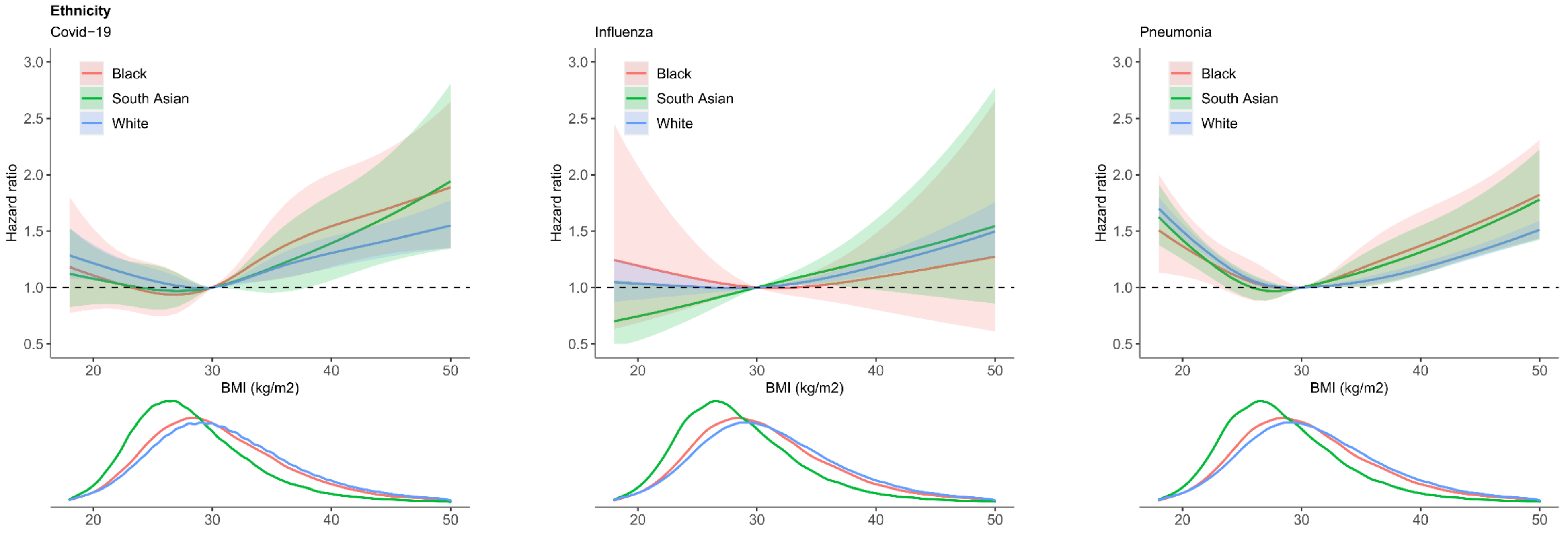

For peer review only - http://bmjopen.bmj.com/site/about/guidelines.xhtml

Supplemental table 6. Baseline characteristics and Covid-19 (2020) and pneumonia (2016-2019) deaths in type 2 diabetes.

	2020 cohort	Covid-19 deaths (in 2020 cohort)	2016 cohort	Pneumonia deaths (in 2016 cohort)
**Number of individuals**	584854	2635	585289	12652
**Mean (SD) follow-up, days**		266.1 (35.5)		917.0 (223.2)
**Sex**				
Female	253360 (43.3)	1023 (38.8)	256656 (43.9)	5499 (43.5)
Male	331494 (56.7)	1612 (61.2)	328633 (56.1)	7153 (56.5)
**Age group, years**				
<40	12898 (2.2)	7 (0.3)	13468 (2.3)	9 (0.1)
40-49	46075 (7.9)	38 (1.4)	49649 (8.5)	91 (0.7)
50-59	114537 (19.6)	150 (5.7)	113285 (19.4)	404 (3.2)
60-69	151493 (25.9)	381 (14.5)	153782 (26.3)	1401 (11.1)
70-79	155424 (26.6)	729 (27.7)	152401 (26.0)	3667 (29.0)
80-89	89871 (15.4)	1047 (39.7)	88492 (15.1)	5370 (42.4)
90+	14556 (2.5)	283 (10.7)	14212 (2.4)	1710 (13.5)
**Ethnicity**				
White	445160 (76.1)	1944 (73.8)	457714 (78.2)	11398 (90.1)
South Asian	77253 (13.2)	341 (12.9)	71050 (12.1)	748 (5.9)
Black	35144 (6.0)	267 (10.1)	33175 (5.7)	326 (2.6)
Other	9886 (1.7)	52 (2.0)	8178 (1.4)	86 (0.7)
Mixed	6267 (1.1)	21 (0.8)	5654 (1.0)	66 (0.5)
Unknown	11144 (1.9)	10 (0.4)	9518 (1.6)	28 (0.2)
**Index of multiple deprivation quintile**				
1 (least deprived)	102950 (17.6)	371 (14.1)	103184 (17.6)	2177 (17.2)
2	106968 (18.3)	418 (15.9)	109006 (18.6)	2378 (18.8)
3	113454 (19.4)	488 (18.5)	113661 (19.4)	2481 (19.6)
4	127048 (21.7)	607 (23.0)	125492 (21.4)	2708 (21.4)
5 (most deprived)	134146 (22.9)	749 (28.4)	133563 (22.8)	2904 (23.0)
Missing	288 (0.0)	2 (0.1)	383 (0.1)	4 (0.0)
**Duration of diagnosed diabetes, years**				
<1	14311 (2.4)	13 (0.5)	36503 (6.2)	331 (2.6)
1-2	60219 (10.3)	120 (4.6)	77510 (13.2)	845 (6.7)
3-5	105094 (18.0)	315 (12.0)	111199 (19.0)	1553 (12.3)
6-9	128817 (22.0)	438 (16.6)	125083 (21.4)	2294 (18.1)
10-14	124471 (21.3)	562 (21.3)	126421 (21.6)	3349 (26.5)
15-19	89704 (15.3)	604 (22.9)	62373 (10.7)	2112 (16.7)
20+	62238 (10.6)	583 (22.1)	46200 (7.9)	2168 (17.1)
**HbA1c, mmol/mol**				
<48	167739 (28.7)	838 (31.8)	175659 (30.0)	4411 (34.9)
48-53	116015 (19.8)	453 (17.2)	116131 (19.8)	2370 (18.7)
53-64	147209 (25.2)	567 (21.5)	142662 (24.4)	2862 (22.6)
64-75	66071 (11.3)	349 (13.2)	62291 (10.6)	1269 (10.0)
75-86	33970 (5.8)	168 (6.4)	32257 (5.5)	642 (5.1)
86+	39727 (6.8)	189 (7.2)	37470 (6.4)	756 (6.0)
Missing	14123 (2.4)	71 (2.7)	18819 (3.2)	342 (2.7)
**Number of microvascular complications**				
0	264011 (45.1)	706 (26.8)	281811 (48.1)	3447 (27.2)
1	216115 (37.0)	957 (36.3)	204550 (34.9)	4804 (38.0)
2	96948 (16.6)	804 (30.5)	91671 (15.7)	3868 (30.6)
3	7780 (1.3)	168 (6.4)	7257 (1.2)	533 (4.2)
**BMI, kg/m2**				
<18.5	3026 (0.5)	47 (1.8)	2801 (0.5)	319 (2.5)
18.5-24.9	86199 (14.7)	587 (22.3)	79018 (13.5)	2900 (22.9)
25-29.9	184626 (31.6)	755 (28.7)	178501 (30.5)	3616 (28.6)
30-34.9	151732 (25.9)	526 (20.0)	151161 (25.8)	2236 (17.7)
35-39.9	74766 (12.8)	246 (9.3)	75339 (12.9)	968 (7.7)
40+	48455 (8.3)	166 (6.3)	48685 (8.3)	656 (5.2)
Missing	36050 (6.2)	308 (11.7)	49784 (8.5)	1957 (15.5)
**Smoking status**				
Active smoker	87220 (14.9)	234 (8.9)	90218 (15.4)	1937 (15.3)
Ex-smoker	336719 (57.6)	1742 (66.1)	316723 (54.1)	7553 (59.7)
Non-smoker	138840 (23.7)	551 (20.9)	149524 (25.5)	2478 (19.6)
Unknown	22075 (3.8)	108 (4.1)	28824 (4.9)	684 (5.4)
**Comorbidities**				
**Cardiovascular**				
Hypertension	425278 (72.7)	2334 (88.6)	419645 (71.7)	11173 (88.3)
Atrial fibrillation	70633 (12.1)	769 (29.2)	63872 (10.9)	4175 (33.0)
Angina	87970 (15.0)	749 (28.4)	91026 (15.6)	3842 (30.4)
Previous myocardial infarction	63738 (10.9)	633 (24.0)	60576 (10.3)	3064 (24.2)
Previous cardiac revascularisation	57332 (9.8)	462 (17.5)	54961 (9.4)	2035 (16.1)
Other ischaemic heart disease	121776 (20.8)	1022 (38.8)	121753 (20.8)	5196 (41.1)
Heart failure	61014 (10.4)	848 (32.2)	53579 (9.2)	4195 (33.2)
Peripheral arterial disease	50045 (8.6)	635 (24.1)	48489 (8.3)	3060 (24.2)

For peer review only - http://bmjopen.bmj.com/site/about/guidelines.xhtml

	2020 cohort	Covid-19 deaths (in 2020 cohort)	2016 cohort	Pneumonia deaths (in 2016 cohort)
**Recent hospitalisation**				
Respiratory infection	25535 (4.4)	563 (21.4)	21353 (3.6)	2387 (18.9)
Anything else	96421 (16.5)	1171 (44.4)	95232 (16.3)	5409 (42.8)
**Respiratory**				
Asthma	125582 (21.5)	683 (25.9)	118207 (20.2)	3395 (26.8)
Chronic obstructive pulmonary disease	65615 (11.2)	642 (24.4)	60108 (10.3)	3755 (29.7)
**Neurological**				
Previous transient ischaemic attack	31024 (5.3)	334 (12.7)	29997 (5.1)	1597 (12.6)
Previous stroke	49873 (8.5)	570 (21.6)	47721 (8.2)	2591 (20.5)
Dementia	20629 (3.5)	572 (21.7)	19548 (3.3)	1831 (14.5)
Other neurological condition	35000 (6.0)	447 (17.0)	33569 (5.7)	1974 (15.6)
**Oncological**				
Haematological cancer	11751 (2.0)	119 (4.5)	10352 (1.8)	550 (4.3)
Solid cancer	79179 (13.5)	555 (21.1)	72001 (12.3)	2965 (23.4)
**Other**				
Solid organ transplant	3682 (0.6)	41 (1.6)	3013 (0.5)	193 (1.5)
Chronic liver disease	69136 (11.8)	322 (12.2)	48011 (8.2)	1034 (8.2)
**Chronic Kidney Disease (CKD) stage**				
Stage 1	165845 (28.4)	239 (9.1)	147798 (25.3)	988 (7.8)
Stage 2	286696 (49.0)	968 (36.7)	290821 (49.7)	4481 (35.4)
Stage 3a	72539 (12.4)	553 (21.0)	80222 (13.7)	2787 (22.0)
Stage 3b	33246 (5.7)	415 (15.7)	37256 (6.4)	2279 (18.0)
Stage 4	9006 (1.5)	186 (7.1)	10490 (1.8)	999 (7.9)
Stage 5	12871 (2.2)	261 (9.9)	13365 (2.3)	1043 (8.2)
Missing	4651 (0.8)	13 (0.5)	5337 (0.9)	75 (0.6)
**Albumin creatinine ratio (ACR) category**				
A1	402847 (68.9)	1271 (48.2)	388839 (66.4)	6044 (47.8)
A2	121002 (20.7)	845 (32.1)	106879 (18.3)	4334 (34.3)
A3	21899 (3.7)	322 (12.2)	18817 (3.2)	1281 (10.1)
Missing	39106 (6.7)	197 (7.5)	70754 (12.1)	993 (7.8)
**Diabetes treatment (last 6 months)**				
Insulin prescription	79286 (13.6)	682 (25.9)	78755 (13.5)	2772 (21.9)
OHA prescription only	362651 (62.0)	1289 (48.9)	349629 (59.7)	6309 (49.9)
No treatment	142917 (24.4)	664 (25.2)	156905 (26.8)	3571 (28.2)

For peer review only

Supplemental figure 4. Association of potential risk factors with Covid-19 (2020), and pneumonia (2016-2019) mortality in type 2 diabetes.

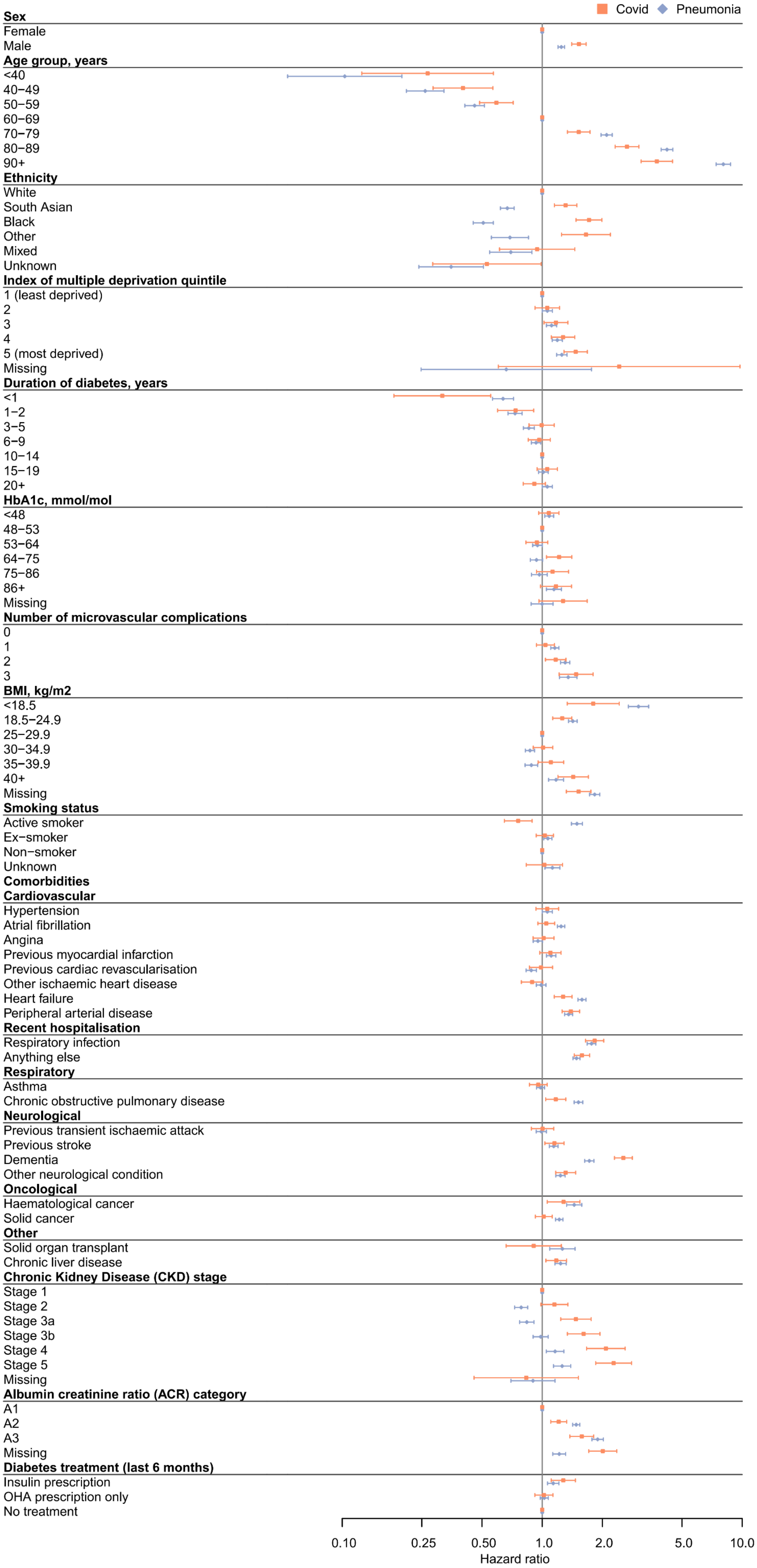

Supplemental table 7. Baseline characteristics and Covid-19 (2020), influenza (2016-2019), and pneumonia (2016-2019) hospitalisations in type 1 diabetes.

	2020 cohort	Covid-19 hospitalisations (in 2020 cohort)	2016 cohort	Influenza hospitalisations (in 2016 cohort)	Pneumonia hospitalisations (in 2016 cohort)
**Number of individuals**	43033	209	42488	208	1401
**Mean (SD) follow-up, days**		267.0 (33.2)		917.3 (223.0)	907.1 (236.2)
**Sex**					
Female	18172 (42.2)	76 (36.4)	17889 (42.1)	103 (49.5)	611 (43.6)
Male	24861 (57.8)	133 (63.6)	24599 (57.9)	105 (50.5)	790 (56.4)
**Age, years**					
<18	4458 (10.4)	1 (0.5)	4502 (10.6)	12 (5.8)	13 (0.9)
18-39	14454 (33.6)	34 (16.3)	13996 (32.9)	59 (28.4)	191 (13.6)
40-49	7480 (17.4)	31 (14.8)	7995 (18.8)	43 (20.7)	208 (14.8)
50-59	8056 (18.7)	42 (20.1)	7911 (18.6)	31 (14.9)	283 (20.2)
60-69	5079 (11.8)	43 (20.6)	4731 (11.1)	34 (16.3)	281 (20.1)
70-79	2689 (6.2)	37 (17.7)	2460 (5.8)	23 (11.1)	239 (17.1)
80+	817 (1.9)	21 (10.0)	893 (2.1)	6 (2.9)	186 (13.3)
**Ethnicity**					
White	38478 (89.4)	170 (81.3)	38337 (90.2)	187 (89.9)	1311 (93.6)
South Asian	1576 (3.7)	14 (6.7)	1494 (3.5)	14 (6.7)	37 (2.6)
Black	1385 (3.2)	16 (7.7)	1253 (2.9)	3 (1.4)	33 (2.4)
Other	464 (1.1)	3 (1.4)	373 (0.9)	0 (0.0)	5 (0.4)
Mixed	525 (1.2)	6 (2.9)	476 (1.1)	4 (1.9)	15 (1.1)
Unknown	605 (1.4)	0 (0.0)	555 (1.3)	0 (0.0)	0 (0.0)
**Index of multiple deprivation quintile**					
1 (least deprived)	9395 (21.8)	43 (20.6)	9251 (21.8)	28 (13.5)	254 (18.1)
2	8710 (20.2)	30 (14.4)	8652 (20.4)	37 (17.8)	237 (16.9)
3	8334 (19.4)	33 (15.8)	8347 (19.6)	44 (21.2)	276 (19.7)
4	8444 (19.6)	38 (18.2)	8342 (19.6)	45 (21.6)	292 (20.8)
5 (most deprived)	8128 (18.9)	65 (31.1)	7864 (18.5)	54 (26.0)	342 (24.4)
Missing	22 (0.1)	0 (0.0)	32 (0.1)	0 (0.0)	0 (0.0)
**Duration of diagnosed diabetes, years**					
<5	5655 (13.1)	8 (3.8)	6556 (15.4)	17 (8.2)	81 (5.8)
5-9	6194 (14.4)	13 (6.2)	6077 (14.3)	21 (10.1)	101 (7.2)
10-14	5609 (13.0)	23 (11.0)	5899 (13.9)	28 (13.5)	144 (10.3)
15-19	5466 (12.7)	27 (12.9)	5355 (12.6)	28 (13.5)	174 (12.4)
20-24	4621 (10.7)	25 (12.0)	4612 (10.9)	22 (10.6)	176 (12.6)
25-29	4101 (9.5)	27 (12.9)	3747 (8.8)	27 (13.0)	149 (10.6)
30+	11387 (26.5)	86 (41.1)	10242 (24.1)	65 (31.2)	576 (41.1)
**HbA1c, mmol/mol**					
<48	3104 (7.2)	12 (5.7)	2796 (6.6)	13 (6.2)	109 (7.8)
48-53	3232 (7.5)	13 (6.2)	2967 (7.0)	13 (6.2)	111 (7.9)
53-64	10995 (25.6)	50 (23.9)	10340 (24.3)	37 (17.8)	263 (18.8)
64-75	9951 (23.1)	36 (17.2)	10098 (23.8)	46 (22.1)	283 (20.2)
75-86	5891 (13.7)	30 (14.4)	5979 (14.1)	32 (15.4)	228 (16.3)
86+	6097 (14.2)	57 (27.3)	6351 (14.9)	45 (21.6)	327 (23.3)
Missing	3763 (8.7)	11 (5.3)	3957 (9.3)	22 (10.6)	80 (5.7)
**BMI, kg/m2**					
<18.5	1462 (3.4)	6 (2.9)	1267 (3.0)	1 (0.5)	37 (2.6)
18.5-24.9	13478 (31.3)	61 (29.2)	13132 (30.9)	70 (33.7)	465 (33.2)
25-29.9	12966 (30.1)	57 (27.3)	12606 (29.7)	54 (26.0)	372 (26.6)
30-34.9	6258 (14.5)	37 (17.7)	5704 (13.4)	24 (11.5)	181 (12.9)
35-39.9	1934 (4.5)	16 (7.7)	1735 (4.1)	11 (5.3)	78 (5.6)
40+	949 (2.2)	8 (3.8)	782 (1.8)	8 (3.8)	57 (4.1)
Missing	5986 (13.9)	24 (11.5)	7262 (17.1)	40 (19.2)	211 (15.1)

For peer review only

Supplemental figure 5. Association of potential risk factors with hospitalisation for Covid-19 (2020), influenza (2016-2019), and pneumonia (2016-2019) in type 1 diabetes.

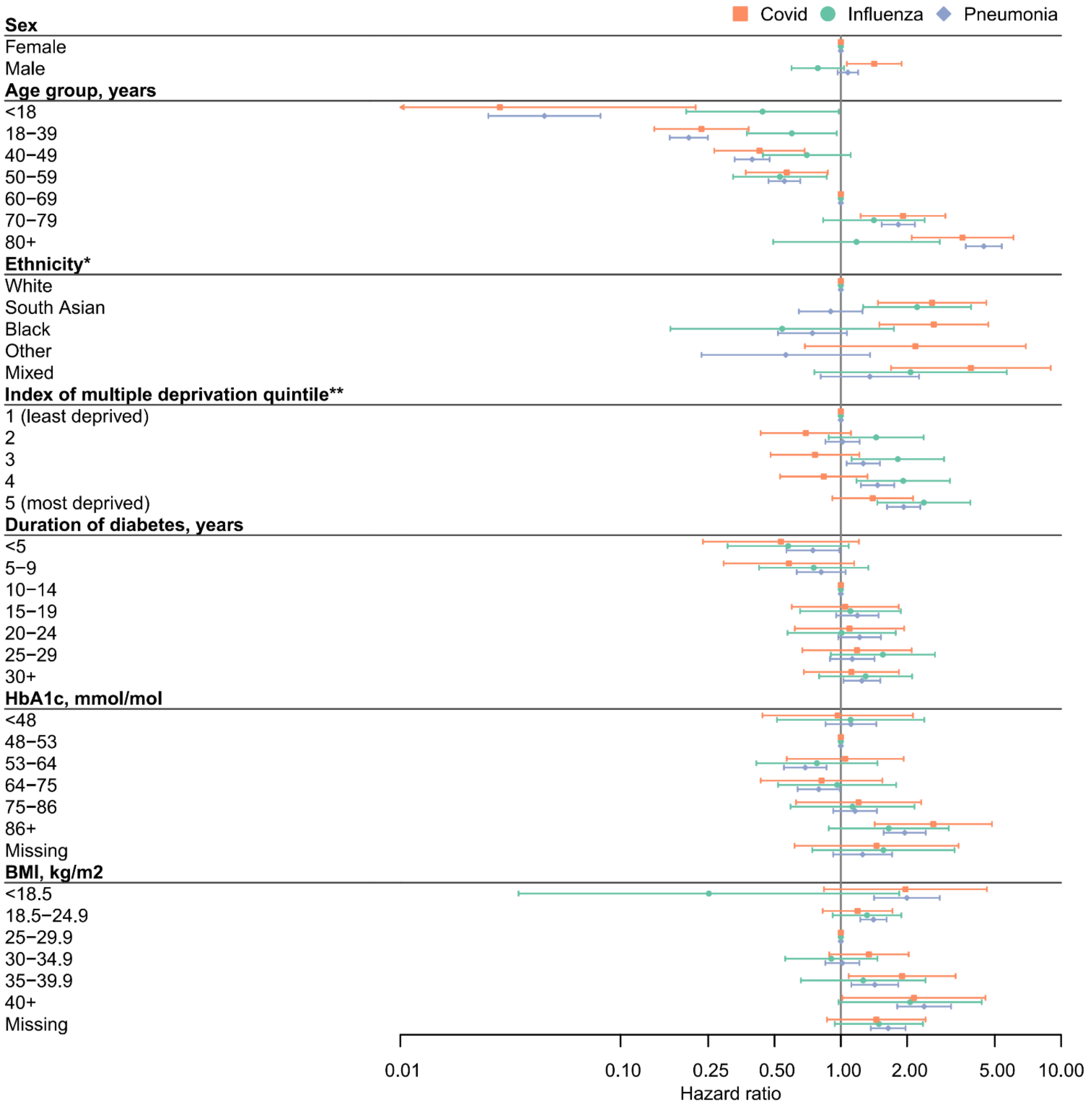

*No influenza hospitalisations occurred in other ethnicity group. No Covid-19, influenza or pneumonia hospitalisations occurred in unknown ethnicity group. ** No Covid-19, influenza or pneumonia hospitalisations occurred in missing IMD group.

For peer review only - http://bmjopen.bmj.com/site/about/guidelines.xhtml

Supplemental figure 6A. Association of continuous HbA1c with hospitalisation for Covid-19 (2020), influenza (2016-2019), and pneumonia (2016-2019) in type 1 diabetes. Density plots show the distribution of HbA1c in each group.

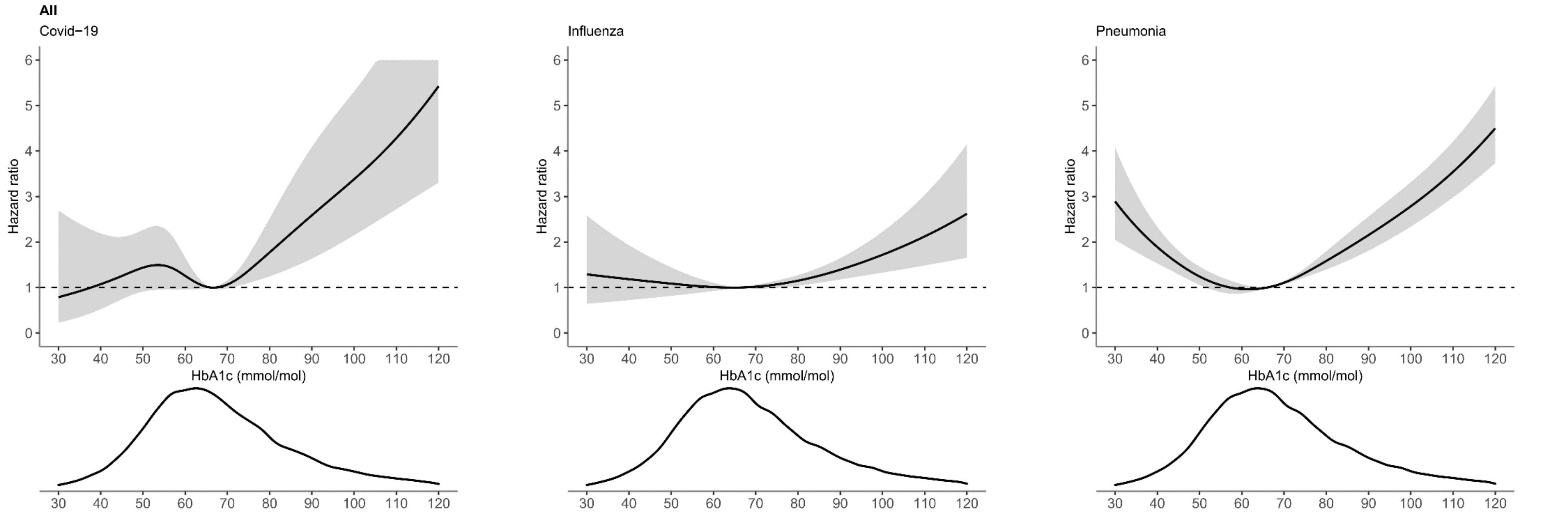

Supplemental figure 6B. Association of continuous BMI with hospitalisation for Covid-19 (2020), influenza (2016-2019), and pneumonia (2016-2019) in type 1 diabetes. Density plots show the distribution of BMI in each group.

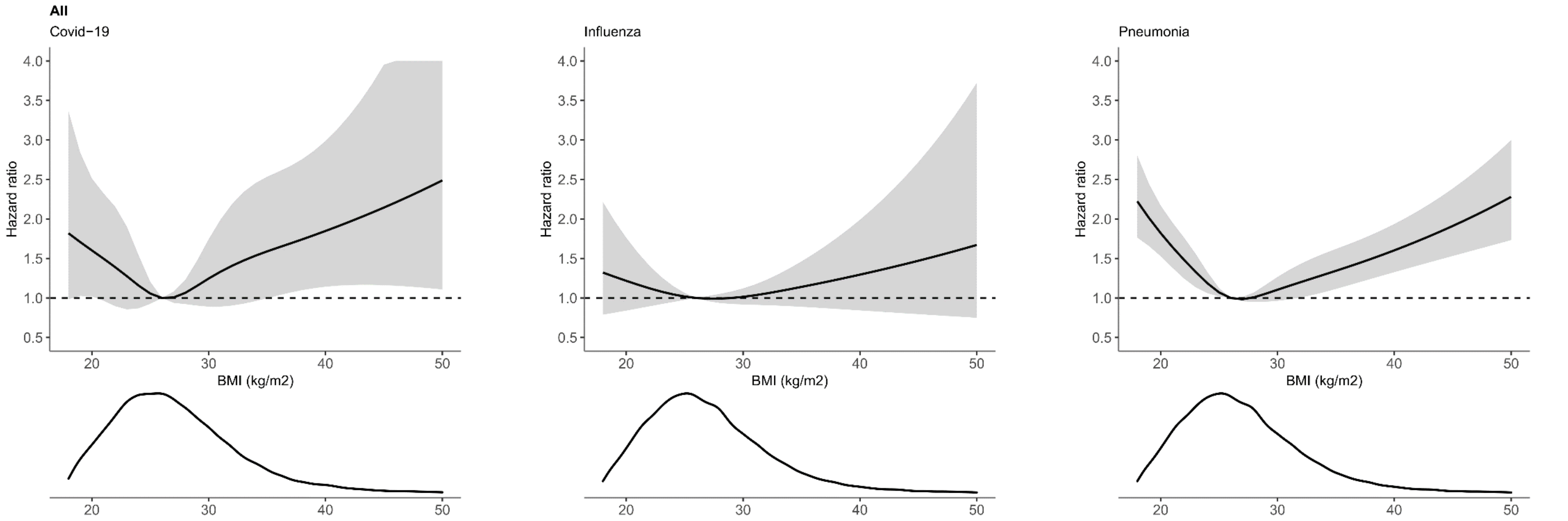

For peer review only - http://bmjopen.bmj.com/site/about/guidelines.xhtml

Supplemental table 8. Baseline characteristics and Covid-19, influenza, and pneumonia hospitalisations in type 1 diabetes, restricting hospitalisation definition to primary diagnosis in HES.

	2020 cohort	Covid-19 hospitalisations (in 2020 cohort)	2016 cohort	Influenza hospitalisations (in 2016 cohort)	Pneumonia hospitalisations (in 2016 cohort)
**Number of individuals**	43033	142	42488	130	803
**Mean (SD) follow-up, days**		267.2 (32.6)		917.9 (222.4)	912.1 (230.3)
**Sex**					
Female	18172 (42.2)	45 (31.7)	17889 (42.1)	68 (52.3)	353 (44.0)
Male	24861 (57.8)	97 (68.3)	24599 (57.9)	62 (47.7)	450 (56.0)
**Age, years**					
<18	4458 (10.4)	0 (0.0)	4502 (10.6)	6 (4.6)	9 (1.1)
18-39	14454 (33.6)	20 (14.1)	13996 (32.9)	35 (26.9)	102 (12.7)
40-49	7480 (17.4)	21 (14.8)	7995 (18.8)	22 (16.9)	115 (14.3)
50-59	8056 (18.7)	31 (21.8)	7911 (18.6)	21 (16.2)	154 (19.2)
60-69	5079 (11.8)	31 (21.8)	4731 (11.1)	25 (19.2)	157 (19.6)
70-79	2689 (6.2)	24 (16.9)	2460 (5.8)	17 (13.1)	148 (18.4)
80+	817 (1.9)	15 (10.6)	893 (2.1)	4 (3.1)	118 (14.7)
**Ethnicity**					
White	38478 (89.4)	111 (78.2)	38337 (90.2)	121 (93.1)	754 (93.9)
South Asian	1576 (3.7)	12 (8.5)	1494 (3.5)	8 (6.2)	17 (2.1)
Black	1385 (3.2)	12 (8.5)	1253 (2.9)	0 (0.0)	20 (2.5)
Other	464 (1.1)	2 (1.4)	373 (0.9)	0 (0.0)	3 (0.4)
Mixed	525 (1.2)	5 (3.5)	476 (1.1)	1 (0.8)	9 (1.1)
Unknown	605 (1.4)	0 (0.0)	555 (1.3)	0 (0.0)	0 (0.0)
**Index of multiple deprivation quintile**					
1 (least deprived)	9395 (21.8)	26 (18.3)	9251 (21.8)	22 (16.9)	157 (19.6)
2	8710 (20.2)	20 (14.1)	8652 (20.4)	21 (16.2)	143 (17.8)
3	8334 (19.4)	28 (19.7)	8347 (19.6)	27 (20.8)	163 (20.3)
4	8444 (19.6)	29 (20.4)	8342 (19.6)	26 (20.0)	141 (17.6)
5 (most deprived)	8128 (18.9)	39 (27.5)	7864 (18.5)	34 (26.2)	199 (24.8)
Missing	22 (0.1)	0 (0.0)	32 (0.1)	0 (0.0)	0 (0.0)
**Duration of diagnosed diabetes, years**					
<5	5655 (13.1)	4 (2.8)	6556 (15.4)	7 (5.4)	55 (6.8)
5-9	6194 (14.4)	10 (7.0)	6077 (14.3)	14 (10.8)	58 (7.2)
10-14	5609 (13.0)	18 (12.7)	5899 (13.9)	14 (10.8)	72 (9.0)
15-19	5466 (12.7)	21 (14.8)	5355 (12.6)	17 (13.1)	105 (13.1)
20-24	4621 (10.7)	11 (7.7)	4612 (10.9)	11 (8.5)	95 (11.8)
25-29	4101 (9.5)	22 (15.5)	3747 (8.8)	22 (16.9)	83 (10.3)
30+	11387 (26.5)	56 (39.4)	10242 (24.1)	45 (34.6)	335 (41.7)
**HbA1c, mmol/mol**					
<48	3104 (7.2)	8 (5.6)	2796 (6.6)	9 (6.9)	67 (8.3)
48-53	3232 (7.5)	8 (5.6)	2967 (7.0)	11 (8.5)	66 (8.2)
53-64	10995 (25.6)	35 (24.6)	10340 (24.3)	25 (19.2)	159 (19.8)
64-75	9951 (23.1)	28 (19.7)	10098 (23.8)	32 (24.6)	167 (20.8)
75-86	5891 (13.7)	22 (15.5)	5979 (14.1)	21 (16.2)	117 (14.6)
86+	6097 (14.2)	33 (23.2)	6351 (14.9)	22 (16.9)	179 (22.3)
Missing	3763 (8.7)	8 (5.6)	3957 (9.3)	10 (7.7)	48 (6.0)
**BMI, kg/m2**					
<18.5	1462 (3.4)	2 (1.4)	1267 (3.0)	1 (0.8)	23 (2.9)
18.5-24.9	13478 (31.3)	39 (27.5)	13132 (30.9)	45 (34.6)	267 (33.3)
25-29.9	12966 (30.1)	40 (28.2)	12606 (29.7)	35 (26.9)	215 (26.8)
30-34.9	6258 (14.5)	24 (16.9)	5704 (13.4)	16 (12.3)	95 (11.8)
35-39.9	1934 (4.5)	11 (7.7)	1735 (4.1)	7 (5.4)	53 (6.6)
40+	949 (2.2)	8 (5.6)	782 (1.8)	5 (3.8)	35 (4.4)
Missing	5986 (13.9)	18 (12.7)	7262 (17.1)	21 (16.2)	115 (14.3)

For peer review only

Supplemental figure 7. Association of potential risk factors with hospitalisation for Covid-19 (2020), influenza (2016-2019), and pneumonia (2016-2019) in type 1 diabetes, restricting hospitalisation definition to primary diagnosis in HES.

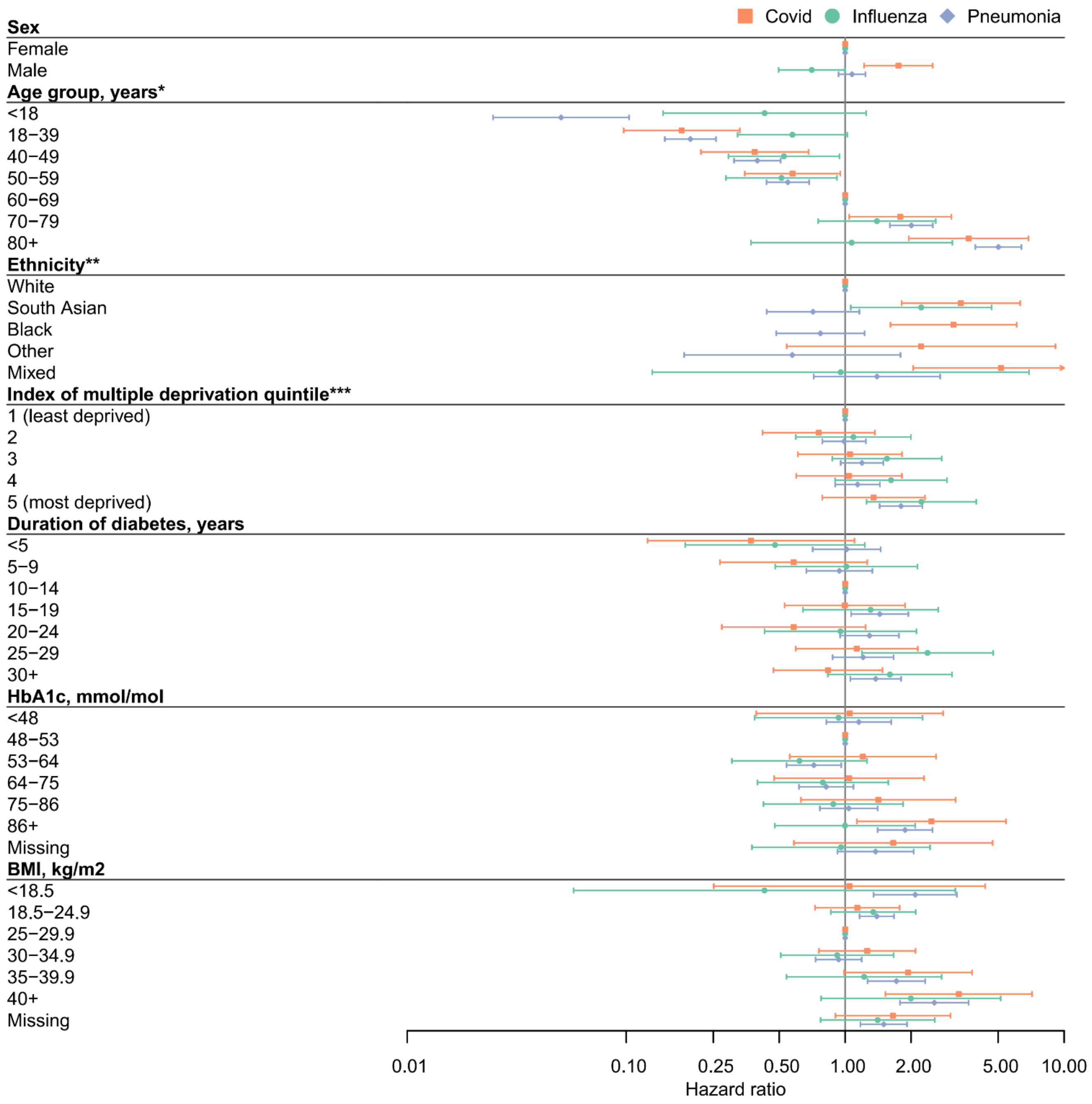

*No Covid-19 hospitalisations occurred in <18 group. **No influenza hospitalisation occurred in Black or other ethnicity groups. No Covid-19, influenza or pneumonia hospitalisation occurred in the unknown ethnicity group. *** No Covid-19, influenza or pneumonia hospitalisations occurred in missing IMD group.

For peer review only - http://bmjopen.bmj.com/site/about/guidelines.xhtml

Supplemental table 9. Baseline characteristics and Covid-19, influenza, and pneumonia hospitalisations in type 2 diabetes, restricting hospitalisation definition to primary diagnosis in HES.

	2020 cohort	Covid-19 hospitalisations (in 2020 cohort)	2016 cohort	Influenza hospitalisations (in 2016 cohort)	Pneumonia hospitalisations (in 2016 cohort)
**Number of individuals**	584854	4781	585289	2189	24944
**Mean (SD) follow-up, days**		265.3 (37.7)		915.7 (224.3)	903.4 (239.5)
**Sex**					
Female	253360 (43.3)	1802 (37.7)	256656 (43.9)	1068 (48.8)	11337 (45.4)
Male	331494 (56.7)	2979 (62.3)	328633 (56.1)	1121 (51.2)	13607 (54.6)
**Age group, years**					
<40	12898 (2.2)	61 (1.3)	13468 (2.3)	28 (1.3)	111 (0.4)
40-49	46075 (7.9)	220 (4.6)	49649 (8.5)	93 (4.2)	453 (1.8)
50-59	114537 (19.6)	625 (13.1)	113285 (19.4)	216 (9.9)	1599 (6.4)
60-69	151493 (25.9)	976 (20.4)	153782 (26.3)	456 (20.8)	3900 (15.6)
70-79	155424 (26.6)	1312 (27.4)	152401 (26.0)	732 (33.4)	7893 (31.6)
80-89	89871 (15.4)	1309 (27.4)	88492 (15.1)	559 (25.5)	8788 (35.2)
90+	14556 (2.5)	278 (5.8)	14212 (2.4)	105 (4.8)	2200 (8.8)
**Ethnicity**					
White	445160 (76.1)	3240 (67.8)	457714 (78.2)	1746 (79.8)	21923 (87.9)
South Asian	77253 (13.2)	766 (16.0)	71050 (12.1)	282 (12.9)	1871 (7.5)
Black	35144 (6.0)	575 (12.0)	33175 (5.7)	105 (4.8)	771 (3.1)
Other	9886 (1.7)	132 (2.8)	8178 (1.4)	25 (1.1)	190 (0.8)
Mixed	6267 (1.1)	56 (1.2)	5654 (1.0)	25 (1.1)	149 (0.6)
Unknown	11144 (1.9)	12 (0.3)	9518 (1.6)	6 (0.3)	40 (0.2)
**Index of multiple deprivation quintile**					
1 (least deprived)	102950 (17.6)	607 (12.7)	103184 (17.6)	332 (15.2)	4085 (16.4)
2	106968 (18.3)	715 (15.0)	109006 (18.6)	368 (16.8)	4559 (18.3)
3	113454 (19.4)	861 (18.0)	113661 (19.4)	422 (19.3)	4765 (19.1)
4	127048 (21.7)	1186 (24.8)	125492 (21.4)	477 (21.8)	5330 (21.4)
5 (most deprived)	134146 (22.9)	1410 (29.5)	133563 (22.8)	589 (26.9)	6190 (24.8)
Missing	288 (0.0)	2 (0.0)	383 (0.1)	1 (0.0)	15 (0.1)
**Duration of diagnosed diabetes, years**					
<1	14311 (2.4)	60 (1.3)	36503 (6.2)	98 (4.5)	953 (3.8)
1-2	60219 (10.3)	337 (7.0)	77510 (13.2)	216 (9.9)	2247 (9.0)
3-5	105094 (18.0)	652 (13.6)	111199 (19.0)	334 (15.3)	3429 (13.7)
6-9	128817 (22.0)	927 (19.4)	125083 (21.4)	428 (19.6)	4686 (18.8)
10-14	124471 (21.3)	967 (20.2)	126421 (21.6)	526 (24.0)	6378 (25.6)
15-19	89704 (15.3)	972 (20.3)	62373 (10.7)	302 (13.8)	3652 (14.6)
20+	62238 (10.6)	866 (18.1)	46200 (7.9)	285 (13.0)	3599 (14.4)
**HbA1c, mmol/mol**					
<48	167739 (28.7)	1327 (27.8)	175659 (30.0)	634 (29.0)	8049 (32.3)
48-53	116015 (19.8)	840 (17.6)	116131 (19.8)	392 (17.9)	4701 (18.8)
53-64	147209 (25.2)	1093 (22.9)	142662 (24.4)	532 (24.3)	5861 (23.5)
64-75	66071 (11.3)	618 (12.9)	62291 (10.6)	289 (13.2)	2606 (10.4)
75-86	33970 (5.8)	335 (7.0)	32257 (5.5)	127 (5.8)	1422 (5.7)
86+	39727 (6.8)	467 (9.8)	37470 (6.4)	165 (7.5)	1676 (6.7)
Missing	14123 (2.4)	101 (2.1)	18819 (3.2)	50 (2.3)	629 (2.5)
**Number of microvascular complications**					
0	264011 (45.1)	1550 (32.4)	281811 (48.1)	761 (34.8)	7915 (31.7)
1	216115 (37.0)	1718 (35.9)	204550 (34.9)	836 (38.2)	9536 (38.2)
2	96948 (16.6)	1261 (26.4)	91671 (15.7)	526 (24.0)	6617 (26.5)
3	7780 (1.3)	252 (5.3)	7257 (1.2)	66 (3.0)	876 (3.5)
**BMI, kg/m2**					
<18.5	3026 (0.5)	48 (1.0)	2801 (0.5)	12 (0.5)	362 (1.5)
18.5-24.9	86199 (14.7)	752 (15.7)	79018 (13.5)	310 (14.2)	4694 (18.8)
25-29.9	184626 (31.6)	1418 (29.7)	178501 (30.5)	614 (28.0)	7025 (28.2)
30-34.9	151732 (25.9)	1100 (23.0)	151161 (25.8)	551 (25.2)	5329 (21.4)
35-39.9	74766 (12.8)	612 (12.8)	75339 (12.9)	289 (13.2)	2674 (10.7)
40+	48455 (8.3)	441 (9.2)	48685 (8.3)	195 (8.9)	1857 (7.4)
Missing	36050 (6.2)	410 (8.6)	49784 (8.5)	218 (10.0)	3003 (12.0)
**Smoking status**					
Active smoker	87220 (14.9)	403 (8.4)	90218 (15.4)	364 (16.6)	4168 (16.7)
Ex-smoker	336719 (57.6)	3124 (65.3)	316723 (54.1)	1266 (57.8)	14903 (59.7)
Non-smoker	138840 (23.7)	1035 (21.6)	149524 (25.5)	436 (19.9)	4558 (18.3)
Unknown	22075 (3.8)	219 (4.6)	28824 (4.9)	123 (5.6)	1315 (5.3)
**Comorbidities**					
**Cardiovascular**					
Hypertension	425278 (72.7)	4017 (84.0)	419645 (71.7)	1818 (83.1)	21575 (86.5)
Atrial fibrillation	70633 (12.1)	1078 (22.5)	63872 (10.9)	473 (21.6)	6940 (27.8)
Angina	87970 (15.0)	1215 (25.4)	91026 (15.6)	596 (27.2)	7284 (29.2)
Previous myocardial infarction	63738 (10.9)	983 (20.6)	60576 (10.3)	422 (19.3)	5472 (21.9)
Previous cardiac revascularisation	57332 (9.8)	742 (15.5)	54961 (9.4)	317 (14.5)	3853 (15.4)
Other ischaemic heart disease	121776 (20.8)	1669 (34.9)	121753 (20.8)	766 (35.0)	9695 (38.9)

For peer review only - http://bmjopen.bmj.com/site/about/guidelines.xhtml

	2020 cohort	Covid-19 hospitalisations (in 2020 cohort)	2016 cohort	Influenza hospitalisations (in 2016 cohort)	Pneumonia hospitalisations (in 2016 cohort)
Heart failure	61014 (10.4)	1243 (26.0)	53579 (9.2)	469 (21.4)	6932 (27.8)
Peripheral arterial disease	50045 (8.6)	895 (18.7)	48489 (8.3)	301 (13.8)	4970 (19.9)
**Recent hospitalisation**					
Respiratory infection	25535 (4.4)	817 (17.1)	21353 (3.6)	280 (12.8)	4469 (17.9)
Anything else	96421 (16.5)	1890 (39.5)	95232 (16.3)	794 (36.3)	10039 (40.2)
**Respiratory**					
Asthma	125582 (21.5)	1275 (26.7)	118207 (20.2)	701 (32.0)	7866 (31.5)
Chronic obstructive pulmonary disease	65615 (11.2)	1022 (21.4)	60108 (10.3)	534 (24.4)	8088 (32.4)
**Neurological**					
Previous transient ischaemic attack	31024 (5.3)	463 (9.7)	29997 (5.1)	203 (9.3)	2812 (11.3)
Previous stroke	49873 (8.5)	763 (16.0)	47721 (8.2)	341 (15.6)	4574 (18.3)
Dementia	20629 (3.5)	573 (12.0)	19548 (3.3)	149 (6.8)	2611 (10.5)
Other neurological condition	35000 (6.0)	584 (12.2)	33569 (5.7)	240 (11.0)	3309 (13.3)
**Oncological**					
Haematological cancer	11751 (2.0)	162 (3.4)	10352 (1.8)	97 (4.4)	990 (4.0)
Solid cancer	79179 (13.5)	855 (17.9)	72001 (12.3)	341 (15.6)	5206 (20.9)
**Other**					
Solid organ transplant	3682 (0.6)	82 (1.7)	3013 (0.5)	32 (1.5)	332 (1.3)
Chronic liver disease	69136 (11.8)	670 (14.0)	48011 (8.2)	210 (9.6)	2142 (8.6)
**Chronic Kidney Disease (CKD) stage**					
Stage 1	165845 (28.4)	790 (16.5)	147798 (25.3)	299 (13.7)	2502 (10.0)
Stage 2	286696 (49.0)	1940 (40.6)	290821 (49.7)	971 (44.4)	10121 (40.6)
Stage 3a	72539 (12.4)	841 (17.6)	80222 (13.7)	453 (20.7)	5273 (21.1)
Stage 3b	33246 (5.7)	544 (11.4)	37256 (6.4)	237 (10.8)	3818 (15.3)
Stage 4	9006 (1.5)	230 (4.8)	10490 (1.8)	88 (4.0)	1473 (5.9)
Stage 5	12871 (2.2)	421 (8.8)	13365 (2.3)	130 (5.9)	1629 (6.5)
Missing	4651 (0.8)	15 (0.3)	5337 (0.9)	11 (0.5)	128 (0.5)
**Albumin creatinine ratio (ACR) category**					
A1	402847 (68.9)	2655 (55.5)	388839 (66.4)	1252 (57.2)	13184 (52.9)
A2	121002 (20.7)	1339 (28.0)	106879 (18.3)	565 (25.8)	7450 (29.9)
A3	21899 (3.7)	495 (10.4)	18817 (3.2)	153 (7.0)	2033 (8.2)
Missing	39106 (6.7)	292 (6.1)	70754 (12.1)	219 (10.0)	2277 (9.1)
**Diabetes treatment (last 6 months)**					
Insulin prescription	79286 (13.6)	1190 (24.9)	78755 (13.5)	468 (21.4)	5380 (21.6)
OHA prescription only	362651 (62.0)	2584 (54.0)	349629 (59.7)	1215 (55.5)	12993 (52.1)
No treatment	142917 (24.4)	1007 (21.1)	156905 (26.8)	506 (23.1)	6571 (26.3)

For peer review only

Supplemental figure 8. Association of potential risk factors with hospitalisation for Covid-19 (2020), influenza (2016-2019), and pneumonia (2016-2019) in type 2 diabetes, restricting hospitalisation definition to primary diagnosis in HES.

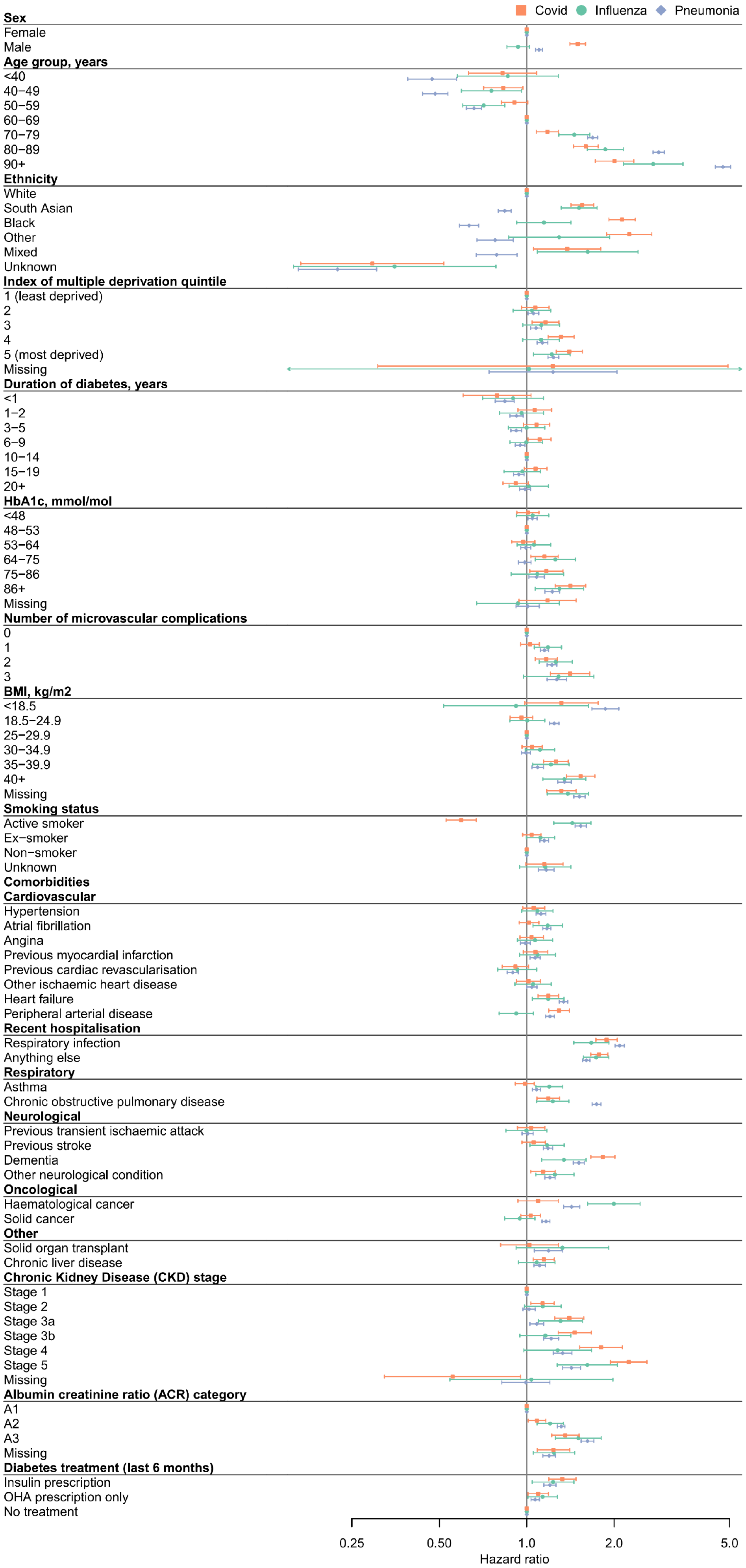

For peer review only - http://bmjopen.bmj.com/site/about/guidelines.xhtml

Supplemental table 10. Baseline characteristics and Covid-19 and pneumonia deaths in type 2 diabetes, restricting mortality definition to primary cause in ONS.

	2020 cohort	Covid-19 deaths (in 2020 cohort)	2016 cohort	Pneumonia deaths (in 2016 cohort)
**Number of individuals**	584854	2086	585289	2653
**Mean (SD) follow-up, days**		266.1 (35.5)		917.0 (223.2)
**Sex**				
Female	253360 (43.3)	796 (38.2)	256656 (43.9)	1268 (47.8)
Male	331494 (56.7)	1290 (61.8)	328633 (56.1)	1385 (52.2)
**Age group, years**				
<40	12898 (2.2)	6 (0.3)	13468 (2.3)	1 (0.0)
40-49	46075 (7.9)	30 (1.4)	49649 (8.5)	23 (0.9)
50-59	114537 (19.6)	118 (5.7)	113285 (19.4)	52 (2.0)
60-69	151493 (25.9)	299 (14.3)	153782 (26.3)	199 (7.5)
70-79	155424 (26.6)	563 (27.0)	152401 (26.0)	566 (21.3)
80-89	89871 (15.4)	847 (40.6)	88492 (15.1)	1227 (46.2)
90+	14556 (2.5)	223 (10.7)	14212 (2.4)	585 (22.1)
**Ethnicity**				
White	445160 (76.1)	1514 (72.6)	457714 (78.2)	2399 (90.4)
South Asian	77253 (13.2)	289 (13.9)	71050 (12.1)	160 (6.0)
Black	35144 (6.0)	216 (10.4)	33175 (5.7)	55 (2.1)
Other	9886 (1.7)	42 (2.0)	8178 (1.4)	20 (0.8)
Mixed	6267 (1.1)	18 (0.9)	5654 (1.0)	10 (0.4)
Unknown	11144 (1.9)	7 (0.3)	9518 (1.6)	9 (0.3)
**Index of multiple deprivation quintile**				
1 (least deprived)	102950 (17.6)	281 (13.5)	103184 (17.6)	493 (18.6)
2	106968 (18.3)	330 (15.8)	109006 (18.6)	490 (18.5)
3	113454 (19.4)	397 (19.0)	113661 (19.4)	525 (19.8)
4	127048 (21.7)	475 (22.8)	125492 (21.4)	550 (20.7)
5 (most deprived)	134146 (22.9)	602 (28.9)	133563 (22.8)	595 (22.4)
Missing	288 (0.0)	1 (0.0)	383 (0.1)	0 (0.0)
**Duration of diagnosed diabetes, years**				
<1	14311 (2.4)	10 (0.5)	36503 (6.2)	53 (2.0)
1-2	60219 (10.3)	93 (4.5)	77510 (13.2)	157 (5.9)
3-5	105094 (18.0)	257 (12.3)	111199 (19.0)	267 (10.1)
6-9	128817 (22.0)	343 (16.4)	125083 (21.4)	452 (17.0)
10-14	124471 (21.3)	443 (21.2)	126421 (21.6)	732 (27.6)
15-19	89704 (15.3)	490 (23.5)	62373 (10.7)	498 (18.8)
20+	62238 (10.6)	450 (21.6)	46200 (7.9)	494 (18.6)
**HbA1c, mmol/mol**				
<48	167739 (28.7)	655 (31.4)	175659 (30.0)	913 (34.4)
48-53	116015 (19.8)	351 (16.8)	116131 (19.8)	498 (18.8)
53-64	147209 (25.2)	462 (22.1)	142662 (24.4)	612 (23.1)
64-75	66071 (11.3)	266 (12.8)	62291 (10.6)	259 (9.8)
75-86	33970 (5.8)	134 (6.4)	32257 (5.5)	135 (5.1)
86+	39727 (6.8)	156 (7.5)	37470 (6.4)	150 (5.7)
Missing	14123 (2.4)	62 (3.0)	18819 (3.2)	86 (3.2)
**Number of microvascular complications**				
0	264011 (45.1)	564 (27.0)	281811 (48.1)	641 (24.2)
1	216115 (37.0)	772 (37.0)	204550 (34.9)	1009 (38.0)
2	96948 (16.6)	629 (30.2)	91671 (15.7)	884 (33.3)
3	7780 (1.3)	121 (5.8)	7257 (1.2)	119 (4.5)
**BMI, kg/m2**				
<18.5	3026 (0.5)	36 (1.7)	2801 (0.5)	55 (2.1)
18.5-24.9	86199 (14.7)	466 (22.3)	79018 (13.5)	664 (25.0)
25-29.9	184626 (31.6)	597 (28.6)	178501 (30.5)	726 (27.4)
30-34.9	151732 (25.9)	418 (20.0)	151161 (25.8)	395 (14.9)
35-39.9	74766 (12.8)	197 (9.4)	75339 (12.9)	183 (6.9)
40+	48455 (8.3)	125 (6.0)	48685 (8.3)	138 (5.2)
Missing	36050 (6.2)	247 (11.8)	49784 (8.5)	492 (18.5)
**Smoking status**				
Active smoker	87220 (14.9)	179 (8.6)	90218 (15.4)	320 (12.1)
Ex-smoker	336719 (57.6)	1377 (66.0)	316723 (54.1)	1566 (59.0)
Non-smoker	138840 (23.7)	441 (21.1)	149524 (25.5)	630 (23.7)
Unknown	22075 (3.8)	89 (4.3)	28824 (4.9)	137 (5.2)
**Comorbidities**				
**Cardiovascular**				
Hypertension	425278 (72.7)	1853 (88.8)	419645 (71.7)	2402 (90.5)
Atrial fibrillation	70633 (12.1)	612 (29.3)	63872 (10.9)	946 (35.7)
Angina	87970 (15.0)	599 (28.7)	91026 (15.6)	812 (30.6)
Previous myocardial infarction	63738 (10.9)	497 (23.8)	60576 (10.3)	650 (24.5)
Previous cardiac revascularisation	57332 (9.8)	363 (17.4)	54961 (9.4)	435 (16.4)
Other ischaemic heart disease	121776 (20.8)	799 (38.3)	121753 (20.8)	1099 (41.4)
Heart failure	61014 (10.4)	658 (31.5)	53579 (9.2)	965 (36.4)

For peer review only - http://bmjopen.bmj.com/site/about/guidelines.xhtml

	2020 cohort	Covid-19 deaths (in 2020 cohort)	2016 cohort	Pneumonia deaths (in 2016 cohort)
Peripheral arterial disease	50045 (8.6)	492 (23.6)	48489 (8.3)	693 (26.1)
**Recent hospitalisation**				
Respiratory infection	25535 (4.4)	439 (21.0)	21353 (3.6)	483 (18.2)
Anything else	96421 (16.5)	911 (43.7)	95232 (16.3)	1117 (42.1)
**Respiratory**				
Asthma	125582 (21.5)	543 (26.0)	118207 (20.2)	507 (19.1)
Chronic obstructive pulmonary disease	65615 (11.2)	502 (24.1)	60108 (10.3)	458 (17.3)
**Neurological**				
Previous transient ischaemic attack	31024 (5.3)	265 (12.7)	29997 (5.1)	325 (12.3)
Previous stroke	49873 (8.5)	457 (21.9)	47721 (8.2)	476 (17.9)
Dementia	20629 (3.5)	477 (22.9)	19548 (3.3)	274 (10.3)
Other neurological condition	35000 (6.0)	362 (17.4)	33569 (5.7)	395 (14.9)
**Oncological**				
Haematological cancer	11751 (2.0)	89 (4.3)	10352 (1.8)	62 (2.3)
Solid cancer	79179 (13.5)	412 (19.8)	72001 (12.3)	488 (18.4)
**Other**				
Solid organ transplant	3682 (0.6)	27 (1.3)	3013 (0.5)	47 (1.8)
Chronic liver disease	69136 (11.8)	261 (12.5)	48011 (8.2)	140 (5.3)
**Chronic Kidney Disease (CKD) stage**				
Stage 1	165845 (28.4)	191 (9.2)	147798 (25.3)	128 (4.8)
Stage 2	286696 (49.0)	760 (36.4)	290821 (49.7)	791 (29.8)
Stage 3a	72539 (12.4)	445 (21.3)	80222 (13.7)	595 (22.4)
Stage 3b	33246 (5.7)	336 (16.1)	37256 (6.4)	561 (21.1)
Stage 4	9006 (1.5)	147 (7.0)	10490 (1.8)	295 (11.1)
Stage 5	12871 (2.2)	195 (9.3)	13365 (2.3)	260 (9.8)
Missing	4651 (0.8)	12 (0.6)	5337 (0.9)	23 (0.9)
**Albumin creatinine ratio (ACR) category**				
A1	402847 (68.9)	1013 (48.6)	388839 (66.4)	1197 (45.1)
A2	121002 (20.7)	647 (31.0)	106879 (18.3)	961 (36.2)
A3	21899 (3.7)	251 (12.0)	18817 (3.2)	314 (11.8)
Missing	39106 (6.7)	175 (8.4)	70754 (12.1)	181 (6.8)
**Diabetes treatment (last 6 months)**				
Insulin prescription	79286 (13.6)	536 (25.7)	78755 (13.5)	581 (21.9)
OHA prescription only	362651 (62.0)	1012 (48.5)	349629 (59.7)	1307 (49.3)
No treatment	142917 (24.4)	538 (25.8)	156905 (26.8)	765 (28.8)

For peer review only

Supplemental figure 9. Association of potential risk factors with Covid-19 (2020), and pneumonia (2016-2019) mortality in type 2 diabetes, restricting mortality definition to primary cause in ONS.

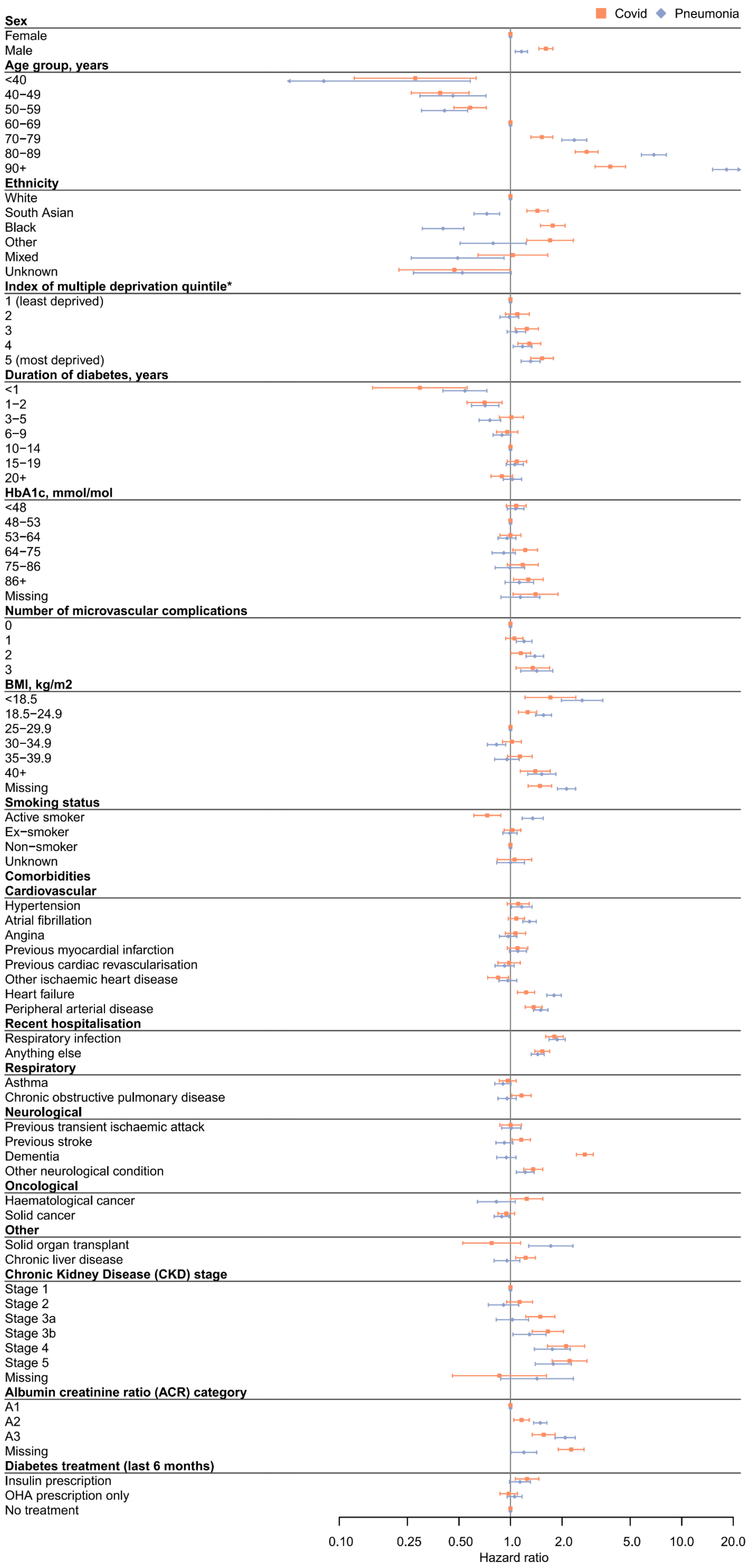

* No pneumonia deaths occurred in missing IMD group. 1 Covid-19 death occurred in missing IMD group.

Supplemental figure 10. Association of potential risk factors with pneumonia hospitalisation (2016-2019) in those with a pneumococcal vaccination and those without.

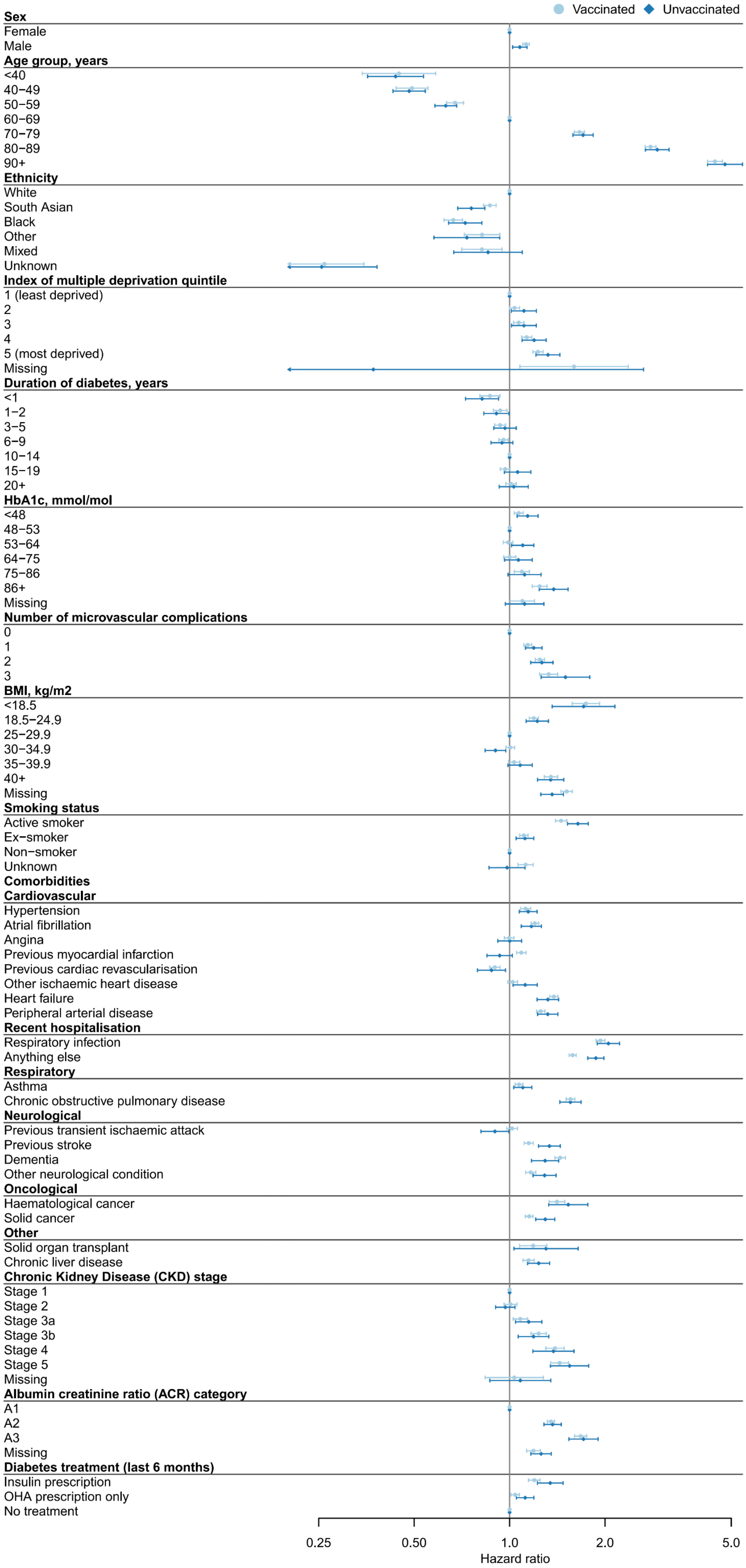

Supplemental figure 11. Association of potential risk factors with influenza hospitalisation (2016-2019) in those with an influenza vaccination and those without.

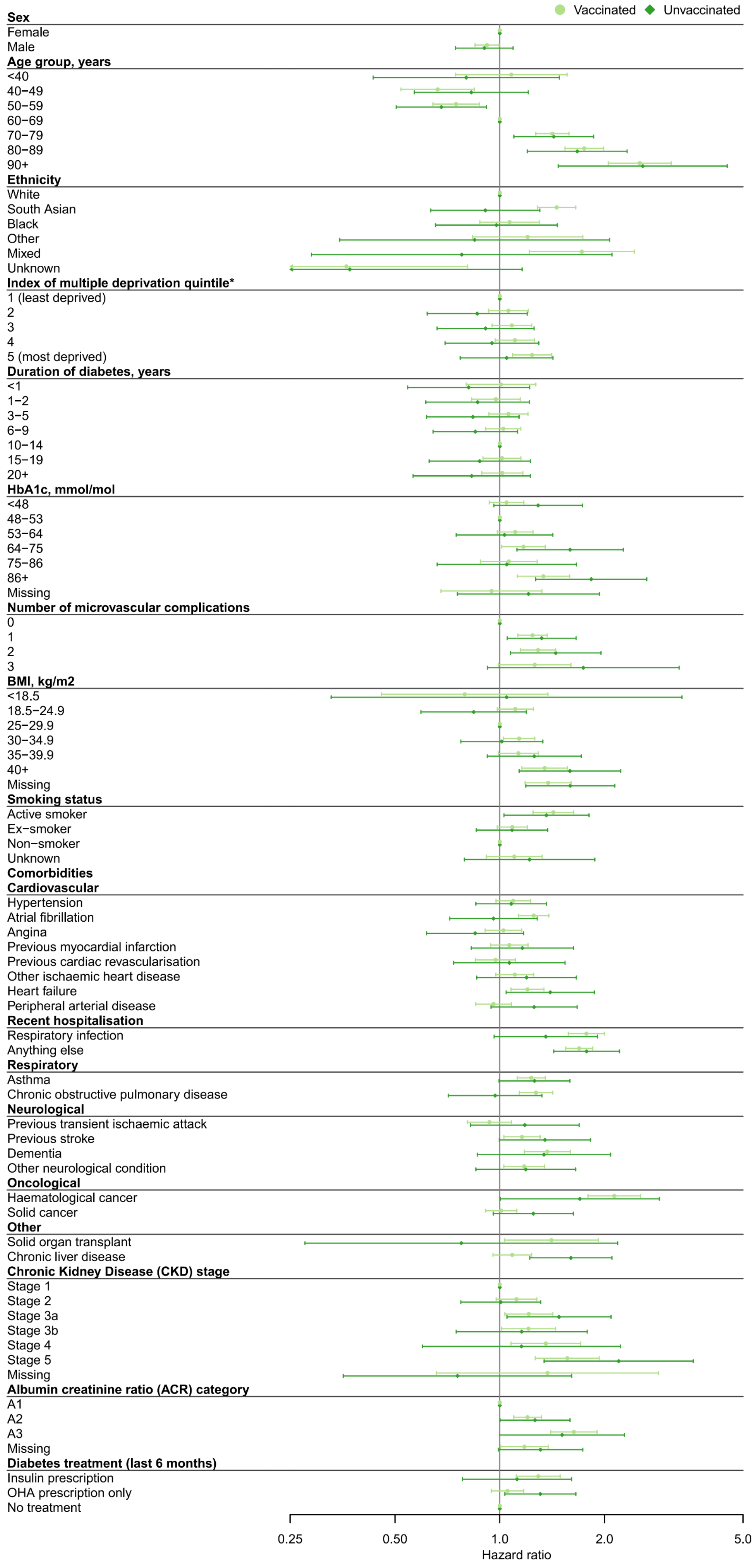

* No influenza hospitalisations occurred in the missing IMD group.

References

1. Mathur R, Bhaskaran K, Chaturvedi N, et al. Completeness and usability of ethnicity data in UK-based primary care and hospital databases. J Public Health (Oxf) 2014;36(4): 684-92. doi: 10.1093/pubmed/fdt116

2. GOV.UK. English indices of deprivation 2015. Available: https://www.gov.uk/government/statistics/english-indices-of-deprivation-2015 [Accessed 4 January 2023]

3. World Health Organisation. A healthy lifestyle - WHO recommendations. 2010. Available from https://www.who.int/europe/news-room/fact-sheets/item/a-healthy-lifestyle---who-recommendations [Accessed 4 January 2023]

4. Rodgers LR, Weedon MN, Henley WE, et al. Cohort profile for the MASTERMIND study: using the Clinical Practice Research Datalink (CPRD) to investigate stratification of response to treatment in patients with type 2 diabetes. BMJ Open 2017;7(10): e017989. doi: 10.1136/bmjopen-2017-017989

5. KDIGO. CKD Evaluation and Management. 2012. Available from https://kdigo.org/guidelines/ckd-evaluation-and-management/ [Accessed 4 January 2023]

6. Clift AK, Coupland CAC, Keogh RH, et al. Living risk prediction algorithm (QCOVID) for risk of hospital admission and mortality from coronavirus 19 in adults: national derivation and validation cohort study. BMJ 2020;371: m3731. doi: 10.1136/bmj.m3731

For peer review only

The RECORD statement – checklist of items, extended from the STROBE statement, that should be reported in observational studies using routinely collected health data.

	Item No.	STROBE items	Location in manuscript where items are reported	RECORD items	Location in manuscript where items are reported
**Title and abstract**					
	1	(a) Indicate the study's design with a commonly used term in the title or the abstract (b) Provide in the abstract an informative and balanced summary of what was done and what was found	a) Study design given in the title and abstract methods section (page 2).<br>b) Summary of the study given in abstract methods section (page 2).	RECORD 1.1: The type of data used should be specified in the title or abstract. When possible, the name of the databases used should be included.<br><br>RECORD 1.2: If applicable, the geographic region and timeframe within which the study took place should be reported in the title or abstract.<br><br>RECORD 1.3: If linkage between databases was conducted for the study, this should be clearly stated in the title or abstract.	Type of data and dataset name given in abstract (page 2).<br><br>Study periods given in abstract (page 2).<br><br>Linkage stated in abstract (page 2).
**Introduction**					
Background rationale	2	Explain the scientific background and rationale for the investigation being reported	Background and rationale are covered in the introduction (page 4).		
Objectives	3	State specific objectives, including any prespecified hypotheses	Aims are stated at the end of introduction (page 4).		
**Methods**					
Study Design	4	Present key elements of study design early in the paper	Study design is reported in the methods (page 5).		
Setting	5	Describe the setting, locations, and relevant dates, including	Data source and study population		

		periods of recruitment, exposure, follow-up, and data collection	sections of methods cover the setting/location and periods of follow-up (page 5).		
Participants	6	*(a) Cohort study* - Give the eligibility criteria, and the sources and methods of selection of participants. Describe methods of follow-up<br>*Case-control study* - Give the eligibility criteria, and the sources and methods of case ascertainment and control selection. Give the rationale for the choice of cases and controls<br>*Cross-sectional study* - Give the eligibility criteria, and the sources and methods of selection of participants<br><br>*(b) Cohort study* - For matched studies, give matching criteria and number of exposed and unexposed<br>*Case-control study* - For matched studies, give matching criteria and the number of controls per case	a) Study population section of methods gives inclusion criteria for participants (flow diagram included as Supplemental figure 1) and follow-up described (page 5).	RECORD 6.1: The methods of study population selection (such as codes or algorithms used to identify subjects) should be listed in detail. If this is not possible, an explanation should be provided.<br><br>RECORD 6.2: Any validation studies of the codes or algorithms used to select the population should be referenced. If validation was conducted for this study and not published elsewhere, detailed methods and results should be provided.<br><br>RECORD 6.3: If the study involved linkage of databases, consider use of a flow diagram or other graphical display to demonstrate the data linkage process, including the number of individuals with linked data at each stage.	Algorithm for selection of diabetes population briefly described in study population section of methods (page 5) and full details available on our github (https://github.com/Exeter-Diabetes/CPRD-Codelists).<br><br>Study confirming accuracy of algorithm referenced (page 5).<br><br>CPRD performed data linkage, number of participants with linked data given in flow diagram (Supplemental figure 1)
Variables	7	Clearly define all outcomes, exposures, predictors, potential confounders, and effect	All outcomes defined in outcomes section of methods (pages 5-6). All	RECORD 7.1: A complete list of codes and algorithms used to classify exposures, outcomes, confounders, and effect modifiers should be provided. If	Codes and algorithms used for all exposures can be found on

For peer review only

		modifiers. Give diagnostic criteria, if applicable.	exposures defined in risk factors and covariates section (page 6) and Supplemental table 2.	these cannot be reported, an explanation should be provided.	our github (https://github.com/Exeter-Diabetes/CPRD-Codelists), codes used to define outcomes in Supplemental table 1.
Data sources/ measurement	8	For each variable of interest, give sources of data and details of methods of assessment (measurement). Describe comparability of assessment methods if there is more than one group	Sources of data and measurement of variables given in Supplemental table 2.		
Bias	9	Describe any efforts to address potential sources of bias	Covered in methods (pages 5-7).		
Study size	10	Explain how the study size was arrived at	Not applicable.		
Quantitative variables	11	Explain how quantitative variables were handled in the analyses. If applicable, describe which groupings were chosen, and why	Supplemental table 2 describes how quantitative variables were handled/ grouped.		
Statistical methods	12	(a) Describe all statistical methods, including those used to control for confounding<br>(b) Describe any methods used to examine subgroups and interactions<br>(c) Explain how missing data were addressed<br>(d) *Cohort study* - If applicable, explain how loss to follow-up was addressed<br>*Case-control study* - If applicable, explain how	Statistical analysis section of methods describes all statistical methods used and methods used for subgroup analysis and sensitivity analysis (pages 6-7). Missing data methods described in methods (page 6)		

For peer review only

		matching of cases and controls was addressed<br>*Cross-sectional study* - If applicable, describe analytical methods taking account of sampling strategy<br>(e) Describe any sensitivity analyses	and Supplemental table 2.		
Data access and cleaning methods		..		RECORD 12.1: Authors should describe the extent to which the investigators had access to the database population used to create the study population.<br><br>RECORD 12.2: Authors should provide information on the data cleaning methods used in the study.	Some authors had access to raw data (stated in acknowledgments – page 15)<br><br>Algorithms used for cleaning and deriving variables given on github
Linkage		..		RECORD 12.3: State whether the study included person-level, institutional-level, or other data linkage across two or more databases. The methods of linkage and methods of linkage quality evaluation should be provided.	CPRD performed linkage of datasets.
**Results**					
Participants	13	(a) Report the numbers of individuals at each stage of the study (*e.g.*, numbers potentially eligible, examined for eligibility, confirmed eligible, included in the study, completing follow-up, and analysed)<br>(b) Give reasons for non-participation at each stage.<br>(c) Consider use of a flow diagram	Study participation flow diagram given as Supplemental figure 1.	RECORD 13.1: Describe in detail the selection of the persons included in the study (*i.e.,* study population selection) including filtering based on data quality, data availability and linkage. The selection of included persons can be described in the text and/or by means of the study flow diagram.	Study flow diagram (Supplemental figure 1) gives detailed summary of selection of participants for study.

For peer review only

Descriptive data	14	(a) Give characteristics of study participants (*e.g.*, demographic, clinical, social) and information on exposures and potential confounders<br>(b) Indicate the number of participants with missing data for each variable of interest<br>(c) *Cohort study* - summarise follow-up time (*e.g.*, average and total amount)	Baseline characteristics of study participants and mean follow-up time given in Table 1 (pages 8-9) and Supplemental table 4. Missing data category included for each variable where applicable.		
Outcome data	15	*Cohort study* - Report numbers of outcome events or summary measures over time<br>*Case-control study* - Report numbers in each exposure category, or summary measures of exposure<br>*Cross-sectional study* - Report numbers of outcome events or summary measures	Numbers of outcome events reported in results section of text (pages 8-11) and in Table 1 (pages 8-9).		
Main results	16	(a) Give unadjusted estimates and, if applicable, confounder-adjusted estimates and their precision (e.g., 95% confidence interval). Make clear which confounders were adjusted for and why they were included<br>(b) Report category boundaries when continuous variables were categorized<br>(c) If relevant, consider translating estimates of relative risk into absolute risk for a meaningful time period	Confounder adjusted estimates with 95% confidence intervals for all risk factors given in Supplemental table 5. Category boundaries given in all relevant tables and plots.		
Other analyses	17	Report other analyses done—e.g., analyses of subgroups and	Results from subgroup analysis and sensitivity		

For peer review only

		interactions, and sensitivity analyses	analyses reported (pages 10-11).		
**Discussion**					
Key results	18	Summarise key results with reference to study objectives	Key results are summarised at the beginning of the discussion section (page 12).		
Limitations	19	Discuss limitations of the study, taking into account sources of potential bias or imprecision. Discuss both direction and magnitude of any potential bias	Limitations of this study were discussed in the strengths and limitations section of the discussion (page 13).	RECORD 19.1: Discuss the implications of using data that were not created or collected to answer the specific research question(s). Include discussion of misclassification bias, unmeasured confounding, missing data, and changing eligibility over time, as they pertain to the study being reported.	Implications of using this data discussed in limitations (page 13).
Interpretation	20	Give a cautious overall interpretation of results considering objectives, limitations, multiplicity of analyses, results from similar studies, and other relevant evidence	Interpretation of results is given in the clinical implications section (14).		
Generalisability	21	Discuss the generalisability (external validity) of the study results	Study data largely representative of UK population (page 5).		
**Other Information**					
Funding	22	Give the source of funding and the role of the funders for the present study and, if applicable, for the original study on which the present article is based	Details of funding given in acknowledgments (page 15).		
Accessibility of protocol, raw data, and programming code		..		RECORD 22.1: Authors should provide information on how to access any supplemental information such as the study protocol, raw data, or programming code.	'CPRD data are available by application to CPRD Independent

For peer review only

					Scientific Advisory Committee' (page 16)<br>Code will be available on github.

*Reference: Benchimol EI, Smeeth L, Guttmann A, Harron K, Moher D, Petersen I, Sørensen HT, von Elm E, Langan SM, the RECORD Working Committee. The REporting of studies Conducted using Observational Routinely-collected health Data (RECORD) Statement. *PLoS Medicine* 2015; in press.

*Checklist is protected under Creative Commons Attribution (CC BY) license.

For peer review only
